# Climate-driven global redistribution of an ocean giant predicts increased threat from shipping

**DOI:** 10.1038/s41558-024-02129-5

**Published:** 2024-10-07

**Authors:** Freya C. Womersley, Lara L. Sousa, Nicolas E. Humphries, Kátya Abrantes, Gonzalo Araujo, Steffen S. Bach, Adam Barnett, Michael L. Berumen, Sandra Bessudo Lion, Camrin D. Braun, Elizabeth Clingham, Jesse E. M. Cochran, Rafael de la Parra, Stella Diamant, Alistair D. M. Dove, Carlos M. Duarte, Christine L. Dudgeon, Mark V. Erdmann, Eduardo Espinoza, Luciana C. Ferreira, Richard Fitzpatrick, Jaime González Cano, Jonathan R. Green, Hector M. Guzman, Royale Hardenstine, Abdi Hasan, Fábio H. V. Hazin, Alex R. Hearn, Robert E. Hueter, Mohammed Y. Jaidah, Jessica Labaja, Felipe Ladino, Bruno C. L. Macena, Mark G. Meekan, John J. Morris, Bradley M. Norman, Cesar R. Peñaherrera-Palma, Simon J. Pierce, Lina Maria Quintero, Dení Ramírez-Macías, Samantha D. Reynolds, David P. Robinson, Christoph A. Rohner, David R. L. Rowat, Ana M. M. Sequeira, Marcus Sheaves, Mahmood S. Shivji, Abraham B. Sianipar, Gregory B. Skomal, German Soler, Ismail Syakurachman, Simon R. Thorrold, Michele Thums, John P. Tyminski, D. Harry Webb, Bradley M. Wetherbee, Nuno Queiroz, David W. Sims

**Affiliations:** 1 Marine Biological Association, The Laboratory, Plymouth, UK; 2 Ocean and Earth Science, National Oceanography Centre Southampton, University of Southampton, Southampton, UK; 3 Wildlife Conservation Research Unit, Department of Zoology, University of Oxford, Tubney, UK; 4 College of Science and Engineering, James Cook University, Cairns, Queensland Australia; 5 Biopixel Oceans Foundation, Cairns, Queensland Australia; 6 Marine Data Technology Hub, James Cook University, Cairns, Queensland Australia; 7 Marine Research and Conservation Foundation, Lydeard St Lawrence, UK; 8 Environmental Science Program, Department of Biological and Environmental Sciences, College of Arts and Sciences, Qatar University, Doha, Qatar; 9 Qatar Whale Shark Research Project, Doha, Qatar; 10 Red Sea Research Center, Division of Biological and Environmental Science and Engineering, King Abdullah University of Science and Technology, Thuwal, Kingdom of Saudi Arabia; 11 Fundación Malpelo y Otros Ecosistemas Marinos, Bogotá, Colombia; 12 MigraMar, Bodega Bay, CA USA; 13 Biology Department, Woods Hole Oceanographic Institution, Woods Hole, MA USA; 14 St Helena Government, Jamestown, St Helena Island; 15 Ch’ooj Ajuail AC, Cancún, Mexico; 16 Madagascar Whale Shark Project, Nosy Be, Madagascar; 17 Georgia Aquarium, Atlanta, GA USA; 18 Marine Science Program, Division of Biological and Environmental Science and Engineering, King Abdullah University of Science and Technology, Thuwal, Kingdom of Saudi Arabia; 19 School of Biomedical Sciences, The University of Queensland, St. Lucia, Queensland Australia; 20 Conservation International New Zealand, University of Auckland, Auckland, New Zealand; 21 Dirección Parque Nacional Galapagos, Puerto Ayora, Ecuador; 22 Australian Institute of Marine Science, Indian Ocean Marine Research Centre, University of Western Australia, Crawley, Western Australia Australia; 23 Comisión Nacional de Áreas Naturales Protegidas, Cancún, México; 24 Galapagos Whale Shark Project, Puerto Ayora, Ecuador; 25 Smithsonian Tropical Research Institute, Panama, Republic of Panama; 26 Konservasi Indonesia Raja Ampat, Sorong, Indonesia; 27 Departamento de Pesca e Aquicultura, UFRPE, Recife/PE, Brazil; 28 Galapagos Science Center, Universidad San Francisco de Quito USFQ, Quito, Ecuador; 29 Mote Marine Laboratory, Sarasota, FL USA; 30 OCEARCH, Park City, UT USA; 31 Large Marine Vertebrates Research Institute Philippines, Jagna, Philippines; 32 Institute of Marine Sciences – OKEANOS, University of the Azores, Horta, Portugal; 33 Institute of Marine Research – IMAR, Department of Oceanography and Fisheries, University of the Azores, Horta, Portugal; 34 Harry Butler Institute, Murdoch University, Murdoch, Western Australia Australia; 35 ECOCEAN Inc., Serpentine, Fremantle, Western Australia Australia; 36 Marine Megafauna Foundation, West Palm Beach, FL USA; 37 University of the Sunshine Coast, Sippy Downs, Queensland Australia; 38 Solmar L5, El Centenario, La Paz, Mexico; 39 School of Biological Sciences, The University of Queensland, St Lucia, Queensland Australia; 40 Sundive Research, Byron Bay, New South Wales Australia; 41 Marine Conservation Society Seychelles, Transvaal House, Beau Vallon, Seychelles; 42 Division of Ecology and Evolution, Research School of Biology, The Australian National University, Canberra, Australian Capital Territory Australia; 43 UWA Oceans Institute and the School of Biological Sciences, The University of Western Australia, Perth, Western Australia Australia; 44 Department of Biological Sciences, The Guy Harvey Research Institute, Nova Southeastern University, Dania Beach, FL USA; 45 Elasmobranch Institute Indonesia, Denpasar, Indonesia; 46 Massachusetts Division of Marine Fisheries, New Bedford, MA USA; 47 Konservasi Indonesia, Jakarta, Indonesia; 48 Department of Biological Science, University of Rhode Island, Kingston, RI USA; 49 CIBIO, Centro de Investigação em Biodiversidade e Recursos Genéticos, InBIO Laboratório Associado, Campus de Vairão, Universidade do Porto, Vairão, Portugal; 50 BIOPOLIS Program in Genomics, Biodiversity and Land Planning, CIBIO, Campus de Vairão, Vairão, Portugal

**Keywords:** Climate-change ecology, Conservation biology, Climate-change impacts

## Abstract

Climate change is shifting animal distributions. However, the extent to which future global habitats of threatened marine megafauna will overlap existing human threats remains unresolved. Here we use global climate models and habitat suitability estimated from long-term satellite-tracking data of the world’s largest fish, the whale shark, to show that redistributions of present-day habitats are projected to increase the species’ co-occurrence with global shipping. Our model projects core habitat area losses of >50% within some national waters by 2100, with geographic shifts of over 1,000 km (∼12 km yr^−1^). Greater habitat suitability is predicted in current range-edge areas, increasing the co-occurrence of sharks with large ships. This future increase was ∼15,000 times greater under high emissions compared with a sustainable development scenario. Results demonstrate that climate-induced global species redistributions that increase exposure to direct sources of mortality are possible, emphasizing the need for quantitative climate-threat predictions in conservation assessments of endangered marine megafauna.

## Abstract (web summary)

The authors use long-term satellite tracking to project climate-induced shifts in whale shark distributions and understand their potential future risk of ship-strike. Under high-emission scenarios, the movement of sharks to current range-edge habitat is linked to 15,000-fold increased co-occurrence with ships.

## Main

Global warming is one of the most pervasive facets of human-driven climate change, with the magnitude of projected temperature rises over the 21st century comparable to that of the largest global changes in the past 65 million years^1,2^. Biological responses to warming are already apparent across terrestrial^3^, freshwater^4^ and marine taxa^5,6^. As environments change, species must either adapt, tolerate, move or face extinction^7–9^. A series of commonly articulated hypotheses have emerged in relation to movement, whereby species are expected to shift their distributions under warming to greater elevations (altitude), higher latitudes or deeper ocean depths to remain within suitable environmental conditions^10–12^.

Marine taxa, in particular, are highly responsive to temperature change, and can closely follow isotherms with fewer physical barriers to dispersal compared with their terrestrial counterparts^5,13,14^. As a result, marine species are moving poleward as much as six times faster^15^, with global redistributions projected for over 12,000 species^16^. The general expectation is that polar and temperate regions will act as ‘sinks’ and tropical regions as ‘sources’^16–20^. Profound alterations to ecosystem structure and function can result from these shifts in marine socioecological systems, ultimately impacting human communities^21^.

For highly mobile marine megafauna that can travel hundreds or thousands of kilometres annually^22^, these hypotheses have only recently begun to be addressed due to logistical difficulties in their monitoring^23^. There is some evidence for potential habitat losses, core habitat displacement and divergent responses among species with differing life histories^24,25^. However, the location of many species’ future habitats remains an open question. In addition, it remains unknown how climate-driven habitat redistribution will affect their exposure to existing anthropogenic threats such as collisions with ships^26,27^ or fishing exploitation^28^, even though such impacts may exacerbate population declines already occurring.

Ocean climate changes may shift marine megafauna into new habitats with busier shipping activity, increasing their vulnerability to collisions and potentially compounded by predicted future increases in shipping traffic^29,30^. Alternatively, habitats may shift into safer areas with less activity, providing refuge. Quantitative understandings of the interactions between wildlife movement, human activities and climate change are now needed for incorporation into conservation assessments, as well as into global strategic planning frameworks (for example, cbd.int/cop). However, global insights based on dynamic animal movements are still lacking^31^.

To address this, we tested whether a highly mobile, globally distributed marine megafauna species—the world’s largest fish, the whale shark (*Rhincodon typus*)—conforms to commonly held distributional hypotheses under climate change across its entire range (for example, ocean basin-scale poleward shifts^32^), while quantifying changes in co-occurrence with shipping. The whale shark serves as a model species to test these ideas for marine megafauna because of its circumtropical distribution and expected climate responses^32,33^, and is classified as ‘Endangered’ in the International Union for Conservation of Nature (IUCN) Red List^34^. Recent evidence suggests that the species is particularly vulnerable to ship collisions due to its extensive use of surface waters and the high overlap of its habitat with marine traffic^26^. Therefore, it is possible that relatively small climate-induced changes in distribution could have a disproportionate impact on collision vulnerability for whale sharks, and potentially other marine megafauna^30^.

To explore potential climate responses and co-occurrence with shipping, we used a whale shark satellite-tracking dataset spanning 15 years, including tagging sites in all major oceans they inhabit (348 individuals collectively tracked for >15,000 days). Using these data, together with oceanographic variables and global climate models from the Coupled Model Intercomparison Phase 6 (CMIP6), distribution models were developed to (1) generate a first-order approximation of global habitat suitability and (2) project the distribution of whale sharks in two future decades under three mitigation scenarios. These were then used to (3) assess habitat changes and horizontal co-occurrence with shipping traffic.

## Results

Whale shark habitat suitability maps—defined as areas where a given environment has the capacity to support whale sharks, thus determining the likelihood of their presence—were generated using a series of correlative distribution models based on a suite of oceanographic variables and tracked animal movements. Data preparation, model algorithm and oceanographic variable selection followed careful procedures with sensitivity checks (Supplementary Information 3.3, 3.4 and 4.4). Final models performed well in quantitative and qualitative validation tests and were used to explore current and projected future habitat areas for whale sharks (Supplementary Information 4.2 and 4.3). Future habitats were based on CMIP6 data for the years 2050 and 2100 under the shared socioeconomic pathways (SSPs) ssp126, ssp370 and ssp585 (Supplementary Information 3.5).

### Ocean-scale habitat shifts

**Fig. 1:**
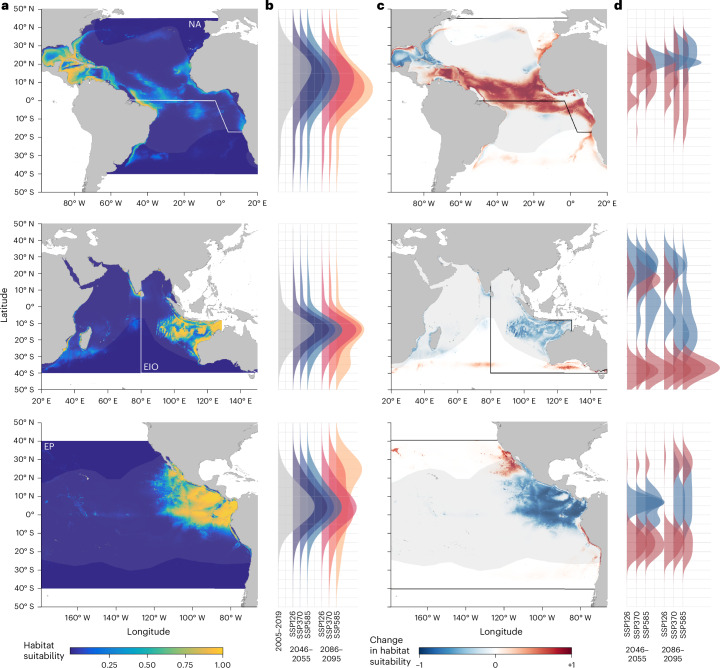
Habitat suitability for whale sharks under current and projected environmental conditions. **a**,**b**, Regions of high (yellow) and low (blue) habitat suitability are indicated for the north Atlantic (NA), east Indian Ocean (EIO) and east Pacific (EP) based on current climatologies (2005–2019) (**a**) and their sum weighted latitudinal density distributions coloured by decade and scenario (**b**). **c**,**d**, Regions of increase (red), decrease (blue) and no change (white) are indicated for NA, EIO and EP based on 2086–2095 ssp585 climatologies (**c**) and their latitudinal density distributions for cells containing positive (>0.5, red) or negative (<−0.5, blue) change values separated by decade and scenario (**d**). Each map shows outputs from GAMs built from tracking data from the respective region projected at the ocean basin scale, and the current IUCN distribution limits are displayed in each map as greyed-out boundaries. Mapped results for other regions are given in Supplementary Figs. 1–7.

**Extended Data Fig. 1:**
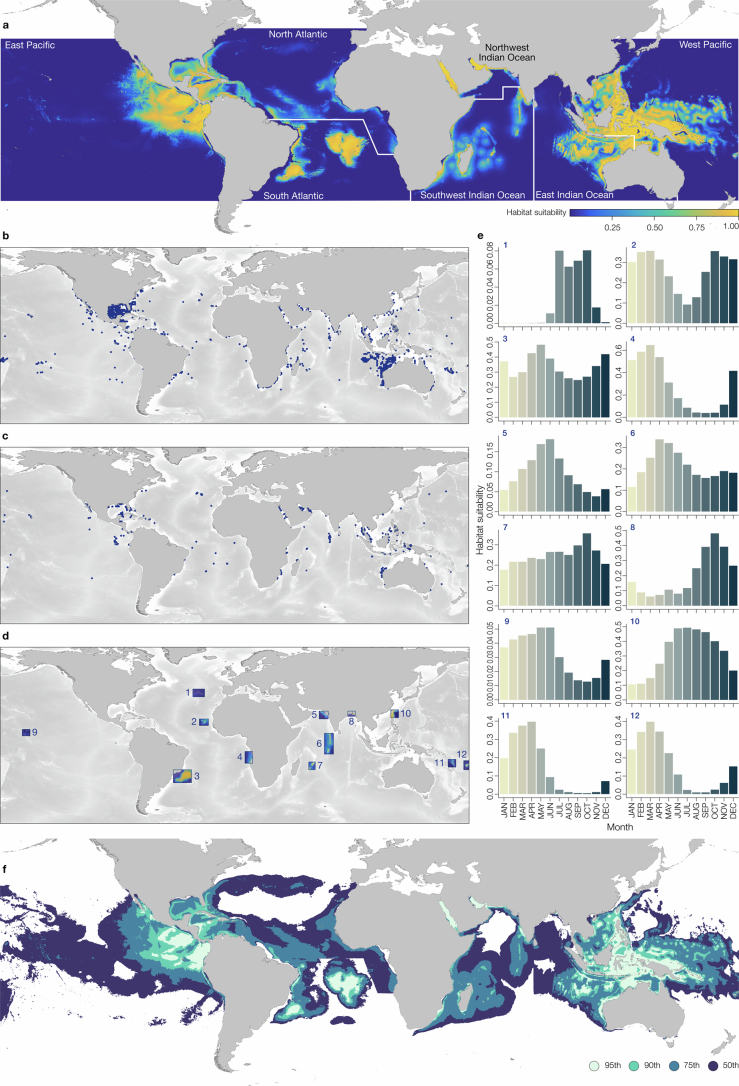
Model outputs and validation. **a**, Regions of high (yellow) and low (blue) habitat suitability are indicated globally where regions have been joined together at boundaries (white border) based on current climatologies (2005–2019). **b**, Encounter locations sourced from the Ocean Biodiversity Information System (OBIS, n = 9,379) and **c**, sharkbook.ai for whale sharks (n = 13,267). **d**, Locations of four qualitative validation regions per major ocean (Atlantic: 1–4, Indian Ocean: 5–8, Pacific: 9–12) with corresponding **e**, mean monthly habitat suitability trends. **f**, The most important core areas for whale sharks currently (2005–2019) indicated by regions within quantile bands (50^th^, dark blue; 75^th^ light blue; 90^th^ green; 95^th^, light green).

Current regions of whale shark habitat suitability were predicted circumglobally within tropical, subtropical and temperate waters (2005–2019; Extended Data Fig. 1a and Supplementary Information 4.5). Using our models to project these habitats into the future, based on changing oceanographic variables, we found increasing habitat suitability at the range edges of current distributions in all decade and scenario combinations (Fig. 1). However, habitat shift patterns varied across regions (Supplementary Figs. 1–7). By the end of the century, under ssp585—also known as the ‘high-emissions’ scenario—the east Pacific shows a habitat reduction in equatorial waters, with potential losses in some currently suitable areas coinciding with expansion into new regions such as the Southern California Bight (Fig. 1c and Supplementary Fig. 7). These changes are related to the unique present-day baseline oceanography of the region and the severity or direction of projected shifts in oceanographic conditions. For example, chlorophyll *a* in the tropical Pacific is linked to the depth of deep scattering layers^35^ within which whale sharks probably forage^36,37^. Warming and decreasing chlorophyll *a* in east Pacific^38^ mid-latitudes point to declining biomass, shallower depths and lower migration proportion of mesopelagic prey. Under these more oligotrophic conditions in future, large areas around the equatorial upwelling may become unsuitable for whale sharks by the end of the century.

**Extended Data Fig. 2:**
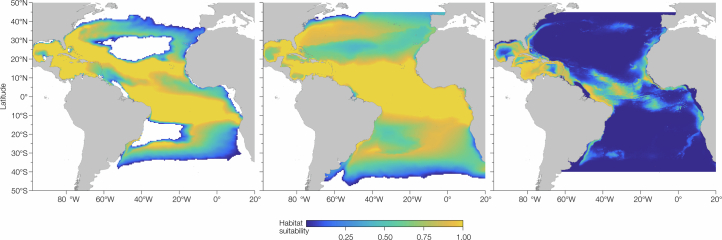
North Atlantic habitat shift comparison with AquaMaps. Regions of high (yellow) and low (blue) habitat suitability in the north Atlantic (NA) generated using the AquaMaps environmental envelope algorithm based on informed parameters from our tracking dataset (n = 1,021, left panel) and independent occurrences from Global Biodiversity Information Framework (GBIF) and Ocean Biodiversity Information System (OBIS) datasets from Food and Agriculture Organization (FAO) major fishing area 31 (n = 312, centre panel) projected into 2050 (decadal average of 2046–2055) under Representative Concentration Pathway (RCP) 5.8. Right panel shows our modelled projections for 2050 (decadal average of 2046–2055) under Shared Socioeconomic Pathway (SSP) 585. All models predict a band of habitats for whale sharks across the central Atlantic basin around equatorial latitudes.

In addition to poleward extensions, north Atlantic habitats show a pronounced shift away from presently important Gulf of Mexico regions into equatorial waters (Fig. 1a and Supplementary Fig. 1). Exploring this further, an AquaMaps environmental envelope model, based on parameters from our tracking dataset, and two independent datasets confirmed increasing habitat suitability in equatorial waters across all tests (Extended Data Fig. 2). This suggests that, unlike other less mobile tropical marine species^13,39,40^, the whale sharks tagged in this study do not occupy their entire fundamental thermal niche and can perhaps tolerate warmer oceans than they currently occupy. The maximum surface temperature experienced by sharks tracked in this region was 31 °C compared with 34 °C in the northwest Indian Ocean. In contrast, it appears that some future surface salinity conditions will exist outside of current preferences. Salinity is projected to increase within the north Atlantic subtropical gyre and surrounding areas under climate change, reflecting an expansion of surface waters characteristic of the gyre, as was seen in the Middle Eocene Climatic Optimum^41^. Low surface productivity driven by climatic and oceanographic processes indicates that the surface waters of subtropical gyres are currently unfavourable habitat for whale sharks, given the sparse foraging opportunities; indeed, movements of other Atlantic migratory sharks across this area appear infrequent^42^ although whale sharks may occur there at deeper depths^43^. Taken together, the expansion of currently unfavourable surface waters in the north central Atlantic, and the fact that present-day upper temperature limits may not have been reached by the sharks tracked in this region, might explain why this species is not expected to conform to commonly held climate change-response hypotheses in all areas across its global geographic range. The influence of localized conditions is an important caveat with all global modelling approaches: our results exemplify a general global trend but with various factors affecting regional patterns (Supplementary Information 4.6).

Habitat change patterns observed in mid-century ssp126 (also known as the ‘sustainable development’ scenario) increased in intensity across ssp370 and ssp585 through to the end of the century (Fig. 1b,d, Extended Data Fig. 3 and Supplementary Figs. 1–15), suggesting that under sustainable development habitat shifts will be less extreme for whale sharks.

**Fig. 2:**
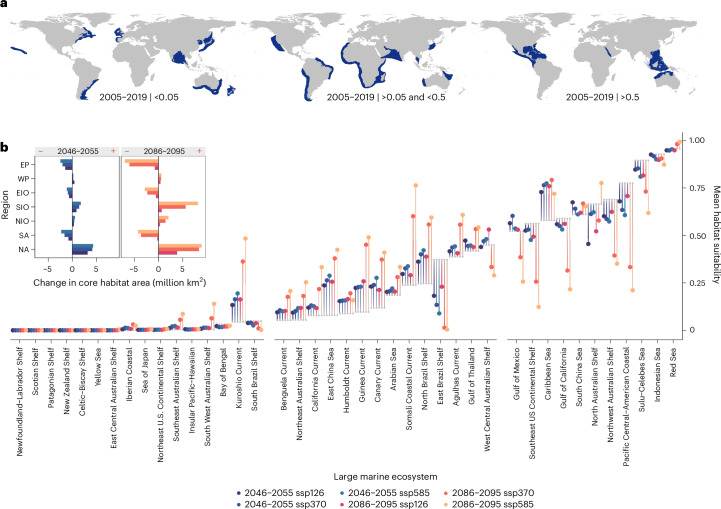
Change in habitat area and quality within boundaries. **a**, Shifts in mean habitat suitability within LMEs ordered from low (left) to high (right) current habitat suitability, with small grey points reflecting the present-day average (2005–2019) and predicted future averages coloured by decade and scenario. World panels show LMEs with ‘low’ (left, <0.05), ‘medium’ (centre, >0.05, <0.5) and ‘high’ (>0.5) current habitat suitability (see Supplementary Fig. 18a for LME climate zones). **b**, Change in total area of habitat suitability (million square kilometres) within the north Atlantic, south Atlantic (SA), northwest Indian Ocean (NIO), southwest Indian Ocean (SIO), east Indian Ocean, west Pacific (WP) and east Pacific between present and future predictions, coloured by decade and scenario, with the periods 2046–2055 and 2086–2095 shown in the left and right panels, respectively, and symbols denoting negative (blue minus) and positive (red plus) change.

**Extended Data Fig. 4:**
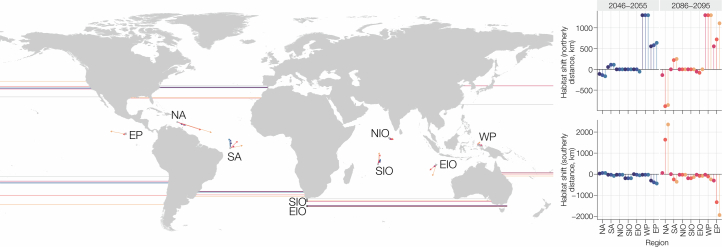
Latitudinal shifts in habitat suitability. Arrows (left panel) indicate shifts in the geographic mean of the relative 90^th^ habitat suitability quantile (core habitat), with the grey mapped points indicating the current geographic mean, and the arrowheads the future geographic mean coloured by decade and scenario. Lines indicate the maximum and minimum latitudes where core habitat was projected, with the grey line indicating the current geographic limits and the future geographic limits coloured by decade and scenario. The lollipop plots (right panel) indicate the corresponding northerly (right upper panel) and southerly (right bottom panel) core habitat limit shifts (distance, km) within each region. Thresholds for core habitat are the relative percentile, calculated from the habitat suitability within each projection subset.

By 2050 the most important habitat area—characterized by the 90th percentile current habitat suitability per region, hereafter core habitat—is expected to decrease in the east Pacific, east Indian Ocean and south Atlantic, and to increase in the western Pacific, southwest Indian Ocean, northwest Indian Ocean and north Atlantic under all scenarios (Fig. 2b and Supplementary Table 1). The same was found for the 2100 projections, but with a greater degree of variation between scenarios, where increases and decreases of >5 million km^2^ (an area larger than the European Union) are expected in the north Atlantic (greater area of core habitat than baseline) and east Pacific (lower area), respectively (Fig. 2b). Our models show that these core habitats may shift latitudinally in future, with changes in distribution limits—expressed in kilometres as the difference between the current and projected latitudinal core habitat limits—differing among regions (median northerly shift, 555 km; mean, 586 ± 496 s.d.; Kruskal–Wallis rank-sum, *χ*² = 20.68, *P* = 0.00037; Extended Data Fig. 4 and Supplementary Table 2). The most pronounced northerly shift was found in the west Pacific, with an overall core habitat northward displacement of >1,300 km expected by 2050, even under scenario ssp126 (Supplementary Figs. 16 and 17 and Supplementary Information 4.7). This is driven by new core habitat areas located around coastal Japan. In the north Atlantic, core habitat limits shifted south when thresholds were calculated within each map (for example, >800 km for 2100 ssp585; Extended Data Fig. 4), and north when thresholds from the baseline years were used (for example >200 km; Supplementary Fig. 17b). This suggests that current core-quality habitats are expanding poleward, but that the most important relative core areas are shifting south.

Overall, northerly core habitat cold edges shifted at a rate of 12 km yr^−1^ (mid-century, 15 km yr^−1^; end of century, 9 km yr^−1^), in keeping with projected responses of chondrichthyan (cartilaginous) fishes^15^, and 2.5 times faster than southerly cold edges (overall, 5 km yr^−1^; mid-century, 6 km yr^−1^; end of century, 3 km yr^−1^), probably driven by the greater rate of ocean warming expected in the Northern Hemisphere^44^. Poleward climate responses are already being empirically validated for whale sharks from new records of individuals^45,46^ and other ectothermic ocean migrants^47^, with more frequent sightings at cold distribution edges, possibly linked to acute warming events^48–51^.

### Ocean-scale temporal trends

**Fig. 3:**
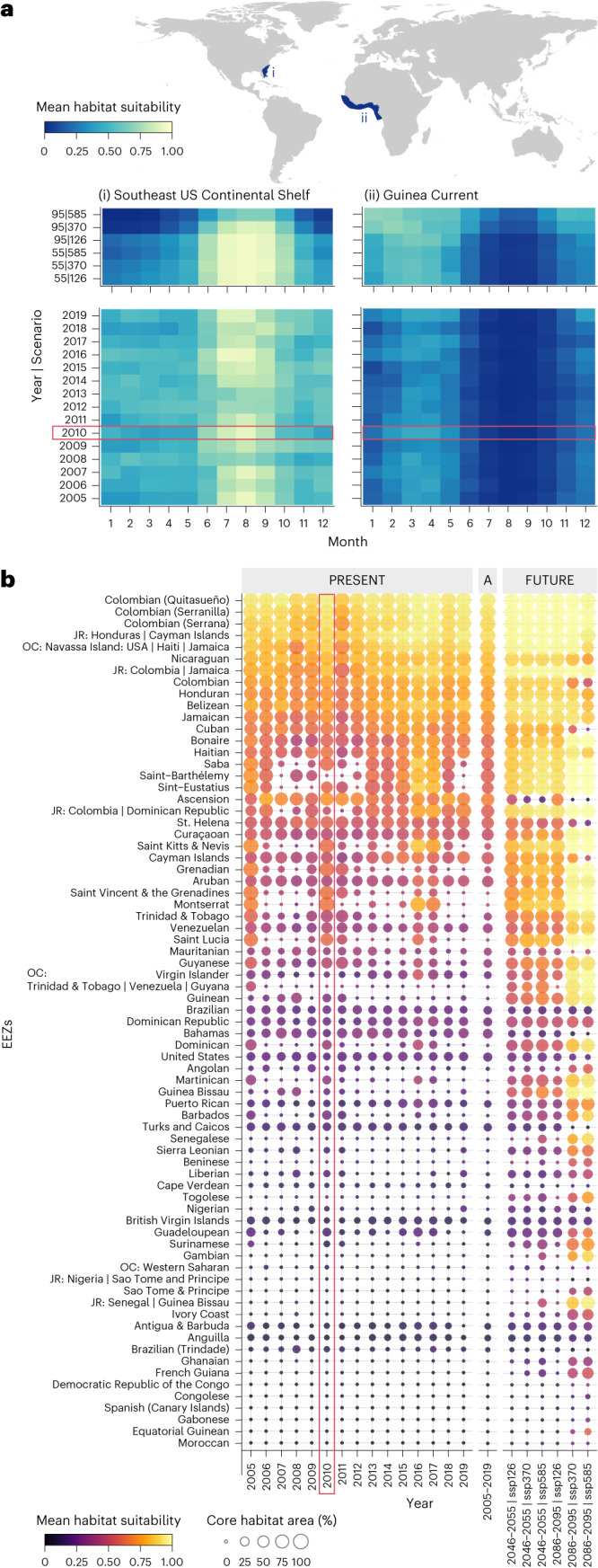
Temporal trends in habitat suitability. **a**, Monthly habitat suitability in which high (yellow) and low (blue) means are summarized within LMEs in the north Atlantic. Upper and lower panels show predicted future for each decade and scenario and present-day annual (2005:2019), respectively, within the Southeast US Continental Shelf LME (left) and Guinea Current LME (right). Axis labels 55 and 95 refer to decadal subsets 2046–2055 and 2086–2095, respectively. **b**, Interannual habitat suitability metrics where high (yellow) and low (black) means and high (large) and low (small) percentage coverage of core habitat area are summarized within EEZs, which are predominately located in the Atlantic Ocean. Left, middle and right, present-day annual (2005–2019), present-day average (2005–2019) and projected future (for each decade and scenario), respectively. Red boxes denote years referenced in the text when past climatic events of note occurred.

We explored how these shifting habitat dynamics might influence nations currently supporting suitable whale shark environments. Both mean habitat suitability and core habitat coverage—that is, the percentage of exclusive economic zone (EEZ; Supplementary Fig. 18b) waters classed as core habitat—shifted latitudinally across years in the Atlantic and, to a lesser extent, in the Pacific and Indian Oceans (Supplementary Figs. 19–21). National waters predominantly located in the Atlantic with currently high mean habitat suitability show increases in the future, exceptions being the Nicaraguan, Colombian and Cuban EEZs where declines in ssp370 and ssp585 by 2100 are expected, reflecting habitat losses in the Gulf of Mexico (Fig. 3b). Many currently less suitable EEZs were also projected to increase in mean habitat suitability in the future, among the most pronounced being the Guinean, Gambian and Senegalese EEZs on the west coast of Africa (Fig. 3b). In contrast, reductions are apparent in several Pacific EEZs currently supporting suitable habitats. To explore intra-annual habitat trends, we calculated the same metrics within large marine ecosystems (LMEs; Supplementary Fig. 18a), finding that seasonal trends in suitability may expand and strengthen in future—for instance in the Guinea Current LME—with increased mean habitat suitability from November to March compared with a more restricted season in the current period (Fig. 3a). The opposite trend is expected in the Southeast US Continental Shelf LME, where the season contracts and weakens by 2100 following a loss of suitable habitats under ssp585 (Fig. 3a).

In some cases, intra- and interannual future projections were similar to trends potentially attributed to past climatic events. For example, the extent of whale shark habitat suitability and its seasonal pattern identified in 2010 had similarities to our projected future in the Atlantic, such as the increasing core habitat coverage in lower-latitude EEZs and relatively strong boreal and austral summer seasons in the Southeast US Continental Shelf and Guinea Current LME, respectively (Fig. 3a,b and Supplementary Fig. 22). This suggests that events such as the documented 2010 Northern Hemisphere heatwave^52^ may predict some future conditions in the Atlantic. Past habitat suitability patterns with similarities to future predictions were also evident in other regions (Supplementary Figs. 23–25 and Supplementary Information 4.8).

### Global redistribution

**Fig. 4:**
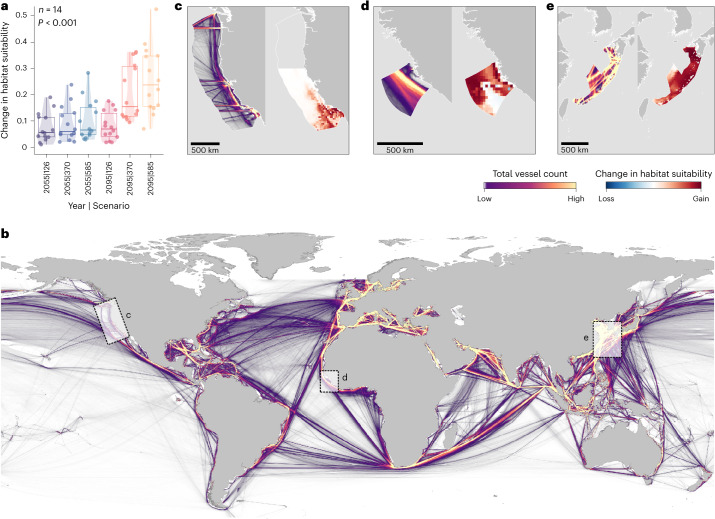
Future redistributions in the context of global shipping. **a**, Projected change in habitat suitability from baseline (absolute, 2005–2019) for 14 LMEs defined as medium importance, in which the result from a Kruskal–Wallis rank-sum test is shown at top left (*χ*² = 32.00, *P* = 5.93 × 10^−6^). Circles denote individual LME values, the thick line denotes the median and boxes bound the interquartile range (25th to 75th percentile), with whiskers extending to the maximum and minimum values. Upper and lower boundaries of violin plots extend to the maximum and minimum values, respectively, and width represents the density of observations. **b**, Global distribution of areas of high (yellow) and low (purple) shipping traffic density defined as the total count of vessels from a 2019 monthly average. **c**–**e**, These areas are shown in close-up in **c**–**e**, respectively. **c**–**e**, Areas of high (yellow) and low (purple) shipping traffic density from a 2019 monthly average (left) and areas of habitat suitability gain (red) and loss (blue) predicted from GAMs (right) shown in the national waters in the United States of America, marine region identification (ID), US part of the north Pacific Ocean (**c**); Sierra Leone, marine region ID, Sierra Leonian part of the north Atlantic Ocean (**d**); Japan, marine region ID, Japanese part of the eastern China Sea (**e**).

Across the global ocean, model projections indicate a more general redistribution of whale sharks from current known centres into current range-edge, or fringing, habitats. Current LMEs with low habitat importance (defined as mean habitat suitability <0.05 based on visual segregation) will remain largely unchanged in the future according to our models, whereas medium-importance areas (mean habitat suitability 0.05–0.5) will become more suitable and high-importance areas (mean habitat suitability >0.5) will become less suitable. This change in habitat differed across decade and scenario combinations (Kruskal–Wallis rank-sum, *χ*² = 42.30, *P* = 5.13 × 10^−8^ for *n* = 28 LMEs with current habitat suitability >0.01; Fig. 2a). For medium-importance regions, the greatest absolute difference in projected habitat suitability means was between the 2050 ssp126 and 2100 ssp585 groups (*n* = 14 LMEs, Kruskal–Wallis rank-sum, *χ*² = 32.00, *P* = 5.93 × 10^−6^; Dunn’s multiple comparisons, *Z* = −4.20, *P* = 0.0004; Fig. 4a). However, in high-importance regions the difference for each decade and scenario combination was less substantial (*n* = 11 LMEs, Kruskal–Wallis rank-sum test, *χ*² = 15.08, *P* = 0.0101), suggesting that the greatest variation in expected absolute change across possible future conditions will occur in currently medium-importance habitats (Fig. 2a), with these areas most impacted by whether we follow a high-emissions or sustainable development scenario.

**Extended Data Fig. 5:**
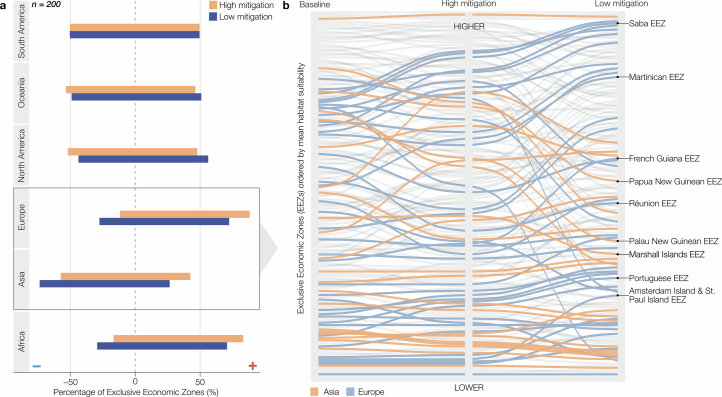
Changing habitats in national waters. **a**, Percentage of Exclusive economic zones (EEZs, n = 200) summarised by continent where positive or negative changes in habitat suitability are projected for the low (blue, ssp585) and high (orange, ssp126) climate mitigation scenarios. **b**, EEZs ordered by the mean habitat suitability showing reshuffling from the baseline (2005–2019) under high mitigation/ sustainable development (ssp126, left to centre panel) and low mitigation/ high emissions (ssp585, centre to right panel) scenarios in 2086–2095. Individual EEZ trajectories are shown with Asia (orange) and Europe (blue) highlighted, demonstrating reshuffling under climate change with examples on the right (for example, where the Portuguese waters increase in relative habitat suitability scores, Papua New Guinean waters decrease).

**Extended Data Fig. 6:**
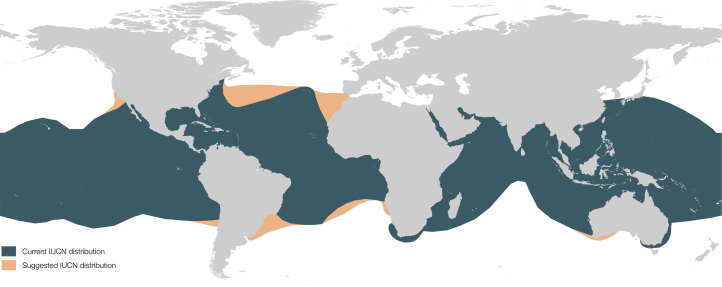
Global known whale shark distribution. The current global extant distribution of whale sharks as defined by the International Union for Conservation of Nature (IUCN) (grey) with expanded range (orange) mapped to identify current predicted and future projected regions where the species is likely to occur based on our models.

Under the high-emissions scenario, globally, 57.5% of EEZs will have suitable habitat losses >50% and 76.5% will have core habitat coverage reductions >50% by 2100 (*n* = 200). These losses were greatest in Asia (where loss is projected for 88.0% of countries with Asian sovereignty, *n* = 25) and least in Europe (42.1%, *n* = 38; Extended Data Fig. 5). Under ssp126, 65.5% of EEZs will gain core habitat coverage of >50% (*n* = 200), with the greatest mean habitat suitability gains apparent in Europe (73.7%, *n* = 38) and least in Asia (28%, *n* = 25; Extended Data Fig. 5). This reshuffling suggests amendment of the currently recognized IUCN range for this species, to account for acute climate events and conservation planning in the future (Extended Data Fig. 6).

### Implications of redistributions

To test whether whale shark vulnerability to ship-strike may change in the future, we applied a previously validated whale shark–ship collision risk index^26^ to the distribution maps and recalculated this as a ship co-occurrence index (SCI; Methods) based on current predicted and future projected habitat suitability and shipping traffic density (Fig. 4b). SCI was calculated within all EEZ marine regions within the range of whale sharks (*n* = 367) for the 2005–2019 baseline and compared with future projections. Here, increases in SCI are driven by (1) habitat suitability increases in new marine regions that overlap with high shipping activity and (2) increases in currently suitable or new regions with lower activity. For example, increased SCI in the US part of the north Pacific Ocean by a factor of 95 can be explained by an increase in newly suitable habitat overlapping busy shipping routes (Fig. 4c). This is also the case in the Japanese part of the eastern China Sea and Sierra Leonian part of the north Atlantic Ocean, where SCI is projected to increase by 272 and 689%, respectively (Fig. 4d,e). In contrast, a SCI increase of 236% in the Somali part of the Indian Ocean is driven by habitat suitability gains expanding into more offshore waters where shipping remains low. Our models suggest that, while these SCI increases occur in some areas, decreases are apparent in others. Similarly, decreases in SCI are driven by both habitat suitability reductions where current habitats overlap high shipping activity, and overall habitat reductions. For example, SCI reductions of 76% in the Mexican part of the Gulf of Mexico result from habitats shifting into more coastal waters away from the busiest shipping routes in the centre of the Gulf. However, reductions of almost 100% in the Clipperton part of the north Pacific Ocean, where shipping is low across the region, are driven by general habitat losses.

**Fig. 5:**
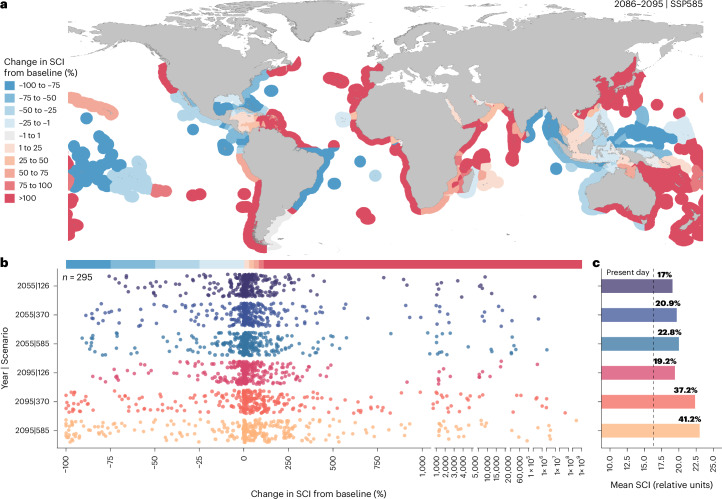
Global habitat reshuffling leads to increased ship co-occurrence. **a**, EEZ marine regions coloured by degree of change in SCI from the 2005–2019 baseline years. Red represents an increase in SCI and blue a decrease for 2100 ssp585. **b**, Percentage change in SCI from the 2005–2019 baseline years within each EEZ marine region, sorted and coloured by decade and scenario combination. **c**, Mean SCI calculated across EEZ marine regions, coloured by decade and scenario combination where the black dotted line represents present-day baseline SCI (2005–2019) and the percentage change from baseline is shown above each bar.

**Extended Data Fig. 7:**
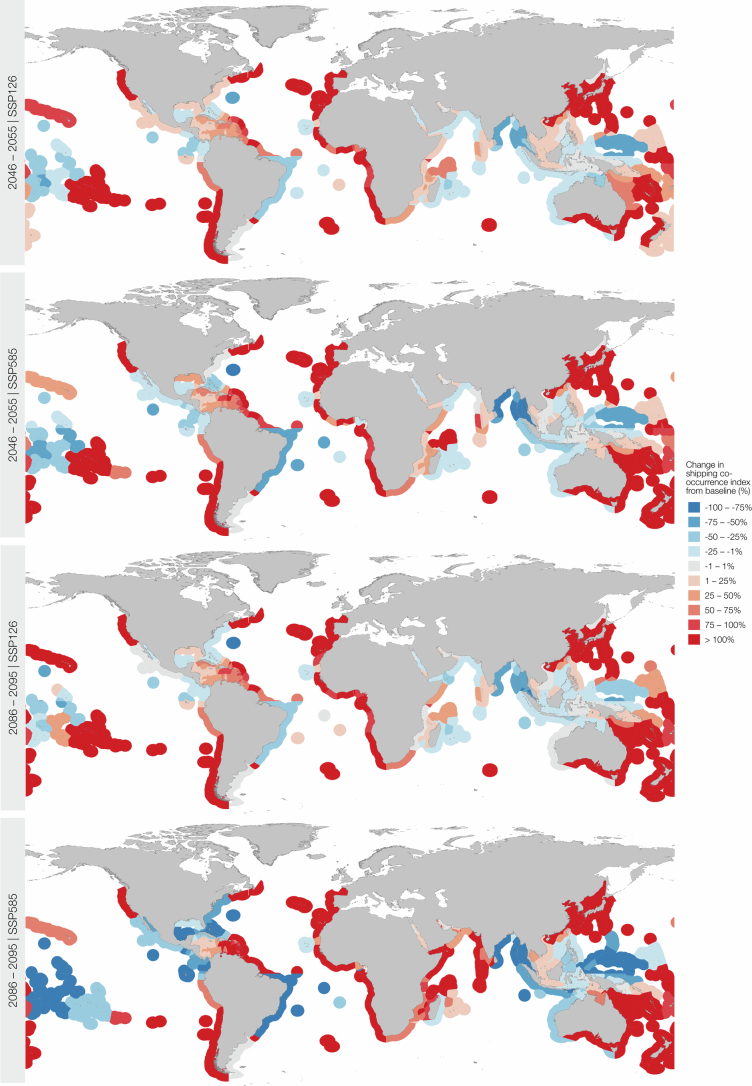
Change in ship co-occurrence index within national waters. Exclusive economic zone (EEZ) marine regions coloured by degree of change in shipping co-occurrence index (SCI) from the 2005–2019 baseline years. Red reflects an increase in SCI and blue a decrease. Scenarios ssp126 and ssp585 are shown for the 2046–2055 (rows 1 and 2) and 2086–2095 (rows 3 and 4) decadal averages.

**Extended Data Fig. 8:**
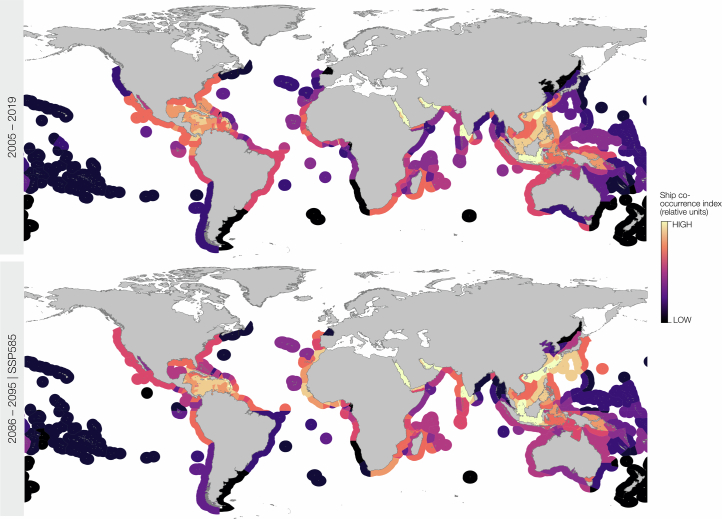
Ship co-occurrence index within national waters. Exclusive economic zone (EEZ) marine regions coloured by shipping co-occurrence index (SCI, relative units where yellow is high and black is low) in the 2005–2019 baseline years (top panel) and 2086–2095 decadal average for ssp585 (bottom panel).

Overall, SCI increased in all future decade and scenario combinations (Fig. 5 and Extended Data Figs. 7 and 8), even when the number of ships was held at current levels compared with the predicted increase in capacity of up to 1,200% by 2050 (ref. ^29^). When regions with mean SCI <0.1 in both the baseline and projected years were removed (*n* = 295 remaining EEZ marine regions), SCI was >15,000 times greater by the end of the century in the high-emissions scenario (ssp585) compared with present-day habitats based on mean change within EEZs (Fig. 5b). Under sustainable development (ssp126) this fell to ~20 times greater, on average. When SCI was averaged across EEZs in the present day and compared with future scenarios, an overall increase of 41.2% by 2100 under high emissions was halved (19.2%) under sustainable development (Fig. 5c). Furthermore, the change from baseline levels was asymmetrical, with substantial increases in SCI projected for most regions (>66%, *n* = 295; Fig. 5a). This is a concerning threat trajectory for the species, considering that there are currently no measures in place to protect whale sharks from shipping^53^. Collision threat for whale sharks also depends on their diving behaviour^26,28^, and moving to address the vertical dimension is an essential next step for assessing the effects of compound climate change events on marine megafauna^54^ (Supplementary Information 4.9).

## Discussion

Using dynamic, individual-animal tracking data, oceanographic variables and state-of-the-art climate models, our study projects habitat changes of the world’s largest fish in two future decades up to 2100 and three climate change scenarios. Projections across worldwide regions showed poleward shifts of over 1,000 km and areas of increasing habitat suitability at latitudinal fringes of current distributions, which were most extreme at the end of the century under the high-emissions scenario. Resultant global-scale redistribution from current centres into fringing, range-edge habitats is possible for this species, with varied ocean basin-scale patterns in direction and location of future habitats relative to present-day distributions. Such a global reshuffling could potentially lead to core habitat losses in national waters currently supporting the species and increased levels of ship co-occurrence as oceans continue warming and other variables shift.

Whale shark ecology and life history mean that habitat shifts may have complex consequences. These are large-bodied, highly mobile ectotherms, closely associated with temperature at multiple scales^36,55–57^. We tracked individuals in surface waters, where they are known to spend large amounts of time^26^, with relatively high temperatures between 18 and 34 °C. Warming of both surface and subsurface layers^58^ will expand the lower temperature limits of the whale sharks’ range polewards, and our models predict that currently suitable habitat areas will follow a similar pattern. This could mean that key aggregation sites^53^—crucial for juveniles and subadults to forage on high prey densities—become difficult to access or remote in the context of individuals’ annual movements. Alternatively, unexpected ocean conditions in new, unfamiliar environments may lead to mortality, as suggested for whale sharks off South Africa^59^. When coupled with the potential reduction in habitat quality expected in some currently suitable regions, these shifting dynamics could have population-level consequences. For example, although it is not yet known where mature adults breed or where females birth their young, shifting habitats could alter these locations, subjecting neonatal whale sharks to increased predation levels or insufficient foraging opportunities.

The implications of the diverse distribution changes we present are highly relevant to conservation. Shifts among national waters could alter protection levels for key demographics and may also impact income for countries with whale shark tourism operations^60^. The potential for increased ship co-occurrence in future highlights the importance of factoring climate change into discussions around endangered species management. The methods developed here to estimate these trends can be adopted for other species to help inform national and international initiatives to conserve biodiversity. This could be by identifying priority areas where effects of compound stressors (for example, ocean heat waves and deoxygenation^54^) are minimized, assessing the resilience of current Marine Protected Area coverage to climate change^61,62^ or designing protection networks that encompass the full range of future habitats, including aggregations, hotspots and refugia^63,64^.

## Methods

### Study regions and tag deployment

The study used a dataset of tracked whale sharks (*n* = 348; Supplementary Fig. 26) tagged in seven large-scale ocean regions; north Atlantic (*n* = 39 individual tracks), south Atlantic (*n* = 14 tracks), northwest Indian Ocean (*n* = 44 tracks), southwest Indian Ocean (*n* = 26 tracks), east Indian Ocean (*n* = 74 tracks), west Pacific (*n* = 62 tracks) and east Pacific (*n* = 89 tracks). This dataset covers 15 years (2005–2019), with 15,508 collective transmission days from field campaigns undertaken by researchers involved in the Global Shark Movement Project (www.globalsharkmovement.org). Details of tag types and deployment methods are provided in ref. ^26^, and ethical approvals and permits are given in Supplementary Information 8.

### Tracked location processing

Tracking data were relayed through the Argos Data Collection and Location System (www.argos-system.org). Argos provides geographic locations estimated via Doppler-shift calculations for ARGOS transmitter tags. For pop-off satellite archival transmitter tags, calculations of light level, temperature and swimming depth were used to estimate geographic locations. The filtering approach described in ref. ^26^ was applied to the tracking data to address spatial error and sampling interval inconsistencies, following which gaps in transmission were interpolated across sections of track having no location estimates, up to a maximum of 3 days. Locations recorded after December 2019 (due to lags in environmental data availability) or that were deemed erroneous due to technology failure or early detachment—determined on a case-by-case basis using an algorithm to detect transmissions indicative of a floating device as opposed to one attached to the animal—were removed from the dataset, resulting in 18,745 regularized daily location estimates across regions (north Atlantic, 2,017; south Atlantic, 331; east Indian Ocean, 4,342; northwest Indian Ocean, 986; southwest Indian Ocean, 946; east Pacific, 3,090; west Pacific, 7,033) (Supplementary Table 3 and Supplementary Fig. 26).

### Species distribution modelling

To identify the environmental drivers important for whale shark movements and space use, we built a series of species distribution models that were used to generate a first-order approximation of present-day (hereafter current) whale shark distribution and project their future distribution under two decade and three climatic scenario combinations (Supplementary Fig. 27). The six-step procedure comprised the components described below.

#### Model data preparation

**Extended Data Fig. 9:**
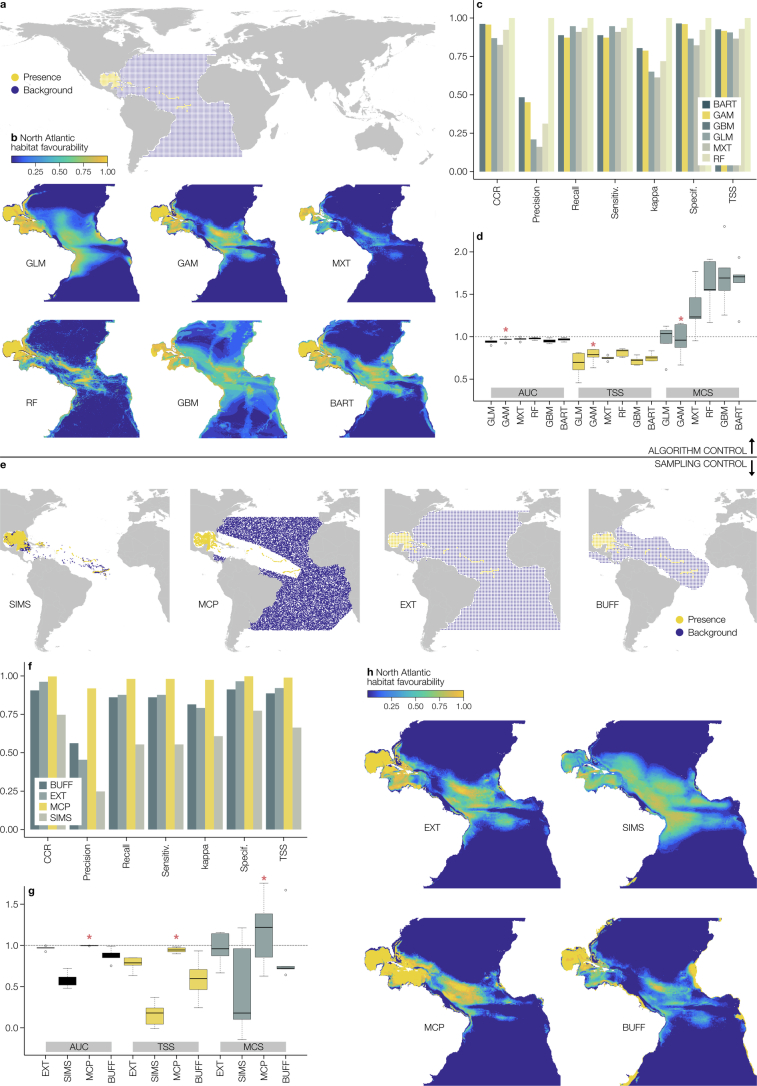
Model controls and performance. **a**, Map of simplified whale shark (presences, yellow) and background (blue) location dataset in the north Atlantic used in the algorithm control procedure detailed in Supplementary Fig. 27 0a. **b**, Regions of high (yellow) and low (blue) habitat favourability in the north Atlantic identified by each algorithm. **c**, Internal validation performance metrics coloured by algorithm with the chosen method (Generalised Additive Models, GAM) in yellow. Note that after high Random Forest (RF) scores were disregarded due to concerns with data overfitting in mapped outputs (**b**), GAMs had high correct classification rate (CCR), precision, kappa and specificity scores second only to Bayesian Additive Regression Trees (BART), whereas Generalised Linear Models (GLM), Generalised Boosted Models (GBM) and Maximum Entropy (MXT) were better at correctly predicting presences (sensitivity and recall). **d**, Cross-fold validation performance metrics based on five spatial folds coloured by test where the black line denotes the median, boxes bound the interquartile range (IQR) (25^th^ to 75^th^ percentile) and whiskers extend to the smaller quantity of data extremes or medians ± 1.5× IQR with outliers shown as open circles and a red asterisk positioned above the chosen method (GAM). GAMs performed well across measures and outperformed BART (which showed better internal validation scores (**c**)), when measuring the true skill statistic (TSS) and Millers calibration statistic (MCS), with the closest MCS value to 1 across all models tested. **e**, Map of simplified whale shark (presences, yellow) and background (blue) location dataset in the north Atlantic used in the background sampling selection procedure detailed in Supplementary Fig. 27 0b. **f**, Internal validation performance metrics coloured by sampling method with the chosen method (MCP) in yellow. MCP had the highest score across tests. **g**, Cross-fold validation performance metrics based on five spatial folds coloured by test where the black line denotes the median, boxes bound the interquartile range (IQR) (25^th^ to 75^th^ percentile) and whiskers extend to the smaller quantity of data extremes or medians ± 1.5× IQR with outliers shown as open circles and a red asterisk is positioned above the chosen method (MCP). The MCP method performed well across measures. **h**, Regions of high (yellow) and low (blue) habitat favourability in the north Atlantic identified by each sampling method. The abbreviations in **a**–**d** refer to the algorithms tested and in **e**–**h** to the background sampling methods tested.

To characterize the biophysical environment at observed tracking locations, a suite of 28 dynamic and physical essential ocean variables (EOVs) were explored as potential drivers of whale shark distribution (Supplementary Table 4 and Supplementary Information 3.1). First, we performed a background sampling selection where locations were generated for each track within its assigned region, sampled at the basin scale cropped to 40° north and south of the Equator (Supplementary Fig. 26). The minimum convex polygon of presence locations, which represents the geographic extent used by individuals tracked in the study—including the spatial error field associated with tracking technologies^42,65^—was masked off within each region and allocated as a buffer extent within which background locations could not be sampled (see Supplementary Information 3.4 and Extended Data Fig. 9 for details on other methods tested)^66,67^. This method of sampling background environments from within the entire accessible range of whale sharks captured essential aspects of the species’ life history (that is, the tendency to aggregate coastally) whilst also allowing for biologically realistic broad-scale extrapolation into current and future oceans. However, it may not be suited to all species and analyses situations, and background sampling selection should be carefully considered on a case-by-case basis in future studies (Supplementary Information 3.4).

For each daily time stamp along a whale shark track, 100 background locations were randomly generated outside of this buffer area up to the latitudinal limits and within a maximum of 40° (ref. ^66^). Then, to avoid potential overfitting and artificially inflated model metrics related to an excessive number of background locations relative to the number of presences in the modelling dataset, background locations were further sampled to obtain a 1:10 presence:background ratio in the models^32,67,68^. For each presence and background location, 28 EOVs (Supplementary Table 4) were extracted from the closest point in space and time using interpolation methods. First, 100 randomized locations were generated within a radius around each original shark location, calculated as the normal distribution plus half the standard deviation of the error radius associated with ARGOS tags (0.12° latitude, 0.12° longitude)^26^ and pop-off satellite archival tags (1.08° latitude, 0.53° longitude)^26^. For each randomized location, a spiral of cells rotating outwards were averaged up to the analysis resolution (0.25°), before all 100 locations were averaged to give a single value per location. Second, to obtain the closest value in time for each location we used a temporal interpolation method, where each consecutive day between downloaded environmental data time stamps was assigned a value based on time differences between available data. Extracted EOVs were windsorized (truncated to percentile) where necessary before being centred and scaled for each region.

#### Model training and oceanographic variable selection

To determine the most important EOVs for predicting the presence of the species, we then developed and compared a set of presence-background, case-control classification models. We used generalized additive models (GAMs; see Supplementary Information 3.3 and Extended Data Fig. 9 for details on other methods tested), applying the bam function with the fast maximum-likelihood method within the mgcv R package^69^, and discretized covariates to improve storage and efficiency^70^. To avoid overfitting, we added a gamma value of 1.4 into all models, which assigns a higher value to penalize lambda (or smoothness) of the parameter relationships^71^, and ensured low *k-*values. To reduce spatiotemporal autocorrelation, we also performed a data-thinning procedure by subsetting locations that were at least 2 days apart and thus removed consecutive daily locations^72^ (Supplementary Table 5). We built models with a reduced number of EOVs to test ecologically relevant driver combinations important for whale shark movements^42,73,74^ (Supplementary Information 3.2).

Eight hypotheses, that included both surface (0 m depth) and subsurface (100 m depth) EOVs, were developed and run on the entire tracking dataset including whale shark positions from all regions (Supplementary Table 6). Sex and size were included as random effects, and month of occurrence as a cyclic cubic regression spline, in all models. The relative performance for each global hypothesis was assessed using the weights of the Akaike information criterion (wAIC). EOVs included in the best-performing global model were then reorganized into a further eight hypotheses to investigate independently within each region. For the region-based models, shark identity was also included as a random effect. This framework allowed for testing of hypotheses containing surface only (hereafter surface) or surface and subsurface (hereafter subsurface) EOVs built from those known to be important for the species generally (Supplementary Fig. 28). The most parsimonious version of each region-based model was chosen based on removal of non-significant EOVs (*P* > 0.05) and comparison of wAIC between model sets before selecting the best-performing model for surface and subsurface hypotheses using wAIC. Following inspection, surface models (comprising surface EOVs) were chosen to take forward to ensure that shallow, near-shore regions were accounted for globally.

Then, to control for variable selection, we ran an automated hypothesis framework in which all non-collinear variables were included in each regional model (Supplementary Fig. 29) and removed epipelagic micronekton for which there are currently no accurate future EOV projections and some current uncertainty (Supplementary Fig. 30). Finally, to control for algorithm selection, we repeated the best-performing region-based hypotheses using a second algorithm, Bayesian additive regression trees (BART), to compare the two modelled and mapped outputs and determine areas of regional model agreement to complement the main analyses based on GAM models (Extended Data Fig. 9, Supplementary Figs. 31–33 and Supplementary Table 7). Here, BART models were run using 200 trees and model defaults^75^.

#### Model validation

Final regional-based model generalization performance was evaluated holistically by testing explanatory power, predictive skill and biological realism^76^. Explanatory power was evaluated using the percentage deviance explained for each hypothesis (Supplementary Tables 8 and 9). Predictive skill was tested internally using tenfold cross-validation conducted on each regional dataset with the dismo package^77^ (measuring model accuracy, precision, sensitivity, specificity, area under the curve, kappa and true skill statistic; Supplementary Table 10 and Supplementary Information 4.2), and on two external, independent datasets: observations of whale sharks downloaded from the Ocean Biodiversity Information System (OBIS, https://obis.org/; *n* = 9,379) and a set of verified observations of marked individuals from Sharkbook.ai for whale sharks (https://www.sharkbook.ai/; *n* = 13,437), with model performance measured with continuous Boyce index (Extended Data Fig. 1). Biological realism was tested qualitatively by visual inspection of the prediction maps and evaluation of the ability of the models to predict known general patterns of species distributions throughout the course of the year (Extended Data Fig. 1 and Supplementary Information 4.3). We assessed maps using validation areas that were beyond those used by tracked individuals in the model training dataset. Four regions were selected per ocean (Atlantic, Indian Ocean, Pacific), reviewed for seasonal habitat suitability and then compared with published literature, opportunistic news reports and expert knowledge (Supplementary Table 11). We also used the AquaMaps^78^ environmental envelope algorithm based on occurrence records from the Global Biodiversity Information Facility and OBIS, located in Food and Agriculture Organization major fishing area 31, to explore alongside our projected habitat maps in the north Atlantic and compare outputs. Here, we used these freely available occurrence records and our tracking data from the region to generate parameters for sea surface temperature (SST), bathymetric depth (DEPTH) and salinity (SAL) and fit into the envelope model. We then compared mapped outputs generated from the independent occurrences with those from our tracking data using the AquaMaps algorithm and our main analysis results (Extended Data Fig. 2). For this comparison, AquaMaps projections for the year 2050 were generated based on a decadal average (2046–2055) for representative concentration pathway 8.5 from the Max Planck Institute Earth System Model, using a debiasing approach similar to that applied in our main analysis^78^.

#### Predictions of current distribution

Monthly and overall predictions from the final GAM model were generated to provide a map of the potential distribution of whale sharks within each region. Maps represented the probability (0–1) in each grid cell of those containing a presence (as opposed to a background) track location, reflecting a given environment’s capacity to support the species which, here, was interpreted as a relative measure of habitat suitability. For the overall habitat maps (with no monthly or annual component), EOV layers were averaged across the extent of tracking years available (2005–2019), and for monthly predictions averages for each month were taken from across the extent of tracking years available or from within a specific year accordingly. For independent dataset validation, region-based predictions were joined together by defined boundaries to create a single global map (Extended Data Fig. 1a). For the main analyses we used regional models to predict at the ocean basin scale; for example, the north Atlantic model was used to generate maps for the entire Atlantic cropped to within known species’ latitudinal distribution limits. In this case, predictions beyond region boundaries (Extended Data Fig. 1a) may represent potential overextrapolation and should be interpreted with caution due to distance of predicted habitats from tracked individuals included in the training dataset. As such, region boundaries were included in all visualizations to aid interpretation. Our method of predicting presence probability includes the effect of whale shark prevalence (proportion of presences) in the modelled dataset. To account for this, we standardized the presence background ratio across regions and compared only those mapped habitat suitability outputs based on the same tracking datasets such that the effect of prevalence remained consistent. In addition, we also calculated current habitat favourability by incorporating dataset-specific prevalence into the predicted outputs using the ‘fuzzySim’ package^79^, finding that habitat patterns remained consistent (Supplementary Fig. 34).

#### Predictions of future distribution

**Extended Data Fig. 10:**
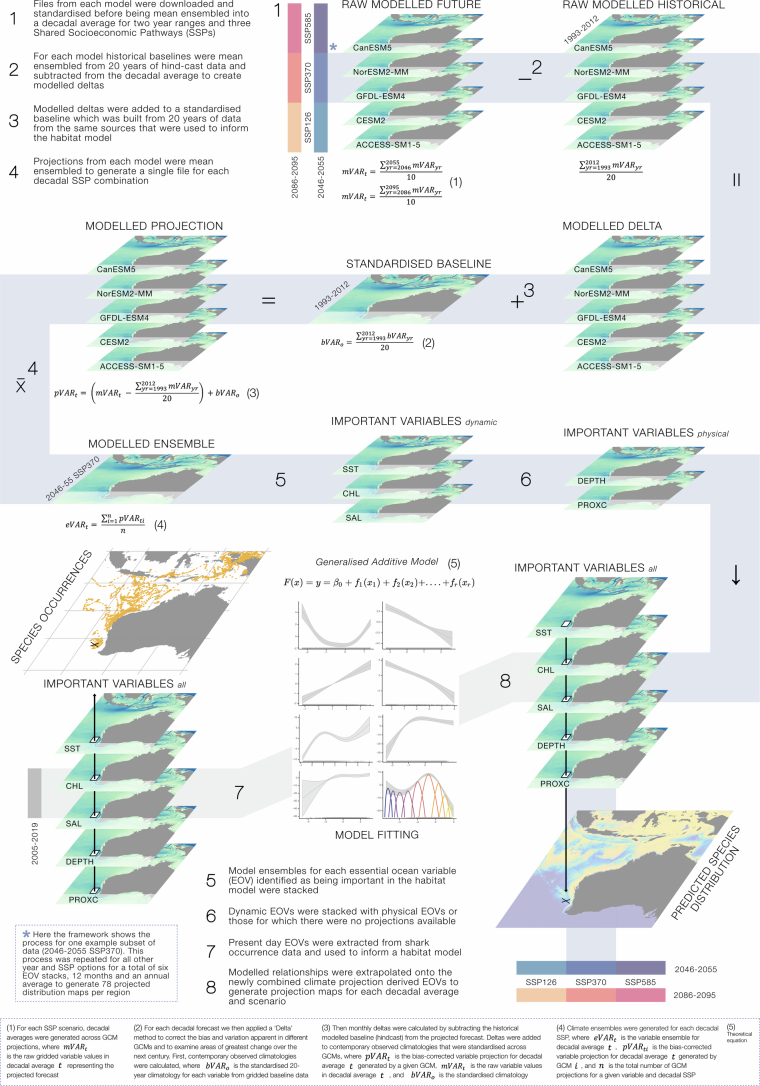
Projection method framework. Steps taken to prepare global climate model (GCM) data for use in the study. Steps 1 to 6 were undertaken first to prepare the data for section 5 in Supplementary Fig. 27. Steps 7 and 8 are a summarised version of section 0a to 2b in Supplementary Fig. 27. Steps 1 to 5 were undertaken for each essential ocean variable (EOV) which were then stacked in steps 5 and 6, all of which was repeated for each decade and scenario combination. See Supplementary Information 3.5 for detailed description and equations used.

Data from the projected EOVs were extracted monthly and overall from two future global climate model decadal means: mid-century (2050; 2046–2055) and end of century (2100; 2086–2095), and under three SSPs: the most optimistic scenario (ssp126, reflecting most closely the 1.5 °C warming target under the Paris Agreement), a medium–high-forcing scenario (ssp370) and the high-forcing scenario (ssp585, retaining a strong reliance on fossil fuels in the future) (Supplementary Information 3.5 and Supplementary Figs. 35–37). From CMIP6, ACCESS-ESM1-5, CanESM5, CESM2-WACCM, CMCC-ESM2, GFDL-ESM4, IPSL-CM6A-LR, MPI-ESM1-2-HR and NorESM2-MM were applied in a delta change framework (see Supplementary Information 3.5 for further details on calculations and Extended Data Fig. 10 for the methods schematic). Climatic projections of future distributions within each region were generated using these forecast maps.

Overall change in habitat suitability was estimated as the difference between the area covered by current core suitable habitat (>90th percentile habitat suitability) and projected habitat (>90th percentile current habitat suitability). Relative change in the location of important core habitats within future projection maps was based on the >90th percentile habitat suitability within each projection subset. The 90th threshold was chosen as representative of the most important core habitat within each region, to ensure balanced datasets for comparisons. The 90th threshold also reflects the aggregative nature of the species that is known to form high relative-abundance ‘hotspots’ on an often seasonal basis^80^. Habitat change calculations were performed spatially (including within geopolitical boundaries; Supplementary Fig. 18) and across time. They were also explored as a binary output whereby the cells from GAM and BART model-predicted change were used to identify regions of model agreement. All area calculations were an estimate based on a uniform 0.25° grid calculated in the raster package^81^. Latitudinal change in northerly and southerly habitat shift was assessed by calculating the movement of whale sharks (expressed in kilometres) as the difference between the current latitudinal range and limits and the projected latitudinal range and limits in future, primarily based on the 90th percentile and rerun on the 50th, 75th and 95th percentiles for additional support. The main results present shifts based on calculation of core habitat within each projection subset to determine where the most important areas have shifted irrespective of overall habitat changes. Shifts based on comparison of future core habitat limits with present-day thresholds are presented in Supplementary Tables 2 and 12 and Supplementary Figs. 16 and 17. These were also explored quarterly. All statistical comparisons were checked for normality using the Shapiro–Wilk test.

#### Current and future shipping co-occurrence estimates

Global shipping data were sourced from Global Fishing Watch (www.globalfishingwatch.org) and comprised a total monthly count of all commercial vessels >300 gross tons transiting the ocean in 2019, which were then aggregated into an annual average at 0.2° grid cell resolution. A SCI was calculated by adapting the collision risk index described in ref. ^26^, where shipping data were fixed to 2019 and whale shark spatial density used in that study was substituted for habitat suitability in the current study, which represents the probability of a whale shark occurring within a cell (0–1). SCI was then averaged spatially by calculating the mean of all cells within each EEZ marine region. Projected future difference in SCI was calculated as a percentage change from the 2005–2019 SCI baseline. This calculation is a measure of whale shark habitat suitability and ship co-occurrence and does not include dynamic movements of either ships or sharks. Shipping traffic density will probably shift in future in response to socioeconomic factors, including population growth, global trade and the worldwide transport of materials^29^. In addition, as a highly mobile species, whale sharks will not occupy all suitable habitats at all times of the year. This means that SCI does not represent absolute collision risk, but rather an estimate of where and to what extent these two groups may overlap in future compared with current oceans.

### Reporting summary

Further information on research design is available in the Nature Portfolio Reporting Summary linked to this article.

## Online content

Any methods, additional references, Nature Portfolio reporting summaries, source data, extended data, supplementary information, acknowledgements, peer review information; details of author contributions and competing interests; and statements of data and code availability are available at 10.1038/s41558-024-02129-5.

## Supplementary information

Supplementary Information Supplementary Tables 1–12, Figs. 1–37, methods, results and discussion, references, acknowledgements and details of ethical compliance and approvals.

Reporting Summary

## Figures and Tables

**Extended Data Fig. 3:**
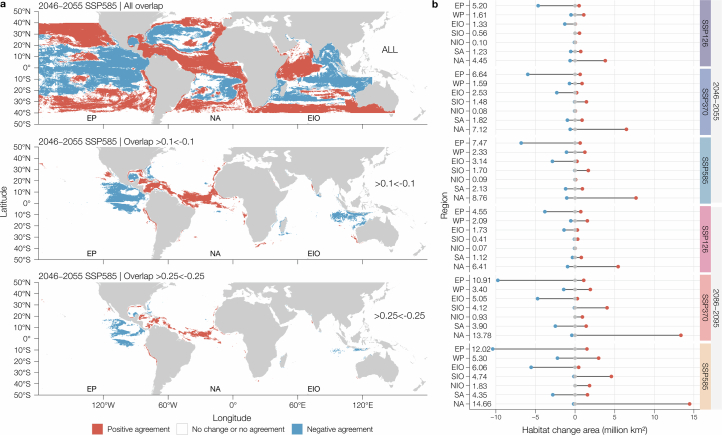
Location and area of predicted changes in habitat suitability. **a**, Regions of positive (red), negative (blue) or no change (or agreement, white) identified by both Generalised Additive Model (GAM) and Bayesian Additive Regression Tree (BART) algorithms in the east Pacific (EP), north Atlantic (NA) and east Indian Ocean (EIO) with top panel showing all regions of either positive or negative model agreement, centre panel showing regions of positive or negative agreement >0.1 or <−0.1, respectively, and lower panel showing regions of positive or negative agreement >0.25 or <−0.25, respectively for 2050 ssp585. **b**, Area (in million km^2^) of predicted change in habitat importance (>0.1 or <−0.1) identified by both GAM and BART models in the NA, south Atlantic (SA), northwest Indian Ocean (NIO), southwest Indian Ocean (SIO), EIO, west Pacific (WP) and EP coloured by decade and scenario were values on the left of each panel denote total area of change and points denote area of positive (red) and negative (blue) change. Mapped results for other regions are given in the Supplementary Figs. 9–15.

## Data Availability

Environmental data are available at https://data.marine.copernicus.eu/products. CMIP6 data are available at https://esgf-ui.ceda.ac.uk/cog/search/cmip6-ceda/. Shipping data are available on request to Global Fishing Watch (https://www.globalfishingwatch.org). OBIS and SharkBook whale shark observation data are available at https://obis.org (open) and https://www.sharkbook.ai/ (on request), respectively. AquaMaps data are available at https://www.aquamaps.org. EEZ boundary data are available at https://www.marineregions.org/downloads.php. LME boundary data are available at https://github.com/datasets/lme-large-marine-ecosystems/. Land boundary data are available at https://www.naturalearthdata.com. IUCN boundary data are available at https://www.iucnredlist.org/ja/species/19488/2365291. Derived whale shark habitat suitability maps for the present day and future are available on GitHub^82^.

## Code availability

Code used in this analysis to calculate SCI is available on GitHub^82^ with examples based on the derived open-access datasets (as listed in the Data Availability statement).
