# Supplementary material for: Climate-driven global redistribution of an ocean giant predicts increased threat from shipping
Source: Nat Clim Chang. 2024 Oct 7;14(12):1282–91. doi: 10.1038/s41558-024-02129-5 (PMC11618081; doi:10.1038/s41558-024-02129-5)
Supplement: Supplementary file 1 — Supplementary Tables 1–12, Figs. 1–37, methods, results and discussion, references, acknowledgements and details of ethical compliance and approvals. [file 41558_2024_2129_MOESM1_ESM.pdf]

nature climate change

Supplementary information https://doi.org/10.1038/s41558-024-02129-5

# Climate-driven global redistribution of an ocean giant predicts increased threat from shipping

In the format provided by the authors and unedited

## Contents

**I. Supplementary Tables ........ 2**

**2. Supplementary Figures ........ 36**

**3. Supplementary Methods ........ 83**

3.1 Oceanographic variable selection ........ 83

3.2 Hypothesis formation ........ 84

3.3 Algorithm selection control ........ 86

3.4 Background sampling control ........ 87

3.5 Predictions of future distributions ........ 88

**4. Supplementary Results and Discussion ........ 91**

4.1 Whale shark tracking dataset ........ 91

4.2 Model performance and oceanographic variable relationships ........ 91

4.3 Model validation ........ 92

4.4 Sensitivity analyses ........ 93

4.5 Current whale shark habitats ........ 94

4.6 Discussion of whale shark habitat drivers ........ 96

*4.6.1 Atlantic ........ 96*

*4.6.2 Indian Ocean ........ 97*

*4.6.3 Pacific ........ 99*

4.7 Future range shifts ........ 101

4.8 Past climatic events ........ 102

4.9 Study limitations ........ 102

**5. Supplementary References ........ 104**

**6. Supplementary Acknowledgements ........ 111**

**8. Details of ethical compliance and approvals ........ 113**

## I. Supplementary Tables

**Table S1 |** Suitable habitat area and percentage coverage calculated within each region for the current distribution (2005 – 2019) and each projected future decade and scenario combination. Here core habitats are defined as the top 90th percentile of the current distribution. All areas are estimated as million $km^2$.

| *Habitats compared to quantiles within 2005-2019 baseline *areas given as million km²* | | **Current** | **Future** | | | | | |
|---|---|---|---|---|---|---|---|---|
| | | **2005-2019** | **2046-2055** | | | **2086-2095** | | |
| | | | **ssp126** | **ssp370** | **ssp585** | **ssp126** | **ssp370** | **ssp585** |
| North Atlantic | Area* / km² | 6.38 | 9.56 | 10.53 | 10.71 | 10.23 | 14.75 | 15.30 |
| | % | 10.44 | 15.64 | 17.22 | 17.52 | 16.73 | 24.12 | 25.02 |
| South Atlantic | Area / km² | 6.34 | 5.57 | 4.76 | 4.06 | 6.10 | 2.75 | 2.12 |
| | % | 10.37 | 9.11 | 7.79 | 6.65 | 9.98 | 4.49 | 3.46 |
| Northwest Indian Ocean | Area / km² | 5.25 | 5.59 | 5.67 | 5.82 | 5.58 | 6.69 | 7.32 |
| | % | 10.16 | 10.81 | 10.95 | 11.25 | 10.79 | 12.94 | 14.15 |
| Southwest Indian Ocean | Area / km² | 5.24 | 6.12 | 6.67 | 7.02 | 5.90 | 10.85 | 13.40 |
| | % | 10.13 | 11.82 | 12.89 | 13.57 | 11.41 | 20.97 | 25.90 |
| East Indian Ocean | Area / km² | 5.29 | 4.63 | 4.39 | 4.17 | 4.77 | 2.98 | 2.44 |
| | % | 10.22 | 8.95 | 8.49 | 8.05 | 9.21 | 5.76 | 4.72 |
| West Pacific | Area / km² | 5.16 | 5.61 | 5.37 | 5.38 | 5.57 | 5.67 | 5.77 |
| | % | 10.39 | 11.28 | 10.80 | 10.82 | 11.20 | 11.40 | 11.61 |
| East Pacific | Area / km² | 9.06 | 7.61 | 7.05 | 6.62 | 8.30 | 3.06 | 2.11 |
| | % | 10.50 | 8.83 | 8.18 | 7.67 | 9.63 | 3.55 | 2.44 |

**Table S2 |** Minimum and maximum latitudinal limits calculated for each percentile (50th, 75th, 90th, 95th) of habitat suitability for the current distribution and each projected future decade and scenario combination at 0.25 × 0.25° resolution within each region. Here core habitats are defined as:

**a)** The top Xth of the relative distribution.

| 50th | | | | | | | | |
|---|---|---|---|---|---|---|---|---|
| *Habitats compared to quantiles within each respective map* | | **Current** | **Future** | | | | | |
| | | **2005-2019** | **2046-2055** | | | **2086-2095** | | |
| | | | **ssp126** | **ssp370** | **ssp585** | **ssp126** | **ssp370** | **ssp585** |
| North Atlantic | Max | 40.625 | 42.875 | 43.125 | 43.375 | 42.875 | 44.875 | 44.875 |
| | Min | -37.375 | -38.375 | -38.625 | -38.625 | -37.625 | -39.375 | -39.875 |
| South Atlantic | Max | 40.375 | 42.875 | 42.875 | 42.875 | 42.875 | 43.375 | 44.625 |
| | Min | -34.125 | -34.125 | -34.375 | -34.625 | -34.125 | -37.625 | -39.625 |
| Northwest Indian Ocean | Max | 30.125 | 30.125 | 30.125 | 30.125 | 30.125 | 30.125 | 30.125 |
| | Min | -39.875 | -39.875 | -39.875 | -39.875 | -39.875 | -39.875 | -39.875 |
| Southwest Indian Ocean | Max | 30.125 | 30.125 | 30.125 | 30.125 | 30.125 | 30.125 | 30.125 |
| | Min | -37.875 | -38.125 | -38.375 | -38.375 | -38.125 | -38.625 | -38.625 |
| East Indian Ocean | Max | 30.125 | 30.125 | 30.125 | 30.125 | 30.125 | 30.125 | 30.125 |
| | Min | -39.875 | -39.875 | -39.875 | -39.875 | -39.875 | -39.875 | -39.875 |
| West Pacific | Max | 38.875 | 38.875 | 36.875 | 38.875 | 38.875 | 37.125 | 38.875 |
| | Min | -22.125 | -22.375 | -22.625 | -22.375 | -22.375 | -30.125 | -32.125 |
| East Pacific | Max | 35.875 | 36.375 | 36.625 | 36.875 | 36.625 | 39.875 | 39.875 |
| | Min | -34.875 | -35.125 | -36.625 | -38.125 | -36.625 | -39.875 | -39.875 |
| **75th** | | | | | | | | |
| *Habitats compared to quantiles within each respective map* | | **Current** | **Future** | | | | | |
| | | **2005-2019** | **2046-2055** | | | **2086-2095** | | |
| | | | **ssp126** | **ssp370** | **ssp585** | **ssp126** | **ssp370** | **ssp585** |
| North Atlantic | Max | 39.375 | 39.375 | 40.625 | 40.625 | 39.375 | 39.375 | 39.375 |
| | Min | -36.125 | -36.125 | -36.125 | -36.375 | -36.125 | -36.375 | -36.625 |
| South Atlantic | Max | 38.875 | 38.875 | 39.125 | 39.125 | 39.125 | 42.125 | 42.875 |
| | Min | -31.375 | -31.625 | -32.125 | -32.625 | -31.875 | -33.625 | -34.625 |
| Northwest Indian Ocean | Max | 30.125 | 30.125 | 30.125 | 30.125 | 30.125 | 30.125 | 30.125 |
| | Min | -39.875 | -39.875 | -39.875 | -39.875 | -39.875 | -39.875 | -39.875 |
| Southwest Indian Ocean | Max | 29.875 | 29.875 | 29.875 | 29.625 | 29.875 | 29.375 | 29.375 |
| | Min | -36.375 | -37.125 | -37.125 | -37.125 | -37.375 | -36.875 | -36.875 |
| East Indian Ocean | Max | 30.125 | 30.125 | 30.125 | 30.125 | 30.125 | 30.125 | 30.125 |
| | Min | -39.625 | -39.875 | -39.875 | -39.875 | -39.875 | -39.875 | -39.875 |
| West Pacific | Max | 36.875 | 36.875 | 36.875 | 36.875 | 36.875 | 37.125 | 37.125 |
| | Min | -18.375 | -19.125 | -20.625 | -20.625 | -20.375 | -22.125 | -23.375 |
| East Pacific | Max | 35.625 | 35.625 | 35.875 | 35.875 | 35.875 | 39.875 | 39.875 |
| | Min | -30.375 | -32.375 | -33.125 | -34.375 | -32.125 | -39.625 | -39.875 |

**b)** The top X^th^ of the relative distribution.

| 90th | | | | | | | | |
|---|---|---|---|---|---|---|---|---|
| *Habitats compared to quantiles within each respective map* | | Current | Future | | | | | |
| | | 2005-2019 | 2046-2055 | | | 2086-2095 | | |
| | | | ssp126 | ssp370 | ssp585 | ssp126 | ssp370 | ssp585 |
| North Atlantic | Max | 37.125 | 36.125 | 35.875 | 35.625 | 35.875 | 29.125 | 29.375 |
| | Min | -34.125 | -33.875 | -33.625 | -33.625 | -33.625 | -19.375 | -12.875 |
| South Atlantic | Max | 37.125 | 37.625 | 38.125 | 38.125 | 37.125 | 39.125 | 39.375 |
| | Min | -30.125 | -30.375 | -30.375 | -30.875 | -30.125 | -32.375 | -33.375 |
| Northwest Indian Ocean | Max | 30.125 | 30.125 | 30.125 | 30.125 | 30.125 | 30.125 | 30.125 |
| | Min | -38.125 | -38.375 | -38.375 | -38.375 | -38.375 | -38.375 | -38.375 |
| Southwest Indian Ocean | Max | 28.375 | 28.375 | 28.375 | 28.375 | 28.375 | 28.375 | 28.375 |
| | Min | -34.875 | -36.625 | -36.625 | -36.625 | -36.625 | -36.625 | -36.125 |
| East Indian Ocean | Max | 30.125 | 30.125 | 30.125 | 29.625 | 29.625 | 29.375 | 30.125 |
| | Min | -39.125 | -39.125 | -39.375 | -39.625 | -39.375 | -39.875 | -39.875 |
| West Pacific | Max | 25.125 | 36.875 | 36.875 | 36.875 | 36.875 | 36.875 | 36.875 |
| | Min | -18.125 | -18.375 | -18.375 | -18.375 | -18.375 | -19.125 | -20.375 |
| East Pacific | Max | 29.875 | 34.875 | 35.125 | 35.625 | 34.875 | 36.375 | 39.875 |
| | Min | -20.875 | -23.625 | -24.375 | -24.875 | -23.625 | -32.875 | -38.375 |

| 95th | | | | | | | | |
|---|---|---|---|---|---|---|---|---|
| *Habitats compared to quantiles within each respective map* | | Current | Future | | | | | |
| | | 2005-2019 | 2046-2055 | | | 2086-2095 | | |
| | | | ssp126 | ssp370 | ssp585 | ssp126 | ssp370 | ssp585 |
| North Atlantic | Max | 35.375 | 32.875 | 32.125 | 30.625 | 31.375 | 28.625 | 29.125 |
| | Min | -33.125 | -11.125 | -11.375 | -11.125 | -11.125 | -11.125 | -11.125 |
| South Atlantic | Max | 35.625 | 35.875 | 36.875 | 37.125 | 36.375 | 38.125 | 38.375 |
| | Min | -29.375 | -29.625 | -29.875 | -30.125 | -29.625 | -31.125 | -32.375 |
| Northwest Indian Ocean | Max | 30.125 | 30.125 | 30.125 | 30.125 | 30.125 | 30.125 | 30.125 |
| | Min | -38.125 | -38.125 | -38.125 | -38.125 | -38.125 | -38.125 | -38.125 |
| Southwest Indian Ocean | Max | 28.375 | 28.375 | 28.375 | 28.375 | 28.375 | 28.125 | 28.125 |
| | Min | -34.375 | -36.375 | -36.375 | -36.375 | -36.375 | -35.125 | -34.625 |
| East Indian Ocean | Max | 30.125 | 29.625 | 29.625 | 29.125 | 29.375 | 29.125 | 29.625 |
| | Min | -38.875 | -39.125 | -39.125 | -39.375 | -39.125 | -39.875 | -39.875 |
| West Pacific | Max | 24.375 | 25.375 | 36.875 | 36.875 | 36.875 | 36.875 | 36.875 |
| | Min | -17.125 | -17.375 | -18.125 | -18.125 | -18.125 | -18.375 | -19.375 |
| East Pacific | Max | 28.375 | 28.375 | 29.125 | 32.875 | 27.875 | 35.625 | 39.875 |
| | Min | -17.875 | -23.125 | -23.625 | -23.625 | -23.125 | -26.375 | -34.875 |

**c)** The top X$^{th}$ of the current distribution (2005 – 2019).

| 50th | | | | | | | | |
|---|---|---|---|---|---|---|---|---|
| *Habitats compared to quantiles within 2005-2019 baseline* | | **Current** | **Future** | | | | | |
| | | **2005-2019** | **2046-2055** | | | **2086-2095** | | |
| | | | **ssp126** | **ssp370** | **ssp585** | **ssp126** | **ssp370** | **ssp585** |
| North Atlantic | Max | 40.625 | 42.875 | 43.125 | 43.375 | 43.125 | 44.875 | 44.875 |
| | Min | -37.375 | -38.375 | -38.625 | -38.625 | -38.375 | -39.375 | -39.625 |
| South Atlantic | Max | 40.375 | 42.875 | 42.875 | 42.875 | 42.875 | 42.875 | 43.875 |
| | Min | -34.125 | -34.125 | -34.375 | -34.625 | -34.375 | -35.125 | -38.625 |
| Northwest Indian Ocean | Max | 30.125 | 30.125 | 30.125 | 30.125 | 30.125 | 30.125 | 30.125 |
| | Min | -39.875 | -39.875 | -39.875 | -39.875 | -39.875 | -39.875 | -39.875 |
| Southwest Indian Ocean | Max | 30.125 | 30.125 | 30.125 | 30.125 | 30.125 | 30.125 | 30.125 |
| | Min | -37.875 | -38.375 | -38.625 | -38.625 | -38.375 | -39.375 | -39.375 |
| East Indian Ocean | Max | 30.125 | 30.125 | 30.125 | 30.125 | 30.125 | 30.125 | 30.125 |
| | Min | -39.875 | -39.875 | -39.875 | -39.875 | -39.875 | -39.875 | -39.875 |
| West Pacific | Max | 38.875 | 38.875 | 38.875 | 38.875 | 38.875 | 37.125 | 38.875 |
| | Min | -22.125 | -24.625 | -25.125 | -25.875 | -26.875 | -31.125 | -32.625 |
| East Pacific | Max | 35.875 | 36.625 | 36.875 | 39.875 | 37.875 | 39.875 | 39.875 |
| | Min | -34.875 | -35.125 | -37.875 | -38.875 | -38.125 | -39.875 | -39.875 |
| **75th** | | | | | | | | |
| *Habitats compared to quantiles within 2005-2019 baseline* | | **Current** | **Future** | | | | | |
| | | **2005-2019** | **2046-2055** | | | **2086-2095** | | |
| | | | **ssp126** | **ssp370** | **ssp585** | **ssp126** | **ssp370** | **ssp585** |
| North Atlantic | Max | 39.375 | 40.875 | 40.875 | 41.125 | 40.875 | 41.125 | 41.875 |
| | Min | -36.125 | -37.375 | -37.375 | -37.375 | -37.125 | -37.375 | -38.125 |
| South Atlantic | Max | 38.875 | 38.875 | 39.125 | 39.125 | 39.125 | 39.875 | 40.125 |
| | Min | -31.375 | -31.625 | -32.125 | -32.375 | -31.875 | -32.875 | -33.875 |
| Northwest Indian Ocean | Max | 30.125 | 30.125 | 30.125 | 30.125 | 30.125 | 30.125 | 30.125 |
| | Min | -39.875 | -39.875 | -39.875 | -39.875 | -39.875 | -39.875 | -39.875 |
| Southwest Indian Ocean | Max | 29.875 | 30.125 | 29.875 | 30.125 | 30.125 | 30.125 | 30.125 |
| | Min | -36.375 | -37.375 | -37.375 | -37.625 | -37.375 | -37.875 | -37.875 |
| East Indian Ocean | Max | 30.125 | 30.125 | 30.125 | 30.125 | 30.125 | 30.125 | 30.125 |
| | Min | -39.625 | -39.875 | -39.875 | -39.875 | -39.875 | -39.875 | -39.875 |
| West Pacific | Max | 36.875 | 36.875 | 36.875 | 36.875 | 36.875 | 37.125 | 37.125 |
| | Min | -18.375 | -19.125 | -20.625 | -20.625 | -20.625 | -22.125 | -23.375 |
| East Pacific | Max | 35.625 | 35.625 | 35.875 | 35.875 | 35.875 | 39.875 | 39.875 |
| | Min | -30.375 | -32.375 | -32.875 | -34.125 | -32.375 | -38.375 | -39.875 |

**d)** The top X^th^ of the current distribution (2005 – 2019) continued.

| 90th | | | | | | | | |
|---|---|---|---|---|---|---|---|---|
| *Habitats compared to quantiles within 2005-2019 baseline* | | Current | Future | | | | | |
| | | 2005-2019 | 2046-2055 | | | 2086-2095 | | |
| | | | ssp126 | ssp370 | ssp585 | ssp126 | ssp370 | ssp585 |
| North Atlantic | Max | 37.125 | 37.125 | 37.625 | 37.625 | 37.625 | 39.375 | 39.375 |
| | Min | -34.125 | -35.125 | -35.125 | -35.375 | -35.375 | -35.875 | -36.375 |
| South Atlantic | Max | 37.125 | 37.625 | 37.625 | 37.375 | 37.125 | 38.125 | 37.625 |
| | Min | -30.125 | -30.125 | -30.125 | -30.375 | -30.125 | -31.125 | -31.875 |
| Northwest Indian Ocean | Max | 30.125 | 30.125 | 30.125 | 30.125 | 30.125 | 30.125 | 30.125 |
| | Min | -38.125 | -38.375 | -38.375 | -38.375 | -38.375 | -39.875 | -39.875 |
| Southwest Indian Ocean | Max | 28.375 | 28.375 | 28.625 | 28.375 | 28.375 | 29.375 | 29.375 |
| | Min | -34.875 | -36.625 | -36.625 | -36.625 | -36.625 | -36.875 | -36.875 |
| East Indian Ocean | Max | 30.125 | 29.625 | 29.625 | 29.625 | 29.625 | 29.125 | 29.375 |
| | Min | -39.125 | -39.125 | -39.375 | -39.375 | -39.125 | -39.875 | -39.875 |
| West Pacific | Max | 25.125 | 36.875 | 36.875 | 36.875 | 36.875 | 36.875 | 36.875 |
| | Min | -18.125 | -18.375 | -18.375 | -18.375 | -18.375 | -19.375 | -20.625 |
| East Pacific | Max | 29.875 | 33.875 | 34.125 | 34.875 | 34.875 | 35.625 | 36.375 |
| | Min | -20.875 | -23.625 | -23.625 | -23.625 | -23.625 | -25.875 | -27.125 |

| 95th | | | | | | | | |
|---|---|---|---|---|---|---|---|---|
| *Habitats compared to quantiles within 2005-2019 baseline* | | Current | Future | | | | | |
| | | 2005-2019 | 2046-2055 | | | 2086-2095 | | |
| | | | ssp126 | ssp370 | ssp585 | ssp126 | ssp370 | ssp585 |
| North Atlantic | Max | 35.375 | 35.625 | 35.875 | 35.625 | 35.625 | 35.625 | 35.375 |
| | Min | -33.125 | -33.625 | -33.625 | -33.625 | -33.625 | -33.625 | -33.375 |
| South Atlantic | Max | 35.625 | 35.625 | 36.375 | 36.375 | 36.375 | 36.875 | 36.875 |
| | Min | -29.375 | -29.375 | -29.375 | -29.625 | -29.375 | -29.875 | -29.875 |
| Northwest Indian Ocean | Max | 30.125 | 30.125 | 30.125 | 30.125 | 30.125 | 30.125 | 30.125 |
| | Min | -38.125 | -38.125 | -38.125 | -38.125 | -38.125 | -38.375 | -38.375 |
| Southwest Indian Ocean | Max | 28.375 | 28.375 | 28.375 | 28.375 | 28.375 | 28.375 | 28.875 |
| | Min | -34.375 | -36.375 | -36.375 | -36.625 | -36.375 | -36.875 | -36.875 |
| East Indian Ocean | Max | 30.125 | 29.375 | 29.375 | 29.125 | 29.125 | 22.875 | 22.875 |
| | Min | -38.875 | -39.125 | -39.125 | -39.125 | -39.125 | -39.375 | -39.875 |
| West Pacific | Max | 24.375 | 25.375 | 36.875 | 36.875 | 36.875 | 36.875 | 36.875 |
| | Min | -17.125 | -18.125 | -18.125 | -18.125 | -18.125 | -18.375 | -18.375 |
| East Pacific | Max | 28.375 | 27.625 | 28.125 | 29.125 | 27.625 | 33.625 | 34.875 |
| | Min | -17.875 | -23.125 | -23.125 | -23.125 | -23.125 | -23.875 | -25.125 |

**Table S3 |** Summary of locations from satellite tracks for 348 whale sharks tagged between 2005 and 2019 in the Atlantic, Indian, and Pacific Oceans. Tag ratios denote ARGOS:PSAT tracks, sex ratios denote Male:Female:Unknown individuals, and size ratios denote Large:Small of sexed individuals. Region abbreviations: NA, north Atlantic; SA, south Atlantic; NIO, northwest Indian Ocean; SIO, southwest Indian Ocean; WP, west Pacific; EP, east Pacific.

| Region | Total tracks | Tag ratio | Sex ratio | Size ratio | Tracking date range | Sum tracking duration (d) | Mean tracking duration (d) | Sum locations | Mean locations | Total distance travelled (km) | Mean distance travelled (km) | Mean (± s.d.) speed (km $d^{-1}$) |
|---|---|---|---|---|---|---|---|---|---|---|---|---|
| NA | 39 | 12:27 | 21:18:00 | 33:06 | 2005-2019 | 3388 | 86.87 | 2017 | 51.72 | 83143.14 | 2131.88 | 25.45 (14.48) |
| SA | 14 | 05:09 | 07:07:00 | 08:01 | 2010-2019 | 1130 | 80.71 | 331 | 23.64 | 15918.37 | 1137.03 | 16.68 (17.06) |
| NIO | 44 | 07:37 | 16:14:14 | 09:31 | 2009-2014 | 3261 | 74.11 | 986 | 22.41 | 27632.11 | 628 | 10.51 (11.96) |
| SIO | 26 | 22:04 | 20:06:00 | 08:18 | 2006-2017 | 1441 | 55.42 | 946 | 36.38 | 30910.99 | 1188.88 | 27.30 (19.54) |
| EIO | 74 | 74:00 | 44:17:13 | 33:40 | 2005-2019 | 10539 | 142.42 | 4342 | 58.68 | 129444.70 | 1749.25 | 18.95 (13.72) |
| WP | 62 | 61:01 | 52:06:04 | 10:51 | 2009-2019 | 15709 | 253.37 | 7033 | 113.44 | 1535510 | 2476.63 | 12.70 (12.22) |
| EP | 89 | 75:14 | 05:38:46 | 37:06 | 2007-2018 | 7362 | 82.72 | 3090 | 34.72 | 128131.90 | 1439.68 | 24.05 (23.66) |

**Table S4** | Summary of essential ocean variables (EOV) explored during model development and selection including their associated units spatial (Res, as a fraction of 1 degree) and temporal resolutions downloaded from the source listed. Resolutions were standardised following the processes outlined in the Online methods section.

| EOV | Description | Units | Res | Temporal | Source (*ftp://my.cmems-du.eu/Core/) |
|---|---|---|---|---|---|
| CHL | Chlorophyll-*a* | mg / $m^{-3}$ | 24 | Monthly | http://hermes.acri.fr/index.php?class=archive |
| O2 | Dissolved oxygen | mmol / $m^{-3}$ | 4 | Weekly | *global_reanalysis_bio_001_029/global-reanalysis-bio-001-029-daily |
| O2100m | Dissolved oxygen at 100m depth | mmol / $m^{-3}$ | 4 | Weekly | *global_reanalysis_bio_001_029/global-reanalysis-bio-001-029-daily |
| PP | Primary productivity | g $m^{-3}$ / day | 12 | Monthly | *global_reanalysis_bio_001_029/global-reanalysis-bio-001-029-monthly |
| PP100m | Primary productivity at 100m depth | g $m^{-3}$ / day | 12 | Monthly | *global_reanalysis_bio_001_029/global-reanalysis-bio-001-029-monthly |
| PHYC | Phytoplankton concentration | mmol / $m^{-3}$ | 12 | Monthly | *global_reanalysis_bio_001_029/global-reanalysis-bio-001-029-monthly |
| PHYC100m | Phytoplankton concentration at 100m depth | mmol / $m^{-3}$ | 12 | Monthly | *global_reanalysis_bio_001_029/global-reanalysis-bio-001-029-monthly |
| SST | Sea surface temperature | °C | 20 | Weekly | *sst_glo_sst_l4_rep_observations_010_011/metoffice-glo-sst-l4-rep-obs-sst |
| SSTSlope | Maximum slope from surrounding pixels | °C / 1° | 20 | Weekly | *sst_glo_sst_l4_rep_observations_010_011/metoffice-glo-sst-l4-rep-obs-sst |
| TMP100m | Temperature at 100m depth | °C | 12 | Monthly | *global_reanalysis_phy_001_030/global-reanalysis-phy-001-030-daily |
| TMP100mSlope | Maximum slope from surrounding pixels | °C / 1° | 12 | Monthly | *global_reanalysis_phy_001_030/global-reanalysis-phy-001-030-daily |
| VELOCITY | Geostrophic velocity | m / s | 12 | Weekly | *global_reanalysis_phy_001_030/global-reanalysis-phy-001-030-daily |
| SAL | Salinity | psu | 12 | Weekly | *global_reanalysis_phy_001_030/global-reanalysis-phy-001-030-daily |
| SAL100m | Salinity at 100m depth | psu | 12 | Weekly | *global_reanalysis_phy_001_030/global-reanalysis-phy-001-030-daily |
| SSH | Sea surface height | m / 1° | 12 | Weekly | *global_reanalysis_phy_001_030/global-reanalysis-phy-001-030-daily |
| SSHSlope | Maximum slope from surrounding pixels | m | 12 | Weekly | *global_reanalysis_phy_001_030/global-reanalysis-phy-001-030-daily |
| MLD | Mixed layer depth | m | 12 | Weekly | *global_reanalysis_phy_001_030/global-reanalysis-phy-001-030-daily |
| ZOOC | Epipelagic zooplankton concentration | gC $m^{-2}$ | 4 | Weekly | *global_reanalysis_bio_001_033/global-reanalysis-bio-001-033-weekly |
| EPI_ZOOC | Zooplankton concentration | gC $m^{-2}$ | 4 | Weekly | *global_reanalysis_bio_001_033/global-reanalysis-bio-001-033-weekly |
| EPI_MNKC | Epipelagic micronekton concentration | WWg $m^{-2}$ | 4 | Weekly | *global_reanalysis_bio_001_033/global-reanalysis-bio-001-033-weekly |
| UMESO_MNKC | Upper-mesopelagic micronekton concentration | WWg $m^{-2}$ | 4 | Weekly | *global_reanalysis_bio_001_033/global-reanalysis-bio-001-033-weekly |
| LMESO_MNKC | Lower-mesopelagic micronekton concentration | WWg $m^{-2}$ | 4 | Weekly | *global_reanalysis_bio_001_033/global-reanalysis-bio-001-033-weekly |
| DEPTH | Gebco 30 arcsecond bathymetry | m | 120 | -- | https://www.gebco.net/data_and_products/gridded_bathymetry_data/#global |
| SLOPE | Maximum slope from surrounding pixels | m / 1° | 120 | -- | https://www.gebco.net/data_and_products/gridded_bathymetry_data/#global |
| RUGOSITY | Standard deviation of surrounding pixels | m / 1° | 120 | -- | https://www.gebco.net/data_and_products/gridded_bathymetry_data/#global |
| PROXC | Distance to 0 m in Gebco 30 s | km | -- | -- | https://www.gebco.net/data_and_products/gridded_bathymetry_data/#global |
| PROX200m | Distance to 200 m depth in Gebco 30 s | km | -- | -- | https://www.gebco.net/data_and_products/gridded_bathymetry_data/#global |
| PROX1km | Distance to 1000 m in Gebco 30 s | km | -- | -- | https://www.gebco.net/data_and_products/gridded_bathymetry_data/#global |

**Table S5 |** Summary of tracking data used in model training process. NA, north Atlantic; SA, south Atlantic; NIO, northwest Indian Ocean; SIO, southwest Indian Ocean; WP, west Pacific; EP, east Pacific.

**a)** Movement and distance metrics calculated before and after the data thinning procedure which removed consecutive daily locations (see Methods for more details).

| | Before | | | | After | |
|---|---|---|---|---|---|---|
| | Daily movement (km d$^{-1}$) | | Step length (km) | | | |
| **Region** | **Mean** | **± S.D.** | **Mean** | **± S.D.** | **Mean** | **± S.D.** |
| North Atlantic | 25.45 | 14.48 | 42.03 | 83.01 | 77.46 | 83.01 |
| South Atlantic | 16.68 | 17.06 | 50.22 | 116.66 | 96.43 | 116.66 |
| Northwest Indian Ocean | 10.51 | 11.96 | 29.33 | 50.78 | 53.01 | 50.78 |
| Southwest Indian Ocean | 27.30 | 19.54 | 33.60 | 62.04 | 61.50 | 62.04 |
| East Indian Ocean | 18.95 | 13.72 | 30.33 | 79.27 | 55.89 | 79.27 |
| West Pacific | 12.70 | 12.22 | 22.03 | 54.51 | 39.95 | 54.51 |
| East Pacific | 24.05 | 23.66 | 42.70 | 148.27 | 81.25 | 148.27 |
| Global | 19.27 | 17.68 | 30.64 | 83.77 | 56.48 | 120.15 |

**b)** Monthly summary of tracking locations within each region used in the model training process.

| Month | NA | SA | NIO | SIO | EIO | WP | EP | Sum | % |
|---|---|---|---|---|---|---|---|---|---|
| JAN | 95 | 54 | 84 | 98 | 252 | 535 | 268 | 1386 | 7.39 |
| FEB | 3 | 72 | 19 | 6 | 199 | 503 | 262 | 1064 | 5.68 |
| MAR | 16 | 62 | 19 | 27 | 134 | 563 | 263 | 1084 | 5.78 |
| APR | 0 | 79 | 161 | 1 | 114 | 614 | 165 | 1134 | 6.05 |
| MAY | 66 | 28 | 170 | 4 | 377 | 704 | 138 | 1487 | 7.93 |
| JUN | 170 | 6 | 133 | 0 | 386 | 690 | 160 | 1545 | 8.24 |
| JUL | 241 | 9 | 88 | 23 | 580 | 623 | 257 | 1821 | 9.71 |
| AUG | 446 | 21 | 105 | 110 | 687 | 629 | 305 | 2303 | 12.29 |
| SEP | 289 | 0 | 29 | 33 | 571 | 471 | 462 | 1855 | 9.90 |
| OCT | 236 | 0 | 57 | 93 | 545 | 409 | 375 | 1715 | 9.15 |
| NOV | 244 | 0 | 70 | 333 | 334 | 627 | 233 | 1841 | 9.82 |
| DEC | 211 | 0 | 51 | 218 | 163 | 665 | 202 | 1510 | 8.06 |

**Table S6 |** Hypotheses developed to help identify the environmental drivers important for whale shark movements from a total of 28 essential ocean variables (EOVs, see Table S4 for EOV descriptions and units) based on previous assessments of the species and other pelagic sharks.

| Hypothesis | EOVs and description |
|---|---|
| HYP #1 | DEPTH, RUGOSITY, PROXC, SSH, SSHSlope, VELOCITY<br><br>Observed whale shark presence is explained mostly by their relationship with habitat types characterised by fixed physical features and dynamic ocean topography. |
| HYP #2 | DEPTH, TMP100m, MLD, O2100m, SAL100m, PP<br><br>Observed whale shark presence is explained mostly by their relationship with subsurface oceanographic variables and affected by the surface thermocline, depth and surface primary productivity. |
| HYP #3 | DEPTH, PROXC, SST, EPI_ZOOC, EPI_MNKC, MLD, SAL, SSH, CHL, O2100m<br><br>Observed whale shark presence is explained mostly by their relationship with surface temperature and prey (and a proxy for prey) and affected by the surface thermocline, topography, salinity, oxygen concentration at depth and distance to coastal areas where they aggregate. |
| HYP #4 | DEPTH, CHL, SST, PP, EPI_ZOOC, EPI_MNKC, TMP100m, SSTSlope, TMP100mSlope<br><br>Observed whale shark presence is explained mostly by their relationship with areas characterised by productivity and surface/ subsurface temperature and gradients. |
| HYP #5 | DEPTH, MLD, EPI_MNKC, SSTSlope, CHL, PP, O2<br><br>Observed whale shark presence is explained mostly by their relationship with habitat types characterised by surface temperature gradients (fronts; thermoclines), dissolved oxygen and prey/ productivity (and a proxy for prey) and affected by the surface thermocline and depth. |
| HYP #6 | DEPTH, PROXC, TMP100m, MLD, O2100m, LMESO_MNKC, VELOCITY<br><br>Observed whale shark presence is explained mostly by their relationship by the interaction between subsurface temperature, oxygen and prey fields and affected by the surface thermocline, velocity, and distance to regions of upwelling. |
| HYP #7 | DEPTH, O2100m, PROXC, SST, EPI_MNKC, CHL, SAL, TMP100m, TMP100mSlope, PP100m<br><br>Observed whale shark presence is explained mostly by their relationship with habitat types characterised by surface and subsurface temperature, salinity and prey availability and distance to coastal areas where they aggregate. |
| HYP #8 | DEPTH, TMP100m, TMP100mSlope, SAL100m, O2100m, PP100m, LMESO_MNKC<br><br>Observed whale shark presence is explained mostly by their relationship with habitat types characterised by subsurface temperature gradients, productivity, and salinity. |

**Table S7 |** Habitat suitability metric comparisons between Generalised Additive Models (GAM) and Bayesian Additive Regression Trees (BART) where core habitats are defined as the top 90th of the current distribution. All areas are estimated as million km$^2$. Comparison metrics are:

**a)** Total coverage of each region considered core habitat as a percentage.

| **Total percentage coverage** | | | | | | | | |
|---|---|---|---|---|---|---|---|---|
| *Habitats compared to quantiles within 2005-2019 baseline* | | **Present** | **Future** | | | | | |
| | | **2005-2019** | **2046-2055** | | | **2086-2095** | | |
| | | | **ssp126** | **ssp370** | **ssp585** | **ssp126** | **ssp370** | **ssp585** |
| North Atlantic | GAM % | 10.44 | 15.64 | 17.22 | 17.52 | 16.73 | 24.12 | 25.02 |
| | BART % | 10.43 | 13.13 | 15.02 | 16.08 | 14.35 | 24.42 | 28.21 |
| South Atlantic | GAM % | 10.37 | 9.11 | 7.79 | 6.65 | 9.98 | 4.49 | 3.46 |
| | BART % | 10.28 | 13.10 | 13.50 | 13.74 | 12.64 | 14.73 | 15.80 |
| Northwest Indian Ocean | GAM % | 10.16 | 10.81 | 10.95 | 11.25 | 10.79 | 12.94 | 14.15 |
| | BART % | 9.88 | 9.61 | 9.88 | 10.10 | 9.58 | 11.53 | 13.30 |
| Southwest Indian Ocean | GAM % | 10.13 | 11.82 | 12.89 | 13.57 | 11.41 | 20.97 | 25.9 |
| | BART % | 10.26 | 12.34 | 13.03 | 13.23 | 11.98 | 15.25 | 16.10 |
| East Indian Ocean | GAM % | 10.22 | 8.95 | 8.49 | 8.05 | 9.21 | 5.76 | 4.72 |
| | BART % | 10.22 | 8.39 | 8.10 | 7.75 | 8.41 | 6.71 | 6.09 |
| West Pacific | GAM % | 10.39 | 11.28 | 10.80 | 10.82 | 11.20 | 11.40 | 11.61 |
| | BART % | 10.38 | 12.07 | 12.03 | 11.90 | 12.43 | 11.16 | 10.61 |
| East Pacific | GAM % | 10.50 | 8.83 | 8.18 | 7.67 | 9.63 | 3.55 | 2.44 |
| | BART % | 10.51 | 8.31 | 7.77 | 7.46 | 9.19 | 3.80 | 2.50 |

**b)** Total area (million km$^2$) within each region considered core habitat.

| Total area | | | | | | | | |
|---|---|---|---|---|---|---|---|---|
| *Habitats compared to quantiles within 2005-2019 baseline *areas given as million km$^2$* | | **Present** | **Future** | | | | | |
| | | | **2046-2055** | | | **2086-2095** | | |
| | | **2005-2019** | **ssp126** | **ssp370** | **ssp585** | **ssp126** | **ssp370** | **ssp585** |
| North Atlantic | GAM Area / km$^2$ | 6.38 | 9.56 | 10.53 | 10.71 | 10.23 | 14.75 | 15.30 |
| | BART Area / km$^2$ | 6.38 | 8.03 | 9.18 | 9.83 | 8.77 | 14.93 | 17.25 |
| South Atlantic | GAM Area / km$^2$ | 6.34 | 5.57 | 4.76 | 4.06 | 6.10 | 2.75 | 2.12 |
| | BART Area / km$^2$ | 6.28 | 8.01 | 8.26 | 8.40 | 7.73 | 9.01 | 9.66 |
| Northwest Indian Ocean | GAM Area / km$^2$ | 5.25 | 5.59 | 5.67 | 5.82 | 5.58 | 6.69 | 7.32 |
| | BART Area / km$^2$ | 5.11 | 4.97 | 5.11 | 5.22 | 4.96 | 5.96 | 6.88 |
| Southwest Indian Ocean | GAM Area / km$^2$ | 5.24 | 6.12 | 6.67 | 7.02 | 5.90 | 10.85 | 13.40 |
| | BART Area / km$^2$ | 5.30 | 6.38 | 6.74 | 6.84 | 6.20 | 7.89 | 8.33 |
| East Indian Ocean | GAM Area / km$^2$ | 5.29 | 4.63 | 4.39 | 4.17 | 4.77 | 2.98 | 2.44 |
| | BART Area / km$^2$ | 5.29 | 4.34 | 4.19 | 4.01 | 4.35 | 3.47 | 3.15 |
| West Pacific | GAM Area / km$^2$ | 5.16 | 5.61 | 5.37 | 5.38 | 5.57 | 5.67 | 5.77 |
| | BART Area / km$^2$ | 5.16 | 6.00 | 5.98 | 5.92 | 6.18 | 5.55 | 5.28 |
| East Pacific | GAM Area / km$^2$ | 9.06 | 7.61 | 7.05 | 6.62 | 8.30 | 3.06 | 2.11 |
| | BART Area / km$^2$ | 9.06 | 7.16 | 6.70 | 6.43 | 7.92 | 3.27 | 2.15 |

**Table S8 |** Summary of global models from eight hypotheses (HYP, see Table S6 for hypothesis descriptions) built from a total of 28 essential ocean variables (EOVs, see Table S4 for EOV descriptions and units) showing Degrees of Freedom (df), the coefficient of determination ($r^2$), explained deviance (dev%), Akaike Information Criterion (AIC) and delta AIC (*d*AIC). Each hypothesis was trained on the full dataset with locations in all seven regions (note coastal locations [< 100m depth] removed by N/A row filtering, n = 69,206).

| Hypothesis | Modelled EOVs | df | $r^2$ | dev% | AIC | *d*AIC |
|---|---|---|---|---|---|---|
| HYP #1 | DEPTH, RUGOSITY, PROXC, SSH, SSHSlope, VELOCITY | 50.52 | 0.37 | 40.60 | 20767.05 | 5707.99 |
| HYP #2 | O2100m*TMP100m DEPTH, TMP100m, O2100m, MLD, SAL100m, PP | 51.97 | 0.50 | 51.10 | 17115.10 | 2056.03 |
| HYP #3 | DEPTH, O2100m, SSH, PROXC, SST, MLD, EPI_MNKC, CHL, SAL | 60.2 | 0.41 | 54.40 | 15988.56 | 929.49 |
| HYP #4 | DEPTH, SST, SSTSlope, CHL, TMP100m, TMP100mSlope, PP, EPI_MNKC | 54.75 | 0.47 | 50.20 | 17421.46 | 2362.39 |
| HYP #5 | SSTSlope, MLD, O2, PP, EPI_MNKC, DEPTH, CHL | 41.97 | 0.40 | 45.50 | 19171.77 | 4112.70 |
| HYP #6 | DEPTH, PROXC, VELOCITY, TMP100m, O2100m, MLD, LMESO_MNKC | 51.56 | 0.39 | 43.00 | 19913.98 | 4854.91 |
| HYP #7 | DEPTH, O2100m, PROXC, SST, EPI_MNKC, CHL, SAL, TMP100m, TMP100mSlope, PP100m | 41.97 | 0.54 | 57.10 | 15059.06 | 0 |
| HYP #8 | DEPTH, TMP100m, TMP100mSlope, SAL100m, O2100m, PP100m, LMESO_MNKC | 53.02 | 0.50 | 51.70 | 16912.69 | 1853.87 |

**Table S9** | Summary of region-based models built from essential ocean variables (EOVs) included in the best performing global model (Table S8):

**a)** Showing Degrees of Freedom (df), the coefficient of determination ($r^2$) and explained deviance (dev%). Each model was trained on the region specific datasets (NA, north Atlantic, n = 8,645; SA, south Atlantic, n = 1,017; NIO, northwest Indian Ocean, n = 5,676; SIO, southwest Indian Ocean, n = 5,092; EIO, east Indian Ocean, n = 14,313; WP, west Pacific, n = 24,772; EP, east Pacific, n = 16,380, note coastal locations [< 100m depth] retained, see Fig. S29 and Table S3 for region dataset summaries). Shark ID wasn't included as a random effect in these summaries.

| Region | df | $r^2$ | dev% |
|---|---|---|---|
| North Atlantic | 23.81 | 0.71 | 72.50 |
| South Atlantic | 15.41 | 0.88 | 87.00 |
| Northwest Indian Ocean | 12.77 | 0.95 | 94.40 |
| Southwest Indian Ocean | 21.24 | 0.69 | 72.30 |
| East Indian Ocean | 29.78 | 0.82 | 81.20 |
| West Pacific | 24.58 | 0.79 | 79.90 |
| East Pacific | 27.10 | 0.77 | 78.00 |

**b)** EOV summaries for the north Atlantic.

| Variable | Min | Max | Mean | Median | ± S.D. |
|---|---|---|---|---|---|
| DEPTH | 4.34 | 5532.67 | 1525.41 | 954.10 | 1431.31 |
| SLOPE | 46.67 | 10069.89 | 2651.11 | 2209.20 | 2182.76 |
| RUGOSITY | 17.34 | 4589.92 | 1280.63 | 1097.37 | 1029.23 |
| PROXC | 1.00 | 1350.00 | 143.44 | 76.00 | 184.06 |
| PROX200m | 4.00 | 1132.00 | 103.95 | 61.00 | 146.77 |
| PROX1km | 4.00 | 896.00 | 85.48 | 57.00 | 88.73 |
| SST | 19.70 | 30.88 | 27.94 | 28.69 | 2.13 |
| SSTSlope | 0.10 | 8.90 | 1.38 | 0.86 | 1.38 |
| TMP100m | 8.44 | 28.35 | 21.60 | 21.38 | 3.41 |
| TMP100mSlope | 0.53 | 54.15 | 10.05 | 6.90 | 8.70 |
| SSH | -0.42 | 0.51 | -0.01 | -0.06 | 0.16 |
| SSHSlope | 0.01 | 0.88 | 0.26 | 0.22 | 0.18 |
| MLD | 8.39 | 99.29 | 23.16 | 16.01 | 16.02 |
| CHL | 0.05 | 10.23 | 0.40 | 0.19 | 0.61 |
| O2 | 193.34 | 226.44 | 202.39 | 201.62 | 5.15 |
| O2100m | 125.76 | 229.12 | 199.14 | 202.35 | 16.86 |
| SAL | 31.75 | 36.98 | 36.01 | 36.07 | 0.49 |
| SAL100m | 35.47 | 37.17 | 36.50 | 36.52 | 0.18 |
| VELOCITY | 0.01 | 1.14 | 0.33 | 0.27 | 0.23 |
| PHYC | 0.84 | 3.51 | 1.39 | 1.35 | 0.29 |
| PHYC100m | 0.29 | 1.14 | 0.74 | 0.78 | 0.17 |
| PP | 0.93 | 23.13 | 5.39 | 3.19 | 4.65 |
| PP100m | 0.05 | 5.97 | 2.05 | 1.83 | 1.36 |
| ZOOC | 0.06 | 2.27 | 0.27 | 0.20 | 0.25 |
| EPI_MNKC | 0.17 | 8.85 | 1.50 | 0.97 | 1.34 |
| LMESO_MNKC | 0.71 | 4.65 | 2.17 | 2.05 | 0.56 |
| UMESO_MNKC | 0.08 | 2.07 | 0.60 | 0.51 | 0.31 |
| EPI_ZOOC | 4.17E-04 | 4.06E-02 | 3.00E-03 | 2.00E-03 | 4.00E-03 |

**c)** EOV summaries for the south Atlantic.

| Variable | Min | Max | Mean | Median | ± S.D. |
|---|---|---|---|---|---|
| DEPTH | 2856.62 | 5729.02 | 3868.95 | 3761.04 | 468.93 |
| SLOPE | 101.22 | 7141.40 | 3734.77 | 4189.24 | 2156.13 |
| RUGOSITY | 53.72 | 2990.28 | 1562.45 | 1660.48 | 857.58 |
| PROXC | 6.00 | 1000.00 | 278.23 | 143.00 | 289.33 |
| PROX200m | 4.00 | 996.00 | 216.79 | 45.00 | 259.83 |
| PROX1km | 5.00 | 792.00 | 149.57 | 42.00 | 193.87 |
| SST | 22.34 | 29.36 | 25.15 | 25.23 | 1.32 |
| SSTSlope | 0.16 | 1.03 | 0.48 | 0.46 | 0.18 |
| TMP100m | 17.37 | 23.26 | 19.54 | 19.10 | 1.20 |
| TMP100mSlope | 0.72 | 4.45 | 2.18 | 2.10 | 0.72 |
| SSH | -0.12 | 0.18 | 0.00 | 0.00 | 0.05 |
| SSHSlope | 0.01 | 0.08 | 0.04 | 0.04 | 0.02 |
| MLD | 10.55 | 86.22 | 26.78 | 20.33 | 16.94 |
| CHL | 0.04 | 0.19 | 0.08 | 0.07 | 0.02 |
| O2 | 199.91 | 222.57 | 211.40 | 210.54 | 4.50 |
| O2100m | 107.18 | 236.01 | 217.01 | 221.93 | 18.37 |
| SAL | 35.79 | 37.05 | 36.59 | 36.57 | 0.22 |
| SAL100m | 35.76 | 36.77 | 36.22 | 36.18 | 0.23 |
| VELOCITY | 0.04 | 0.68 | 0.12 | 0.11 | 0.06 |
| PHYC | 0.83 | 1.63 | 1.28 | 1.30 | 0.21 |
| PHYC100m | 0.51 | 1.05 | 0.89 | 0.90 | 0.11 |
| PP | 0.40 | 13.52 | 1.22 | 1.03 | 1.52 |
| PP100m | 0.27 | 4.60 | 2.63 | 2.72 | 1.02 |
| ZOOC | 0.14 | 0.75 | 0.31 | 0.29 | 0.11 |
| EPI_MNKC | 0.23 | 1.69 | 0.98 | 1.05 | 0.33 |
| LMESO_MNKC | 1.37 | 4.09 | 2.71 | 2.90 | 0.69 |
| UMESO_MNKC | 0.40 | 1.69 | 0.91 | 0.88 | 0.29 |
| EPI_ZOOC | 9.18E-04 | 8.95E-03 | 3.00E-03 | 2.00E-03 | 1.00E-03 |

**d)** EOV summaries for the northwest Indian Ocean.

| Variable | Min | Max | Mean | Median | ± S.D. |
|---|---|---|---|---|---|
| DEPTH | 6.01 | 2148.26 | 334.32 | 201.52 | 334.39 |
| SLOPE | 40.48 | 4822.02 | 1400.48 | 1419.90 | 1112.20 |
| RUGOSITY | 17.50 | 1937.22 | 638.55 | 638.57 | 515.52 |
| PROXC | 1.00 | 140.00 | 44.33 | 44.00 | 28.28 |
| PROX200m | 3.00 | 745.00 | 150.96 | 38.00 | 215.49 |
| PROX1km | 4.00 | 844.00 | 188.00 | 59.00 | 246.62 |
| SST | 21.47 | 34.06 | 29.14 | 29.80 | 2.72 |
| SSTSlope | 0.28 | 4.22 | 1.42 | 1.37 | 0.55 |
| TMP100m | 6.55 | 25.98 | 22.23 | 23.26 | 3.03 |
| TMP100mSlope | 0.46 | 53.21 | 12.71 | 10.74 | 8.79 |
| SSH | -0.06 | 0.47 | 0.19 | 0.20 | 0.09 |
| SSHSlope | 0.02 | 1.30 | 0.24 | 0.14 | 0.24 |
| MLD | 8.51 | 55.22 | 16.95 | 13.78 | 9.05 |
| CHL | 0.07 | 6.42 | 1.10 | 0.91 | 0.89 |
| O2 | 179.43 | 220.36 | 196.68 | 196.40 | 6.89 |
| O2100m | 41.32 | 208.46 | 165.50 | 179.88 | 36.14 |
| SAL | 31.05 | 38.75 | 37.22 | 37.09 | 0.82 |
| SAL100m | 35.59 | 40.05 | 38.62 | 38.94 | 1.22 |
| VELOCITY | 0.02 | 0.57 | 0.13 | 0.12 | 0.07 |
| PHYC | 1.04 | 2.92 | 1.64 | 1.67 | 0.25 |
| PHYC100m | 0.08 | 1.01 | 0.75 | 0.83 | 0.20 |
| PP | 1.29 | 39.83 | 6.41 | 4.65 | 5.67 |
| PP100m | 0.01 | 5.80 | 2.62 | 2.90 | 1.53 |
| ZOOC | 0.07 | 3.77 | 0.46 | 0.28 | 0.44 |
| EPI_MNKC | 0.69 | 13.10 | 4.18 | 3.94 | 2.18 |
| LMESO_MNKC | 0.10 | 8.24 | 1.31 | 0.75 | 1.33 |
| UMESO_MNKC | 1.16E-06 | 173.02 | 1.04 | 0.44 | 6.90 |
| EPI_ZOOC | 6.47E-04 | 9.92E-02 | 8.00E-03 | 4.00E-03 | 1.00E-02 |

**e)** EOV summaries for the southwest Indian Ocean.

| Variable | Min | Max | Mean | Median | ± S.D. |
|---|---|---|---|---|---|
| DEPTH | 82.44 | 4987.47 | 1311.89 | 540.47 | 1408.22 |
| SLOPE | 233.91 | 8053.63 | 3092.56 | 2853.27 | 1539.68 |
| RUGOSITY | 121.86 | 3636.24 | 1411.68 | 1279.17 | 713.14 |
| PROXC | 3.00 | 597.00 | 74.92 | 16.00 | 115.87 |
| PROX200m | 5.00 | 494.00 | 58.59 | 19.00 | 89.93 |
| PROX1km | 5.00 | 468.00 | 64.81 | 40.00 | 78.97 |
| SST | 22.01 | 30.07 | 27.02 | 27.90 | 2.23 |
| SSTSlope | 0.14 | 3.54 | 1.15 | 1.07 | 0.65 |
| TMP100m | 9.58 | 25.50 | 20.79 | 21.89 | 3.09 |
| TMP100mSlope | 0.19 | 48.41 | 8.33 | 2.65 | 9.92 |
| SSH | 0.01 | 0.98 | 0.48 | 0.54 | 0.18 |
| SSHSlope | 0.01 | 2.63 | 0.44 | 0.12 | 0.52 |
| MLD | 8.09 | 80.74 | 15.01 | 13.12 | 7.39 |
| CHL | 0.05 | 5.15 | 0.49 | 0.29 | 0.63 |
| O2 | 193.17 | 220.00 | 205.25 | 203.14 | 5.97 |
| O2100m | 94.85 | 237.05 | 177.23 | 187.34 | 28.19 |
| SAL | 31.96 | 35.57 | 35.15 | 35.18 | 0.33 |
| SAL100m | 34.83 | 35.72 | 35.23 | 35.26 | 0.12 |
| VELOCITY | 0.01 | 1.23 | 0.26 | 0.19 | 0.21 |
| PHYC | 1.03 | 10.65 | 1.62 | 1.48 | 0.74 |
| PHYC100m | 0.32 | 1.09 | 0.77 | 0.85 | 0.17 |
| PP | 0.92 | 198.85 | 9.03 | 2.68 | 17.09 |
| PP100m | 0.15 | 5.81 | 2.45 | 3.10 | 1.51 |
| ZOOC | 0.10 | 2.00 | 0.37 | 0.29 | 0.26 |
| EPI_MNKC | 0.19 | 4.02 | 0.98 | 0.98 | 0.56 |
| LMESO_MNKC | 0.58 | 3.12 | 1.85 | 1.82 | 0.40 |
| UMESO_MNKC | 0.13 | 1.30 | 0.55 | 0.54 | 0.17 |
| EPI_ZOOC | 6.71E-04 | 2.54E-02 | 5.00E-03 | 3.00E-03 | 4.00E-03 |

**f)** EOV summaries for the east Indian Ocean.

| Variable | Min | Max | Mean | Median | ± S.D. |
|---|---|---|---|---|---|
| DEPTH | 10.14 | 5943.83 | 1347.34 | 604.56 | 1705.21 |
| SLOPE | 75.82 | 11482.80 | 3046.79 | 2886.52 | 1553.00 |
| RUGOSITY | 39.37 | 5590.73 | 1444.73 | 1309.04 | 772.49 |
| PROXC | 1.00 | 1293.00 | 99.31 | 10.00 | 186.50 |
| PROX200m | 3.00 | 1286.00 | 88.13 | 13.00 | 172.54 |
| PROX1km | 4.00 | 1238.00 | 93.04 | 50.00 | 131.78 |
| SST | 19.09 | 30.95 | 26.20 | 26.45 | 2.48 |
| SSTSlope | 0.09 | 16.99 | 2.84 | 1.81 | 2.91 |
| TMP100m | 9.02 | 27.53 | 20.56 | 21.53 | 4.01 |
| TMP100mSlope | 0.17 | 57.84 | 16.39 | 9.34 | 15.78 |
| SSH | -0.01 | 0.84 | 0.43 | 0.50 | 0.23 |
| SSHSlope | 0.01 | 2.92 | 1.02 | 0.43 | 0.98 |
| MLD | 8.45 | 113.17 | 28.69 | 22.79 | 18.59 |
| CHL | 0.05 | 5.80 | 0.50 | 0.40 | 0.42 |
| O2 | 194.57 | 230.40 | 207.89 | 206.88 | 6.78 |
| O2100m | 63.01 | 235.02 | 187.56 | 203.68 | 31.54 |
| SAL | 31.16 | 35.92 | 34.60 | 34.70 | 0.62 |
| SAL100m | 34.24 | 35.94 | 34.91 | 34.91 | 0.30 |
| VELOCITY | 0.01 | 1.54 | 0.19 | 0.14 | 0.15 |
| PHYC | 0.78 | 13.44 | 1.50 | 1.41 | 0.75 |
| PHYC100m | 0.10 | 2.29 | 0.82 | 0.78 | 0.28 |
| PP | 0.38 | 190.16 | 7.61 | 5.39 | 11.06 |
| PP100m | 0.01 | 15.91 | 2.74 | 1.94 | 2.52 |
| ZOOC | 0.06 | 6.76 | 0.98 | 0.66 | 0.93 |
| EPI_MNKC | 0.15 | 15.55 | 2.18 | 1.49 | 1.90 |
| LMESO_MNKC | 0.65 | 7.76 | 2.58 | 2.57 | 0.62 |
| UMESO_MNKC | 0.03 | 3.60 | 0.76 | 0.66 | 0.43 |
| EPI_ZOOC | 6.09E-04 | 1.44E-01 | 1.20E-02 | 8.00E-03 | 1.30E-02 |

**g)** EOV summaries for the west Pacific.

| Variable | Min | Max | Mean | Median | ± S.D. |
|---|---|---|---|---|---|
| DEPTH | 1.06 | 5465.31 | 792.15 | 180.08 | 1222.37 |
| SLOPE | 16.89 | 13726.62 | 2187.14 | 1187.42 | 1929.77 |
| RUGOSITY | 8.17 | 6988.11 | 941.78 | 449.47 | 900.23 |
| PROXC | 1.00 | 562.00 | 37.68 | 9.00 | 75.29 |
| PROX200m | 3.00 | 566.00 | 45.50 | 15.00 | 91.40 |
| PROX1km | 4.00 | 577.00 | 111.81 | 128.00 | 87.45 |
| SST | 24.56 | 31.00 | 29.39 | 29.59 | 0.91 |
| SSTSlope | 0.05 | 3.88 | 0.66 | 0.56 | 0.43 |
| TMP100m | 7.90 | 29.01 | 20.35 | 21.35 | 4.77 |
| TMP100mSlope | 0.06 | 61.37 | 23.96 | 26.61 | 20.32 |
| SSH | 0.01 | 0.88 | 0.62 | 0.64 | 0.11 |
| SSHSlope | 2.37E-03 | 3.27 | 1.60 | 2.10 | 1.22 |
| MLD | 7.02 | 61.10 | 14.12 | 12.06 | 5.77 |
| CHL | 0.04 | 12.87 | 0.56 | 0.35 | 1.00 |
| O2 | 193.79 | 212.02 | 200.56 | 200.10 | 2.53 |
| O2100m | 34.51 | 212.33 | 147.82 | 151.60 | 37.45 |
| SAL | 30.09 | 35.89 | 33.69 | 33.82 | 0.74 |
| SAL100m | 34.09 | 35.66 | 34.99 | 35.06 | 0.31 |
| VELOCITY | 0.01 | 1.24 | 0.18 | 0.14 | 0.15 |
| PHYC | 0.90 | 5.01 | 1.80 | 1.70 | 0.53 |
| PHYC100m | 0.21 | 1.03 | 0.63 | 0.62 | 0.16 |
| PP | 1.11 | 86.76 | 15.39 | 11.90 | 11.84 |
| PP100m | 0.07 | 6.23 | 1.34 | 0.94 | 1.18 |
| ZOOC | 0.05 | 6.47 | 0.27 | 0.22 | 0.21 |
| EPI_MNKC | 0.01 | 17.79 | 1.06 | 0.94 | 0.81 |
| LMESO_MNKC | 0.14 | 5.43 | 2.22 | 2.26 | 0.89 |
| UMESO_MNKC | 0.09 | 5.23 | 0.91 | 0.84 | 0.57 |
| EPI_ZOOC | 3.38E-04 | 1.04E-01 | 4.00E-03 | 3.00E-03 | 6.00E-03 |

**h)** EOV summaries for the east Pacific.

| Variable | Min | Max | Mean | Median | ± S.D. |
|---|---|---|---|---|---|
| DEPTH | 28.68 | 6141.04 | 1951.47 | 2201.48 | 1421.09 |
| SLOPE | 158.30 | 8729.35 | 2235.33 | 2030.37 | 1434.43 |
| RUGOSITY | 78.73 | 4746.58 | 970.08 | 848.59 | 665.69 |
| PROXC | 1.00 | 1675.00 | 208.89 | 81.00 | 305.83 |
| PROX200m | 4.00 | 1681.00 | 203.81 | 62.00 | 303.21 |
| PROX1km | 5.00 | 1223.00 | 157.33 | 75.00 | 199.94 |
| SST | 18.09 | 30.84 | 26.13 | 26.61 | 2.59 |
| SSTSlope | 0.06 | 12.70 | 1.39 | 1.03 | 1.21 |
| TMP100m | 4.52 | 28.02 | 14.29 | 14.56 | 2.99 |
| TMP100mSlope | 0.15 | 48.72 | 9.53 | 1.76 | 12.10 |
| SSH | 0.01 | 1.08 | 0.26 | 0.26 | 0.11 |
| SSHSlope | 0.00 | 1.89 | 0.22 | 0.06 | 0.35 |
| MLD | 8.71 | 89.78 | 16.23 | 11.53 | 8.90 |
| CHL | 0.04 | 12.33 | 1.05 | 0.34 | 1.49 |
| O2 | 177.70 | 244.13 | 211.58 | 210.60 | 6.10 |
| O2100m | 1.03 | 241.73 | 84.55 | 94.06 | 51.94 |
| SAL | 25.23 | 35.60 | 32.97 | 33.41 | 1.79 |
| SAL100m | 33.65 | 35.64 | 34.87 | 34.94 | 0.26 |
| VELOCITY | 0.01 | 1.61 | 0.27 | 0.20 | 0.22 |
| PHYC | 0.87 | 9.01 | 2.02 | 1.67 | 1.13 |
| PHYC100m | 0.05 | 1.43 | 0.51 | 0.43 | 0.28 |
| PP | 0.66 | 147.50 | 19.49 | 11.62 | 21.10 |
| PP100m | 0.01 | 2.18 | 0.37 | 0.13 | 0.55 |
| ZOOC | 0.05 | 6.51 | 1.04 | 0.60 | 1.15 |
| EPI_MNKC | 0.10 | 20.30 | 1.87 | 1.36 | 2.23 |
| LMESO_MNKC | 1.21 | 12.24 | 3.13 | 2.93 | 1.19 |
| UMESO_MNKC | 0.24 | 23.06 | 2.19 | 1.60 | 1.65 |
| EPI_ZOOC | 3.15E-04 | 2.39E-01 | 2.00E-02 | 8.00E-03 | 3.40E-02 |

**i)** Global EOV summaries. Region refers to where the value in the preceding column was recorded.

| Variable | Min | Region | Max | Region | Mean | S.D. |
|---|---|---|---|---|---|---|
| DEPTH | 1.06 | WP | 6141.04 | EP | 1247.23 | 1494.12 |
| SLOPE | 16.89 | WP | 13726.62 | WP | 2475.77 | 1816.92 |
| RUGOSITY | 8.17 | WP | 6988.11 | WP | 1118.13 | 862.86 |
| PROXC | 1.00 | NA \| NIO \| EIO \| EP \| WP | 1675.00 | EP | 98.10 | 189.31 |
| PROX200m | 3.00 | NIO \| EIO \| WP | 1681.00 | EP | 97.09 | 186.98 |
| PROX1km | 4.00 | NA \| NIO \| EIO \| WP | 1238.00 | EIO | 114.44 | 139.48 |
| SST | 18.09 | EP | 34.06 | NIO | 27.74 | 2.50 |
| SSTSlope | 0.05 | WP | 16.99 | EIO | 1.43 | 1.80 |
| TMP100m | 4.52 | EP | 29.01 | WP | 19.40 | 4.74 |
| TMP100mSlope | 0.06 | WP | 61.37 | WP | 15.93 | 16.88 |
| SSH | -0.42 | NA | 1.08 | EP | 0.41 | 0.27 |
| SSHSlope | 2.37E-03 | WP | 3.27 | WP | 0.94 | 1.08 |
| MLD | 7.02 | WP | 113.17 | EIO | 18.85 | 13.06 |
| CHL | 0.04 | EP | 12.87 | WP | 0.62 | 0.97 |
| O2 | 177.70 | EP | 244.13 | EP | 204.48 | 6.93 |
| O2100m | 1.03 | EP | 241.73 | EP | 158.52 | 53.77 |
| SAL | 25.23 | EP | 38.75 | NIO | 34.37 | 1.58 |
| SAL100m | 33.65 | EP | 40.05 | NIO | 35.35 | 0.98 |
| VELOCITY | 0.01 | WP | 1.61 | EP | 0.21 | 0.18 |
| PHYC | 0.78 | EIO | 13.44 | EIO | 1.69 | 0.72 |
| PHYC100m | 0.05 | EP | 2.29 | EIO | 0.70 | 0.26 |
| PP | 0.38 | EIO | 198.85 | SIO | 12.02 | 14.07 |
| PP100m | 0.01 | EP | 15.91 | EIO | 1.82 | 1.89 |
| ZOOC | 0.05 | EP | 6.76 | EIO | 0.57 | 0.76 |
| EPI_MNKC | 0.01 | WP | 20.30 | EP | 1.66 | 1.71 |
| LMESO_MNKC | 3.27E-09 | WP | 65.80 | EIO | 1.84 | 2.32 |
| UMESO_MNKC | 0.10 | NIO | 12.24 | EP | 2.41 | 0.96 |
| EPI_ZOOC | 7.52E-05 | WP | 38.84 | NIO | 1.51 | 0.99 |

* NA, north Atlantic; SA, south Atlantic; NIO, northwest Indian Ocean; SIO, southwest Indian Ocean; EIO, east Indian Ocean; WP, west Pacific; EP, east Pacific.

**Table S10 |** Summary of the performance metrics used to evaluate each run of ten folds based on essential ocean variables (EOVs) included in the best performing region-based hypothesis. Values for accuracy, precision, sensitivity, specificity, true skill statistic (TSS) and Cohen's kappa (kappa) are means and standard deviation (± S.D.) over the 10 runs of randomised data within each fold to assess inter-run variability.

| Region | Test | Mean | ± S.D. |
|---|---|---|---|
| North Atlantic | AUC | 0.98 | 4.44E-03 |
| | kappa | 0.70 | 1.72E-02 |
| | Precision | 0.61 | 2.02E-02 |
| | Sensitivity | 0.96 | 2.02E-02 |
| | Specificity | 0.92 | 7.33E-03 |
| | TSS | 0.88 | 1.77E-02 |
| South Atlantic | AUC | 0.99 | 1.21E-02 |
| | kappa | 0.75 | 1.07E-01 |
| | Precision | 0.70 | 1.40E-01 |
| | Sensitivity | 0.94 | 9.48E-02 |
| | Specificity | 0.93 | 3.91E-02 |
| | TSS | 0.87 | 8.93E-02 |
| Northwest Indian Ocean | AUC | 1.00 | 1.23E-03 |
| | kappa | 0.93 | 1.77E-02 |
| | Precision | 0.89 | 3.46E-02 |
| | Sensitivity | 0.99 | 1.36E-02 |
| | Specificity | 0.99 | 4.50E-03 |
| | TSS | 0.98 | 1.11E-02 |
| Southwest Indian Ocean | AUC | 0.98 | 6.85E-03 |
| | kappa | 0.66 | 4.08E-02 |
| | Precision | 0.55 | 4.34E-02 |
| | Sensitivity | 0.95 | 2.91E-02 |
| | Specificity | 0.92 | 1.47E-02 |
| | TSS | 0.87 | 2.94E-02 |
| East Indian Ocean | AUC | 0.99 | 1.80E-03 |
| | kappa | 0.77 | 2.34E-02 |
| | Precision | 0.68 | 3.10E-02 |
| | Sensitivity | 0.96 | 1.05E-02 |
| | Specificity | 0.95 | 6.81E-03 |
| | TSS | 0.91 | 1.08E-02 |
| West Pacific | AUC | 0.99 | 3.55E-03 |
| | kappa | 0.73 | 2.19E-02 |
| | Precision | 0.64 | 2.84E-02 |
| | Sensitivity | 0.95 | 1.31E-02 |
| | Specificity | 0.94 | 7.65E-03 |
| | TSS | 0.89 | 1.16E-02 |
| East Pacific | AUC | 0.99 | 4.99E-03 |
| | kappa | 0.76 | 1.15E-02 |
| | Precision | 0.66 | 1.62E-02 |
| | Sensitivity | 0.97 | 1.12E-02 |
| | Specificity | 0.95 | 3.46E-03 |
| | TSS | 0.92 | 9.71E-03 |

**Table S11 |** Qualitative external validation summaries for each major ocean basin (Atlantic, Indian Ocean and Pacific) where sub-region name and location are provided (see Extended Data Fig. 1d for mapped locations) along with months in which mean habitat suitability was greater than the annual average (referred to here as season). Occurrence details, quotes (in italics) and references are provided to support each case.

**a)** Atlantic

| Sub-region | Location | Season | Occurrence details | Reference |
|---|---|---|---|---|
| Azores (Portugal) | 27.5° E, 38.0° N | July<br>August<br>September<br>October<br>November | *'It all started towards the end of August, when the first whale sharks were sighted in Princess Alice. Then in September and October, we had many of them much closer to shore, all along the South coast of Pico'*<br>*'The program deploys trained observers since 1998 in about 50 % of the fleet during the entire tuna (warm) season, which typically extends from May to November'…'We analysed 20,079 fishing events recorded by observers between 1998 and 2013, 753 of these events were associated with whale sharks.'*<br>*'The tuna season typically extends from May to November during which boats actively seek for tuna and come into port every 2–8 days, depending on the amount of fish caught. The observers are permanently deployed and observations may occur whenever the boat is out of the harbour. A fishing event with a whale shark associated to tuna will always result in the sighting of the animal very close to the boat's deck'* | 1<br>2<br>3 |
| Cape Verde | 23.5° E, 15.5° N | January<br>February<br>March<br>April<br>May<br>September<br>October<br>November<br>December | *'From the months of July to November, it is the best opportunity to spot a whale shark in the waters around Cape Verde'*<br>*'Over the course of the expedition [25th September – 1st October 2017] seven oceanic manta rays (Manta birostris) and two whale sharks (Rhincodon typus) were observed either directly or through BRUVs and associate tenders. We successfully in-water tagged the two mantas and one whale shark encountered with SPOT6 torpedo towed tags.'* | 4<br>5 |

**b)** Atlantic continued

| Sub-region | Location | Season | Occurrence details | Reference |
|---|---|---|---|---|
| Brazil (southeast) | 40.0° E, 26.0° S | May | *‘Presence of whale shark in southeastern Brazil is based on 38 known records (1983–2020). Rio de Janeiro state coast is a possible feeding site for the whale shark. Its presence is related to upwelling and warmer SST, mainly from February to May’*<br>*‘The R. typus individual was sighted during a fish survey on 12 May 2011 c. 60 km off the Brazilian central coast’*<br>*‘Footage released on Friday, May 1st, by the 'Baía Viva' Movement, shows a whale shark in the waters of Guanabara Bay surrounding Rio de Janeiro.’…‘Experts comment that the animal was being lured by the sardines in the region.’…’According to experts, the smaller number of boats in Guanabara Bay has allowed the presence of animals that would not otherwise be found in the region.’* | 6<br>7<br>8 |
| Angola | 11.0° E, 11.5° S | January<br>February<br>March<br>April<br>May<br>June<br>December | *‘Deep sea graveyard near Angola’*<br>*‘Temporal coverage of 31 years for the Atlantic Ocean (1980–2010) from 21°N to15°S and 34°W to14°E’*<br>*‘Eight of the records were from oceanic waters exceeding 1000 m, while single records originated from shelf-edge and continental shelf waters. The records support a year-round occurrence of whale sharks in offshore West African waters, although the majority (N= 8) of sightings occurred during the spring and summer months between September and January.’* | 9<br>10<br>11 |

**c)** Indian Ocean

| Sub-region | Location | Season | Occurrence details | Reference |
|---|---|---|---|---|
| Gujarat | 68.5° E, 21.0° N | March<br>April<br>May<br>June<br>July<br>August | *'There appears to be a seasonal migration along the coastal waters, from the south towards the north along the west coast and from the north towards the south along the east coast of India. The whale shark fishery starts from December in the west coast and reaches peak during March – June with maximum aggregation in Gujarat. The aggregation during the pre-monsoon seasons along the west coast (April-June) suggests that the whale sharks migrate away from the Indian coasts during the monsoon.'*<br>*'In the 1990s, a targeted whale shark fishery existed off the Gujarat coast following increased demand for the flesh in some other Asian countries. Since the ban, landings of whale sharks have decreased substantially with only 79 recorded between 2001 and 2011. Landings were recorded in each year and in each month of the year with the highest landings in January and February.'*<br>*'Six of the eight individuals remained close to their tagging locations, although two sharks displayed wide ranging movements into the Arabian Sea, following frontal zones between water masses of different sea surface temperatures.'* | 12<br>13<br>14 |

**d)** Indian Ocean continued

| Sub-region | Location | Season | Occurrence details | Reference |
|---|---|---|---|---|
| Maldives | 72.5° E, 1.0° S | March<br>April<br>May<br>June | *'In the Maldives, whale sharks, Rhincodon typus, are thought to have a semi-annual residency pattern, moving west from December to April and east from May to November.'*<br>*'The northeast monsoon, from December to April, brings the "dry season" with limited rainfalls, whilst the southwest monsoon "wet season" occurs from May to November bringing increased rainfalls. The findings from the present study highlighted the presence of whale shark especially during the dry season (northeast monsoon) but data did not show a seasonal peak in the first half of the year. Along the entire Maldives archipelago, whale sharks show a pattern of distribution strongly related to the monsoon seasons.'*<br>*'We made surveys for whale sharks Rhincodon typus on a total of 99 d from April through June each year from 2006 to 2008 along the southern fringe of the South Ari Atoll, Maldives Archipelago.'… 'We recorded the length and sex of each shark observed and made photographs to facilitate repeated identification from their spot patterns using pattern-recognition software. We identified 64 whale sharks from digital photographs taken during 220 sightings over 3 yr.'* | 15<br>16<br>17 |
| Mauritius | 59.5° E, 18.0° S | September<br>October | *'There are few other reports of very young whale sharks: one was a 61 cm TL specimen found alive in the stomach of a blue marlin, Makaira mazara, off Mauritius in 1993 (D. Goorah, personal communication and cited in Colman1997).'*<br>Multimedia: Whale Shark encounter in Mauritius while Big game fishing with Ivan Charoux & Vacances Adventures<br>*'In terms of their spatial distribution, whale sharks occur in the following Indian Ocean states: Australia (Western Australia), Bangladesh, Djibouti, India, Indonesia, Kenya, Madagascar, Malaysia, the Maldives,* ***Mauritius****, Mozambique, Seychelles, Somalia, South Africa, Sri Lanka, Tanzania and Thailand.'* | 18<br>19<br>20 |

**e)** Indian Ocean continued

| Sub-region | Location | Season | Occurrence details | Reference |
|---|---|---|---|---|
| Bangladesh | 90.0° E, 22.0° N | January<br>August<br>September<br>October<br>November<br>December | *'The Whale-Shark in these waters is next heard of at the mouth of the Hooghly River, recorded by Lloyd (1908). This was a small specimen (14 feet long). The description of its colour and of its teeth definitely justify the diagnosis of it as a Whale-Shark.'*<br>*'In terms of their spatial distribution, whale sharks occur in the following Indian Ocean states: Australia (Western Australia),* ***Bangladesh****, Djibouti, India, Indonesia, Kenya, Madagascar, Malaysia, the Maldives, Mauritius, Mozambique, Seychelles, Somalia, South Africa, Sri Lanka, Tanzania and Thailand.'*<br>News: Giant whale shark caught in Chattogram<br>*'Deep-sea, pelagic and migratory elasmobranchs are quite unlikely to be caught in abundance in the shallow depths predominantly fished by Bangladeshi artisanal fisheries. For example, whale sharks and thresher sharks were poorly reported, though there were anecdotal whale shark reports in industrial fisheries (news articles, pers. comm. 2019).'*<br>*'Recently a new member has been found within this territory is Whale Shark by fishermen while acting under their fish catching commercial activities. They caught it, killed it, sold it for 5000 takas and made 'Dry-fish' from the flesh of that 25 feet long and around 400 kg weighted Whale Shark fish, according to the news report of Channel Ekattor TV broadcast on February 2018.'* | 21<br>20<br>22<br>23<br>24 |

**f)** Pacific

| Sub-region | Location | Season | Occurrence details | Reference |
|---|---|---|---|---|
| Palmyra | 160.0°W, 7.5° N | January<br>February<br>March<br>April<br>May<br>June | *'Closest I got was hearing over the radio about a Whale Shark sighting made by NOAA divers in 2005'*<br>*'There have been 2 guaranteed Whale Shark sightings at Palmyra since I have been there. I swam with one about 2 miles off the north shore of the atoll in August 2008 and then some scientific researchers swam with one off the SE side of the atoll while on a scuba safety stop in roughly 2010-2011. I have not heard of or seen any other sightings since then.'* | Personal communications: **Amanda Lei Perron** |
| Taiwan | 122.0° E, 22.0° N | May<br>June<br>July<br>August<br>September<br>October | *'Of the 113 Whale Sharks caught around Taiwan, most (93 fish, 82%) were caught off Taiwan's eastern coast in the Pacific Ocean. The remainder were harvested in the Taiwan Strait (13 fish, 12%) and Penghu Archipelago (seven fish, 6%) (see Figure 2). Forty-four per cent of the Whale Sharks were caught by set net, 36% by harpoon and 20% by other methods such as trawl. The data identify May (22%) and November (18%) as the peak catch months.'*<br>*'Another shark tagged in November moved above the sea ridges in the first month after being released then migrated along the eastern and northern coastal waters of Taiwan during winter.'* | 25<br>26 |

**g)** Pacific continued

| Sub-region | Location | Season | Occurrence details | Reference |
|---|---|---|---|---|
| Vanuatu | 166.0° E, 16.0° S | January<br>February<br>March<br>April<br>May<br>June | *'Shark and ray bycatch has rarely been significant in the Vanuatu-flagged purse seine fishery – the biggest issue has been several catches of whale sharks. The Vanuatu-flagged longline fishery active in the WCPFC Convention area carried no observers until 2012 (data not yet available) – no estimates can be determined.'*<br>Multimedia: *'Here is a short video clip, filmed by Liam Donnelly of Santo Island Dive, of a 5 mt juvenile female whale shark swimming with Matthew and guests of Santo Island Dive on the SS President Coolidge on Sunday 29 September 2013. The shark spent about 30 minutes with the divers around the boat.'* | 27<br>28 |
| Fiji | 177.0° E, 17.5° S | January<br>February<br>March<br>April<br>May<br>December | Multimedia: Whale Shark in Kadavu, Fiji<br>Social media: Whale shark at Volivoli Beach Resort, Fiji | 29<br>30 |

**Table S12 |** Habitat suitability weighted centroid and geographical mean calculated for each percentile (50th, 75th, 90th, 95th) for the current distribution and each projected future decade and scenario combination from 0.25 ×0.25° resolution cells within each region. Here core habitats are defined as:

**a)** The top Xth of the relative distribution.

| Weighted centroid | | | | | | | | |
|---|---|---|---|---|---|---|---|---|
| *Habitats compared to quantiles within each respective map* | | Current | Future | | | | | |
| | | 2005-2019 | 2046-2055 | | | 2086-2095 | | |
| | | | ssp126 | ssp370 | ssp585 | ssp126 | ssp370 | ssp585 |
| North Atlantic | Lon | 12.057 | 10.515 | 10.283 | 10.009 | 9.765 | 7.372 | 5.985 |
| | Lat | -59.071 | -52.865 | -51.827 | -50.687 | -51.455 | -41.142 | -37.109 |
| South Atlantic | Lon | -2.959 | 0.362 | 1.875 | 3.442 | -2.175 | 1.213 | -0.846 |
| | Lat | -29.883 | -29.127 | -29.260 | -30.688 | -26.988 | -24.746 | -23.158 |
| Northwest Indian Ocean | Lon | 21.295 | 20.989 | 20.826 | 20.732 | 21.025 | 18.666 | 16.794 |
| | Lat | 48.674 | 49.253 | 49.156 | 49.683 | 49.274 | 53.303 | 57.099 |
| Southwest Indian Ocean | Lon | -12.412 | -10.734 | -10.360 | -9.872 | -10.712 | -7.038 | -5.564 |
| | Lat | 67.779 | 67.926 | 67.431 | 67.468 | 67.460 | 66.435 | 66.287 |
| East Indian Ocean | Lon | -13.879 | -14.035 | -14.311 | -14.786 | -14.978 | -15.825 | -18.510 |
| | Lat | 98.740 | 99.756 | 99.746 | 99.652 | 97.633 | 101.246 | 103.786 |
| West Pacific | Lon | 1.560 | 1.867 | 1.976 | 1.898 | 1.621 | 2.368 | 2.632 |
| | Lat | 134.563 | 136.209 | 134.982 | 136.268 | 138.356 | 134.556 | 136.216 |
| East Pacific | Lon | 5.771 | 5.734 | 5.867 | 5.889 | 5.628 | 6.335 | 11.829 |
| | Lat | -98.835 | -98.126 | -97.724 | -97.556 | -98.618 | -97.501 | -104.081 |
| 50th | | | | | | | | |
| *Habitats compared to quantiles within each respective map* | | Current | Future | | | | | |
| | | 2005-2019 | 2046-2055 | | | 2086-2095 | | |
| | | | ssp126 | ssp370 | ssp585 | ssp126 | ssp370 | ssp585 |
| North Atlantic | Lon | -35.416 | -34.462 | -34.089 | -34.039 | -34.045 | -32.414 | -30.963 |
| | Lat | 5.170 | 5.303 | 4.956 | 5.060 | 4.266 | 2.848 | 1.296 |
| South Atlantic | Lon | -33.861 | -33.256 | -33.297 | -33.509 | -33.366 | -32.348 | -30.774 |
| | Lat | 4.765 | 5.076 | 5.699 | 5.788 | 5.193 | 5.621 | 4.340 |
| Northwest Indian Ocean | Lon | 71.996 | 72.046 | 72.036 | 72.007 | 72.087 | 72.015 | 71.886 |
| | Lat | -5.209 | -5.385 | -5.429 | -5.447 | -5.399 | -5.336 | -5.286 |
| Southwest Indian Ocean | Lon | 72.130 | 71.206 | 70.831 | 70.773 | 70.725 | 70.537 | 70.864 |
| | Lat | -9.265 | -8.596 | -8.577 | -8.301 | -8.553 | -7.512 | -6.756 |
| East Indian Ocean | Lon | 76.783 | 76.320 | 76.897 | 76.637 | 75.925 | 77.605 | 78.271 |
| | Lat | -12.641 | -12.643 | -13.886 | -14.293 | -13.059 | -17.730 | -19.258 |
| West Pacific | Lon | 142.808 | 142.429 | 142.134 | 142.267 | 142.754 | 142.371 | 142.623 |
| | Lat | 2.307 | 2.975 | 2.838 | 2.931 | 2.625 | 2.669 | 2.600 |
| East Pacific | Lon | -125.895 | -123.796 | -122.421 | -122.935 | -124.549 | -120.489 | -124.558 |
| | Lat | 1.429 | 1.140 | 0.429 | 1.357 | 1.907 | 2.111 | 1.501 |

**b)** The top X$^{th}$ of the relative distribution continued.

| 75th | | | | | | | | |
|---|---|---|---|---|---|---|---|---|
| *Habitats compared to quantiles within each respective map* | | Current | Future | | | | | |
| | | 2005-2019 | 2046-2055 | | | 2086-2095 | | |
| | | | ssp126 | ssp370 | ssp585 | ssp126 | ssp370 | ssp585 |
| North Atlantic | Lon | -45.117 | -43.709 | -43.610 | -43.247 | -43.721 | -38.592 | -36.006 |
| | Lat | 8.407 | 8.417 | 8.610 | 8.479 | 7.863 | 7.273 | 6.240 |
| South Atlantic | Lon | -35.333 | -34.087 | -33.600 | -33.808 | -33.691 | -30.316 | -28.847 |
| | Lat | 2.991 | 4.087 | 4.668 | 4.981 | 2.918 | 3.527 | 2.231 |
| Northwest Indian Ocean | Lon | 73.321 | 73.527 | 73.547 | 73.619 | 73.637 | 73.823 | 73.856 |
| | Lat | -2.406 | -2.508 | -2.649 | -2.560 | -2.508 | -2.485 | -2.316 |
| Southwest Indian Ocean | Lon | 65.907 | 65.247 | 65.145 | 65.083 | 64.773 | 65.158 | 65.540 |
| | Lat | -11.475 | -10.687 | -10.352 | -10.127 | -10.747 | -7.211 | -5.393 |
| East Indian Ocean | Lon | 82.920 | 82.433 | 82.450 | 82.111 | 81.478 | 87.277 | 89.369 |
| | Lat | -15.496 | -15.937 | -16.270 | -16.597 | -16.698 | -20.342 | -22.000 |
| West Pacific | Lon | 133.721 | 135.089 | 134.088 | 135.319 | 137.251 | 134.062 | 135.520 |
| | Lat | 1.895 | 2.004 | 2.209 | 1.991 | 1.601 | 2.587 | 2.837 |
| East Pacific | Lon | -113.116 | -112.202 | -111.027 | -111.605 | -113.259 | -108.851 | -115.903 |
| | Lat | 3.273 | 3.718 | 3.152 | 3.504 | 4.408 | 1.094 | 3.305 |
| 90th | | | | | | | | |
| *Habitats compared to quantiles within each respective map* | | Current | Future | | | | | |
| | | 2005-2019 | 2046-2055 | | | 2086-2095 | | |
| | | | ssp126 | ssp370 | ssp585 | ssp126 | ssp370 | ssp585 |
| North Atlantic | Lon | -64.616 | -61.205 | -61.296 | -59.224 | -59.818 | -49.535 | -42.542 |
| | Lat | 13.898 | 12.500 | 12.504 | 11.927 | 12.020 | 9.018 | 7.337 |
| South Atlantic | Lon | -30.209 | -29.694 | -29.971 | -30.958 | -27.696 | -25.567 | -22.380 |
| | Lat | -2.648 | 0.900 | 2.504 | 3.241 | -1.480 | 1.564 | -1.025 |
| Northwest Indian Ocean | Lon | 73.183 | 74.356 | 74.615 | 74.838 | 74.905 | 75.758 | 75.844 |
| | Lat | 3.814 | 2.988 | 2.798 | 2.856 | 2.812 | 2.766 | 3.115 |
| Southwest Indian Ocean | Lon | 65.965 | 67.351 | 67.286 | 67.507 | 66.610 | 67.221 | 66.686 |
| | Lat | -12.837 | -10.881 | -10.285 | -9.786 | -11.056 | -6.915 | -5.687 |
| East Indian Ocean | Lon | 103.784 | 103.698 | 103.139 | 102.480 | 102.289 | 99.787 | 101.468 |
| | Lat | -13.291 | -13.531 | -13.889 | -14.544 | -14.460 | -16.515 | -20.160 |
| West Pacific | Lon | 131.098 | 131.568 | 130.955 | 131.958 | 133.614 | 131.061 | 132.111 |
| | Lat | -0.780 | -0.392 | -0.487 | -0.458 | -0.708 | 0.401 | 1.228 |
| East Pacific | Lon | -98.159 | -98.067 | -98.386 | -98.626 | -98.135 | -99.790 | -107.985 |
| | Lat | 6.263 | 6.175 | 6.715 | 6.772 | 6.163 | 6.025 | 8.028 |

**c)** The top X$^{th}$ of the relative distribution continued.

| 95$^{th}$ | | | | | | | | |
|---|---|---|---|---|---|---|---|---|
| *Habitats compared to quantiles within each respective map* | | **Current** | **Future** | | | | | |
| | | **2005-2019** | **2046-2055** | | | **2086-2095** | | |
| | | | **ssp126** | **ssp370** | **ssp585** | **ssp126** | **ssp370** | **ssp585** |
| North Atlantic | Lon | -75.374 | -71.506 | -71.501 | -69.079 | -69.179 | -56.117 | -45.435 |
| | Lat | 16.382 | 14.887 | 14.729 | 13.872 | 13.854 | 10.227 | 8.215 |
| South Atlantic | Lon | -25.916 | -25.535 | -27.219 | -29.791 | -22.462 | -24.518 | -23.083 |
| | Lat | -6.947 | -2.299 | 0.807 | 3.510 | -5.987 | 1.020 | -2.296 |
| Northwest Indian Ocean | Lon | 63.278 | 64.907 | 65.148 | 65.559 | 65.273 | 66.950 | 67.085 |
| | Lat | 11.029 | 10.443 | 10.232 | 10.382 | 10.499 | 10.550 | 11.082 |
| Southwest Indian Ocean | Lon | 71.602 | 72.008 | 71.096 | 71.495 | 71.579 | 70.016 | 68.324 |
| | Lat | -12.697 | -10.521 | -10.230 | -9.303 | -10.014 | -6.824 | -5.036 |
| East Indian Ocean | Lon | 107.428 | 107.127 | 106.638 | 106.803 | 106.851 | 106.729 | 108.463 |
| | Lat | -13.340 | -13.555 | -13.453 | -13.956 | -14.145 | -13.909 | -17.027 |
| West Pacific | Lon | 130.787 | 132.867 | 132.468 | 133.385 | 134.499 | 133.705 | 134.578 |
| | Lat | -1.337 | -1.470 | -1.569 | -1.693 | -1.826 | -1.930 | -1.131 |
| East Pacific | Lon | -93.468 | -93.954 | -93.581 | -93.422 | -93.740 | -97.341 | -105.426 |
| | Lat | 5.299 | 5.563 | 5.386 | 5.437 | 4.899 | 7.419 | 13.914 |

**d)** The top X$^{th}$ of the current distribution (2005 – 2019).

| 50$^{th}$ | | | | | | | | |
|---|---|---|---|---|---|---|---|---|
| *Habitats compared to quantiles within 2005-2019 baseline* | | **Current** | **Future** | | | | | |
| | | **2005-2019** | **2046-2055** | | | **2086-2095** | | |
| | | | **ssp126** | **ssp370** | **ssp585** | **ssp126** | **ssp370** | **ssp585** |
| North Atlantic | Lon | -35.416 | -34.353 | -33.729 | -34.016 | -33.774 | -32.257 | -31.115 |
| | Lat | 5.170 | 5.257 | 4.759 | 5.044 | 4.149 | 2.728 | 1.405 |
| South Atlantic | Lon | -33.861 | -33.259 | -33.378 | -33.602 | -33.315 | -32.679 | -31.228 |
| | Lat | 4.765 | 5.074 | 5.660 | 5.768 | 5.230 | 5.690 | 4.504 |
| Northwest Indian Ocean | Lon | 71.996 | 72.073 | 72.096 | 72.096 | 72.116 | 72.293 | 72.410 |
| | Lat | -5.209 | -5.455 | -5.574 | -5.621 | -5.447 | -5.921 | -6.217 |
| Southwest Indian Ocean | Lon | 72.130 | 71.898 | 71.763 | 71.777 | 71.476 | 71.948 | 71.705 |
| | Lat | -9.265 | -8.485 | -8.539 | -8.306 | -8.438 | -7.964 | -7.755 |
| East Indian Ocean | Lon | 76.783 | 76.132 | 76.872 | 76.586 | 75.726 | 78.506 | 80.407 |
| | Lat | -12.641 | -12.549 | -13.862 | -14.218 | -12.794 | -18.298 | -20.529 |
| West Pacific | Lon | 142.808 | 143.192 | 143.068 | 143.376 | 143.502 | 143.025 | 143.216 |
| | Lat | 2.307 | 3.446 | 3.265 | 3.361 | 3.214 | 2.724 | 2.862 |
| East Pacific | Lon | -125.895 | -124.943 | -123.738 | -124.609 | -125.922 | -121.339 | -122.212 |
| | Lat | 1.429 | 1.021 | 0.511 | 1.380 | 1.851 | 2.081 | 2.935 |
| 75$^{th}$ | | | | | | | | |
| *Habitats compared to quantiles within 2005-2019 baseline* | | **Current** | **Future** | | | | | |
| | | **2005-2019** | **2046-2055** | | | **2086-2095** | | |
| | | | **ssp126** | **ssp370** | **ssp585** | **ssp126** | **ssp370** | **ssp585** |
| North Atlantic | Lon | -45.117 | -40.485 | -39.837 | -39.690 | -40.004 | -35.514 | -33.741 |
| | Lat | 8.407 | 7.774 | 7.510 | 7.576 | 6.677 | 5.428 | 4.048 |
| South Atlantic | Lon | -35.333 | -34.097 | -33.472 | -33.619 | -33.707 | -28.353 | -23.654 |
| | Lat | 2.991 | 4.106 | 4.453 | 4.684 | 2.978 | 2.399 | -0.299 |
| Northwest Indian Ocean | Lon | 73.321 | 73.450 | 73.376 | 73.392 | 73.569 | 73.094 | 72.802 |
| | Lat | -2.406 | -2.580 | -2.704 | -2.647 | -2.527 | -2.852 | -2.975 |
| Southwest Indian Ocean | Lon | 65.907 | 65.588 | 65.521 | 65.580 | 65.152 | 67.262 | 68.731 |
| | Lat | -11.475 | -10.435 | -10.189 | -9.816 | -10.448 | -7.022 | -6.030 |
| East Indian Ocean | Lon | 82.920 | 84.580 | 85.261 | 85.487 | 81.813 | 92.298 | 95.894 |
| | Lat | -15.496 | -16.085 | -16.428 | -16.656 | -16.726 | -19.264 | -21.399 |
| West Pacific | Lon | 133.721 | 135.722 | 134.037 | 135.393 | 138.056 | 133.870 | 135.935 |
| | Lat | 1.895 | 1.982 | 2.201 | 1.995 | 1.706 | 2.581 | 2.868 |
| East Pacific | Lon | -113.116 | -112.034 | -110.217 | -111.151 | -113.759 | -106.017 | -111.909 |
| | Lat | 3.273 | 3.760 | 3.366 | 3.651 | 4.320 | 1.351 | 3.742 |

**e)** The top X$^{th}$ of the current distribution (2005 – 2019) continued.

| **90$^{th}$** | | | | | | | | |
|---|---|---|---|---|---|---|---|---|
| *Habitats compared to quantiles within 2005-2019 baseline* | | **Current** | **Future** | | | | | |
| | | **2005-2019** | **2046-2055** | | | **2086-2095** | | |
| | | | **ssp126** | **ssp370** | **ssp585** | **ssp126** | **ssp370** | **ssp585** |
| North Atlantic | Lon | -64.616 | -53.476 | -51.595 | -50.347 | -51.651 | -40.101 | -36.597 |
| | Lat | 13.898 | 10.715 | 10.194 | 9.970 | 9.961 | 7.790 | 6.541 |
| South Atlantic | Lon | -30.209 | -29.232 | -28.735 | -29.714 | -27.473 | -24.621 | -23.487 |
| | Lat | -2.648 | 0.563 | 2.063 | 3.412 | -1.663 | 0.993 | -2.136 |
| Northwest Indian Ocean | Lon | 73.183 | 75.154 | 75.563 | 75.947 | 75.699 | 77.331 | 77.527 |
| | Lat | 3.814 | 2.543 | 2.097 | 2.066 | 2.407 | 0.802 | 0.099 |
| Southwest Indian Ocean | Lon | 65.965 | 66.371 | 65.907 | 65.842 | 65.965 | 65.546 | 65.541 |
| | Lat | -12.837 | -11.088 | -10.615 | -10.201 | -11.183 | -6.916 | -5.392 |
| East Indian Ocean | Lon | 103.784 | 104.329 | 104.376 | 104.472 | 103.843 | 105.956 | 108.948 |
| | Lat | -13.291 | -13.424 | -13.722 | -14.194 | -14.163 | -14.163 | -16.712 |
| West Pacific | Lon | 131.098 | 131.585 | 130.849 | 132.027 | 133.705 | 130.783 | 131.971 |
| | Lat | -0.780 | -0.171 | -0.306 | -0.304 | -0.511 | 0.857 | 1.559 |
| East Pacific | Lon | -98.159 | -96.971 | -96.445 | -96.009 | -97.430 | -95.682 | -103.250 |
| | Lat | 6.263 | 6.110 | 6.223 | 6.037 | 6.049 | 7.312 | 13.869 |
| **95$^{th}$** | | | | | | | | |
| *Habitats compared to quantiles within 2005-2019 baseline* | | **Current** | **Future** | | | | | |
| | | **2005-2019** | **2046-2055** | | | **2086-2095** | | |
| | | | **ssp126** | **ssp370** | **ssp585** | **ssp126** | **ssp370** | **ssp585** |
| North Atlantic | Lon | -75.374 | -64.934 | -62.141 | -59.489 | -61.429 | -44.272 | -38.336 |
| | Lat | 16.382 | 13.488 | 12.751 | 12.012 | 12.488 | 7.882 | 6.280 |
| South Atlantic | Lon | -25.916 | -24.996 | -27.427 | -30.521 | -22.110 | -23.642 | -22.772 |
| | Lat | -6.947 | -2.924 | -0.464 | 3.000 | -6.433 | 0.352 | 1.784 |
| Northwest Indian Ocean | Lon | 63.278 | 66.885 | 67.910 | 68.866 | 67.191 | 72.955 | 74.665 |
| | Lat | 11.029 | 8.644 | 7.902 | 7.601 | 8.877 | 5.012 | 3.849 |
| Southwest Indian Ocean | Lon | 71.602 | 70.529 | 69.271 | 68.991 | 70.252 | 65.644 | 65.220 |
| | Lat | -12.697 | -10.491 | -9.975 | -9.256 | -10.268 | -7.081 | -5.478 |
| East Indian Ocean | Lon | 107.428 | 107.951 | 108.015 | 108.610 | 107.653 | 111.463 | 113.859 |
| | Lat | -13.340 | -13.508 | -13.547 | -13.986 | -14.111 | -13.364 | -14.201 |
| West Pacific | Lon | 130.787 | 132.923 | 132.516 | 133.396 | 134.586 | 133.732 | 134.935 |
| | Lat | -1.337 | -1.510 | -1.601 | -1.727 | -1.830 | -1.919 | -1.584 |
| East Pacific | Lon | -93.468 | -92.672 | -91.204 | -90.702 | -93.009 | -90.459 | -95.446 |
| | Lat | 5.299 | 5.620 | 5.239 | 5.128 | 4.705 | 5.559 | 8.858 |

## 2. Supplementary Figures

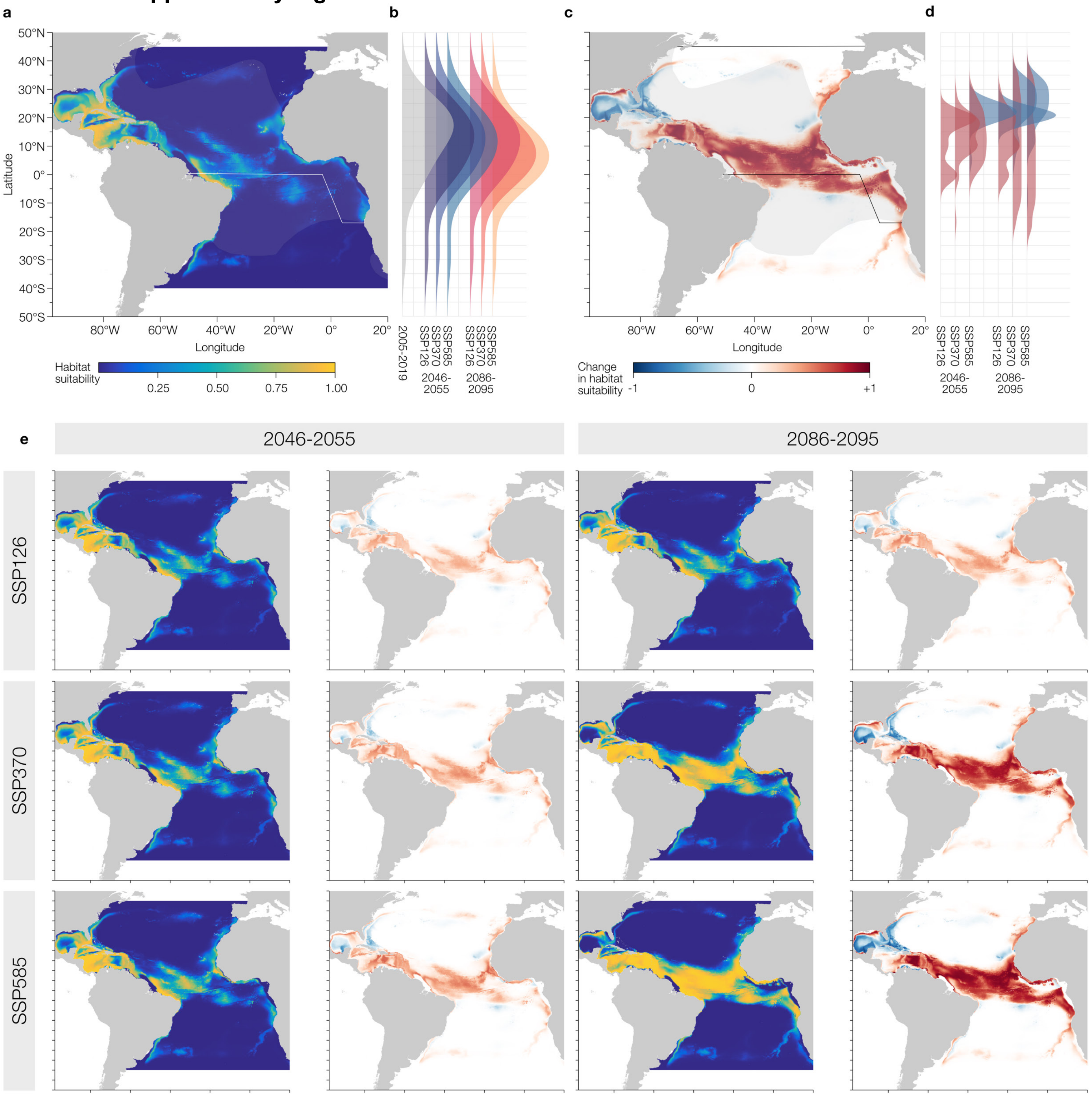


**Figure S1 | Current habitat suitability and change in habitat suitability for whale sharks under projected environmental conditions in the north Atlantic (NA) region. a**, Regions of high (yellow) and low (blue) habitat suitability are indicated for the NA based on current climatology's (2005 – 2019) and their, **b**, sum weighted latitudinal density distributions coloured by decade and scenario. **c**, Regions of increase (red), decrease (blue) and no change (white) are indicated for the NA based on 2086 – 2095 ssp585 climatology's and their, **d**, latitudinal density distributions for cells containing positive (>0.5, red) or negative (<-0.5, blue) values separated by decade and scenario. **e**, Regions of high (yellow) and low (blue) habitat suitability (columns 1 and 3) and regions of increase (red), decrease (blue) and no change (white) (columns 2 and 4) for each decade and scenario combination as indicated by the column and row headings. Note that predictions and projections beyond the white (**a**) and black (**b**) boundaries should be interpreted with caution due to potential extrapolation. The current IUCN distribution limits are displayed in **a** and **c**. Maps are outputs of Generalised Additive Models.

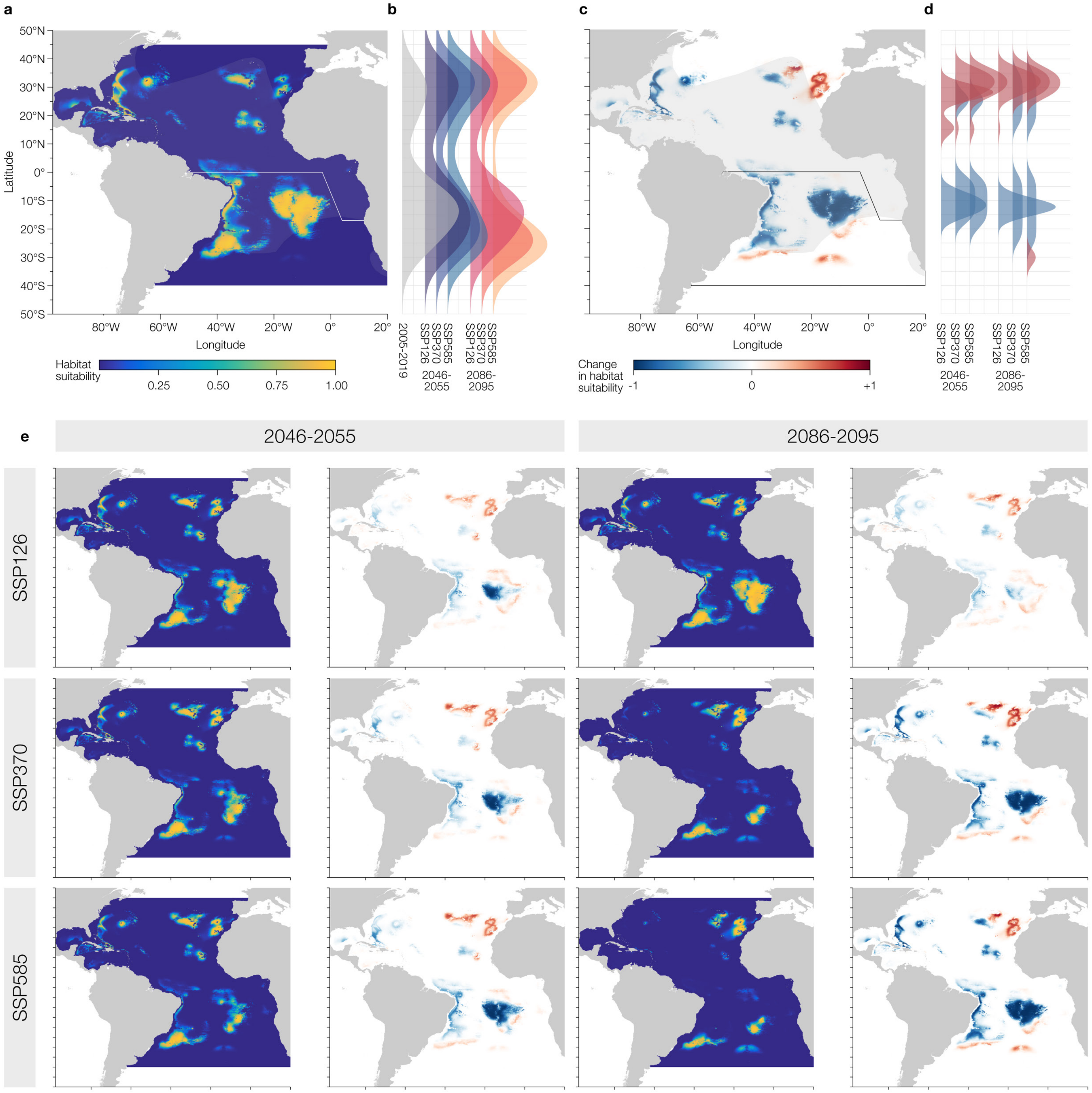


**Figure S2 | Current habitat suitability and change in habitat suitability for whale sharks under projected environmental conditions in the south Atlantic (SA) region. a**, Regions of high (yellow) and low (blue) habitat suitability are indicated for the SA based on current climatology's (2005 – 2019) and their, **b**, sum weighted latitudinal density distributions coloured by decade and scenario. **c**, Regions of increase (red), decrease (blue) and no change (white) are indicated for the SA based on 2086 – 2095 ssp585 climatology's and their, **d**, latitudinal density distributions for cells containing positive (>0.5, red) or negative (<-0.5, blue) values separated by decade and scenario. **e**, Regions of high (yellow) and low (blue) habitat suitability (columns 1 and 3) and regions of increase (red), decrease (blue) and no change (white) (columns 2 and 4) for each decade and scenario combination as indicated by the column and row headings. Note that predictions and projections beyond the white (**a**) and black (**b**) boundaries should be interpreted with caution due to potential extrapolation. The current IUCN distribution limits are displayed in **a** and **c**. Maps are outputs of Generalised Additive Models.

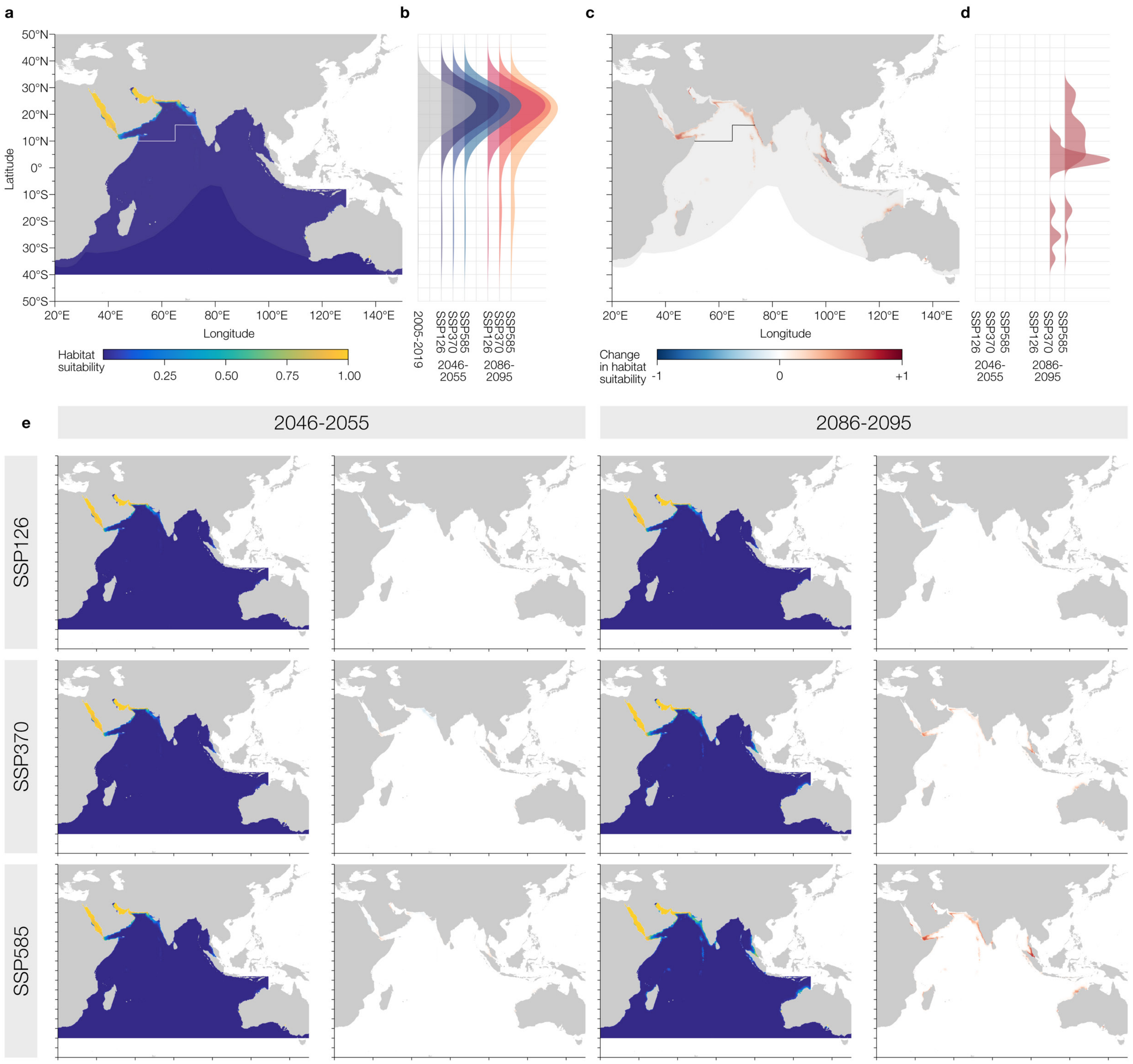


**Figure S3 | Current habitat suitability and change in habitat suitability for whale sharks under projected environmental conditions in the northwest Indian Ocean (NIO) region. a**, Regions of high (yellow) and low (blue) habitat suitability are indicated for the NIO based on current climatology's (2005 – 2019) and their, **b**, sum weighted latitudinal density distributions coloured by decade and scenario. **c**, Regions of increase (red), decrease (blue) and no change (white) are indicated for the NIO based on 2086 – 2095 ssp585 climatology's and their, **d**, latitudinal density distributions for cells containing positive (>0.5, red) or negative (<-0.5, blue) values separated by decade and scenario. **e**, Regions of high (yellow) and low (blue) habitat suitability (columns 1 and 3) and regions of increase (red), decrease (blue) and no change (white) (columns 2 and 4) for each decade and scenario combination as indicated by the column and row headings. Note that predictions and projections beyond the white (**a**) and black (**b**) boundaries should be interpreted with caution due to potential extrapolation. The current IUCN distribution limits are displayed in **a** and **c**. Maps are outputs of Generalised Additive Models.

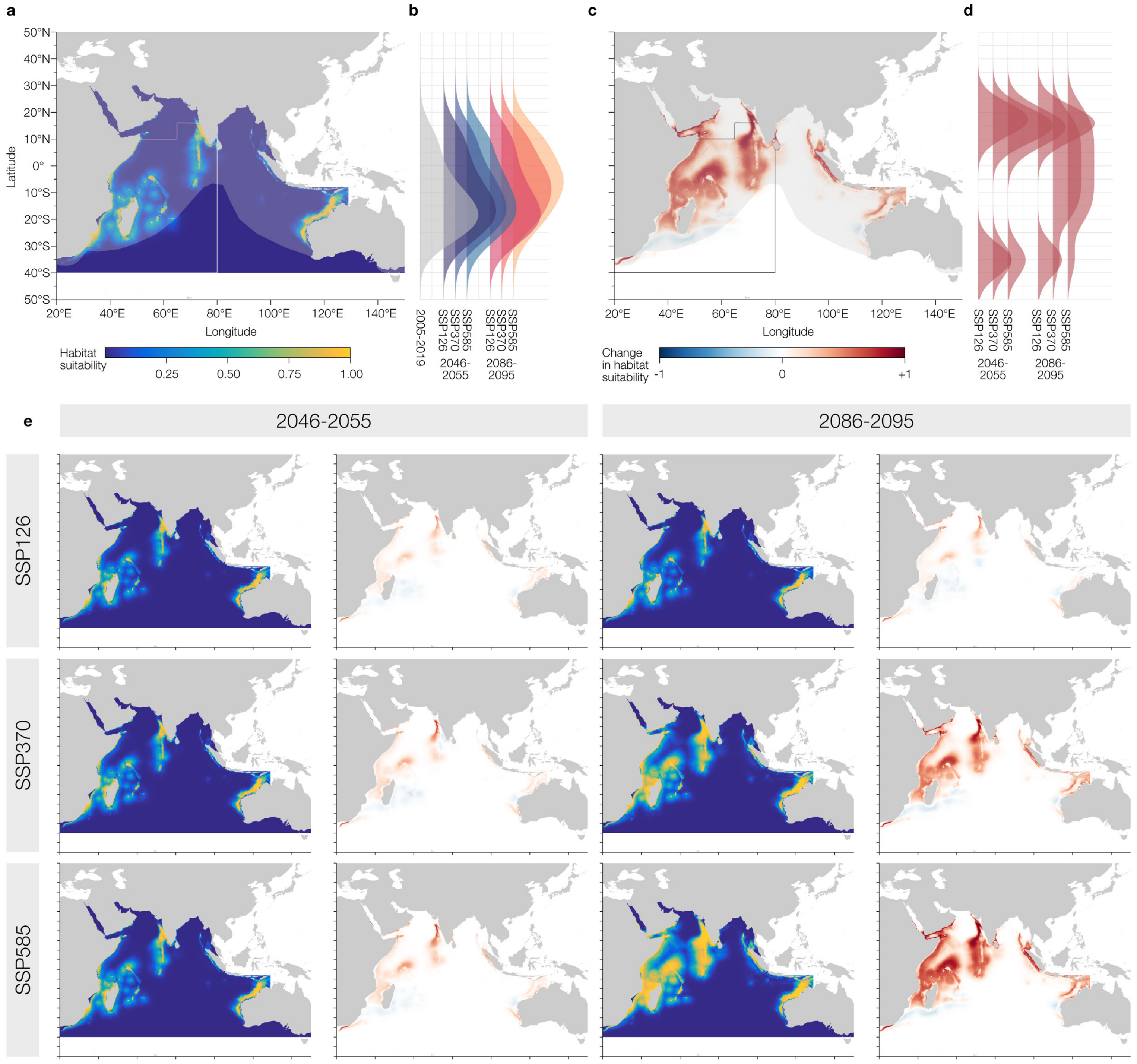


**Figure S4 | Current habitat suitability and change in habitat suitability for whale sharks under projected environmental conditions in the southwest Indian Ocean (SIO) region. a**, Regions of high (yellow) and low (blue) habitat suitability are indicated for the SIO based on current climatology's (2005 – 2019) and their, **b**, sum weighted latitudinal density distributions coloured by decade and scenario. **c**, Regions of increase (red), decrease (blue) and no change (white) are indicated for the SIO based on 2086 – 2095 ssp585 climatology's and their, **d**, latitudinal density distributions for cells containing positive (>0.5, red) or negative (<-0.5, blue) values separated by decade and scenario. **e**, Regions of high (yellow) and low (blue) habitat suitability (columns 1 and 3) and regions of increase (red), decrease (blue) and no change (white) (columns 2 and 4) for each decade and scenario combination as indicated by the column and row headings. Note that predictions and projections beyond the white (**a**) and black (**b**) boundaries should be interpreted with caution due to potential extrapolation. The current IUCN distribution limits are displayed in **a** and **c**. Maps are outputs of Generalised Additive Models.

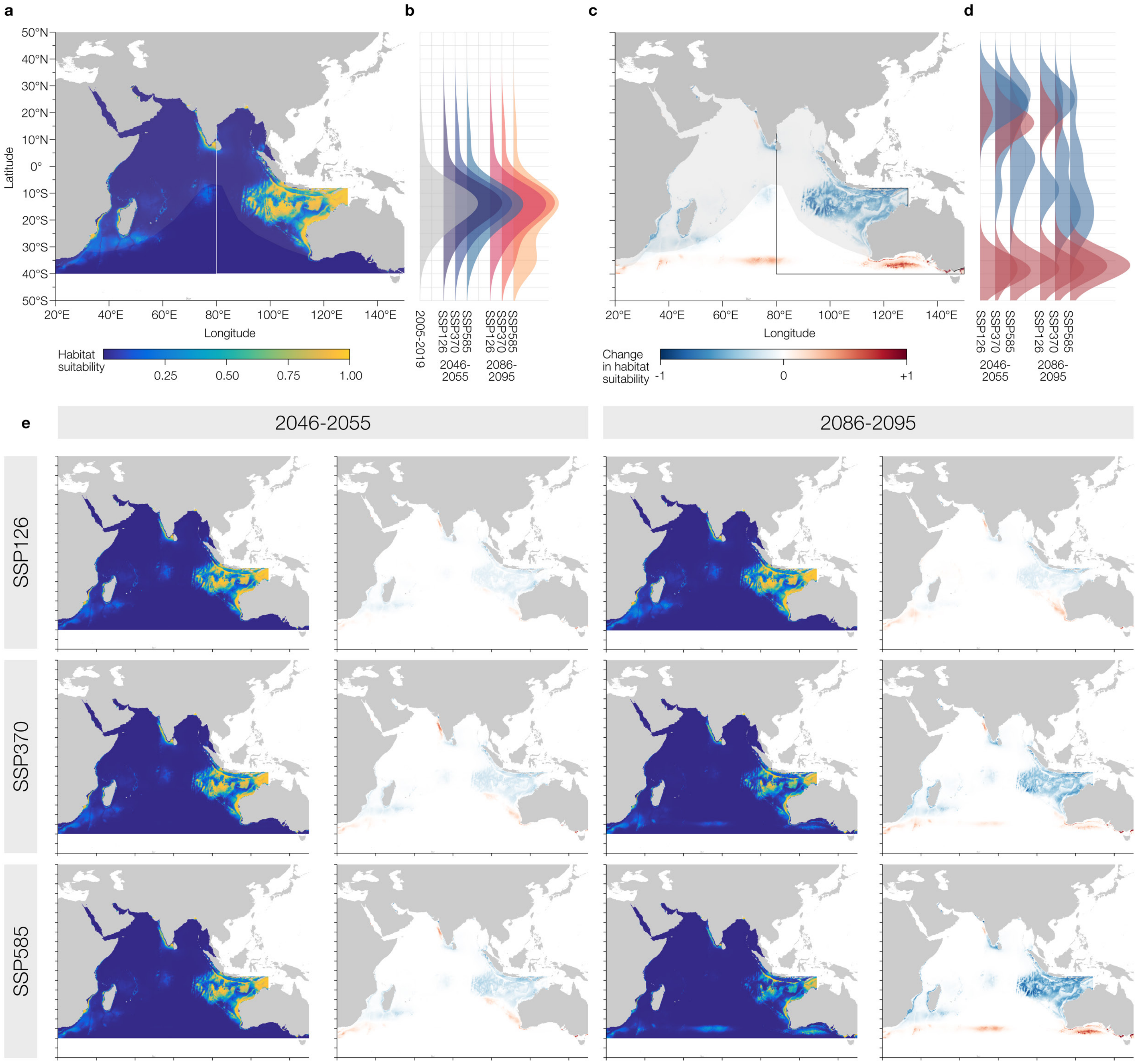


**Figure S5 | Current habitat suitability and change in habitat suitability for whale sharks under projected environmental conditions in the east Indian Ocean (EIO) region. a**, Regions of high (yellow) and low (blue) habitat suitability are indicated for the EIO based on current climatology's (2005 – 2019) and their, **b**, sum weighted latitudinal density distributions coloured by decade and scenario. **c**, Regions of increase (red), decrease (blue) and no change (white) are indicated for the EIO based on 2086 – 2095 ssp585 climatology's and their, **d**, latitudinal density distributions for cells containing positive (>0.5, red) or negative (<-0.5, blue) values separated by decade and scenario. **e**, Regions of high (yellow) and low (blue) habitat suitability (columns 1 and 3) and regions of increase (red), decrease (blue) and no change (white) (columns 2 and 4) for each decade and scenario combination as indicated by the column and row headings. Note that predictions and projections beyond the white (**a**) and black (**b**) boundaries should be interpreted with caution due to potential extrapolation. The current IUCN distribution limits are displayed in **a** and **c**. Maps are outputs of Generalised Additive Models.

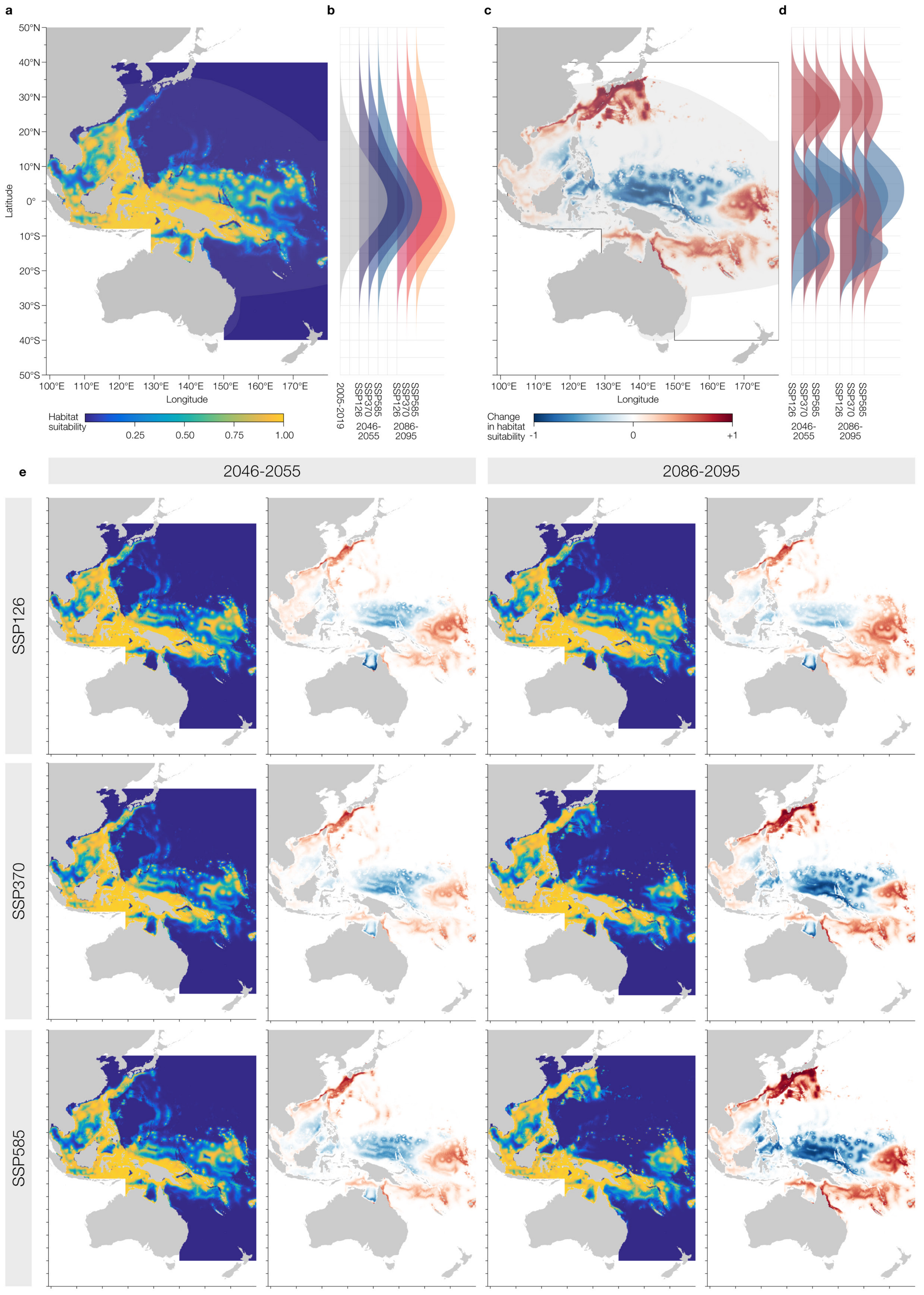

a
b
c
d
50°N
40°N
30°N
20°N
10°N
0°
10°S
20°S
30°S
40°S
50°S
Latitude
100°E
110°E
120°E
130°E
140°E
150°E
160°E
170°E
Longitude
Habitat suitability
0.25
0.50
0.75
1.00
2005-2019
SSP126
SSP370
SSP585
2046-2055
2086-2095
Change in habitat suitability
-1
0
+1
e
2046-2055
2086-2095
SSP126
SSP370
SSP585

**Figure S6 | Current habitat suitability and change in habitat suitability for whale sharks under projected environmental conditions in the west Pacific (WP) region. a**, Regions of high (yellow) and low (blue) habitat suitability are indicated for the WP based on current climatology's (2005 – 2019) and their, **b**, sum weighted latitudinal density distributions coloured by decade and scenario. **c**, Regions of increase (red), decrease (blue) and no change (white) are indicated for the WP based on 2086 – 2095 ssp585 climatology's and their, **d**, latitudinal density distributions for cells containing positive (>0.5, red) or negative (<-0.5, blue) values separated by decade and scenario. **e**, Regions of high (yellow) and low (blue) habitat suitability (columns 1 and 3) and regions of increase (red), decrease (blue) and no change (white) (columns 2 and 4) for each decade and scenario combination as indicated by the column and row headings. Note that predictions and projections beyond the white (**a**) and black (**b**) boundaries should be interpreted with caution due to potential extrapolation. The current IUCN distribution limits are displayed in **a** and **c**. Maps are outputs of Generalised Additive Models.

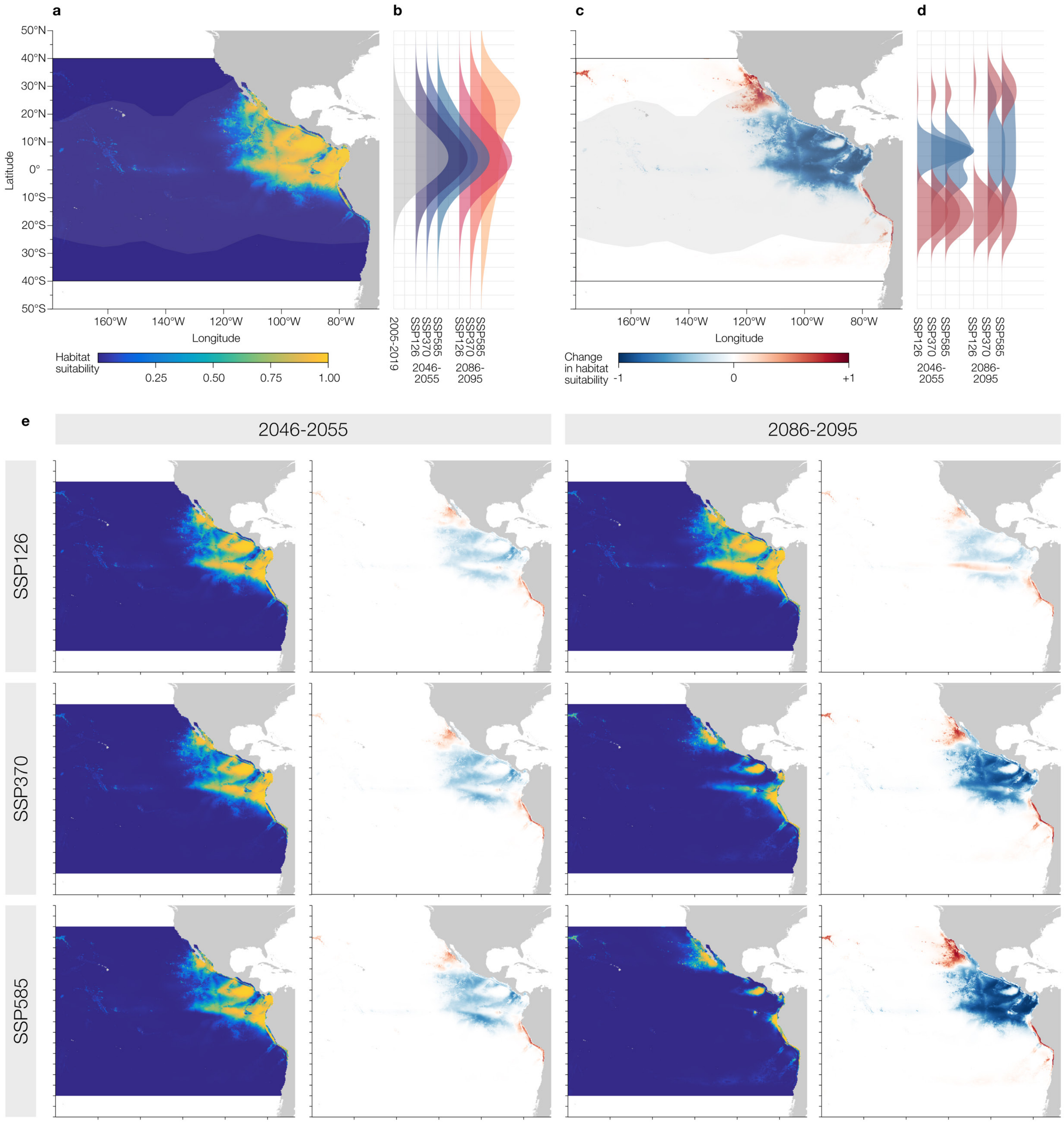


**Figure S7 | Current habitat suitability and change in habitat suitability for whale sharks under projected environmental conditions in the east Pacific (EP) region.** **a**, Regions of high (yellow) and low (blue) habitat suitability are indicated for the EP based on current climatology's (2005 – 2019) and their, **b**, sum weighted latitudinal density distributions coloured by decade and scenario. **c**, Regions of increase (red), decrease (blue) and no change (white) are indicated for the EP based on 2086 – 2095 ssp585 climatology's and their, **d**, latitudinal density distributions for cells containing positive (>0.5, red) or negative (<-0.5, blue) values separated by decade and scenario. **e**, Regions of high (yellow) and low (blue) habitat suitability (columns 1 and 3) and regions of increase (red), decrease (blue) and no change (white) (columns 2 and 4) for each decade and scenario combination as indicated by the column and row headings. Note that predictions and projections beyond the white (**a**) and black (**b**) boundaries should be interpreted with caution due to potential extrapolation. The current IUCN distribution limits are displayed in **a** and **c**. Maps are outputs of Generalised Additive Models.

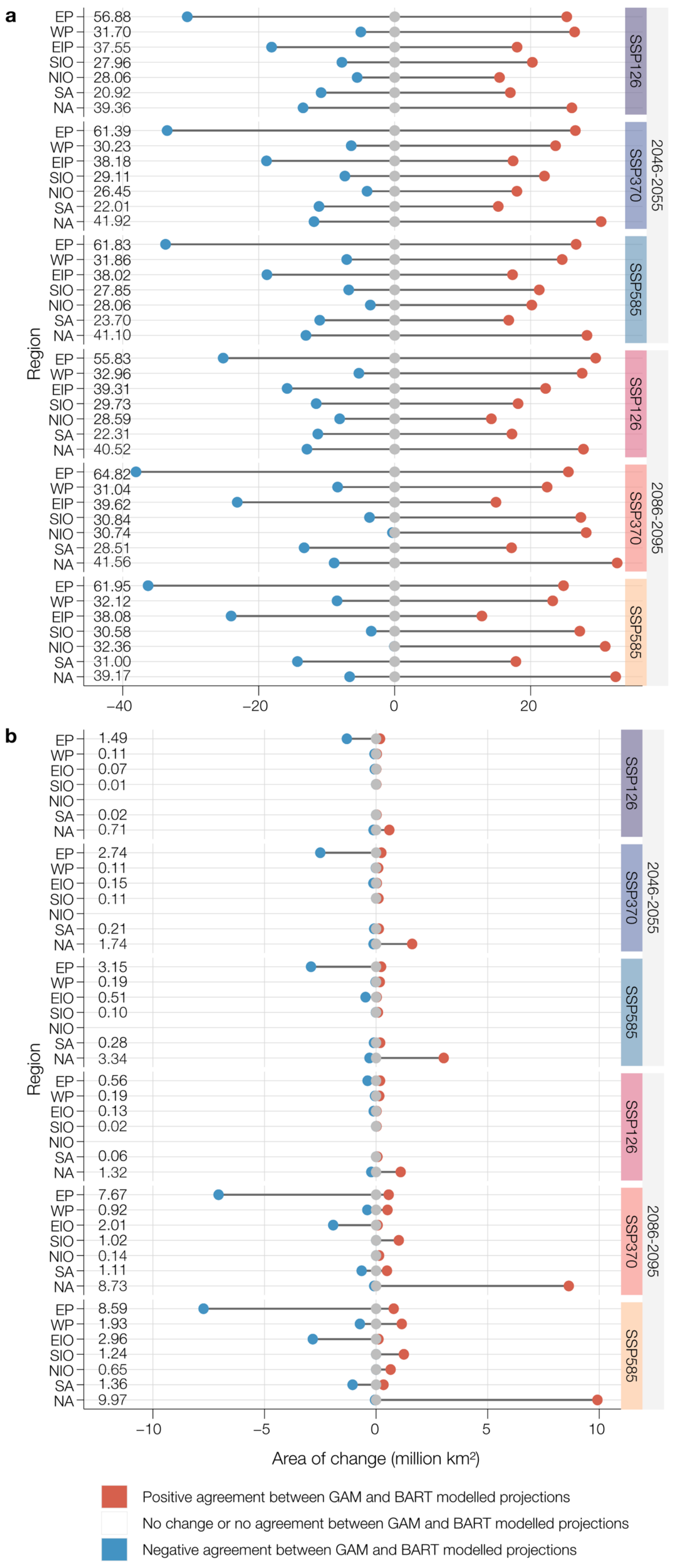


**Figure S8** | **Area of predicted changes in habitat suitability. a**, Total area (change in million km$^2$) of agreement, and **b,** areas with positive or negative agreement of >0.25 or <-0.25 (identified by both Generalised Additive Models and Bayesian Additive Regression Trees), respectively, located within each region. All panels are coloured by decade and scenario where values on the left of each row denote the total area of change and points denote total area of positive (red) and negative (blue) change within each boundary. For the location of regions of agreement see Figures S9 – S15).

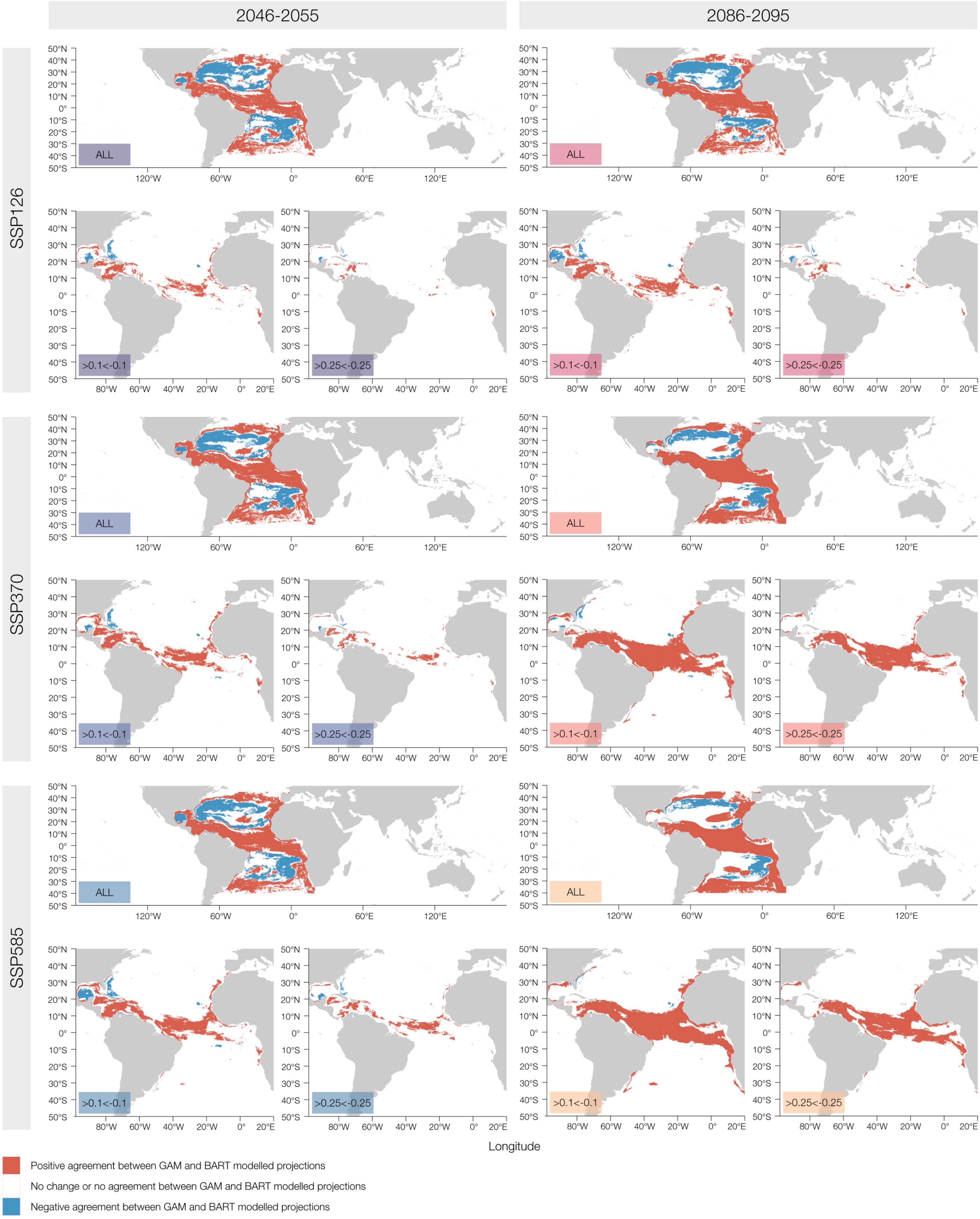


**Figure S9** | **Location of predicted changes in habitat suitability within the north Atlantic region. a**, Regions of positive (red), negative (blue) or no change (or agreement, white) identified by both Generalised Additive Models and Bayesian Additive Regression Trees in the north Atlantic coloured by each decade (columns) and scenario (rows). Within each decade and scenario combination the upper panel shows all regions of either positive or negative model agreement, the lower left shows regions of positive or negative agreement of >0.1 or <-0.1, respectively, and the lower right shows regions of positive or negative agreement >0.25 or <-0.25. For area calculations based on these locations see Figure S8.

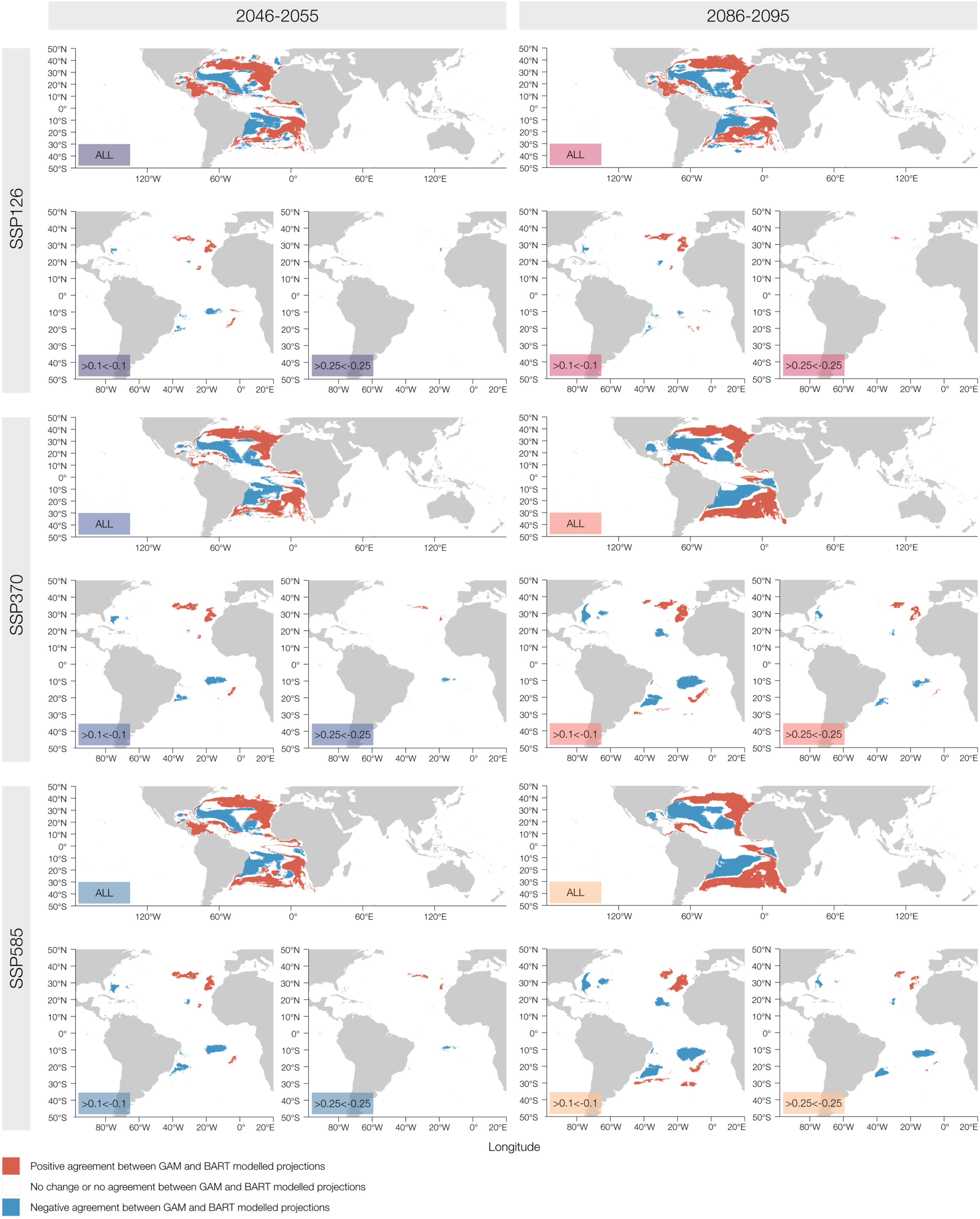


**Figure S10** | **Location of predicted changes in habitat suitability within the south Atlantic region. a**, Regions of positive (red), negative (blue) or no change (or agreement, white) identified by both Generalised Additive Models and Bayesian Additive Regression Trees in the south Atlantic coloured by each decade (columns) and scenario (rows). Within each decade and scenario combination the upper panel shows all regions of either positive or negative model agreement, the lower left shows regions of positive or negative agreement of >0.1 or <-0.1, respectively, and the lower right shows regions of positive or negative agreement >0.25 or <-0.25. For area calculations based on these locations see Figure S8.

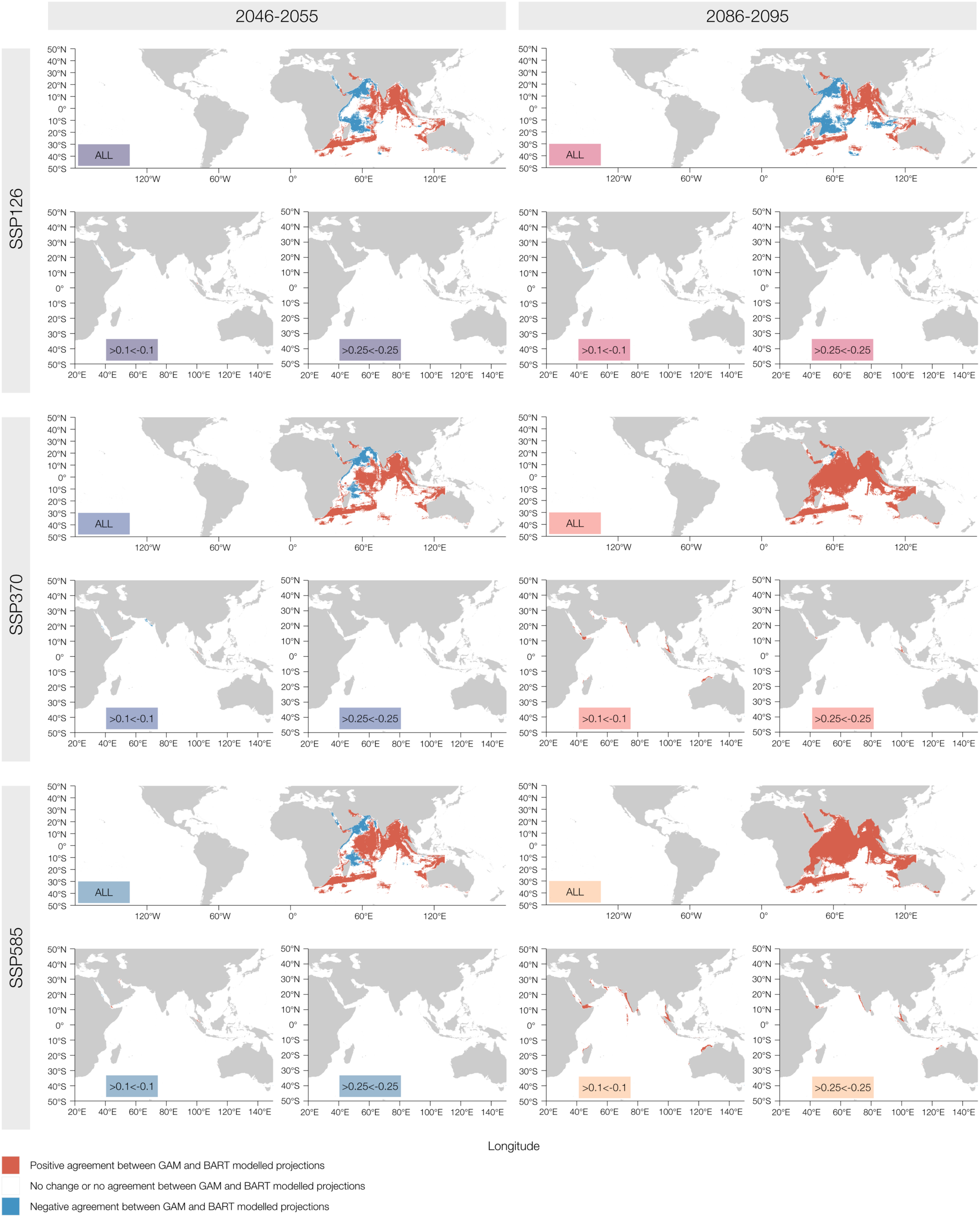


**Figure S11 | Location of predicted changes in habitat suitability within the northwest Indian Ocean region. a**, Regions of positive (red), negative (blue) or no change (or agreement, white) identified by both Generalised Additive Models and Bayesian Additive Regression Trees in the northwest Indian Ocean coloured by each decade (columns) and scenario (rows). Within each decade and scenario combination the upper panel shows all regions of either positive or negative model agreement, the lower left shows regions of positive or negative agreement of >0.1 or <-0.1, respectively, and the lower right shows regions of positive or negative agreement >0.25 or <-0.25. For area calculations based on these locations see Figure S8.

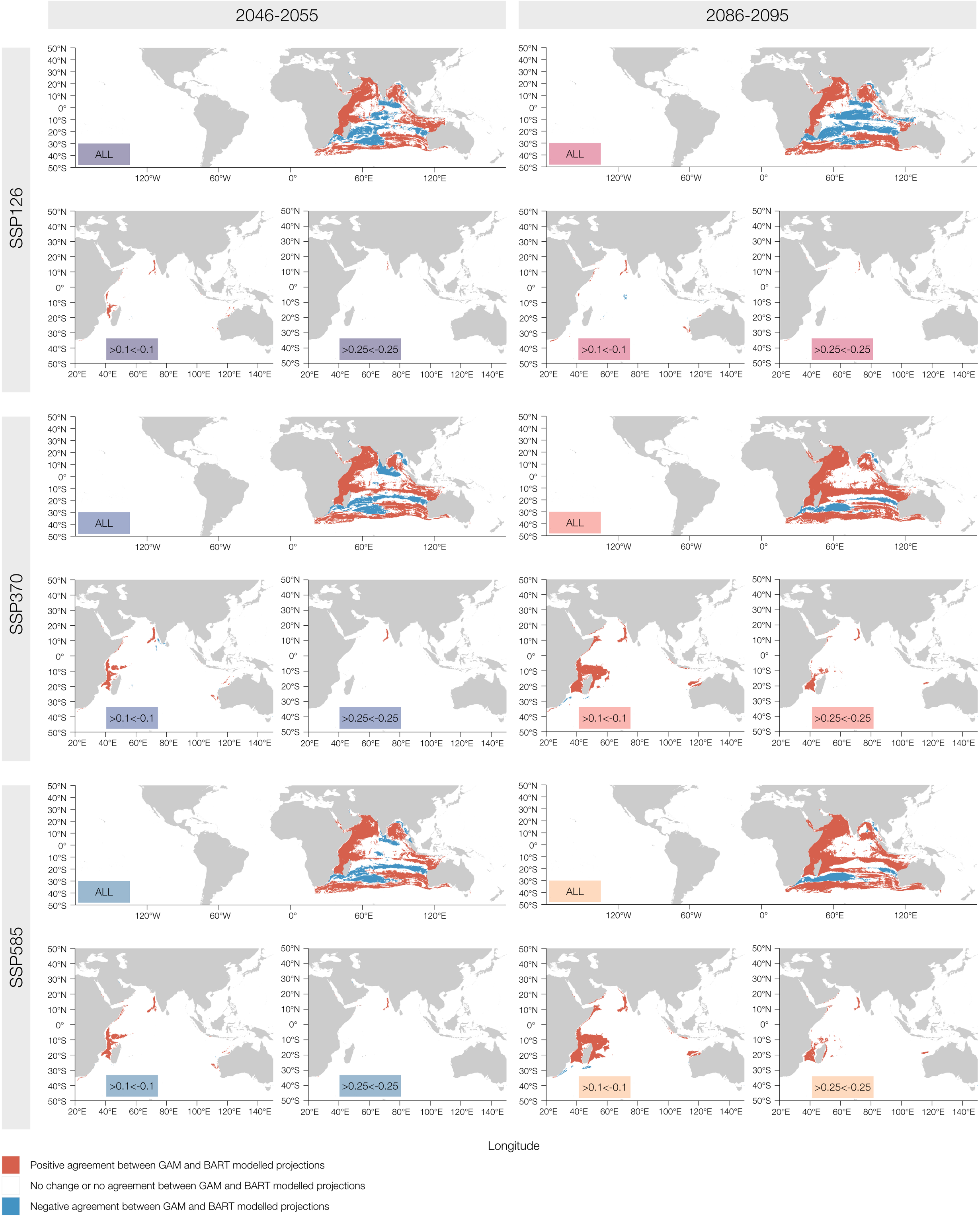


**Figure S12 | Location of predicted changes in habitat suitability within the southwest Indian Ocean region. a**, Regions of positive (red), negative (blue) or no change (or agreement, white) identified by both Generalised Additive Models and Bayesian Additive Regression Trees in the southwest Indian Ocean coloured by each decade (columns) and scenario (rows). Within each decade and scenario combination the upper panel shows all regions of either positive or negative model agreement, the lower left shows regions of positive or negative agreement of >0.1 or <-0.1, respectively, and the lower right shows regions of positive or negative agreement >0.25 or <-0.25. For area calculations based on these locations see Figure S8.

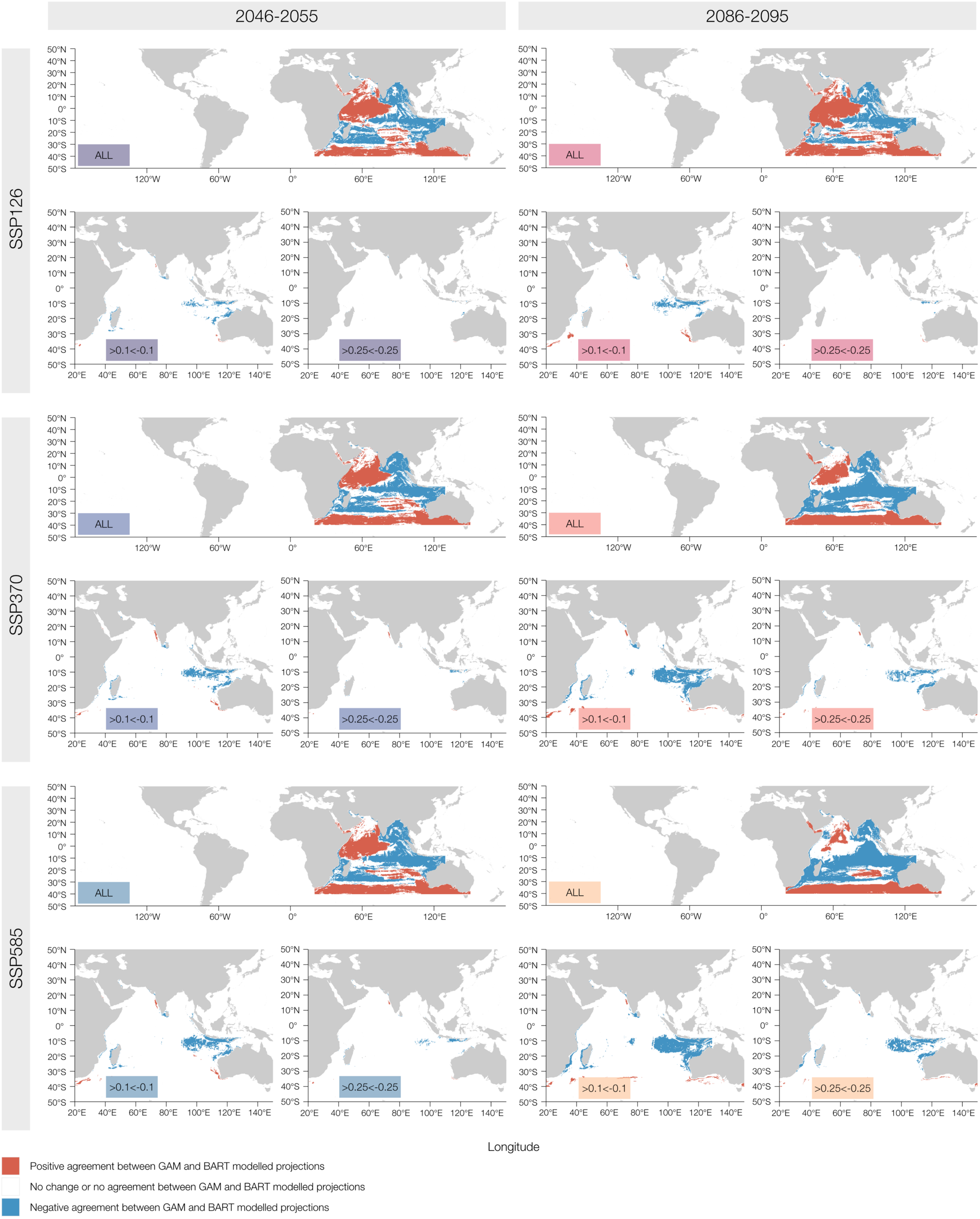


**Figure S13 | Location of predicted changes in habitat suitability within the east Indian Ocean region. a**, Regions of positive (red), negative (blue) or no change (or agreement, white) identified by both Generalised Additive Models and Bayesian Additive Regression Trees in the east Indian Ocean coloured by each decade (columns) and scenario (rows). Within each decade and scenario combination the upper panel shows all regions of either positive or negative model agreement, the lower left shows regions of positive or negative agreement of >0.1 or <-0.1, respectively, and the lower right shows regions of positive or negative agreement >0.25 or <-0.25. For area calculations based on these locations see Figure S8.

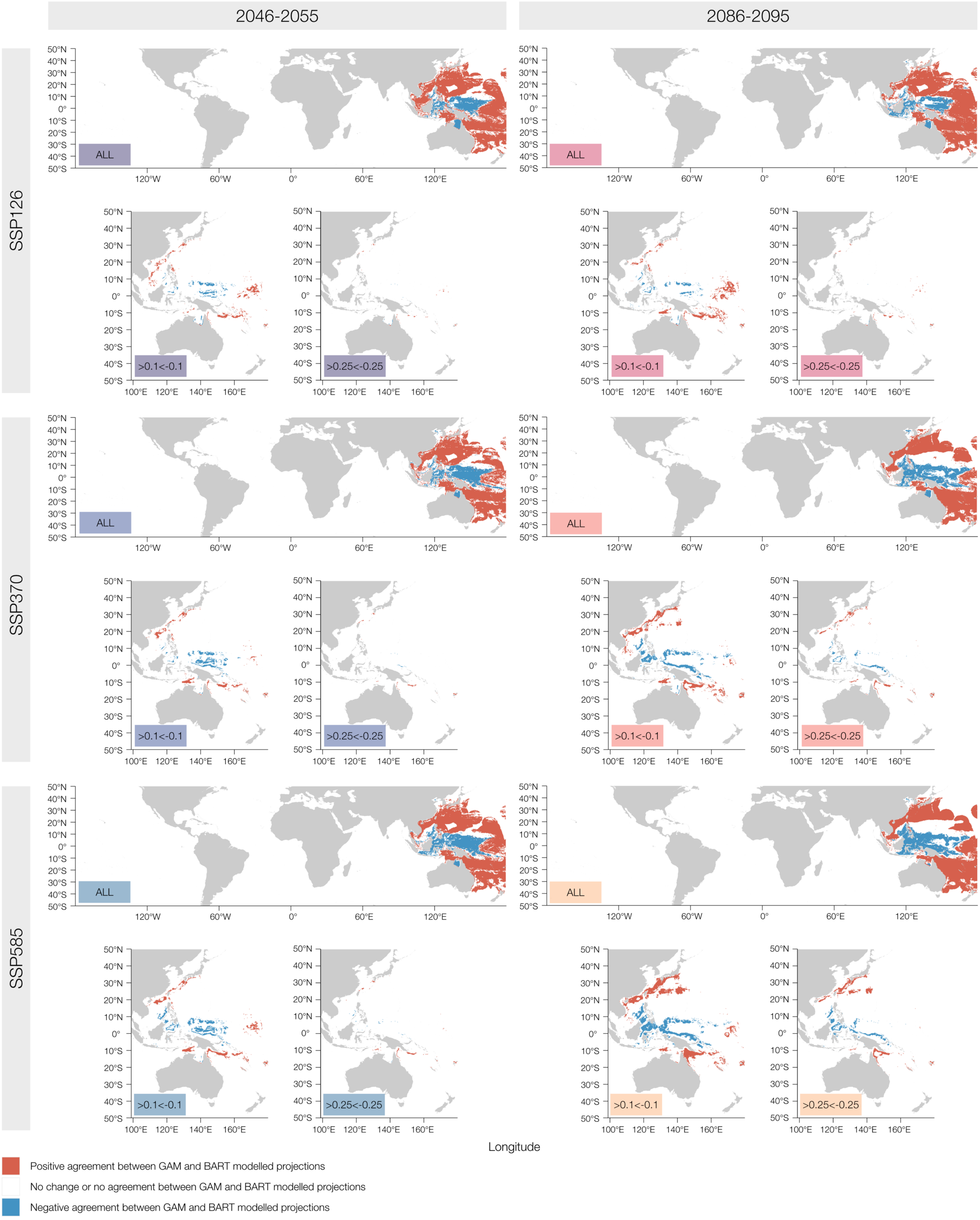


**Figure S14 | Location of predicted changes in habitat suitability within the west Pacific region. a**, Regions of positive (red), negative (blue) or no change (or agreement, white) identified by both Generalised Additive Models and Bayesian Additive Regression Trees in the west Pacific coloured by each decade (columns) and scenario (rows). Within each decade and scenario combination the upper panel shows all regions of either positive or negative model agreement, the lower left shows regions of positive or negative agreement of >0.1 or <-0.1, respectively, and the lower right shows regions of positive or negative agreement >0.25 or <-0.25. For area calculations based on these locations see Figure S8.

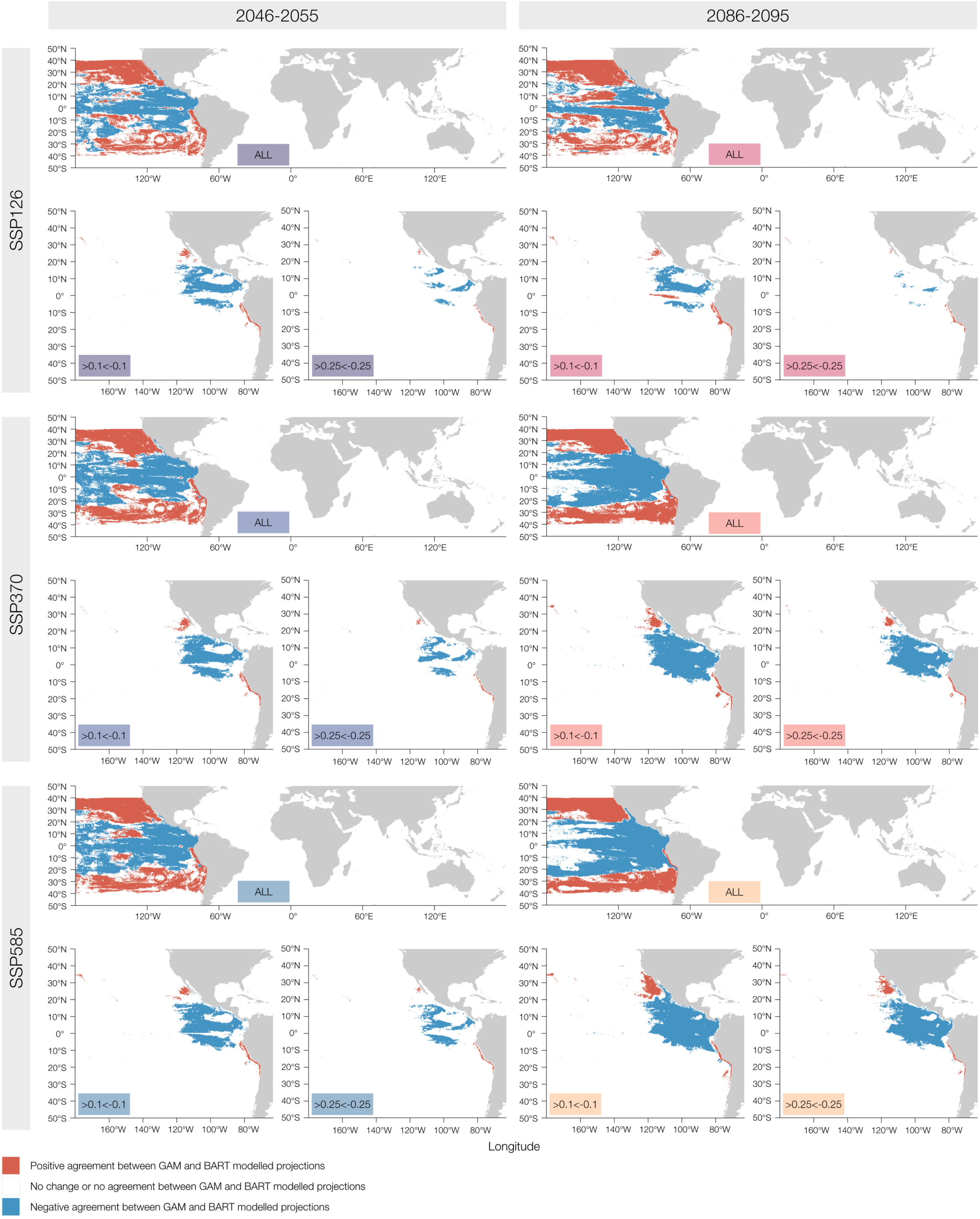


**Figure S15 | Location of predicted changes in habitat suitability within the east Pacific region. a**, Regions of positive (red), negative (blue) or no change (or agreement, white) identified by both Generalised Additive Models and Bayesian Additive Regression Trees in the east Pacific coloured by each decade (columns) and scenario (rows). Within each decade and scenario combination the upper panel shows all regions of either positive or negative model agreement, the lower left shows regions of positive or negative agreement of >0.1 or <-0.1, respectively, and the lower right shows regions of positive or negative agreement >0.25 or <-0.25. For area calculations based on these locations see Figure S8.

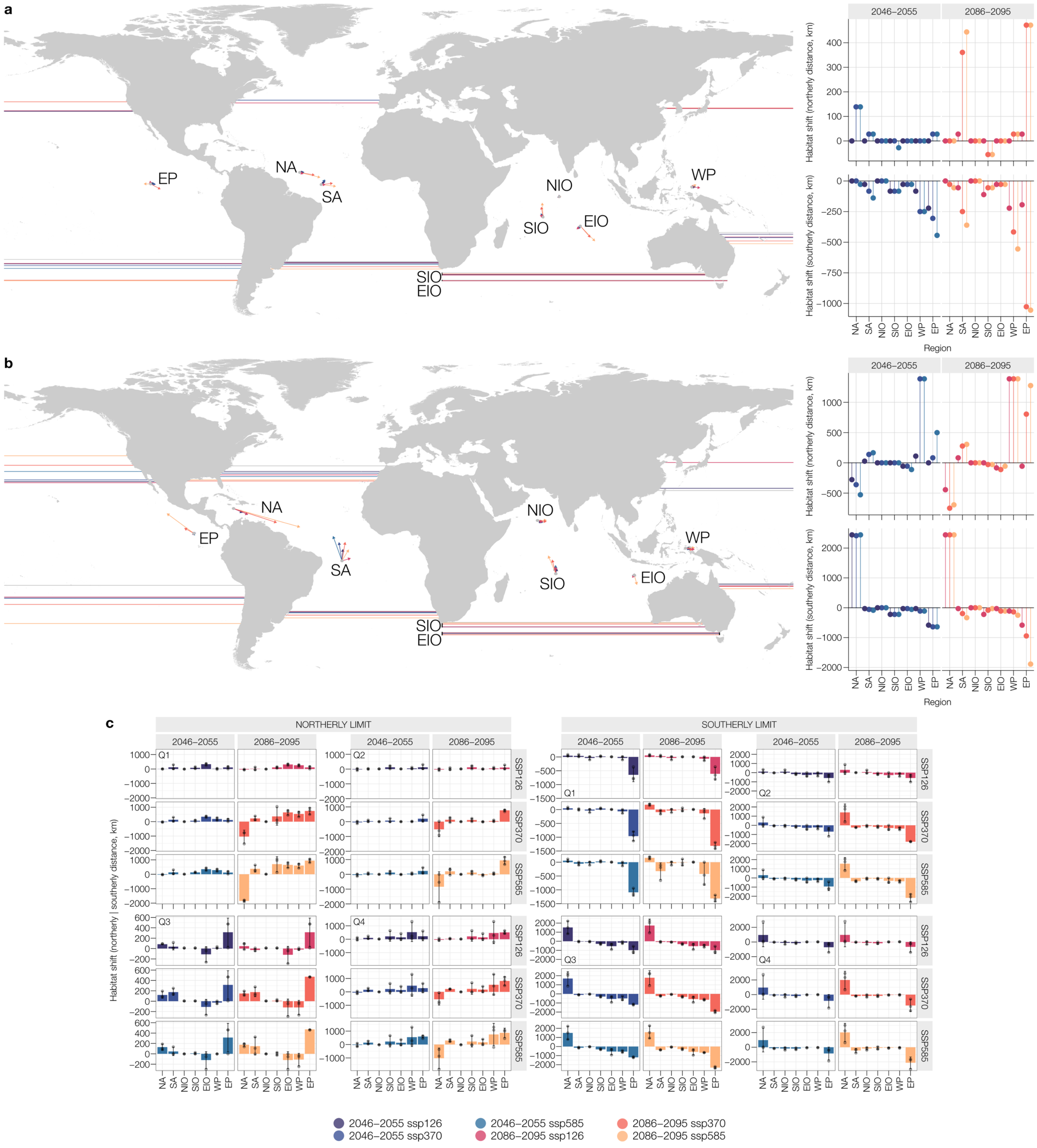


**Figure S16 | Shifts in habitat suitability. a**, Arrows (left panel) indicate shifts in the geographic mean of the relative 75th habitat suitability quantile, with the grey mapped points indicating the current geographic mean, and the arrowheads the future geographic mean coloured by decade and scenario. Lines indicate the maximum and minimum latitudes where the 75th the habitat suitability quantile was projected, with the grey line indicating the current geographic limits and the future geographic limits coloured by decade and scenario. The lollipop plots (right panel) indicate the corresponding northerly (right upper panel) and southerly (right bottom panel) habitat limit shifts (distance, km) within each region. Where **a**, shows the 75th habitat suitability quantile, **b**, shows the 95th quantile. Thresholds for habitat suitability are the relative percentile, calculated from the habitat suitability within each projection subset. Southerly limits for both the southwest Indian Ocean and east Indian Ocean regions are displayed in the maps. **c**, Northerly (left panel) and southerly (right panel) habitat limit shifts (distance, km) within each quarter (Q1: January, February, March | Q2: April, May, June | Q3: July, August, September | Q4: October, November, December) coloured by decade and scenario where the threshold for habitat suitability is the relative 90th percentile, calculated for habitat suitability predictions within each projection subset. The circles, bars and error bars denote individual monthly values, their average and the standard deviation, respectively.

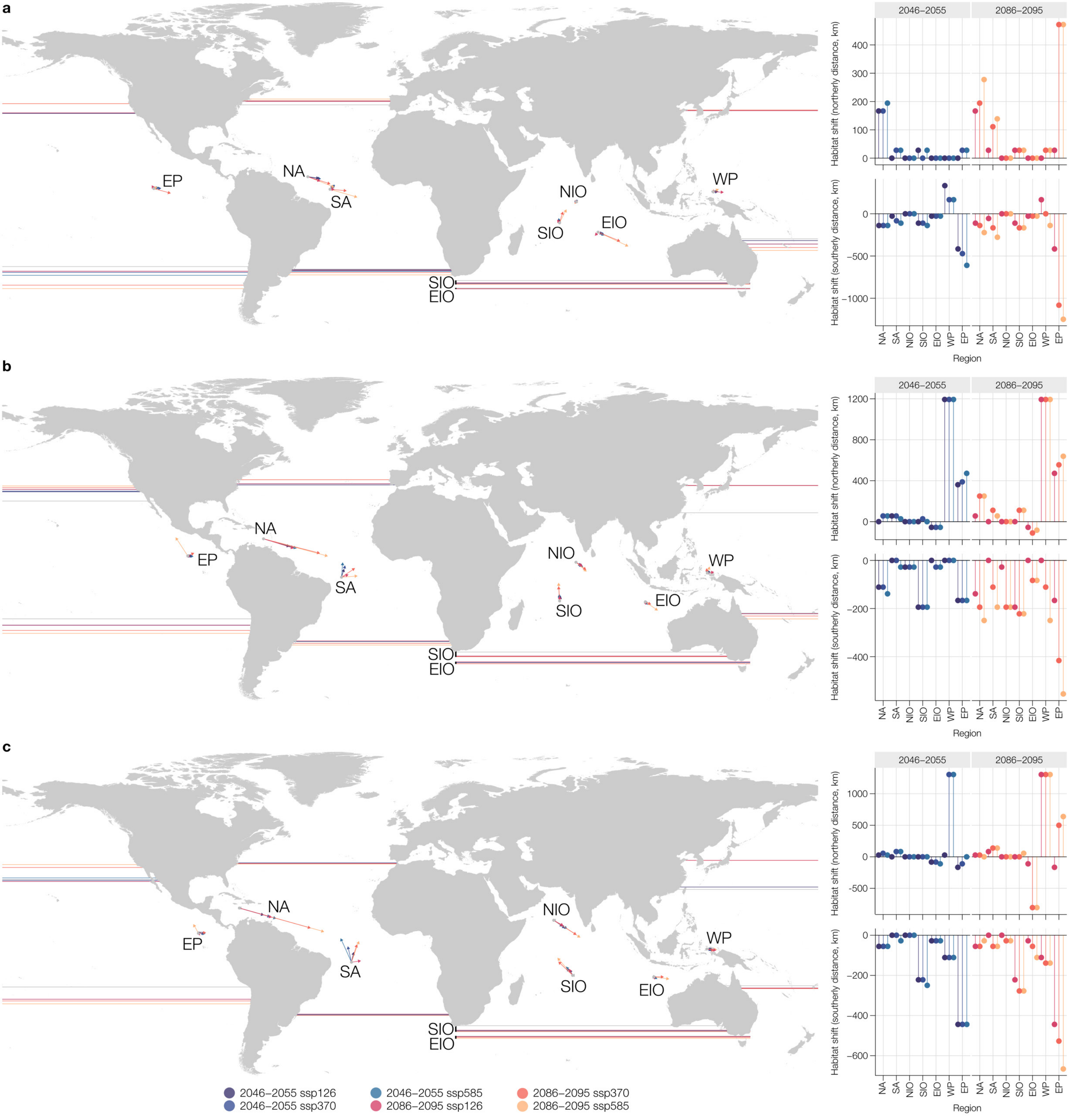


**Figure S17 | Shifts in habitat suitability. a**, Arrows (left panel) indicate shifts in the geographic mean of the 75th habitat suitability quantile, with the grey mapped points indicating the current geographic mean, and the arrowheads the future geographic mean coloured by decade and scenario. Lines indicate the maximum and minimum latitudes where the 75th the habitat suitability quantile was projected, with the grey line indicating the current geographic limits and the future geographic limits coloured by decade and scenario with the lollipop plots (right panel) indicating the corresponding northerly (right upper panel) and southerly (right bottom panel) habitat limit shifts (distance, km) within each region. Where **a**, shows the 75th habitat suitability quantile, **b**, and **c**, show the 90th and 95th quantiles, respectively. Thresholds for habitat suitability are the baseline percentile, calculated from the annual mean habitat suitability for the current distribution (2005 – 2019). Southerly limits for both the southwest Indian Ocean and east Indian Ocean regions are displayed in the maps.

a

b

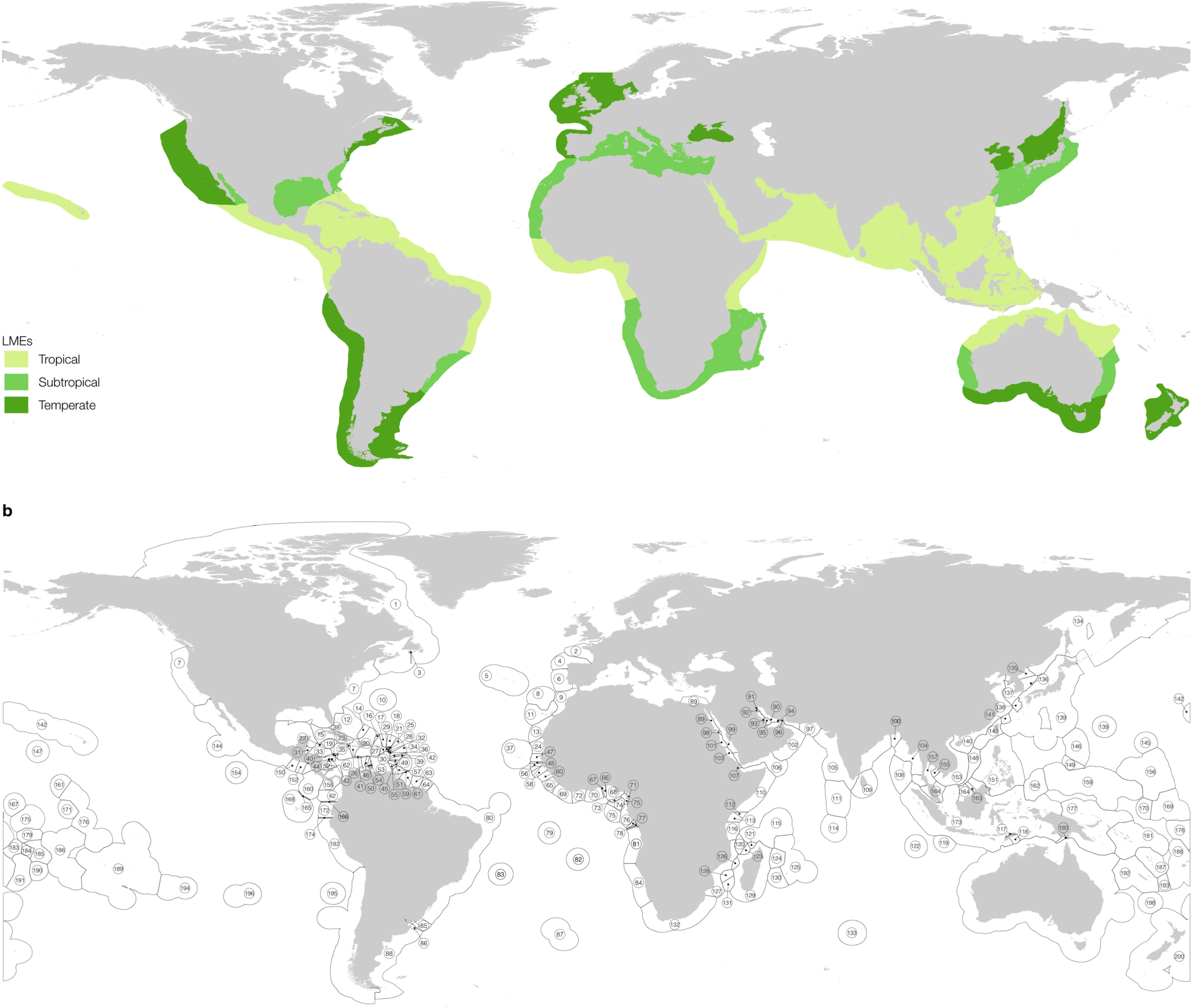


1) Canadian EEZ
2) French EEZ
3) Saint–Pierre and Miquelon EEZ
4) Spanish EEZ
5) Portuguese EEZ (Azores)
6) Portuguese EEZ
7) United States EEZ
8) Portuguese EEZ (Madeira)
9) Moroccan EEZ
10) Bermudian EEZ
11) Spanish EEZ (Canary Islands)
12) Bahamas EEZ
13) OC: Western Saharan EEZ
14) Turks and Caicos EEZ
15) Cuban EEZ
16) OC: Puerto Rico / Dominican Republic
17) British Virgin Islands EEZ
18) Anguilla EEZ
19) Cayman Islands EEZ
20) Dominican Republic EEZ
21) Antigua and Barbuda EEZ
22) JR: Honduras / Cayman Islands
23) OC: Navassa Island: USA / Haiti / Jamaica
24) Mauritanian EEZ
25) Saint–Barthélemy EEZ
26) Haitian EEZ
27) Puerto Rican EEZ
28) Saba EEZ
29) Sint–Eustatius EEZ
30) Virgin Islander EEZ
31) Belizean EEZ
32) Saint Kitts and Nevis EEZ
33) Honduran EEZ
34) Guadeloupean EEZ
35)Jamaican EEZ
36) Montserrat EEZ
37) Cape Verdean EEZ
38) Colombian EEZ (Serranilla)
39) Dominican EEZ
40) JR: Colombia / Jamaica
41) JR: Colombia / Dominican Republic
42) Martinican EEZ
43) Colombian EEZ (Serrana)
44) Colombian EEZ (Quitasueño)
45) Saint Lucia EEZ
46) Aruban EEZ
47) Senegalese EEZ
48) Gambian EEZ
49) Barbados EEZ
50) Curaçaoan EEZ
51) Saint Vincent & the Grenadines EEZ
52) Nicaraguan EEZ
53) Venezuelan EEZ
54) Bonaire EEZ
55) Grenadian EEZ
56) JR: Senegal / Guinea Bissau
57) Trinidad & Tobago EEZ
58) Guinea Bissau EEZ
59) OC: Trinidad & Tobago / Venezuela / Guyana
60) Guinean EEZ
61) Guyanese EEZ
62) Colombian EEZ
63) Surinamese EEZ
64) French Guiana EEZ
65) Sierra Leonian EEZ
66) Beninese EEZ
67) Togolese EEZ
68) Nigerian EEZ
69) Liberian EEZ
70) Ghanaian EEZ
71) Cameroonian EEZ
72) Ivory Coast EEZ
73) JR: Nigeria / Sao Tome and Principe
74) Sao Tome & Principe EEZ
75) Equatorial Guinean EEZ
76) Gabonese EEZ
77) Congolese EEZ
78) Democratic Republic of the Congo EEZ
79) Ascension EEZ
80) Brazilian EEZ
81) Angolan EEZ
82) St. Helena EEZ
83) Brazilian EEZ (Trindade)
84) Namibian EEZ
85) Uruguayan EEZ
86) JR: Argentina / Uruguay
87) Tristan Da Cunha EEZ
88) Argentinean EEZ
89) Egyptian EEZ
90) Iranian EEZ
91) Iraqi EEZ
92) Kuwaiti EEZ
93) Bahraini EEZ
94) OC: Iran / United Arab Emirates
95) Qatari EEZ
96) United Arab Emirates EEZ
97) Pakistani EEZ
98) OC: Sudan / Egypt
99) Saudi Arabian EEZ
100) Bangladeshi EEZ
101) Sudanese EEZ
102) Omani EEZ
103) Eritrean EEZ
104) Myanmar EEZ
105) Indian EEZ
106) Yemeni EEZ
107) Djiboutian EEZ
108) Indian EEZ (Andaman & Nicobar Islands)
109) Sri Lankan EEZ
110) Somali EEZ
111) Maldives EEZ
112) OC: Kenya / Somalia
113) Kenyan EEZ
114) Chagos Archipelago EEZ
115) Seychellois EEZ
116) Tanzanian EEZ
117) Oecussi Ambeno EEZ
118) East Timorian EEZ
119) Christmas Island EEZ
120) Comoran EEZ
121) OC: Glorioso Islands: France / Madagascar
122) Cocos Islands EEZ
123) OC: Mayotte: France / Comoros
124) OC: Ile Tromelin: Reunion / Mada. / Mauritus
125) Mauritian EEZ
126) Juan de Nova EEZ
127) Mozambican EEZ
128) Bassas da India EEZ
129) Madagascan EEZ
130) Réunion EEZ
131) Ile Europa EEZ
132) South African EEZ
133) Amsterdam Island & St. Paul Island EEZ
134) Russian EEZ
135) North Korean EEZ
136) OC: Liancourt Rocks: Japan / South Korea
137) South Korean EEZ
138) JR: area Japan / Korea
139) Japanese EEZ
140) Chinese EEZ
141) OC: Senkaku Islands: Japan / China / Taiwan
142) United States EEZ (Hawaii)
143) Taiwanese EEZ
144) Mexican EEZ
145) Wake Island EEZ
146) Northern Mariana EEZ
147) Johnston Atoll EEZ
148) OC: South China Sea
149) Guam EEZ
150) Guatemalan EEZ
151) Philippines EEZ
152) El Salvador EEZ
153) Vietnamese EEZ
154) Clipperton EEZ
155) Cambodian EEZ
156) Marshall Islands EEZ
157) Thailand EEZ
158) Panamanian EEZ
159) Micronesian EEZ
160) Costa Rican EEZ
161) Palmyra Atoll EEZ
162) Palau EEZ
163) Bruneian EEZ
164) Malaysian EEZ
165) JR: Costa Rica / Ecuador (Galapagos)
166) JR: Ecuador / Colombia
167) Howland & Baker Islands EEZ
168) Ecuadorian EEZ (Galapagos)
169) Kiribati EEZ (Gilbert Islands)
170) Nauruan EEZ
171) Jarvis Island EEZ
172) Ecuadorian EEZ
173) Indonesian EEZ
174) JR: Peru / Ecuador
175) Kiribati EEZ (Phoenix Islands)
176) Kiribati EEZ (Line Islands)
177) Papua New Guinean EEZ
178) Tuvaluan EEZ
179) Tokelau EEZ
180) Protected Zone est. under the Torres Strait Treaty
181) Solomon Islands EEZ
182) Peruvian EEZ
183) Wallis and Futuna EEZ
184) Samoan EEZ
185) American Samoa EEZ
186) Cook Islands EEZ
187) Vanuatu EEZ
188) Fijian EEZ
189) French Polynesian EEZ
190) Niue EEZ
191) Tongan EEZ
192) New Caledonian EEZ
193) OC: Matthew & Hunter Isd.: New Caledonia / Vanuatu
194) Pitcairn Islands EEZ
195) Chilean EEZ (San Felix and San Ambrosio isd.)
196) Chilean EEZ (Easter Island)
197) Australian EEZ
198) Norfolk Island EEZ
199) Chilean EEZ
200) New Zealand EEZ

**Figure S18 | Geo-political boundaries. a**, Large Marine Ecosystem (LME) boundaries coloured by climate zone. **b**, Exclusive Economic Zones (EEZs) within the study regions.

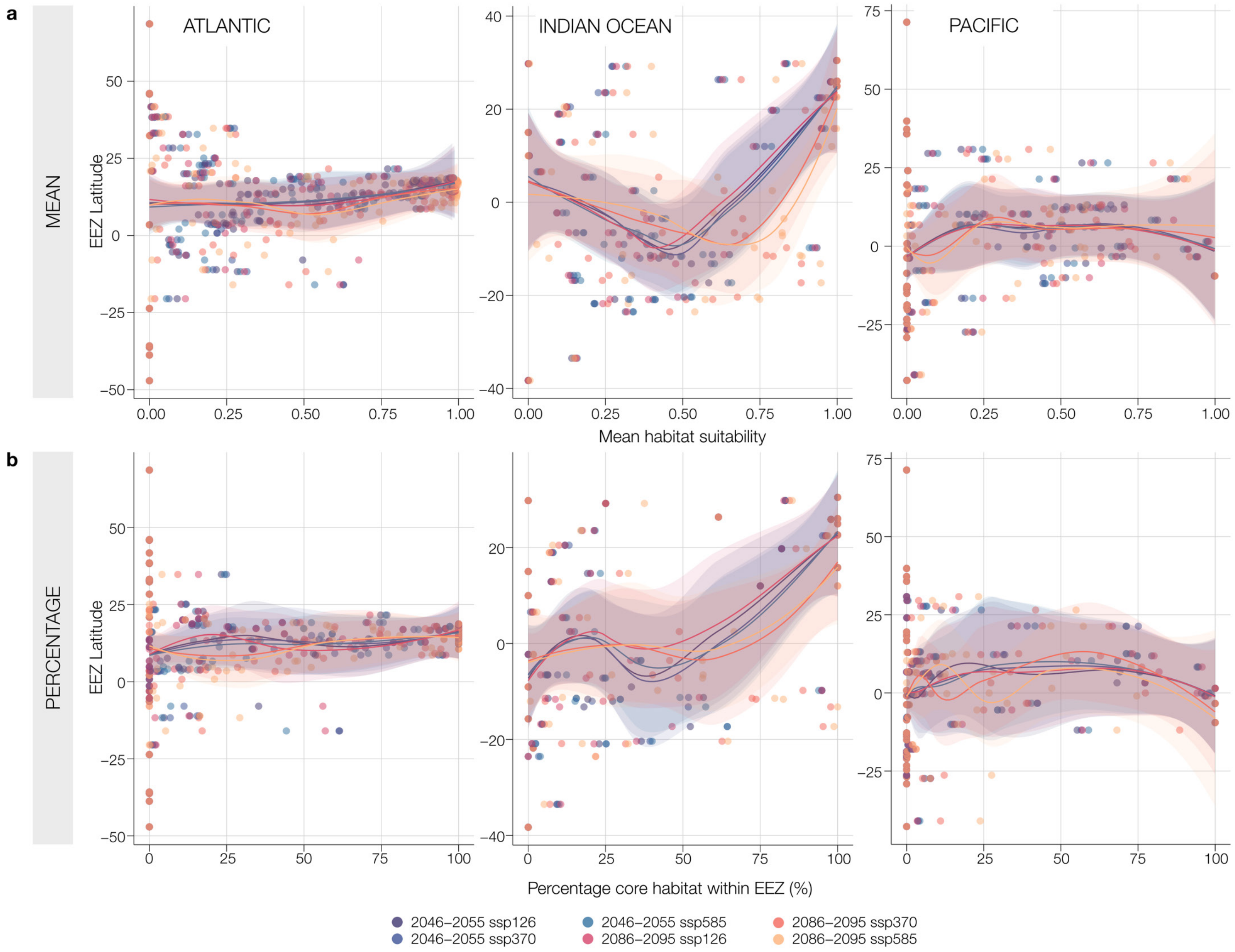


**Figure S19 | Latitudinal habitat suitability metrics. a**, Mean habitat suitability within Exclusive Economic Zones (EEZs) in the Atlantic (left), Indian Ocean (centre) and Pacific (right) coloured by decade and scenario combination and ordered by the latitudinal centroid of the EEZ. Lines show the locally estimated scatterplot smoothing (loess) as a local regression with shaded 95% confidence intervals. **b**, Percentage (%) of EEZ waters classed as core habitat in the Atlantic (left), Indian Ocean (centre) and Pacific (right) coloured by decade and scenario combination and ordered by the latitudinal centroid of the EEZ. Lines show the locally estimated scatterplot smoothing (loess) as a local regression with shaded 95% confidence intervals. Threshold for habitat suitability is the relative 90$^{th}$ percentile, calculated for annual mean habitat suitability within each temporal subset.

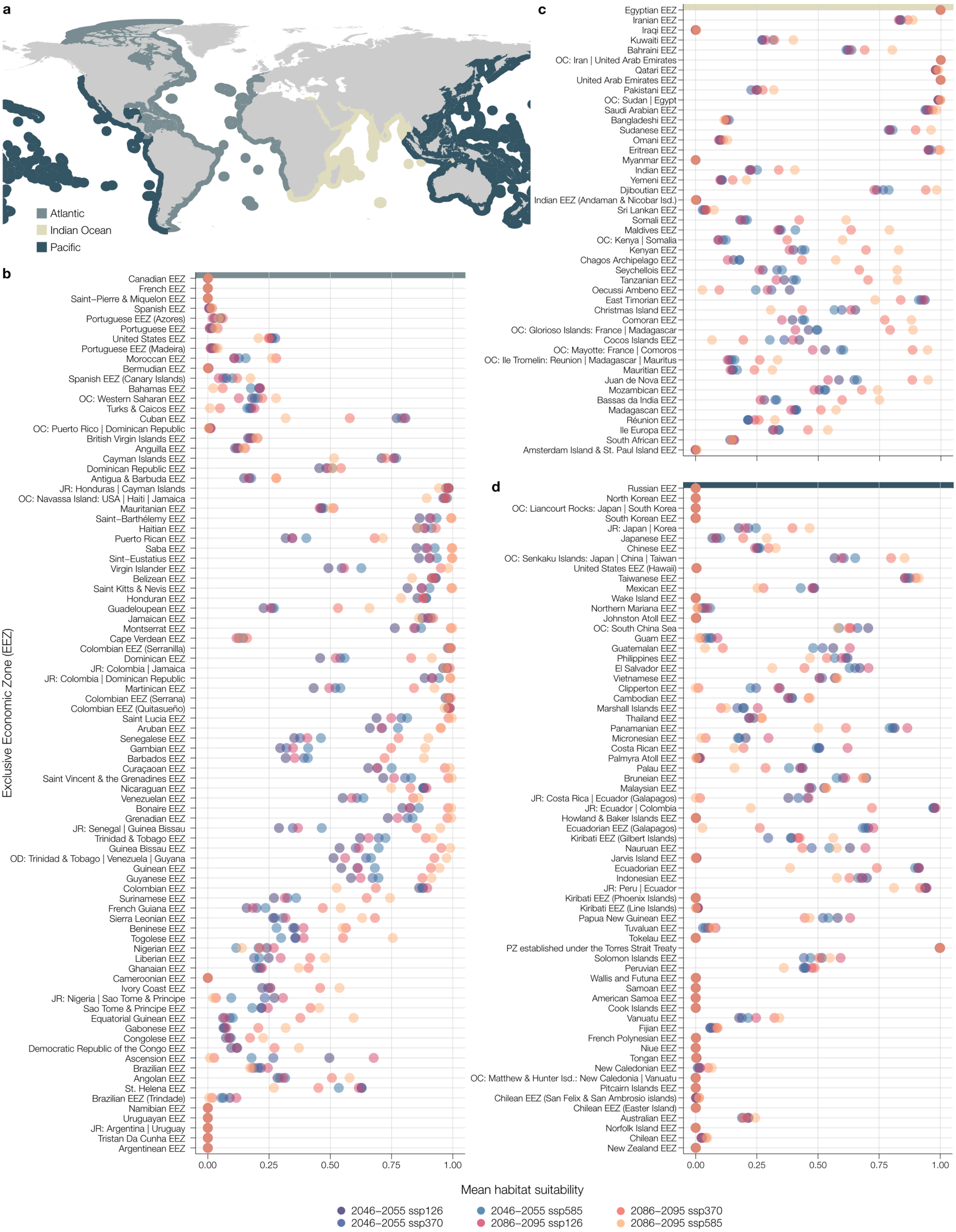


**Figure S20 | Latitudinal habitat suitability metrics. a**, Exclusive Economic Zones (EEZs) coloured by major ocean basin. **b**, Mean habitat suitability within EEZs in the Atlantic coloured by decade and scenario combination and ordered by the latitudinal centroid of the EEZ. The same is shown in the **c**, Indian Ocean and, **d**, Pacific. EEZs were assigned to an ocean based on where the majority of area was located.

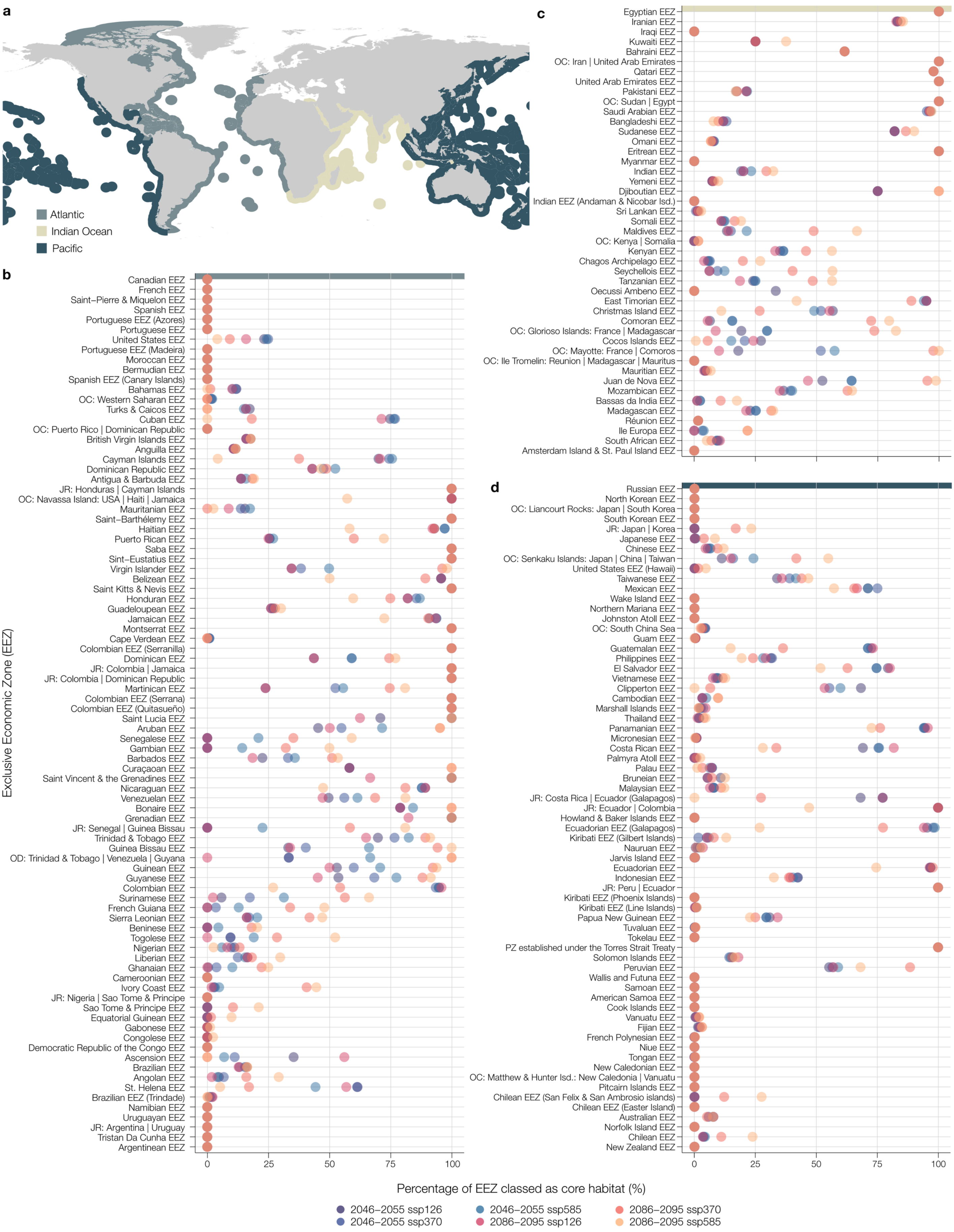


**Figure S21 | Latitudinal habitat suitability metrics. a**, Exclusive Economic Zones (EEZs) coloured by major ocean basin. **b**, Percentage (%) of EEZ classed as core habitat in the Atlantic coloured by decade and scenario combination and ordered by the latitudinal centroid of the EEZ. The same is shown in the **c**, Indian Ocean and, **d**, Pacific. Threshold for habitat suitability is the relative 90[th] percentile, calculated for annual mean habitat suitability within each temporal subset. EEZs were assigned to an ocean based on where the majority of area was located.

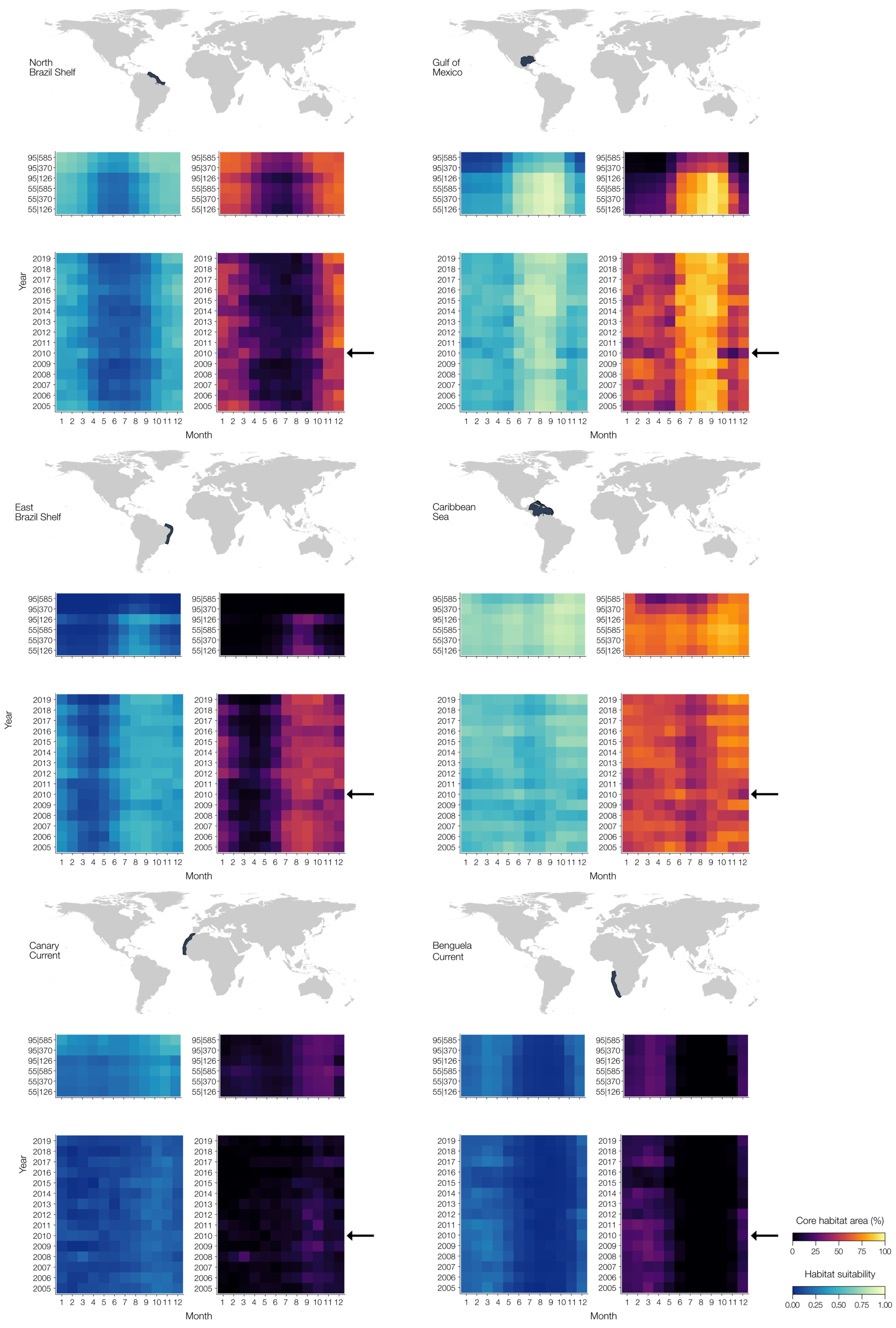
North Brazil Shelf
Gulf of Mexico
East Brazil Shelf
Caribbean Sea
Canary Current
Benguela Current
95|585
95|370
95|126
55|585
55|370
55|126
Year
Month
Core habitat area (%)
0 25 50 75 100
Habitat suitability
0.00 0.25 0.50 0.75 1.00

**Figure S22 | Temporal trends in monthly habitat suitability in the Atlantic.** Large Marine Ecosystems (LMEs) in the Atlantic where low (blue) and high (yellow-green) mean habitat suitability (left panels within each example) and low (black) and high (yellow) core habitat area coverage (%, right panels within each example) are shown for current predictions (lower panels within each example) and future projections (upper panel within each example) across the months. Threshold for habitat suitability is the relative $90^{th}$ percentile, calculated for annual mean habitat suitability within each temporal subset. Axis labels 55 and 95 refer to decadal subsets 2046 – 2055 and 2086 – 2095, respectively. Arrows mark years referenced in the text when past climatic events occurred.

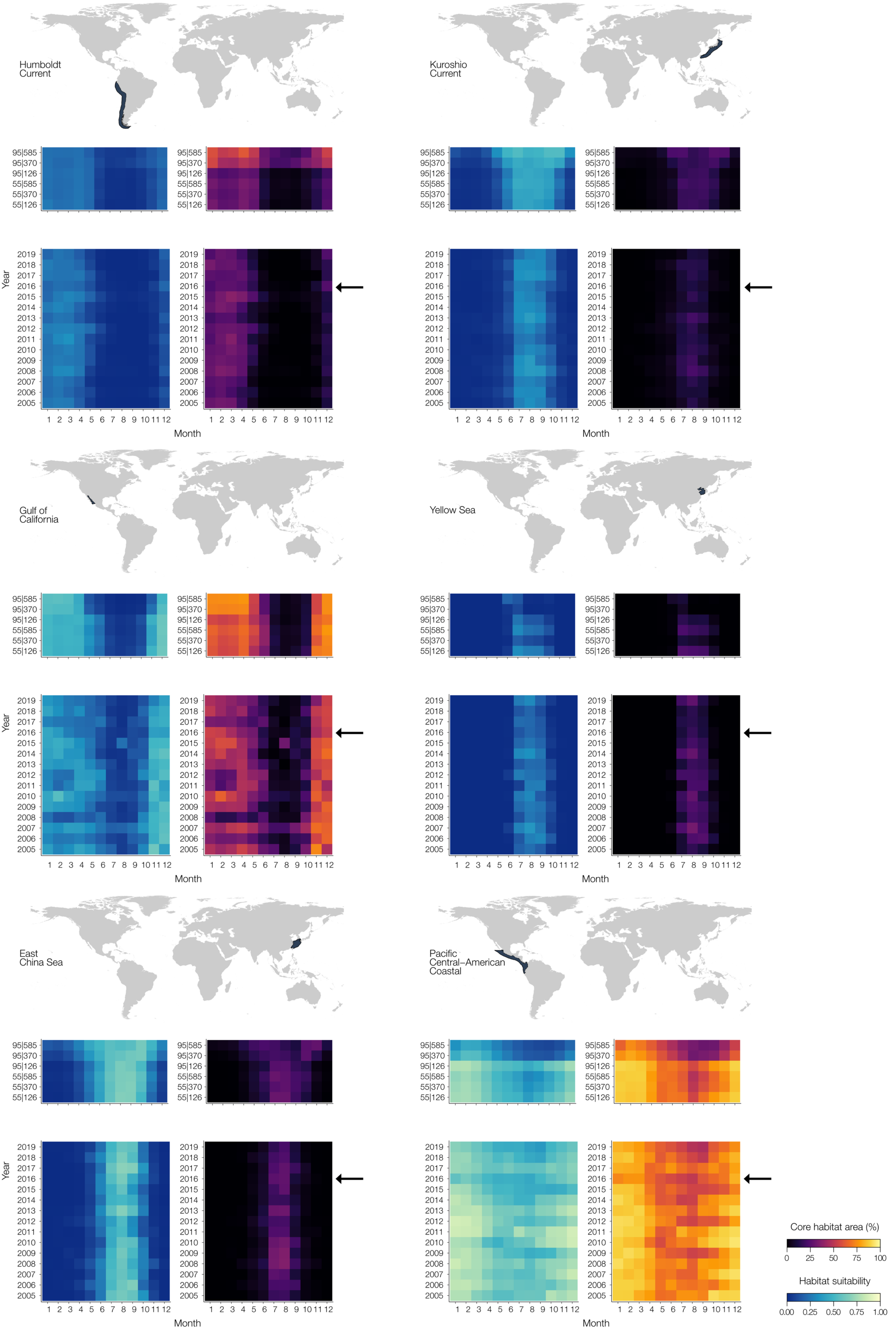
Humboldt Current
Kuroshio Current
Gulf of California
Yellow Sea
East China Sea
Pacific Central–American Coastal
95|585
95|370
95|126
55|585
55|370
55|126
2019
2018
2017
2016
2015
2014
2013
2012
2011
2010
2009
2008
2007
2006
2005
Year
1 2 3 4 5 6 7 8 9 10 11 12
Month
Core habitat area (%)
0 25 50 75 100
Habitat suitability
0.00 0.25 0.50 0.75 1.00

**Figure S23 | Temporal trends in monthly habitat suitability in the Pacific.** Large Marine Ecosystems (LMEs) in the Pacific where low (blue) and high (yellow-green) mean habitat suitability (left panels within each example) and low (black) and high (yellow) core habitat area coverage (%, right panels within each example) are shown for current predictions (lower panels within each example) and future projections (upper panel within each example) across the months. Threshold for habitat suitability is the relative 90$^{th}$ percentile, calculated for annual mean habitat suitability within each temporal subset. Axis labels 55 and 95 refer to decadal subsets 2046 – 2055 and 2086 – 2095, respectively. Arrows mark years referenced in the text when past climatic events occurred.

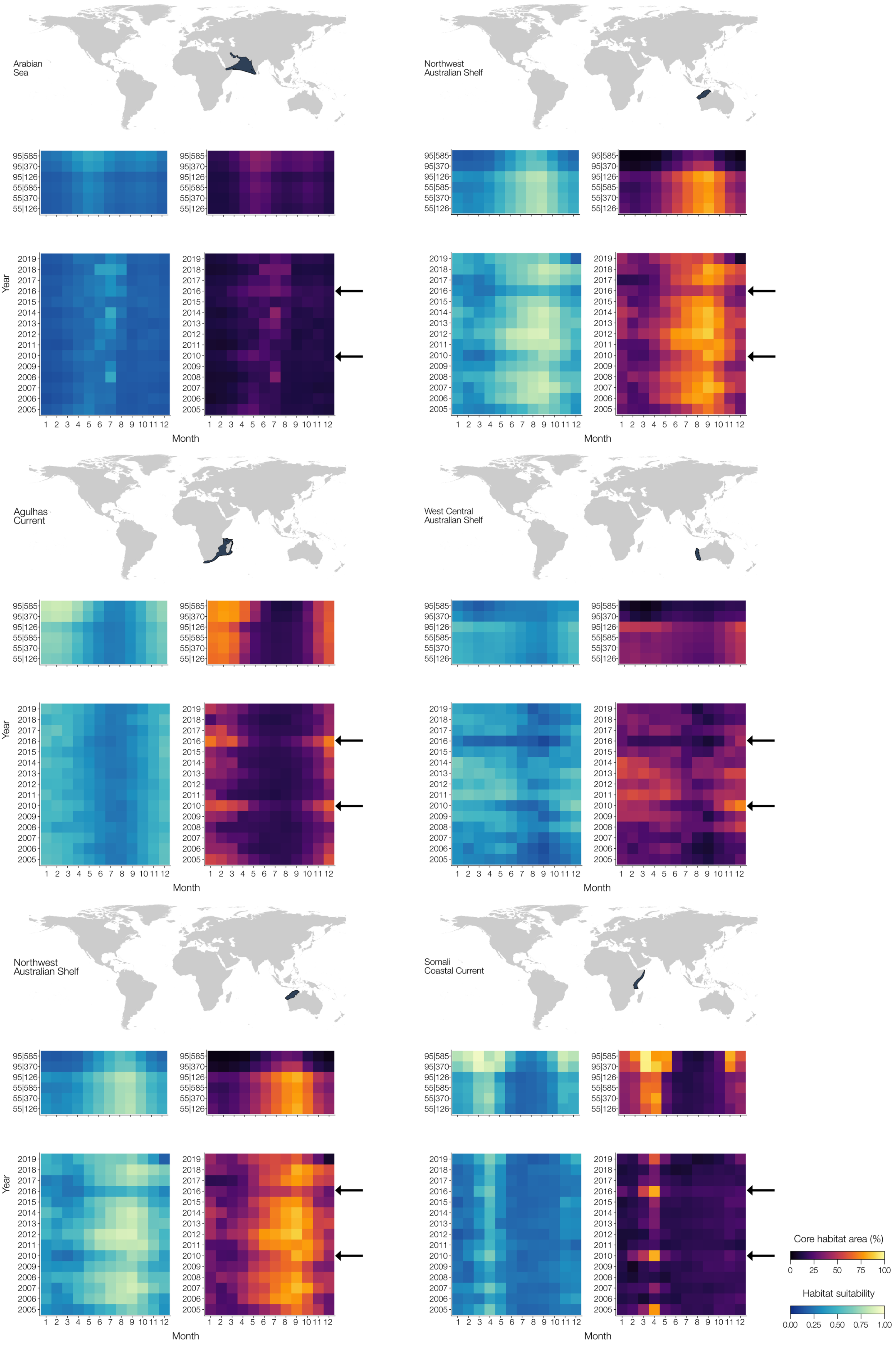

Arabian Sea
Northwest Australian Shelf
Agulhas Current
West Central Australian Shelf
Northwest Australian Shelf
Somali Coastal Current
95|585
95|370
95|126
55|585
55|370
55|126
Year
2019
2018
2017
2016
2015
2014
2013
2012
2011
2010
2009
2008
2007
2006
2005
1 2 3 4 5 6 7 8 9 10 11 12
Month
Core habitat area (%)
0 25 50 75 100
Habitat suitability
0.00 0.25 0.50 0.75 1.00

**Figure S24 | Temporal trends in monthly habitat suitability in the Indian Ocean.** Large Marine Ecosystems (LMEs) in the Indian Ocean where low (blue) and high (yellow-green) mean habitat suitability (left panels within each example) and low (black) and high (yellow) core habitat area coverage (%, right panels within each example) are shown for current predictions (lower panels within each example) and future projections (upper panel within each example) across the months. Threshold for habitat suitability is the relative 90$^{th}$ percentile, calculated for annual mean habitat suitability within each temporal subset. Axis labels 55 and 95 refer to decadal subsets 2046 – 2055 and 2086 – 2095, respectively. Arrows mark years referenced in the text when past climatic events occurred.

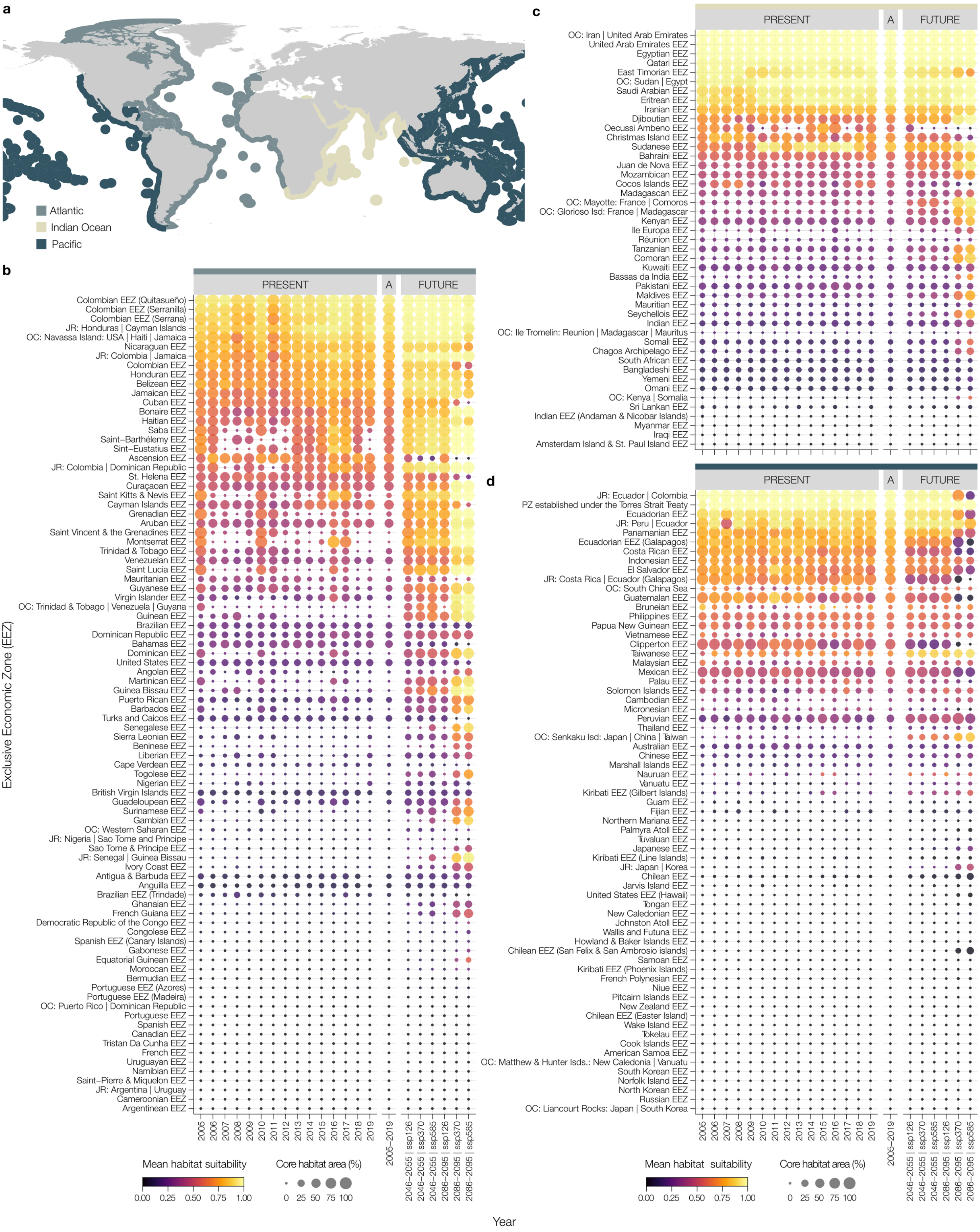


**Figure S25 | Temporal trends in monthly habitat within geo-political boundaries. a**, Exclusive Economic Zones (EEZs) coloured by major ocean basin. **b**, Annual habitat suitability displaying high (yellow) and low (black) means and high (large) and low (small) percentage coverage of core habitat areas (%) within EEZs. Left, centre and right facets show current annual (2005:2019), current average (2005 – 2019) and projected future (for each decade and scenario), respectively. The same is shown in the **c**, Indian Ocean and, **d**, Pacific. Threshold for habitat suitability is the relative 90th percentile, calculated for annual mean habitat suitability within each temporal subset. EEZs were assigned to an ocean based on where the majority of area was located.

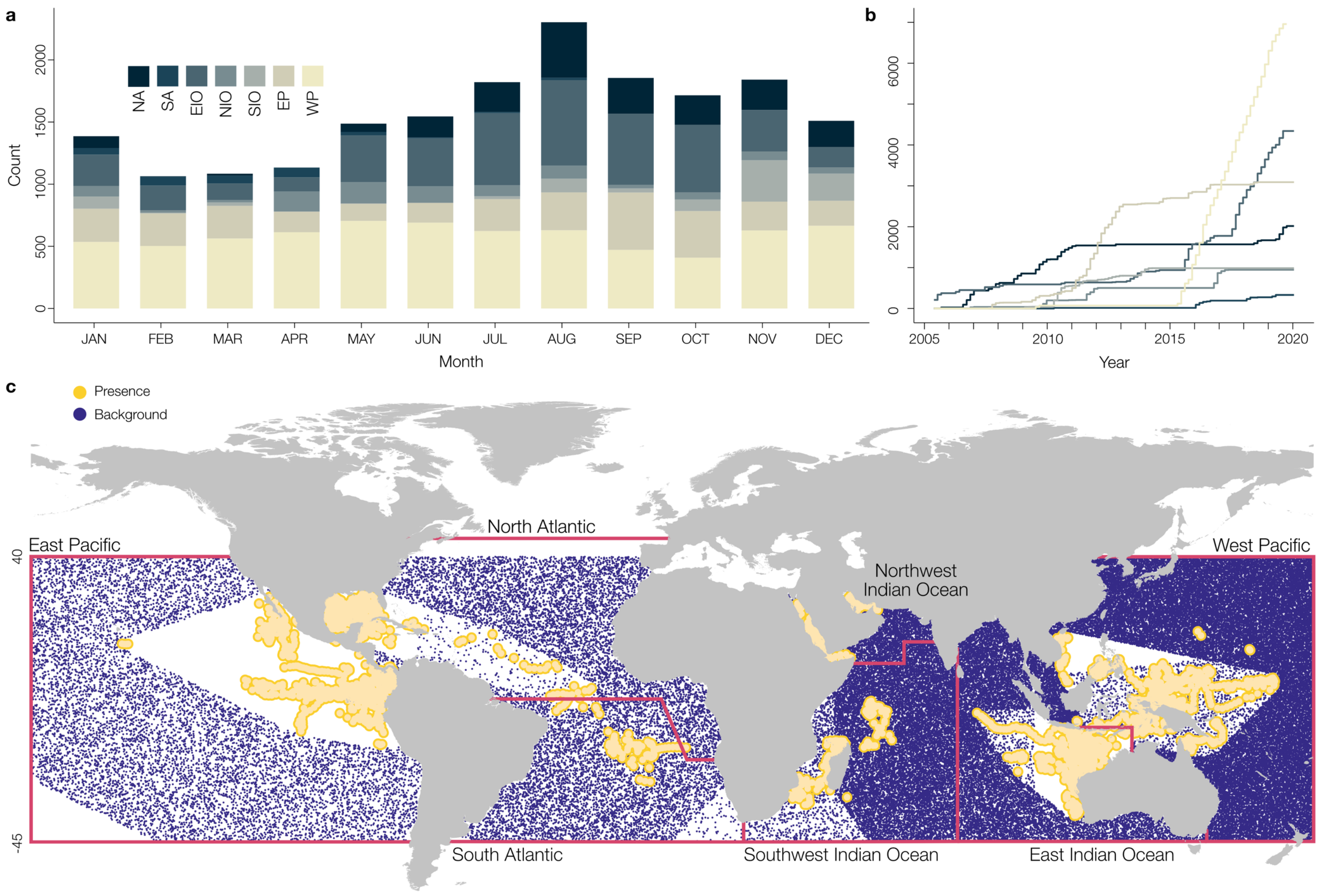


**Figure S26** | **Training data summary. a**, Total count of unfiltered whale shark locations per month and, **b**, cumulative count of unfiltered locations over the study period (2005 – 2019) coloured by region. **c,** Filtered locations (presence, orange) and randomly generated background locations (background, blue) within each region (red boundary). Note that in **c** background locations (blue) sampled from different regions are shown overlapping to demonstrate the extent of the species range (Extended Data Fig. 6) that was sampled from the dataset as a whole. NA, north Atlantic; SA, south Atlantic; NIO, northwest Indian Ocean; SIO, southwest Indian Ocean; EIO, east Indian Ocean; WP, west Pacific; EP, east Pacific.

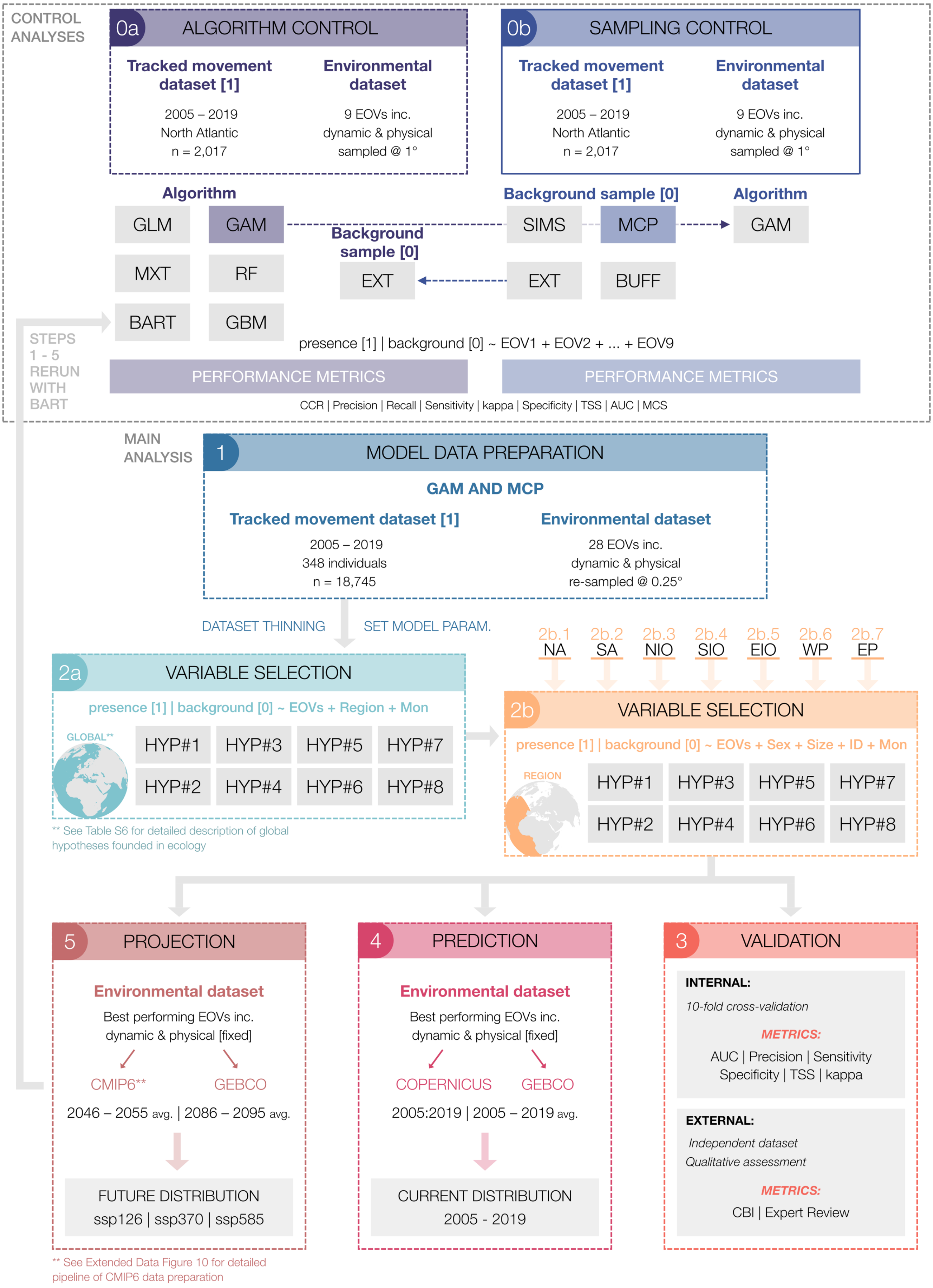


**Figure S27 | Modelling framework.** A six-step (eight sub-step) procedure was followed where sections 0a to 2a were undertaken once and steps 2b, 4 and 5 were conducted separately for each region (n = 7). Section 3 was undertaken once based on globally joined regions. Sections 4 and 5 were repeated using the second-best performing algorithm from step 0a and best performing hypothesis from 2b. The abbreviations in section 0a refer to the algorithms tested and in 0b to the background sampling methods tested (see Methods section for correspondence and abbreviation definitions).

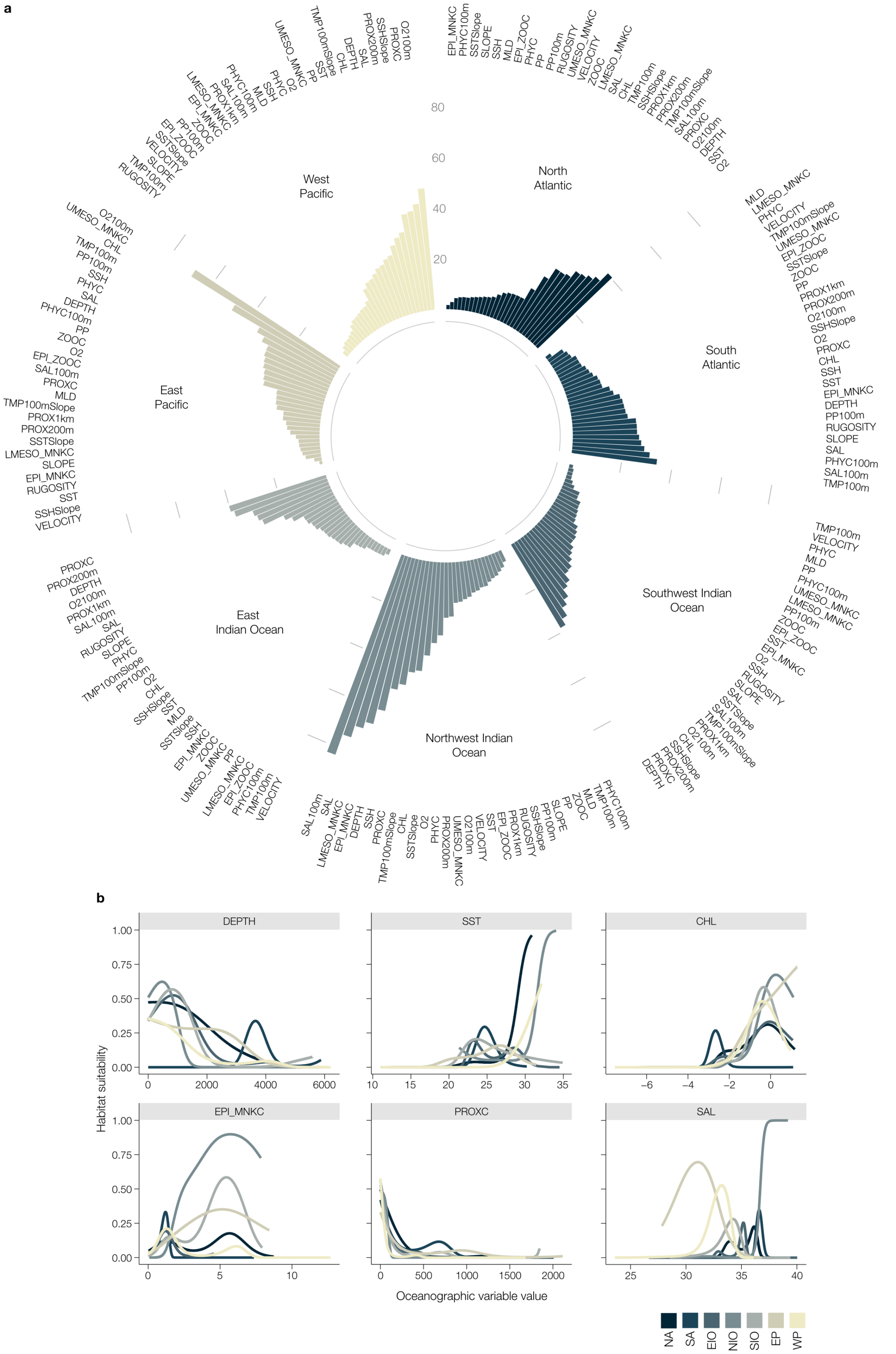


**Figure S28a | Modelled univariate performance summary. a,** Percentage of deviance explained (%dev) for each essential ocean variable (EOV) ordered by %dev and coloured by region. **b**, Whale shark habitat suitability predicted from univariate models from EOVs that were included in the best performing regional hypothesis and coloured by region. See Table S4 for full EOV names, units, and descriptions. NA, north Atlantic; SA, South Atlantic; NIO, northwest Indian Ocean; SIO, southwest Indian Ocean; EIO, east Indian Ocean; WP, west Pacific and EP, East Pacific.

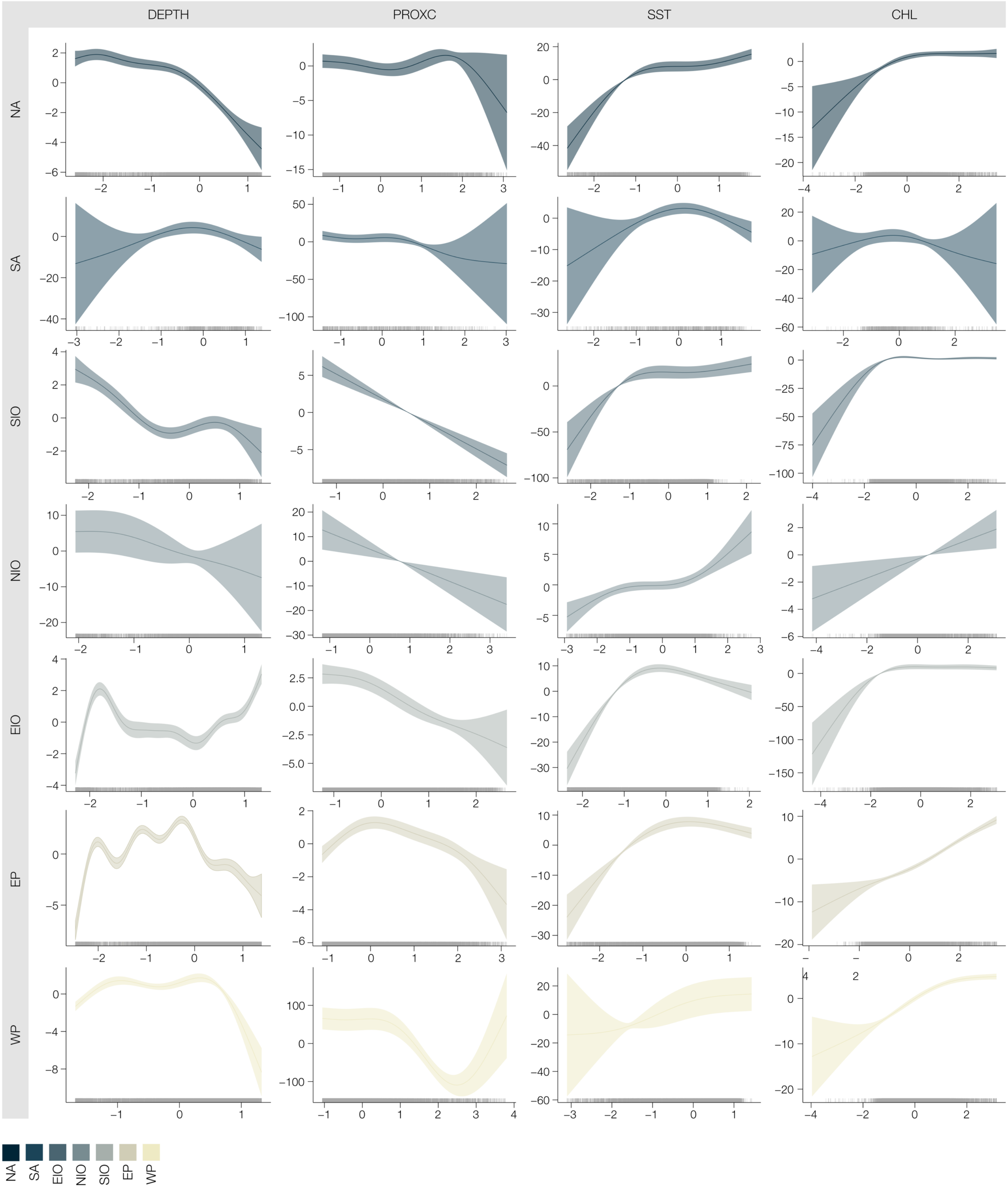


**Figure S28b | Modelled multivariate summary.** Response curves from the best performing surface hypothesis for each region with the response on the y-axis (relative scale) calculated from the multivariate models and scaled predictor variable range on the x-axis. Shaded areas represent the 95% confidence intervals. DEPTH, bathymetric depth (m); SST, sea surface temperature (°C), PROXC, proximity to coastline (km), CHL, naturally logged chlorophyll-*a* (mg m$^{-3}$). See Table S4 for full EOV names, units, and descriptions. NA, north Atlantic; SA, South Atlantic; NIO, northwest Indian Ocean; SIO, southwest Indian Ocean; EIO, east Indian Ocean; WP, west Pacific and EP, East Pacific.

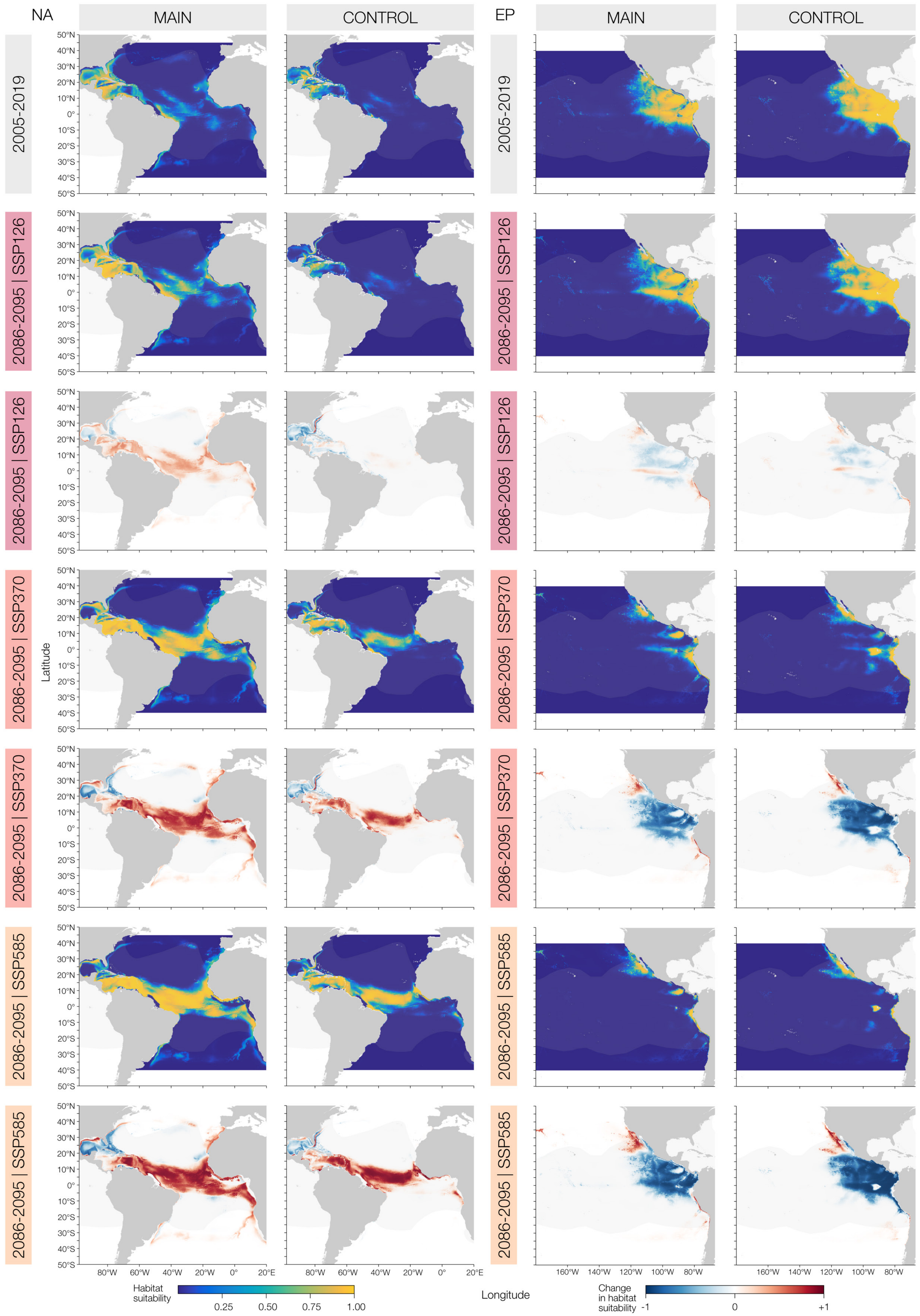

NA
MAIN
CONTROL
EP
MAIN
CONTROL
2005-2019
2086-2095 | SSP126
2086-2095 | SSP126
2086-2095 | SSP370
2086-2095 | SSP370
2086-2095 | SSP585
2086-2095 | SSP585
50°N
40°N
30°N
20°N
10°N
0°
10°S
20°S
30°S
40°S
50°S
Latitude
80°W
60°W
40°W
20°W
0°
20°E
160°W
140°W
120°W
100°W
80°W
Habitat suitability
0.25
0.50
0.75
1.00
Longitude
Change in habitat suitability
-1
0
+1

**Figure S29** | **Variable selection control.** Regions of high (yellow) and low (blue) habitat suitability in the north Atlantic (NA, columns 1 and 2) and east Pacific (EP, columns 3 and 4) predicted for the 2005 – 2019 average (row 1) and projected for each scenario from the 2086 – 2095 average (rows 2,4 and 6). Regions of increase (red), decrease (blue) and no change (white) in the NA (columns 1 and 2) and EP (columns 3 and 4) projected for each scenario from the 2086 – 2095 average (rows 3, 5 and 7). Where the first column for each region shows the mapped outputs from methods used in the main study (Generalised Additive Models) and the second column shows outputs where all non-colinear essential ocean variables were included in the model. Row headings are coloured by decade and scenario. The International Union for Conservation of Nature (IUCN) species distribution boundary is transparently overlaid on maps.

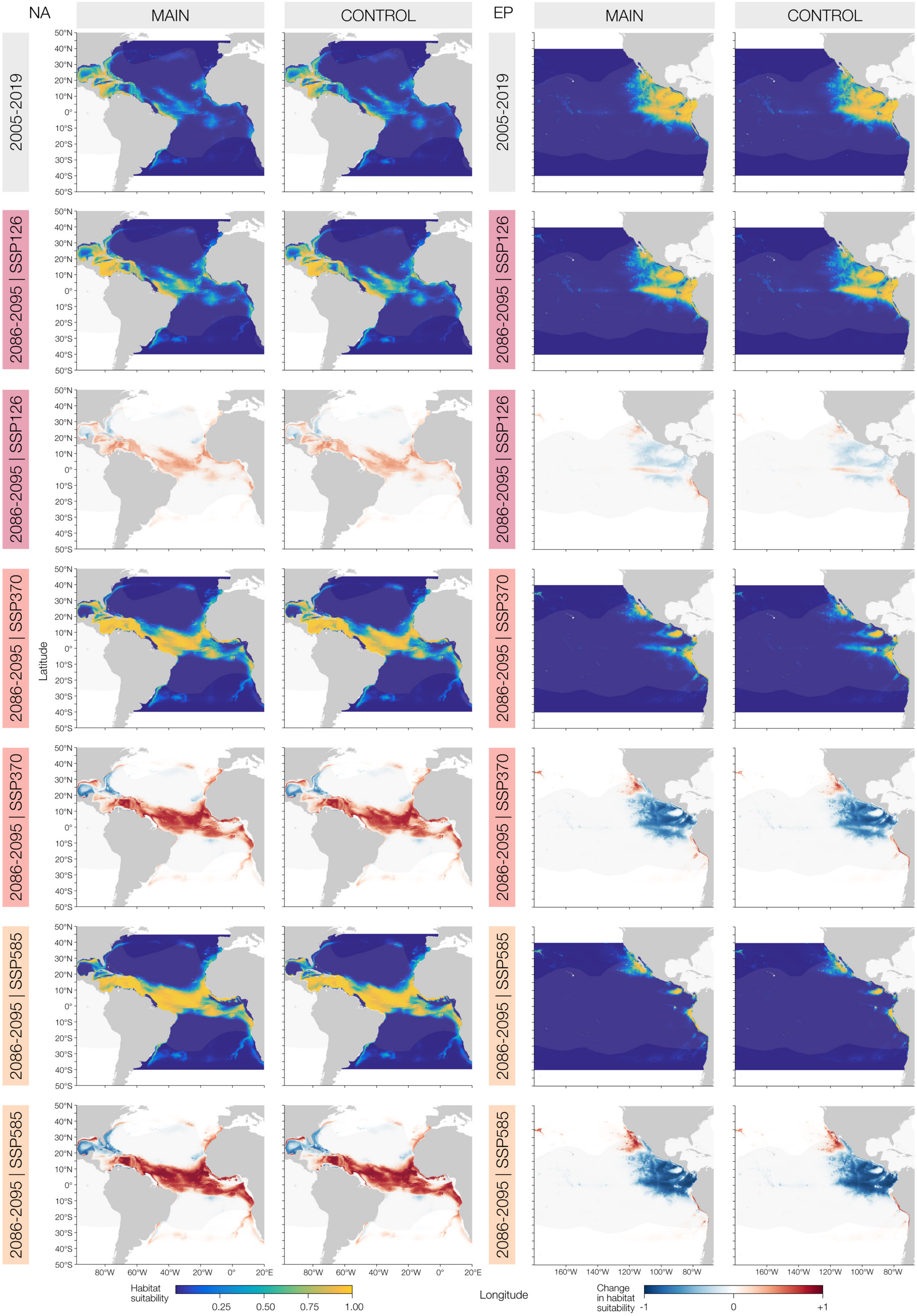

NA
MAIN
CONTROL
EP
MAIN
CONTROL
2005-2019
2086-2095 | SSP126
2086-2095 | SSP126
2086-2095 | SSP370
2086-2095 | SSP370
2086-2095 | SSP585
2086-2095 | SSP585
50°N
40°N
30°N
20°N
10°N
0°
10°S
20°S
30°S
40°S
50°S
Latitude
80°W
60°W
40°W
20°W
0°
20°E
160°W
140°W
120°W
100°W
80°W
Longitude
Habitat suitability
0.25
0.50
0.75
1.00
Change in habitat suitability
-1
0
+1

**Figure S30** | **Dynamic variable selection control.** Regions of high (yellow) and low (blue) habitat suitability in the north Atlantic (NA, columns 1 and 2) and east Pacific (EP, columns 3 and 4) predicted for the 2005 – 2019 average (row 1) and projected for each scenario from the 2086 – 2095 average (rows 2,4 and 6). Regions of increase (red), decrease (blue) and no change (white) in the NA (columns 1 and 2) and EP (columns 3 and 4) projected for each scenario from the 2086 – 2095 average (rows 3, 5 and 7). Where the first column for each region shows the mapped outputs from methods used in the main study (Generalised Additive Models) and the second column shows outputs where epipelagic micro-nekton, an essential ocean variable for which there are currently no reliable present-day predictions or future projections, was removed. Row headings are coloured by decade and scenario. The International Union for Conservation of Nature (IUCN) species distribution boundary is transparently overlaid on maps.

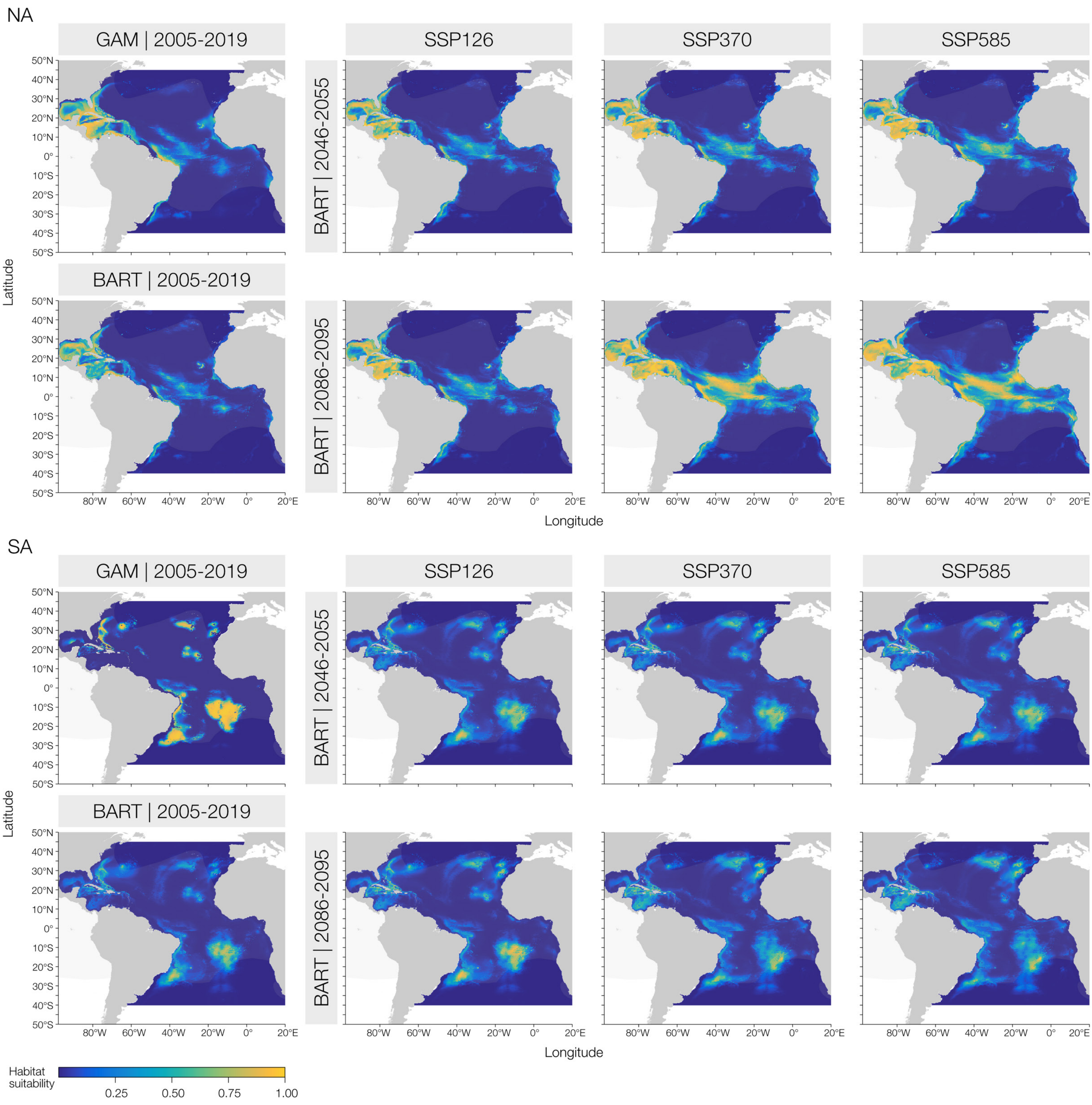


**Figure S31 | Agreement algorithm control.** Regions of high (yellow) and low (blue) habitat suitability in the north Atlantic (NA, rows 1 and 2) and south Atlantic (SA, rows 3 and 4) predicted for the 2005 – 2019 average and projected for each decade and scenario using Generalised Additive Models (GAM) and Bayesian Additive Regression Trees (BART). Where the first column and row for each region shows the mapped outputs for current GAM modelled predictions, the first column and second row show current BART modelled predictions. Columns 2 – 3 show BART modelled future projections for each scenario and decade (rows) combination. See row and column headings for map identification. The International Union for Conservation of Nature (IUCN) species distribution boundary is transparently overlaid on maps.

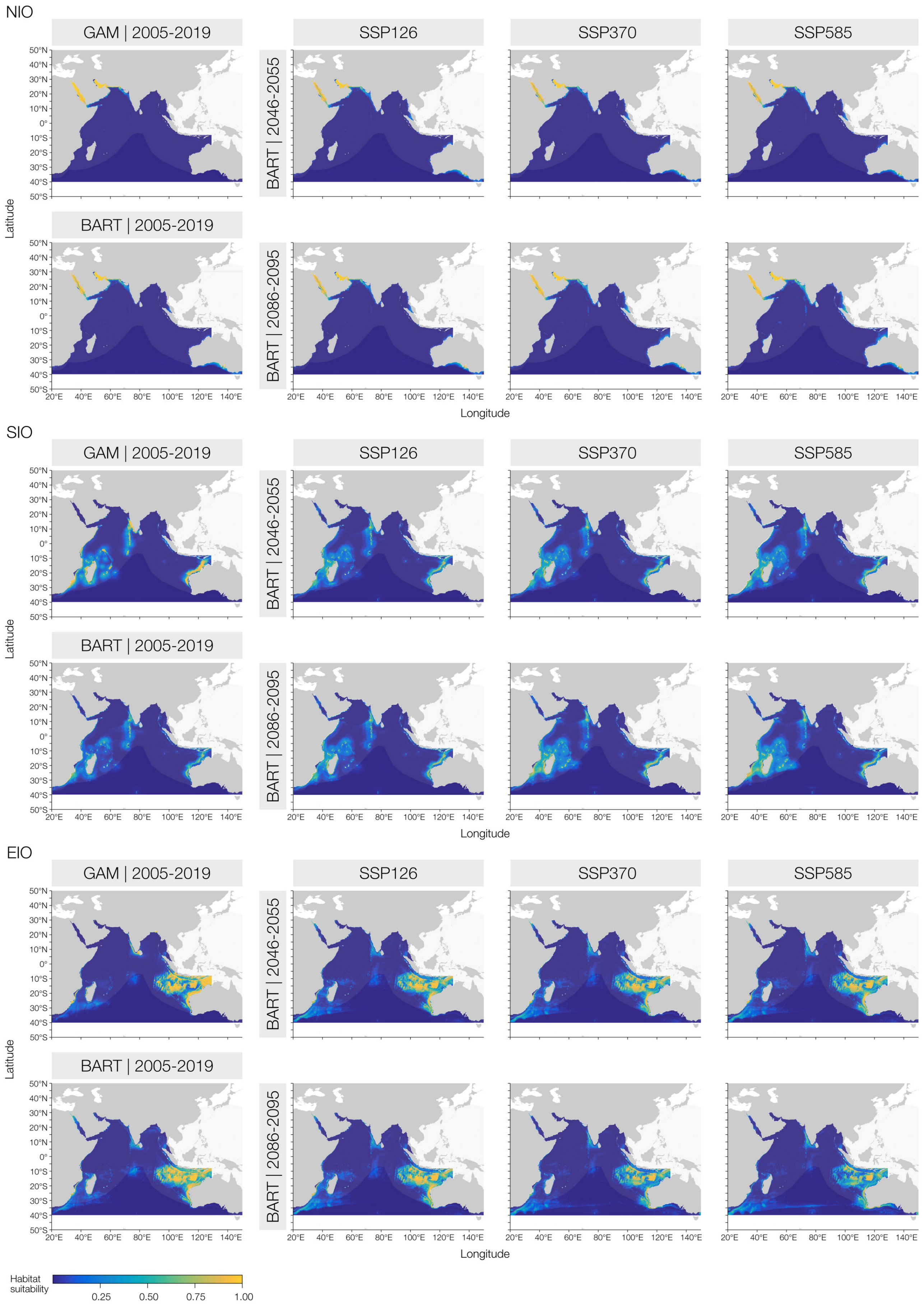


**Figure S32 | Agreement algorithm control.** Regions of high (yellow) and low (blue) habitat suitability in the northwest Indian Ocean (NIO, rows 1 and 2), southwest Indian Ocean (SIO, rows 3 and 4) and east Indian Ocean (EIO, rows 5 and 6) predicted for the 2005 – 2019 average and projected for each decade and scenario using Generalised Additive Models (GAM) and Bayesian Additive Regression Trees (BART). Where the first column and row for each region shows the mapped outputs for current GAM modelled predictions, the first column and second row show current BART modelled predictions. Columns 2 – 3 show BART modelled future projections for each scenario and decade (rows) combination. See row and column headings for map identification. The International Union for Conservation of Nature (IUCN) species distribution boundary is transparently overlaid on maps.

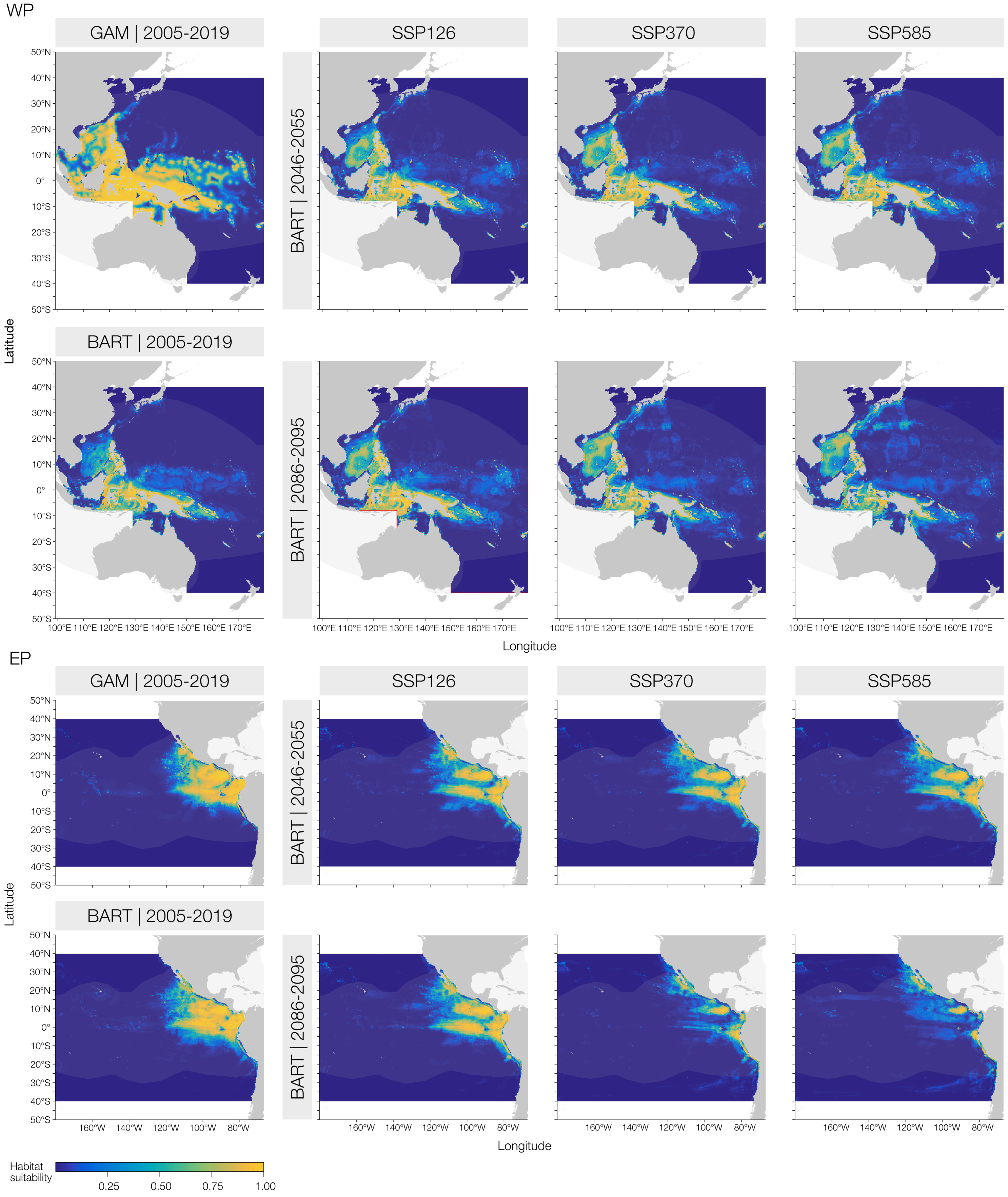


**Figure S33 | Agreement algorithm control.** Regions of high (yellow) and low (blue) habitat suitability in the west Pacific (WP, rows 1 and 2) and east Pacific (EP, rows 3 and 4) predicted for the 2005 – 2019 average and projected for each decade and scenario using Generalised Additive Models (GAM) and Bayesian Additive Regression Trees (BART). Where the first column and row for each region shows the mapped outputs for current GAM modelled predictions, the first column and second row show current BART modelled predictions. Columns 2 – 3 show BART modelled future projections for each scenario and decade (rows) combination. See row and column headings for map identification. The International Union for Conservation of Nature (IUCN) species distribution boundary is transparently overlaid on maps.

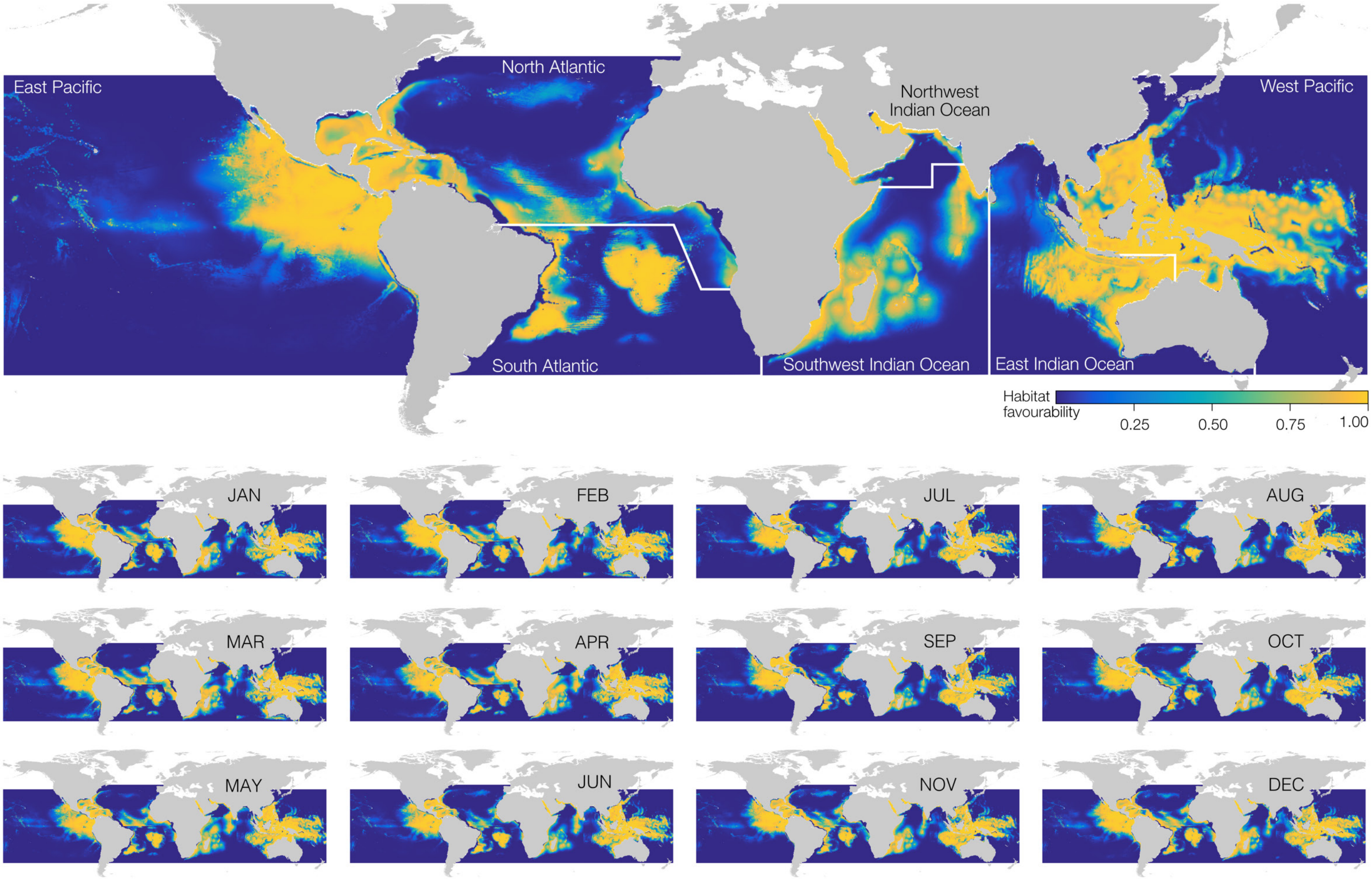


**Figure S34 | Mapped model outputs.** Regions of high (yellow) and low (blue) habitat favourability are indicated globally where study regions have been joined together at boundaries (white border) based on current climatologies (2005 – 2019) annually (top panel) and for each month in a relative year (bottom panels).

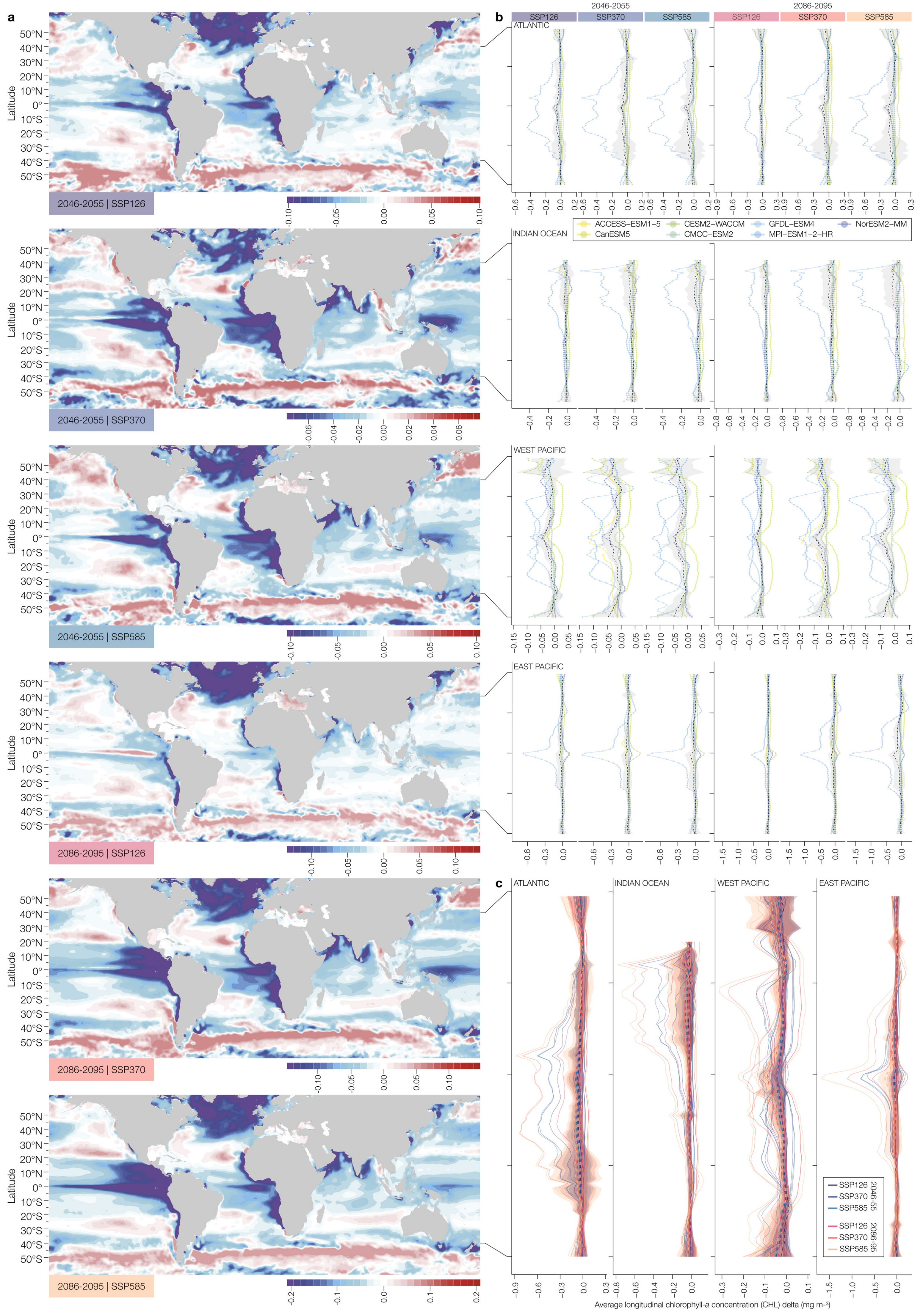

a
b
c
2046-2055 | SSP126
2046-2055 | SSP370
2046-2055 | SSP585
2086-2095 | SSP126
2086-2095 | SSP370
2086-2095 | SSP585
Latitude
2046-2055
2086-2095
SSP126
SSP370
SSP585
ATLANTIC
INDIAN OCEAN
WEST PACIFIC
EAST PACIFIC
ACCESS–ESM1–5
CanESM5
CESM2–WACCM
CMCC–ESM2
GFDL–ESM4
MPI–ESM1–2–HR
NorESM2–MM
2046-55
2086-95
Average longitudinal chlorophyll-a concentration (CHL) delta (mg m-3)

**Figure S35** | **Variable projection summary. a**, Regions of increase (red), decrease (blue) and no change (white) in chlorophyll-*a* concentration (CHL, mg $m^{-3}$) across the world as predicted by CMIP6 global climate models (GCM) coloured and labelled by decade and scenario (bottom left). **b**, Latitudinal means (between 40°N/S) coloured by unique GCMs with latitudinal ensemble mean in black and standard deviation shown in shaded grey for the Atlantic (row 1), Indian Ocean (row 2), west Pacific (row 3) and east Pacific (row 4). Column headings are coloured by decade and scenario. **c**, Values from **b** overlayed into a single plot for comparison, coloured by decade and scenario in the Atlantic (column 1), Indian Ocean (column 2), west Pacific (column 3) and east Pacific (column 4). Dotted line shows the GCM ensemble mean used to inform model projections and shaded areas are the standard deviation. See Extended Data Figure 10 for GCM method framework.

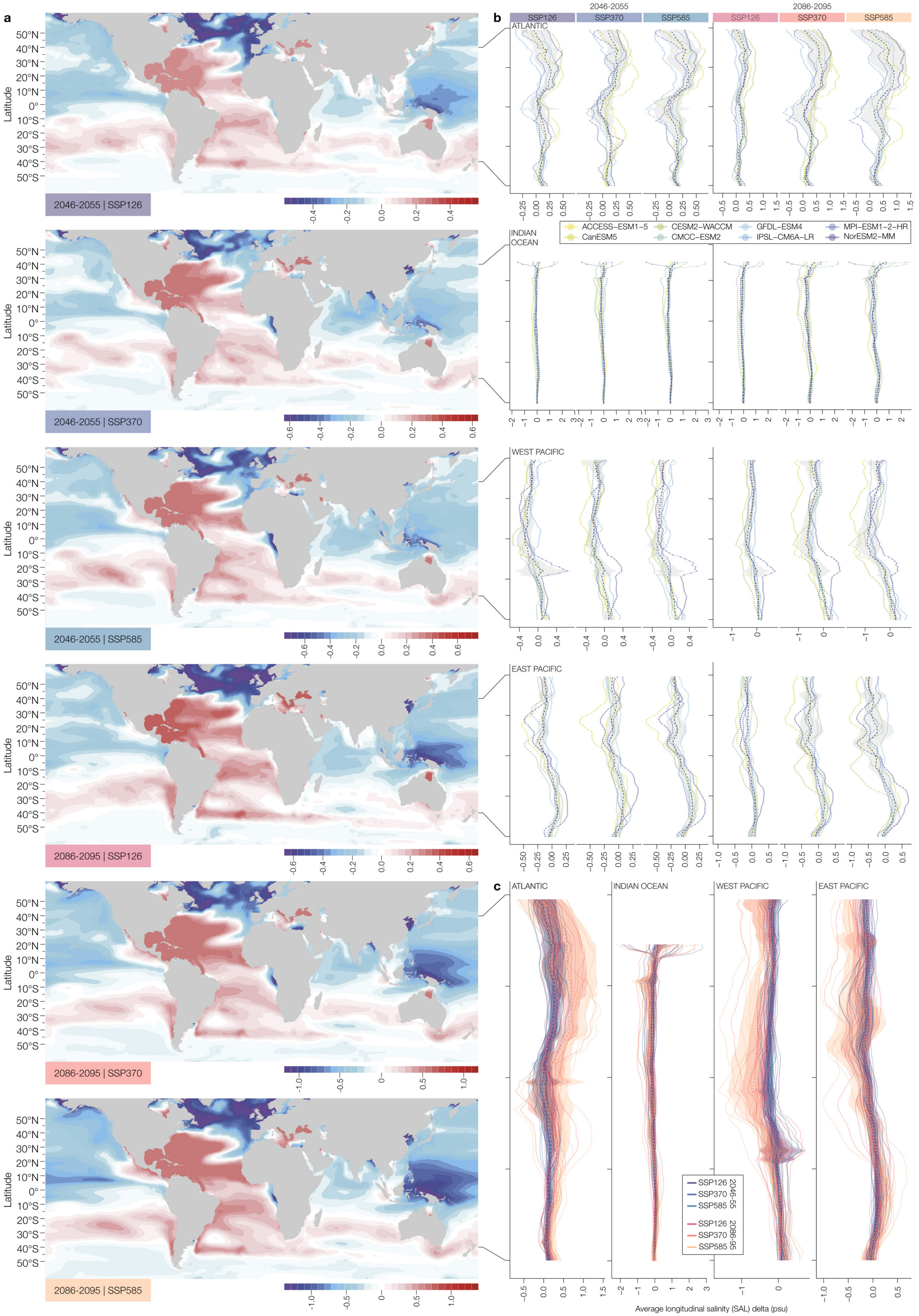

a
b
c
2046-2055
2086-2095
SSP126
SSP370
SSP585
Latitude
50°N
40°N
30°N
20°N
10°N
0°
10°S
20°S
30°S
40°S
50°S
2046-2055 | SSP126
2046-2055 | SSP370
2046-2055 | SSP585
2086-2095 | SSP126
2086-2095 | SSP370
2086-2095 | SSP585
ATLANTIC
INDIAN OCEAN
WEST PACIFIC
EAST PACIFIC
ACCESS-ESM1-5
CanESM5
CESM2-WACCM
CMCC-ESM2
GFDL-ESM4
IPSL-CM6A-LR
MPI-ESM1-2-HR
NorESM2-MM
SSP126 2046-55
SSP370
SSP585
SSP126 2086-95
SSP370
SSP585
Average longitudinal salinity (SAL) delta (psu)

**Figure S36 | Variable projection summary. a**, Regions of increase (red), decrease (blue) and no change (white) in surface salinity (SAL, psu) across the world as predicted by CMIP6 global climate models (GCM) coloured and labelled by decade and scenario (bottom left). **b**, Latitudinal means (between 40°N/S) coloured by unique GCMs with latitudinal ensemble mean in black and standard deviation shown in shaded grey for the Atlantic (row 1), Indian Ocean (row 2), west Pacific (row 3) and east Pacific (row 4). Column headings are coloured by decade and scenario. **c**, Values from **b** overlayed into a single plot for comparison, coloured by decade and scenario in the Atlantic (column 1), Indian Ocean (column 2), west Pacific (column 3) and east Pacific (column 4). Dotted line shows the GCM ensemble mean used to inform model projections and shaded areas are the standard deviation. See Extended Data Figure 10 for GCM method framework.

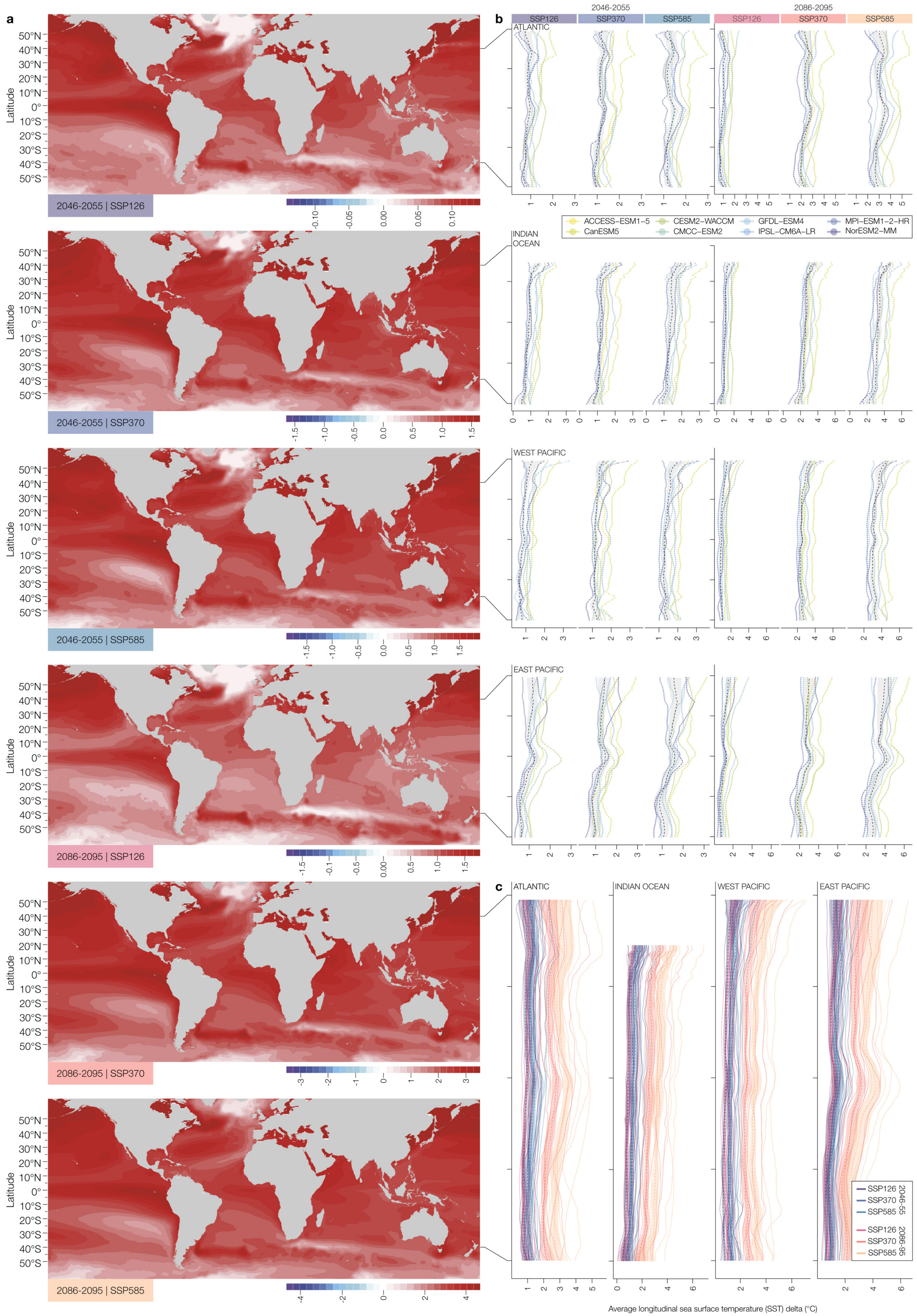

a
b
c
2046-2055 | SSP126
2046-2055 | SSP370
2046-2055 | SSP585
2086-2095 | SSP126
2086-2095 | SSP370
2086-2095 | SSP585
Latitude
50°N
40°N
30°N
20°N
10°N
0°
10°S
20°S
30°S
40°S
50°S
2046-2055
2086-2095
SSP126
SSP370
SSP585
ATLANTIC
INDIAN OCEAN
WEST PACIFIC
EAST PACIFIC
ACCESS−ESM1−5
CanESM5
CESM2−WACCM
CMCC−ESM2
GFDL−ESM4
IPSL−CM6A−LR
MPI−ESM1−2−HR
NorESM2−MM
SSP126 2046-55
SSP370
SSP585
SSP126 2086-95
SSP370
SSP585
Average longitudinal sea surface temperature (SST) delta (°C)

**Figure S37** | **Variable projection summary. a**, Regions of increase (red), decrease (blue) and no change (white) in sea surface temperature (SST, °C) across the world as predicted by CMIP6 global climate models (GCM) coloured and labelled by decade and scenario (bottom left). **b**, Latitudinal means (between 40°N/S) coloured by unique GCMs with latitudinal ensemble mean in black and standard deviation shown in shaded grey for the Atlantic (row 1), Indian Ocean (row 2), west Pacific (row 3) and east Pacific (row 4). Column headings are coloured by decade and scenario. **c**, Values from **b** overlayed into a single plot for comparison, coloured by decade and scenario in the Atlantic (column 1), Indian Ocean (column 2), west Pacific (column 3) and east Pacific (column 4). Dotted line shows the GCM ensemble mean used to inform model projections and shaded areas are the standard deviation. See Extended Data Figure 10 for GCM method framework.

## 3. Supplementary Methods

### *3.1 Oceanographic variable selection*

To characterise the biophysical environment at whale shark tracking locations and randomly generated background locations, we compiled a suite of 28 essential ocean variables (EOVs) that have previously been shown to be important drivers of habitat selection for whale sharks[10,31-36] and other oceanic shark species[37,38].

These comprised a combination of physical and dynamic oceanic conditions (Table S4), including: (i) concentration of chlorophyll-*a* in sea water (mg $m^{-3}$) (abbreviation used in models: CHL) which is often modelled as a proxy for productivity and has been shown to characterise preferred habitats of whale sharks[32-34,39]; (ii) mole concentration of dissolved molecular oxygen in sea water (mmol $m^{-3}$) at the surface (O2) and at 100m depth (O2100m), which have been shown to strongly influence pelagic shark space use[40]; (iii) net primary production of biomass, expressed as carbon per unit volume in sea water per day (g $m^{-3}$ $d^{-1}$) at the surface (PP) and at 100m depth (PP100m); (iv) mole concentration of phytoplankton, expressed as carbon in sea water (mmol $m^{-3}$) at the surface (PHYC) and at 100m depth (PHYC100m) as a direct measure of primary productivity; (v) sea water temperature (°C) at the surface (SST) which is known to influence the presence and movements of whale sharks[10,14] and many other pelagic shark species[37,41]; (vi) maximum thermal gradient (Δ°C per 0.25°) at the surface (SSTSlope) which was calculated using maximum gradient maps where the maximum difference between each grid cell and its surrounding cells was determined; (vii) sea water temperature (°C) at 100m depth (TMP100m); (viii) maximum thermal gradient (Δ°C per 0.25°) at 100m depth (TMP100mSlope) which was calculated as with SSTSlope using maximum gradient maps; (ix) geostrophic current (VELOCITY) measured as velocity (m $s^{-1}$) which has been explored as a preferred movement mode for whale sharks into and away from seasonal aggregation areas[33]; (x) sea water salinity (psu) at the surface (SAL) and at depth (SAL100m) which are important determinants of habitat use in some sharks[42]; (xi) sea-surface height above geoid in metres (m, SSH) and (xii) maximum gradient (Δm per 0.25°), calculated using maximum gradient maps (SSHSlope), which have been shown to represent important mesoscale features for oceanic sharks[43]; (xiii) ocean mixed-layer depth thickness or thermocline depth in meters (m) (MLD) which can affect the foraging behaviour of pelagic sharks[37]; (xiv) meso-zooplankton concentration in carbon (C g $m^{-2}$, ZOOC) defined as small organisms ranging from 200 µm to 2 cm consisting primarily of crustacean plankton (copepods), meroplanktonic larva and smaller individual gelatinous zooplankton that serve as a primary food source for whale sharks[44]; (xv) epipelagic meso-zooplankton concentration in carbon (C g $m^{-2}$, EPI_ZOOC) which was calculated by dividing total meso-zooplankton concentration by the dynamic epipelagic layer depth (m); (xvi) epipelagic micronekton concentration in wet weight (ww g $m^{-2}$, EPI_MNKC) classically

defined by a size range of 2 cm to 20 cm containing a large diversity of fish, crustacean, squid and gelatinous species that inhabit permanently the epipelagic layer, which may attract the species or directly comprise their prey[44]; (xvii) upper mesopelagic micronekton concentration in wet weight (ww g $m^{-2}$, UMESO_MNKC) which is the component of micronekton that inhabits permanently the upper mesopelagic layer; (xviii) lower mesopelagic micronekton concentration in wet weight (ww g $m^{-2}$, LMESO_MNKC) which is the component of the micronekton that inhabits permanently the lower mesopelagic layer; (xix) bathymetric depth in meters (m, DEPTH), (xx) bathymetric slope in meters (Δ°C per 0.25°, SLOPE) which was calculated as the maximum slope from surrounding cells; (xxi) bathymetric rugosity (m, RUGOSITY) which was calculated as standard deviation of surrounding cells; (xxii) distance from coastline (km, PROXC); (xxiii) to the 200m isobath (km, PROX200m) and (xxiv) the 1km isobath (km, PROX1km), features which have all been shown to contribute to whale shark aggregation area suitability[31].

Environmental dataset (i) was downloaded from ACRI-ST GlobColour dataset (HERMES; https://hermes.acri.fr). Environmental datasets (ii) – (iv) were downloaded from Copernicus Marine Environment Monitoring Service (CMEMS; https://marine.copernicus.eu) Global Ocean Biochemistry Hindcast product, datasets (v) – (vi) from CMEMS Global Ocean Ostia Sea Surface Temperature And Sea Ice Reprocessed, datasets (vii) – (xiii) from CMEMS Global Ocean Physics Reanalysis, and datasets (xiv) – (xviii) from CMEMS Global Ocean Low And Mid Trophic Levels Biomass Content Hindcast product. The physical datasets (xix) – (xxiv) were downloaded from General Bathymetric Chart of the Oceans (GEBCO; https://www.gebco.net). HERMES and CMEMS data were available from the years of 2005 to 2019 as weekly and monthly datasets and from resolutions between 0.0083° and 0.25° (120 and 4 cells per degree, respectively; Table S4).

All EOVs were standardized (mean-centred and divided by the standard deviation, s.d.) and collinearity between variables was checked during hypothesis development and before each model run to determine appropriate inclusion in the models. A correlation matrix was used and any variable combinations with a correlation coefficient above 0.75 were not included in the same hypothesis. Because CHL values were highly skewed they were logged before standardisation. Since different sized sharks were tracked in this study, we included size in the models as a random effect based on the median total length (TL) of all individuals (median TL 6.5 m, ± 2.13 s.d.). Individuals with a TL ≤6.5 m were classified as small and those with a TL >6.5 m as large.

### *3.2 Hypothesis formation*

A fundamental first step for our modelling framework lies in articulating a reasonable set of competing hypotheses to accurately identify features that underpin whale shark distributions[45], which are inherently linked to energetic, physiological and reproductive constraints that can influence animal behavioural responses[46,47]. In common with other highly mobile marine

megafauna, whale shark horizontal spatial ecology is governed by scale-dependent interactions between intrinsic and extrinsic processes. For instance, movements may be restricted to temperature ranges that suit specific bioenergetic strategies, perhaps allowing individuals to take advantage of thermal regimes to increase foraging and metabolic efficiency[48,49]. The movements of a whale shark tracked in the region of the Banda Sea, Indian Ocean, were correlated with water temperatures, with the animal following frontal systems while remaining in a sea surface temperature (SST) range of 24 – 29 °C[14]. As an ectothermic species, remaining within regions with warm surface waters may be linked to thermoregulation, where extended periods can be spent at the surface to rewarm, thus maintaining optimal body temperatures after experiencing cooler conditions while searching for prey and foraging at depth[14]. This has been widely observed in species including ocean sunfish (*Mola mola*)[47], swordfish (*Xiphias gladius*)[50], blue shark (*Prionace glauca*)[51] and bluefin tuna (*Thunnus thynnus*)[52]. At broad scales, SST can shift seasonally, and was shown to be the primary driver of the observed cyclical season of whale shark occurrence probability in the Indian Ocean[34]. It was also shown to be a key driver behind potentially shifting distributions under future warming scenarios where range expansion and core habitat contraction was linked to SST, among other shifting EOVs[10].

However, the large body size of whale sharks does provide some temperature stability allowing for movement through highly variable thermal environments, and with the lowest whole-body heat-transfer coefficient of any fish species measured[53], SST may not be the most important driver for whale sharks when considered alongside other key EOVs. For example, predictable and often seasonal prey availability is one of the leading hypotheses behind why large numbers of individuals aggregate in coastal sites around the world[54,55]. Whale sharks filter feed on zooplankton and micro-nektonic prey[44] which can form dense patches throughout the generally surface prey sparse tropics[56]. To locate and exploit prey patches which are highly variable in space and time within their generally less productive niche, whale sharks employ a number of strategies, including extensive searching behaviour patterns[57] and return movements to predictable feeding hotspots at finer scales[44]. At broad horizontal scales, these strategies can be observed in movements of individuals where clusters of activity interspersed by long steps in tracking data may reflect feeding bouts centred on fluid features such as eddies and fronts[36].

Oceanic frontal and upwelling regions comprised of steep thermal gradients can aggregate prey and have been identified as a key driver for many marine megafauna species due to improved foraging efficiency and opportunities[37,58]. Spatial relationships between these dynamic ocean features, along with CHL concentration – which acts as a proxy for phytoplankton biomass and primary productivity – provide support for prey driven movement and distribution hypotheses[32,41,59]. CHL and distance to the continental shelf edge were demonstrated to be among the strongest predictors of whale shark sightings in the Gulf of Mexico, which was indeed explained by the

association of these variables with high prey availability[32]. However, when explored globally there was no evidence that CHL contributed to suitability predictions for whale sharks[10], which was explained by the potential broad spatial scale of the investigation or possible spatiotemporal lags between primary production and secondary consumers[10]. Whale shark foraging strategy may also include non-feeding periods: there are likely to be periods when whale sharks are not able to access prey, and wild sharks may exhibit behaviours observed for captive individuals that appear to fast by foregoing feeding opportunities[60]. During movements throughout sparse prey areas, bathymetric features such as seamounts or continental shelves may provide whale sharks with navigational markers[61], as well as potentially providing increased foraging opportunities at depth. Depth was an important predictor in the Pacific and in the Atlantic when explored globally, but not in the Indian Ocean[10]. Bathymetric features have also been shown to define sites where the species aggregates, through their influence on upwelling events, primary productivity and prey availability[31].

### *3.3 Algorithm selection control*

To control for algorithm selection, a simplified dataset was built whereby nine non-collinear EOVs (bathymetry, DEPTH; surface chlorophyll-*a* concentration, CHL; mixed layer depth, MLD; epipelagic micro-nekton, EPI_MNKC; surface salinity, SAL; sea surface height, SSH; sea surface temperature, SST; sea surface temperature gradient, SSTSlope; and geostatic velocity, UV; see Table S4 for EOV descriptions and units) were extracted from whale shark presence and background locations within the north Atlantic region using the fuzzySim package in R[62]. Here, averaged EOVs (2005 – 2019) within each background (sampled within the entire Atlantic, excluding presence cells) and presence (n = 5,375) location were sampled at 1° resolution (Extended Data Fig. 9a). To find the model that performed the highest when predicting presence of the north Atlantic whale sharks from this simplified dataset, six algorithms were tested: generalised linear models, GLM; Generalised Additive Models, GAM; Maximum Entropy, MXT; Random Forest, RF; Boosted Regression Trees/Generalised Boosted Models, GBM; and Bayesian Additive Regression Trees, BART. These six algorithms were run with the presence of the whale sharks (1: presence versus 0: background) as a response variable using the stats (GLM), gam (GAM), maxent (MXT), randomForest (RF), gbm (GBM) and embarcadero (BART) packages in R[63-67]. Model comparison based on internal validation was measured by accuracy (correct classification rate, CCR), precision (positive prediction rate), sensitivity/recall (true positive rate), Cohen’s kappa (correct classification over chance, kappa), specificity (true negative rate) and true skill statistic (balance between sensitivity and specificity, TSS)[68,69], where the chosen threshold was set to the maximum TSS. For model comparison based on cross-validation the study region was split into 300 km blocks and data within each assigned to one of 5 folds[70]. The area under the receiver operator characteristic curve (AUC), TSS and Miller Calibration line (the intercept and slope of the regression of the response variable on the logit of predicted probabilities, MCS) were

measured on modelled data from each fold before being averaged across folds. Model selection was mainly based on the above performance metrics and also on a visual inspection of the prediction maps from each algorithm (Extended Data Fig. 9b – d). GAMs were determined to be the most appropriate model based on our whale shark tracking dataset (see Extended Data Figure 9 legend for description of results).

### *3.4 Background sampling control*

The best performing algorithm (GAM) was then applied in a similar framework to control for and measure the performance of different background sampling methods. Four methods of sampling were tested: simulated background tracks with a presence background (PB) ratio of 1:10 (here termed SIMS), randomly sampled background locations from outside the minimum convex polygon (MCP) of all presence locations within each region cropped to known latitudinal limits (PB ratio 1:10, MCP), gridded background locations sampled from within the entire study extent (EXT), and gridded background locations sampled from within a buffer (mean pairwise distance between presence locations, BUFF) (Extended Data Fig. 9e). Presence locations sampled in the SIMS (n = 13,658 presence and background locations) and MCP (n = 22,187) methods were based on the actual tracked movements of individuals where nine EOVs (same nine used in the algorithm control) were sampled from the closest 1° cell using the fuzzySim package[62]. The EXT (n = 5,375) and BUFF (n = 2,016) methods were simplified and gridded with a single presence location within each 1° cell (which may have contained more than one actual presence record). Background sampling selection was mainly based on the performance metrics used in the algorithm selection framework but also on a visual inspection of the prediction maps from each method (Extended Data Fig. 9e – h). The MCP approach was determined to be the most appropriate model based on our whale shark tracking dataset (see Extended Data Figure 9 legend for description of results).

The MCP method was developed with the aims of (i) minimising spatial overlap between presences and generated background locations such that replication of locations was avoided (since this can lead to contradictory information when used in a binomial model, i.e. providing the same locations simultaneously as both presence and background)[71]; (ii) sampling environments from within the entire accessible range of whale sharks to capture the oceanographic variable correlations that represent whale sharks' presence in an area thus enabling broad scale current predictions and future projections; and, (iii) capturing essential life-history characteristics associated with the species' habitat selection. In practice, although the MCP method performed the best statistically, this was to be expected since the background locations were likely to be the most dissimilar to presences – with the highest degree of environmental separation – from the methods tested[71,72]. Generally speaking, this can be a concern when modelling species distributions because high model performance metrics do not always result in high levels of biological realism[71]. Given this

caveat we took careful steps to test the biological realism of the MCP method both qualitatively and quantitatively. We found that (i) because whale sharks move widely and use large areas of the ocean yet have a tendency to aggregate in small, localised areas[31], the MCP (background sampling) and GAM (algorithm) combination was able to capture this important aspect of the species' life history by clustering habitats with steep suitability gradients (Extended Data Fig. 1a). This enabled us to (ii) generate a first order global approximation of habitat suitability for the whale shark that included localised habitat nuance as opposed to producing large swathes of habitat as can be seen in some of the other methods tested (Extended Data Fig. 9). Importantly, models achieved this whilst (iii) retaining the ability to predict into new environments (i.e. present-day extrapolation across ocean basins with high biological realism).

There are several key drawbacks and trade-offs to this approach that merit consideration for future studies. Firstly, in terms of the analysis structure, this approach may only be suitable for broad modelling at the ocean basin or larger scale, as it may not otherwise provide enough space from which to sample background locations when applied in a local study site context. In addition, this method of sampling incorporates no information or assumptions regarding characteristics of animal movement, such as direction of movement or distance travelled, and therefore represents the entire geographic space potentially available to the species independent of the underlying movement strategies. This means that, again, models are most appropriately applied to broad, regional and/or global scales, and would perhaps mask some finer scale habitat selection nuance that can be better captured with animal track simulations, for example. We found this method to be well suited to a species which aggregates since movements in and around the same sites were represented well in the structure of the data. Future modelling studies should select background sampling methods on a case-by-case basis considering factors such as (i) the scale of the analysis, (ii) the life-history of the study species and (iii) the planned extrapolation of model coefficients. In all cases, biological realism should be rigorously checked with externally derived information on animal occurrence.

### *3.5 Predictions of future distributions*

Data from the projected EOVs were extracted on a monthly basis following a Global Climate Model (GCM) framework (Extended Data Fig. 10 and Fig. S27). To integrate across annual variability and maximize the climate signal, decadal monthly forecasts of expected change were generated for 2050 (average for 2046 – 2055) and 2090 (referred to in main text as 2100, average for 2086 – 2095) relative to a baseline (average for 1993 – 2012) using climate change forecasts from GCMs in the Coupled Model Intercomparison Project Phase 6 (CMIP6, https://esgf-node.llnl.gov/projects/cmip6/)[73,74]. Because there are large uncertainties in climate projections, a minimum of 5 (maximum 8) ESMs were considered for each EOV to reflect the range of potential outcomes, involving a combination of: ACCESS-ESM1-5, CanESM5, CESM2-WACCM, CMCC-

ESM2, GFDL-ESM4, IPSL-CM6A-LR, MPI-ESM1-2-HR and NorESM2-MM (terminology follows CMIP6 controlled vocabularies; https://pcmdi.llnl.gov/CMIP6/Guide/dataUsers.html). We used model projections forced by three Shared Socioeconomic Pathway (SSP) scenarios, each of which represents a different future socio-economic projection and political environment[73,75]: SSP1-2.6 (referred to throughout as ssp126), SSP3-7.0 (ssp370) and SSP5-8.5 (ssp585). Ssp126, which represents the 'Sustainability' socio-economic family, assumes a nominal radiative forcing level of 2.6 $Wm^{-2}$ is reached at the end of this century, and, of the scenarios chosen here, reflects most closely the 1.5 °C warming target under the Paris Agreement. Ssp370 is a medium-high reference scenario within the 'Regional Rivalry' socio-economic family which was chosen here to represent a middle of the road forecast based on $CO_2$ emissions by the end of the century. Ssp585, which marks the upper edge of the SSP scenario spectrum, represents a high reference scenario (radiative forcing level of 8.5 $Wm^{-2}$ by 2100) in a high 'Fossil-fuel Development' world throughout the 21st century. For each SSP scenario, decadal averages were generated across ESM variable projections:

$$mVAR_t = \frac{\sum_{yr=2046}^{2055} mVAR_{yr}}{10} \qquad or \qquad mVAR_t = \frac{\sum_{yr=2086}^{2095} mVAR_{yr}}{10}$$

where $mVAR_t$ is the raw gridded variable values in decadal average $t$ representing the projected forecast. For each decadal forecast we then applied the 'Delta' method to correct the bias and variation apparent in different ESMs and to examine areas of greatest change over the next century[76,77]. First, contemporary observed climatologies were calculated:

$$bVAR_o = \frac{\sum_{yr=1993}^{2012} bVAR_{yr}}{20}$$

where $bVAR_o$ is the standardised 20-year climatology for each variable from gridded baseline data (see Table S4 for data sources). Then, monthly deltas were calculated by subtracting the historical modelled baseline (hindcast) from the projected forecast ($mVAR_t$). Deltas were then added to contemporary observed climatologies that were standardised across all ESMs ($bVAR_o$):

$$pVAR_t = \left( mVAR_t - \frac{\sum_{yr=1993}^{2012} mVAR_{yr}}{20} \right) + bVAR_o$$

where $pVAR_t$ is the bias-corrected variable projection for $t$ decadal average generated by a given ESM, $mVAR_t$ is the raw variable values in decadal average $t$, and $bVAR_o$ is the standardised

climatology. Bias-corrected variable projections ($pVAR_t$) were uniformly formatted to match global coordinate reference systems and units before being interpolated onto a 0.25° × 0.25° global grid using a bilinear interpolation method. Global uniform grids for each variable were then generated by averaging the projections from each ESM into a mean climate ensemble for each decadal SSP:

$$eVAR_t = \frac{\sum_{i=1}^{n} pVAR_{ti}}{n}$$

where $eVAR_t$ is the variable ensemble for $t$ decadal average, $pVAR_t$ is the bias-corrected variable projection for $t$ decadal average generated by a given ESM, $n$ is the total number of ESM projections for a given variable and decadal SSP combination which is limited to a minimum of 5. $eVAR_t$ maps were standardized based on the mean and s.d. from the model training data (mean-centred and divided by the s.d.).

## 4. Supplementary Results and Discussion

### *4.1 Whale shark tracking dataset*

We analysed 348 tracks from whale sharks covering an area of approximately 4 million km$^2$ (area calculations here and throughout based on a 0.25 × 0.25° standardised grid corrected for latitude). Observed tracked locations within each region were well distributed in space where individuals demonstrated a range of horizontal movement patterns, including localised coastal and extensive offshore movements up to 20,000 km distance travelled (mean 1634.29 km, median 1015.13 km) (Fig. S26). The daily movement distance (global mean 19.27 km d$^{-1}$, ± 17.68 s.d.) and the step length between locations (global mean 30.64 km, ± 83.77 s.d.) varied on a region basis (Table S5). After undertaking the data thinning procedure to remove consecutive daily locations, the step length (global mean 56.48 km, ± 120.15 s.d.) between locations was more than double the daily movement distance globally and across the regional subsets (Table S5) and greater than the spatial resolution of the environmental datasets used (0.25 × 0.25° resolution) in all regions, confirming the reduction of spatial and temporal autocorrelation within the dataset[102]. Observed tracking presence data was also distributed relatively evenly across the months with a slight peak from July to September (32%) and the least representation from February to April (18%), with the south Atlantic and southwest Indian Ocean containing months where no species occurrences were available (south Atlantic, September – December, southwest Indian Ocean, June; Table S5).

### *4.2 Model performance and oceanographic variable relationships*

After testing model performance based on tracking data spanning 2005 – 2019, GAMs were chosen as the main algorithm for the analysis. Predictor variables differed in their ranked importance for whale sharks across regions determined from GAM outputs. Bathymetric depth (DEPTH, mean 35.42% ± 12.28 s.d.), dissolved oxygen at 100m depth (O2100m, mean 33.77%, ± 19.15 s.d.), proximity to coastline (PROXC, 33.64%, ± 12.29 s.d.), salinity at 100m depth (SAL100m, mean 30.39%, ± 24.34 s.d.) and at the surface (SAL, mean 29.82%, ± 21.02 s.d.), proximity to the 200m isobath (PROX200m, mean 26.64%, ± 11.06 s.d.), chlorophyll-*a* (CHL, mean 25.29, ± 11.74 s.d.), temperature gradient at 100m depth (TMP100mSlope, mean 22.24%, ± 11.90 s.d.), dissolved oxygen at the surface (O2, mean 21.92%, ± 10.54 s.d.) and sea surface height (SSH, mean 21.93, ± 15.74 s.d.) were often the most important predictors of habitat suitability, explaining over 20% of the univariate modelled deviance, on average, across regions (Fig. S28). The best performing model from the pooled global dataset explained 57.1% of the deviance ($r^2$ = 0.54) and contained the above (excluding those which were collinear, PROX200m [with PROXC] and O2 [with sea surface temperature, SST]) and additional variables that were selected based on ecologically relevant hypotheses (Table S8). Variables included in this model were further subdivided to

generate regional hypotheses for surface and sub-surface sets (including and excluding subsurface variables, respectively, Table S9). The best performing surface model in all regions was hypothesis #8 which contained the variables DEPTH, SAL, PROXC, CHL, SST and epipelagic micro-nekton (EPI_MNKC) and explained 80.76% (± 7.89 s.d.) of the deviance on average (Table S9). Within the best performing surface model and univariate models, variable suitability and fitted response curves differed among regions (Fig. S28).

The best performing surface models were used to estimate spatial habitat suitability informed by new data from averaged EOV layers (2005 – 2019 mean of all EOVs that were included in the hypothesis) to generate a global map that was interpreted as a measure of habitat suitability (Extended Data Fig. 1a). Here, 34,309 grid cells (0.25 × 0.25° resolution) were defined as ecologically important global core habitats (core habitats defined as the top 90$^{th}$ percentile of global habitat suitability; 0.69 habitat suitability in the present day) for whale sharks, which equated to a total area of approximately 26 million km$^2$. Over 50% of cumulative habitat suitability occurred within 10 degrees north and south of the Equator (50.56% ± 10°, 84.17% ± 20°, 98.98% ± 30°, Extended Data Fig. 1a) and between 100 degrees east and west of the international date line (180° longitude) (51.55% ± 100°, 69.89%, ± 80°, 77.61%, ± 60°, Extended Data Fig. 1a).

### *4.3 Model validation*

We evaluated the performance of the GAM models used in the main analysis. Here, internal tenfold cross-validation showed good overall performance with a mean area under the receiver operating characteristic curve (AUC) score of 0.99 (± 0.005 s.d.) across regions (Table S10). Model accuracy varied between 0.92 (± 0.01 s.d., southwest Indian Ocean) and 0.99 (± 0.003 s.d., northwest Indian Ocean), precision between 0.55 (± 0.05 s.d., southwest Indian Ocean) and 0.90 (± 0.03 s.d., northwest Indian Ocean), sensitivity between 0.95 (± 0.04 s.d., south Atlantic) and 0.99 (± 0.009 s.d., northwest Indian Ocean), and specificity between 0.92 (± 0.01 s.d., southwest Indian Ocean) and 0.99 (± 0.003 s.d., northwest Indian Ocean). True skill statistic (TSS) scores ranged between 0.87 (± 0.03 s.d., southwest Indian Ocean) and 0.98 (± 0.01 s.d., northwest Indian Ocean) and kappa between 0.65 (± 0.04 s.d., southwest Indian Ocean) and 0.93 (± 0.02 s.d., northwest Indian Ocean) (Table S10). In addition, globally mapped habitat suitability was externally validated by comparison with whale shark sightings data from the Ocean Biodiversity Information System (OBIS, n = 9,379) and Sharkbook.ai (n = 13,267). Here, regional predictions were stitched together to create global model predictions. The continuous Boyce index (CBI) values of this global map were 0.95 and 0.73, respectively (Extended Data Fig. 1a – c).

A qualitative biological realism assessment confirmed that whale sharks have been sighted in validation sub-regions, which were specifically selected to be geographically far removed from the tagging and tracking regions used to train the model (Extended Data Fig. 1d). Whale shark sighting

records included a combination of anecdotal news reports and videos, published scientific literature and personal communication with experts in the field. In many cases modelled monthly suitability peaks (where habitat suitability was higher than the annual average) were matched by known seasonal peaks, which supports model realism. For example, our models predicted presence in the Azores Archipelago (Portugal) which is situated in the mid-North Atlantic at approximately 38°N, >10,000 km away from the tracked whale shark movements that were used to train the model. Here, above average habitat suitability was identified from July to November, peaking in July and October (Extended Data Fig. 1e). This prediction corroborates known occurrence of the species in the area which has been shown to coincide with the tuna season extending from May to November (Table S11). Locally, whale shark detections have been linked to SST and bathymetric features such as DEPTH and CHL[3], all of which were included in the best performing model. In the Indian Ocean our model identified the coastline of Gujarat (India) as an area of habitat suitability peaking from March to August (Extended Data Fig. 1e). Whale sharks have long been known to occur in these waters and a targeted fishery existed in the 1990s before protection measures were implemented[13] (Table S11). Peak fishery season (March to June)[12] closely matched model predictions despite the tracking data used to form these predictions being almost entirely situated within the Red Sea and Gulf of Arabia, which suggests the population sampled in the northwest Indian Ocean extrapolated well across the entire region. Although our model predicted low likelihood of whale sharks occurring in the oceanic waters of the Arabian Sea, individuals tracked in Gujarat have been shown to travel east into offshore waters[14]. Habitat suitability was also predicted around several small island nations such as Fiji and Vanuatu in the Pacific, which shared similar suitability peaks from January to May (Extended Data Fig. 1e). With limited dedicated whale shark research apparent at either location, occurrence records were obtained from social media (e.g. YouTube, Table S11). In Vanuatu, a whale shark was videoed by divers in September, which is outside of the models predicted habitat suitability peak, and although several whale shark bycatch events have been identified within the Exclusive Economic Zone (EEZ), no dates were provided making it challenging to verify seasonality in this case (Table S11).

### *4.4 Sensitivity analyses*

Variable and algorithm selection can potentially affect the spatial patterns of habitat suitability that we explored both in the present day and in future. However, we undertook an environment-variable control analysis and found that broad patterns of habitat shift – i.e. regions of habitat loss or gain in future – remained consistent irrespective of whether we included variables selected using a hypothesis-driven framework or including all that were non-collinear (Fig. S29). Patterns of habitat shift also remained consistent when we removed epipelagic micro-nekton from the models due to the uncertainty in present day estimates of low-trophic-level biomass and distribution (Fig. S30). To control for algorithm selection, we quantitively compared a range of models and ran both

Generalised Additive Models (GAM) and Bayesian Additive Regression Trees (BART) to explore regions of expected change identified by both algorithms, referred to as 'model agreement' areas. We also checked consistency in our results with an environment-envelope algorithm from AquaMaps (www.aquamaps.org) based on our tracking data and independent occurrence records to visually compare projections in the north Atlantic. Both approaches showed similar patterns of habitat shift in equatorial waters that we determined from GAMs (Extended Data Fig. 2).

### *4.5 Current whale shark habitats*

In the north Atlantic, ~2.3 million km$^2$ of the region was identified as core habitat by the model (Extended Data Fig. 1f). Core habitat within the Gulf of Mexico and Caribbean Sea were located around continental shelves including the Bay of Campeche, Campeche Bank, Yucatán Channel, Straits of Florida, Nicaraguan Rise, Cayman Ridge, Bonaire Basin and the Aves Ridge (Extended Data Fig. 1a, f). Habitat suitability was lower within the deeper waters of the Mexico, Yucatán, Cayman, Colombian and Venezuelan Basins. A band of important habitat extended north across the Blake Plateau following the Gulf Stream to a maximum northern latitude of approximately 40°N level with New Jersey, USA. Oceanic waters around this latitude were identified as less important, with predicted habitats located around bathymetric ocean features such as the New England and Corner Rise seamounts. In the central north Atlantic, the mid-Atlantic ridge showed importance in the region of the Pico, Hayes and Oceanographer Fracture Zones. The model showed that the farther north Kurchatov Fracture zone and Azorean archipelago were important from July to November with a peak in October around the central islands in the Azores. Around the same latitude and timeframe the model identified habitats farther east in the shelf waters of Portugal including the Nazaré Canyon, Estremadura Prontory and waters off Cabo São Vincente. Further south, the nearshore waters of West Africa were important up to the latitude of the Canary Islands from December to April, with the more southerly Cape Verde Plateau and Islands important across all months with hotspots south of the islands of Maio and Boa Vista. The waters off Guinea-Bissau at the shelf edge and region of the Guinea Terrace close to the Nadir Seamount, and the shelf edge near Grand Cess Canyon and Cape Palmas Seamount in Liberian waters were also identified as among the most important in the NA. Within the Gulf of Guinea, the Cote d'Ivoire Escarpment, Bight of Benin and Niger Fan were identified as important habitat areas by the model. At the same latitude, habitats in oceanic waters in the central Atlantic were predicted around the mid-Atlantic ridge in the region of the St. Peter and St. Paul fracture zones extending north to the Fifteen–Twenty fracture zone. Nearshore waters to the east in the region of the Amazon Delta were not identified as important by our model, but the shelf-edge waters approximately 100 km from the Brazilian coastline were core habitats, in the region of the Great Amazon Reef. This was also the case in the region of the Orinoco Delta further north.

In the south Atlantic, where the model predicted that ~2.8 million km$^2$ was the most important for whale sharks (Extended Data Fig. 1a, f), there was a large area of core habitat in the central Atlantic along the mid-Atlantic ridge which peaked from June to January. This area surrounded the islands of St. Helena and Ascension, but deeper waters in the Brazil (to the west) and Angola (to the east) Basins were not as important with the exception of the Guinea Rise which connected habitats identified on the West Coast of Africa, such as those in Angolan waters at approximately 11° – which were important from December to May – to island features in the central SA. On the western side of the south Atlantic, core habitats were identified in the region of the Rio Grande Rise, which extended north to approximately 8°S, and had a maximum southerly extent of 30°S. At this latitude there was also an oceanic area of importance identified around the Discovery Seamounts.

In the northwest Indian Ocean, ~780K km$^2$ of core habitat area was identified by the model, where important habitats extended out form the Red Sea through the Bad-el-Mandeb strait into the Gulf of Aden generally located in nearshore, shelf areas (Extended Data Fig. 1a, f). Important areas for whale sharks also extended south along the coast of Somalia including Socotra Island and east along the coastlines of Yemen and Oman. The Gulf of Oman and continental shelves of Iran, Pakistan and India were predicted as important with core habitat apparent in the waters off Gujarat and Karnataka, India. Habitats extended offshore along the Chagos-Laccadive Ridge into the Maldivian atolls where they varied seasonally but were consistently high at the inner atolls. Generally, oceanic waters were identified as less important in the Indian Ocean, with core habitats concentrated in coastal and shelf areas.

In the southwest Indian Ocean ~1 million km$^2$ was predicted as core habitat (Extended Data Fig. 1a, f), with a band of habitat extending south from Seychelles across the Mascarene Plateau to Mauritius and Reunion Island including areas in the Mascarene Basin with a hotspot on the Nazareth Bank. The shelf region of Madagascar was also important habitat with core areas located on the western central side of the island which extended into the Mozambique Channel, where a large portion of the channel was important for whale sharks, including the Comoros islands. On the east coast of Africa, nearshore, shelf regions around the Mozambique plateau and the waters of Kenya, Tanzania and Mozambique were identified as important habitats by the model.

Approximately 2.9 million km$^2$ of the east Indian Ocean was identified as the most important for the species (Extended Data Fig. 1a, f), with core habitats located in the coastal waters of Bangladesh where flow from the Ganges River passes through the delta into the northern Bay of Bengal. A band of lower suitability was predicted in the Andaman Sea and around the Nicobar Islands that intensified with decreasing latitude. The southern coastline of Java and the Lesser Sunda Islands were important areas, with habitats extending west into the oceanic waters around Cocos and Christmas Island and south into the North Australian Basin. Around the coast of Western Australia,

the Exmouth Plateau, Cuvier Plateau and shelf edge surrounding the Perth Basin were core habitats with a maximum southerly latitude of approximately 34°S, which extended around Cape Mentelle from January to March. On the northern Australian coast important habitats were located in both the Timor and Arafura Sea on the continental shelf.

In the west Pacific ~9.9 million km$^2$ of the region was predicted as core habitat, much of which was located throughout the Banda Sea and Celebes, Sulu, West Caroline and East Coraline Basins surrounding the islands of the Philippines, Borneo, Celebes and New Guinea (Extended Data Fig. 1a, f). These areas extended north to Taiwan and Japan connected by the Ryukyu Islands with a maximum northerly latitude of approximately 35°N from June to November contracting to approximately 30°N from December to May. In the oceanic waters south of Japan important habitats were predicted in the region of the Bonin and Izu Trench's, the Mariana Trough and near the Mariana Islands and Guam. Farther east, seamounts and island features proved important for whale sharks within the west and central Pacific, including the Caroline, Marshall, Solomon and Gilbert Islands and more southerly features including Vanuatu, Fiji Islands and New Caledonia from January to May.

Central oceanic waters in the east Pacific region, where ~6.5 million km$^2$ was predicted to be core habitat (Extended Data Fig. 1a, f), contained more disparate areas of importance located around oceanic features including the Magellan Rise, Palmyra Atoll, Kiribati and the Line Islands. A band of oceanic habitats at approximately 2°N connected oceanic features to important areas within the Guatemala Basin and nearshore regions of Central and South America. In the north of the region core habitats were located in Baja California, Islas Revillagigedo, Mathematicians Seamounts and the eastern end of the Clipperton Fracture Zone, with a northernmost latitude of approximately 34°N from July to October and 28°N for the rest of the year. In the waters off Central America, important habitat was identified in the Guatemalan and Panama Basins, the Colon, Cocos and Carnegie Ridges, and the Galapagos Rift and Islands, with the most important areas for whale sharks within 110°E and approximately 22°S, with habitats extending down the Peruvian continental shelf as far as the Nazca Ridge.

### *4.6 Discussion of whale shark habitat drivers*

#### 4.6.1 Atlantic

In the north Atlantic, DEPTH and PROXC were important predictors, differing from the south Atlantic region. This is likely due to the whale shark demography included in each compiled telemetry dataset. In the south Atlantic individuals were tagged around the island of St. Helena. St. Helena represents the only known locality in the world where adult males and females congregate in equal numbers[78]. Given this equal sex ratio, and eyewitness accounts of courtship behaviour, it

is hypothesised that the island serves as an important reproductive habitat. Interestingly oceanographic variables reflecting productivity at depth were important predictors in the south Atlantic, which was also seen in the east Pacific region. Perhaps subsurface foraging opportunities play an important role for adults which spend more time in oceanic waters where prey is generally sparce at the surface across their tropical distribution[79]. Here, we did not predict habitats in the Mediterranean Sea to avoid over extrapolation, due to its departure from environments used in the presence and background training datasets. However, whale sharks have recently been sighted there, with one juvenile whale shark potentially travelling through the Suez Canal. Based on high suitability within the Red Sea, our global model supports this hypothesis, however with favourable habitats extending across the west coast of Africa into the Gulf of Cadiz, it is also possible that the shark travelled through the Strait of Gibraltar to enter the Mediterranean Sea. In October 2011 an individual was sighted in a set-net in south Portugal in the region of the Gulf of Cadiz close to the Strait of Gibraltar[80].

Interestingly, EPI_MNKC explained the lowest amount of univariate modelled deviance in the north Atlantic but was the highest explanatory predictor from the biological oceanographic variables modelled in the south Atlantic. It may be that modelled plankton is too coarse within the Gulf of Mexico – where the majority of north Atlantic individuals were tracked – to pick up the nuances of spatio-temporal prey distributions and relationships with whale sharks tracked in the area. CHL was modelled as a proxy for prey in the northern Gulf of Mexico, where there was a strong association with shark sightings[32]. Although CHL was a reasonable predictor in the north Atlantic, showing a very similar pattern to sharks in the east Indian Ocean, finer scale prey nuances were lost at the board scale, which may explain the inability of the model to match local aggregation seasonality within the Gulf of Mexico which have been shown to be heavily linked to prey availability[81,82].

The regional divide between the north Atlantic and south Atlantic was one of the most stark globally and suggests potential environmental niche separation between populations in the Atlantic, which may be explained by the primarily juvenile male dataset used to train the model in the north Atlantic in contrast to the predominantly adult set used in the south Atlantic. However, both models predicted suitability along the coast of Brazil which suggests potential coastal connectivity across the basin. This coastline, along with the western coast of Africa remains largely understudied and more research is needed to explore the suitability of these areas for whale sharks.

### 4.6.2 Indian Ocean

Previous models have shown a seasonal shift in habitat suitability following a clockwise direction from the south-west Indian Ocean in autumn, shifting north into central oceanic waters in winter and spring, and back to southern waters in summer[34]. These observations were driven by changes in SST which reflected a ‘C’ shape in mapped outputs where individuals inhabited a narrow range

of temperatures (90% of observations occurring between 26.5 and 30 °C)[34]. Our model outputs presented here showed less pronounced monthly trends with a slight November to April peak in the coastal southwest Indian Ocean, followed by January to May peaks in northern and central coastal waters and June to November peaks in the eastern basin. Here, whale sharks tracked in the Indian Ocean showed a broader SST range, with only 60% of individual locations tracked in cells where temperature ranged from 26 to 30 °C, which may go some way to explaining the difference in seasonality we observed, which did not follow SST thermoclines as closely as previously predicted at the ocean basin scale based on fisheries observations (which are known to track SST)[34]. Observed trends were more closely matched to previous global assessments, where high suitability was recorded in northern latitudes of the Indian Ocean, including Maldivian, Indian and Bangladesh waters from January to March and, more locally, in Ningaloo and Gujarat from April to June[10]. Univariate models revealed that DEPTH, PROXC and CHL where more important predictors of whale shark presence in the southwest, northeast and east Indian Ocean than SST. Indeed, in the southwest Indian Ocean, PROXC and DEPTH explained the most deviance from the complete set of oceanographic variables included in the best performing hypothesis. At the southern extreme of their range in the Indian Ocean there was a band of habitat extending along the eastern coastline of South Africa where a section of important area contracted with decreasing latitude and eventually disappeared. It could be the case that individuals in the Indian Ocean follow these favourable habitats south and are met with sudden changes in conditions, such as SST drops, which may help explain the large number of whale shark stranding's recorded there[83]. A reduction in metabolic rate due to sudden changes in water temperature and/ or a steeply-sloping continental shelf combined with heavy wave action have been pointed out as possible causes of stranding events[84], and we suggest that sharks reach these unsuitable conditions by following the band of habitat identified by the model and becoming caught out by rapid environmental changes. This hypothesis may also go some way toward explaining stranding events on India's eastern coastline where a band of important habitat could lead sharks into less favourable waters. Here, abrupt decreases in dissolved oxygen occur seasonally within the Bay of Bengal, driven by shoaling of the OMZ[85,86], potentially leading to surface waters that are too anoxic for whale sharks to remain within for extended periods. This habitat fragmentation could cause a major issue for this species in future with expanding OMZs, possibly resulting in increased stranding events[86].

The model predicted presence in Gujarat and the Maldives corroborating both observations and previous local studies[79]. It also suggests that suitable habitats are located along the coastline of Somalia, with peaks in April, May and from September to November. To date, individuals have not been tracked in Somali waters and there are limited research initiatives in place to validate these predictions. The Somali EEZ may act as an important transiting or feeding area for whale sharks, perhaps moving between northern and southern waters in the Indian Ocean. This is somewhat supported by microsatellite DNA markers that found aggregation sites north and south of Somalia

have similar levels of allelic richness and appear to be derived from the same source population[87]. It is crucial that the species is protected across their entire range if population declines[88] are to be appropriately managed in the future. Focus should be on ensuring remote and difficult to access habitats are incorporated into research and management initiatives, which in the Indian Ocean includes habitats located in Indian, Bangladesh and Somali waters, among others.

One of the primary hypotheses explaining seasonal coastal distributions is that individuals gather to feed on dense prey patches to obtain sufficient energy for somatic and reproductive growth within otherwise sparse, oligotrophic tropical surface waters[44,89]. EPI_MNKC concentrations of approximately 6 ww g $m^{-2}$ led to habitat suitability values greater than 0.5 when univariate models in the southwest Indian Ocean were explored, although the variable explained less than 20% of the deviance within the region. This indeed suggests that dense prey patches play a role in the southwest Indian Ocean in that they positively influence presence, but that perhaps at broad scales their low spatial frequency leads to lower deviance when compared to DEPTH and PROXC, for example. Many well-known coastal aggregation areas are located in the southwest Indian Ocean[79], including Mafia Island in Tanzania[90], Tofo Beach in Mozambique[91], Nosy Be in Madagascar[92] and Mahe in Seychelles[93], where residency ranges from approximately 15.2 (Mozambique) to 79.1 (Tanzania) days per year[94]. Some of these sites have been well explained by bathymetric features[31], and with high site residency in some locations, the predicted lower probability of whale shark presence observed in oceanic waters could suggest that individuals in the region spend considerable time in these coastal aggregation regions. This is supported by the lack of evidence of connectivity between aggregation sites in the Indian Ocean[95].

### 4.6.3 Pacific

In the Pacific, previous broad scale habitat projections have been limited due to the low number of fisheries encounter records in the region[10]. Our modelled predictions show a markedly higher oceanic distribution than the Indian Ocean with shifting northerly and southerly limits in both the east Pacific and west Pacific, which appear to be connected by a band or ‘corridor’ of favourable oceanic habitat at approximately 2°N in line with the Equatorial Current. Tracking data from the region has indicated that whale sharks exhibit preferential occupancy of current linked thermo-biological frontal systems[36], an observation that has also been noted in the planktivorous basking shark *Cetorhinus maximus* in the Eastern Atlantic[96].

In the west Pacific, habitat suitability showed shifting northern and southern latitudinal limits with a maximum latitude of 40°N from July to September and a minimum of 20°S from January to June linked to dynamic thermoclines given the strong reported relationship with SST. This is similar to known occurrence trends where whale sharks have been recorded during interactions with tuna purse seine fishery activities near Japan in the northern west Pacific[97]. Between 1996 and 2002,

sets where whale sharks were used as a mark by fishermen to find skipjack and albacore tuna schools were observed in the area of 20 – 25°N in March and April, rapidly increasing in the area of 30 – 40°N and 140 – 150°E in June with a peak around July and August[97]. In the southern west Pacific, a number of whale sharks were identified in the waters of New Caledonia by OBIS citizen scientists, and they have even been recorded as far south as New Zealand[98]. In New Zealand, sightings have historically been concentrated over the outer shelf and shelf break in areas influenced by the East Auckland Current at SSTs of 21 – 24°C, occurring from late spring to early autumn (November through to April) but most frequent in midsummer (February) when upwelling along the north-east shelf is weakest[98]. Although this observation matches the modelled southerly distribution season, New Zealand waters were not identified as important habitats by the model. This may be due to low connectivity between the tracked individuals in the west Pacific and the preference of sharks occupying waters further south at fringe habitats, highlighting the possibility of the model not being able to identify all suitable habitat regions.

Most tracked individuals in the east Pacific were large (> 6.5 m TL) females suggesting that productive rather than temperature driven ocean features are important for this particular demographic. The regular presence of mature, female sharks in deep waters in the southern part of the Gulf of California and Galapagos Marine Reserve suggests that their occurrence in this region is related to reproduction[99,100]. High subsurface productivity could provide optimal conditions for pupping, although relatively few neonates have been observed in the east Pacific[101]. Interestingly there was a strong association with dissolved oxygen concentration at depth (O2100m) observed in the east Pacific, with habitat suitability increasing with decreasing O2100m values and the highest suitability occurring over anoxic subsurface waters. Given their large body size and deep diving behaviour, adult whale sharks may be capable of moving through low oxygen environments and OMZs could provide ideal protection for neonates that will be more difficult to access by oxygen dependent predators such as the blue shark or blue marlin, both of which have been known to prey on whale shark young in the Atlantic and Indian Ocean. This may also be the case in the northwest Indian Ocean OMZ that seasonally persists in the Arabian Sea and Bay of Bengal[86]. Here, neonates could feed on specialised prey such as *Lucicutia grandis*[85] at the lower OMZ boundary whilst remaining protected from pelagic predators. Indeed, a large number of neonates have been accidentally captured or sighted in close proximity to the Indian Ocean OMZ which may relate to its more dynamic nature displacing young when compared to other global OMZs[18]. Further fine scale studies exploring more accurate dissolved oxygen requirements and capabilities using novel sensors are needed for whale sharks.

Sea surface height (SSH) was a strong predictor in the east Pacific explaining over 20% of the deviance, and with El Niño being associated with increases in SSH which becomes negatively associated with whale shark presence with increasing magnitude, a next step would be to explore

inter-annual variability within the region and whether El Niño influences habitat availability at the broad scale. Connectivity between Galapagos and Peru has been previously identified with peak occurrences in Peru during austral summer from January to March, which was matched by our modelled predicted habitats which peaked from December to May[101].

### *4.7 Future range shifts*

An overall difference in southerly limit shifts among regions was projected by our models (median shift 69 km; mean, 278 km ± 532 s.d.; Kruskal-Wallis rank sum test, P<0.01, Table S2), with the greatest shift expected in the north Atlantic (2,359 km by 2100 under scenario ssp585) followed by the east Pacific (1,943 km by 2100 under scenario ssp585), albeit in the opposite direction (Extended Data Fig. 4 and Figs. S20, S21). Distribution shifts calculated using the weighted habitat centroid (centre point of the weighted habitat; median 258 km; 471 mean ± 509 s.d.; Kruskal-Wallis rank sum test, P<0.01, Table S12) and core habitat centroid (median shift, 325 km; mean, 592 km ± 669 s.d.; Kruskal-Wallis rank sum test, P<0.001, Table S12) were apparent among regions, with the greatest core habitat shift expected in the north Atlantic (3,174 km by 2100 under scenario ssp585) and least in the west Pacific (55 km by 2100 under scenario ssp585, Figs. S20, S21). The smallest shift in habitat centroid coupled with the largest northerly and southerly shifts suggest that in the west Pacific and east Pacific, respectively, change is distributed evenly north and south of current distribution centres, with a slight northward skew which was reflected in both maps and density plots (Fig. 1 and Figs. S6, S7).

Quarterly northern limit shifts were different at the global scale (Kruskal-Wallis rank sum test, P<0.001), with the greatest disparity apparent between Q2 (April – June) and Q4 (October – December) (Dunn's multiple comparisons, P<0.01), and the difference in southerly limits (Kruskal-Wallis rank sum test, P<0.001) was apparent across all quarterly combinations (Dunn's multiple comparisons, P<0.001 for all quarter comparisons, Fig. S16c). By the end of the century under scenario ssp585, the northerly limit of core habitats in the north Atlantic shifted south by almost 2,000 km in Q1 (January – March) which was in contrast to all other regions, including the east Pacific where northward shifts of over 1,000 km are expected with a higher degree of consistency across the year (Fig. S16c). Southern core habitat limits were also contrasting in the north Atlantic and east Pacific where northward shifts of >2,000 km in Q4 in the north Atlantic and southward shifts of >2,000 km in Q2 in the east Pacific are expected (Fig. S16c). The difference between Q1 and Q3 was most pronounced when modelling the shift in weighted habitat centroid (Kruskal-Wallis rank sum test, P<0.05, Dunn's multiple comparisons, P<0.01 for Q1 – Q3), and second to the difference between Q2 and Q3 when modelling the change in core habitat centroid (Kruskal-Wallis rank sum test, P<0.01, Dunn's multiple comparisons, P=0.01 for Q1 – Q3 and P<0.01 for Q2 – Q3).

### *4.8 Past climatic events*

Similar to those detected in the north Atlantic, potential similarities between past and future habitat trends were also evident the Pacific, notably in 2015 and 2016 (Figs. S23, S25) when the Northeast Pacific marine heatwave occurred in 2014 – 2016[103-105]. In the Indian Ocean, habitat patterns in both 2010 and 2016 – coinciding with the Northern hemisphere heatwaves of 2010 (with record high surface temperatures in the northern Indian Ocean[106]) and the long-lasting marine heatwaves that took place in 2015/ 2016[107,108] – were similar to future projections (Figs. S24, S25).

### *4.9 Study limitations*

Here, we focussed on a single species to ensure that complex habitat relationships were captured sufficiently and validated holistically[109], and included environmental variables other than temperature to ensure potential climatic responses were not masked[110]. We also divided the global ocean into geographic regions within which environments were sampled at the ocean basin scale to reflect local oceanography[111,112]. However, a limitation of our study, and all other studies to date, was that only a small sample of whale sharks inhabiting each region could be satellite tracked, potentially underrepresenting contributing factors that can influence distributions. This could mean that important behaviours – such as more extensive oceanic movements – were not captured sufficiently in our first order estimation of suitable habitats due to logistical challenges with tracking technology being able to capture multi-year cycles of individual movements. Although we tracked nearly 350 individuals, there are still large gaps in the coverage we were able to achieve globally, including sites such as the Azores in the Atlantic and the Maldives in the Indian Ocean. These sites were selected to explore biological realism of our model which showed a good level of quantitative and qualitative performance. However, more movement tracking data would provide insights into underrepresented demographics and enable the increased sample sizes needed to explore more mechanistic drivers of movement for whale sharks.

Although tracking data can provide important insights into species habitat use, deployments on marine megafauna can be costly and logistically challenging, potentially hindering data collection of this kind in some regions. Where tracking data is not available, other records of animal occurrence can also be valuable in informing models of animal distributions[113]. In data-sparse regions where shipping has been identified as an issue for whale sharks, or other marine megafauna, steps to overcome data limitations should be taken so that baseline estimates of current and future threat overlap can be generated[113,114], including mining citizen science databases such as those included in our model validation analyses. Our study helps identify regions where these more local assessments could be undertaken.

Other important weaknesses include the uncertainties of climate projections or modelled environmental data and the inherent correlative nature of some distribution models, as well as the potential for unreliable extrapolation outside observed environmental space[115]. Although our various control analyses and validation steps go some way to limiting such concerns, it is evident that our approach represents a first step toward more accurately delineating important future habitats and to provide novel, baseline estimates of future potential co-occurrence of whale sharks and shipping.

## 5. Supplementary References


1 Schouten, M. & Villa, E. Gifted by many whale sharks in *CW Azores* (2020) [Accessed Feb 2024].

2 Fontes, J., McGinty, N., Machete, M. & Afonso, P. Whale shark-tuna associations, insights from a small pole-and-line fishery from the mid-north Atlantic. *Fisheries Research* **229**, 105598 (2020).

3 Afonso, P., McGinty, N. & Machete, M. Dynamics of whale shark occurrence at their fringe oceanic habitat. *PLoS One* **9**, e102060 (2014).

4 Unknown. Whale shark in *CapeVergeIslands.org* (2023) [Accessed Feb 2024].

5 Graham, R., Seymour, Z., Monteiro, J. L., Lima, C. & Lima, J. Cabo Verde Expedtion. (2020).

6 Di Beneditto, A. P. M., Moreira, S. C. & Siciliano, S. Endangered whale sharks in southeastern Brazil: Records and management issues. *Ocean & Coastal Management* **201**, 105491 (2021).

7 Andrades, R., Pinheiro, H. T., Santos, R. G., Martins, A. S. & Costa, P. A. S. A new record of whale shark *Rhincodon typus* in Brazilian waters: a report of association with Caranx crysos. *Journal of Fish Biology* **81**, 2092-2094 (2012).

8 Cardin, A. Whale shark spotted in Rio’s Guanabara bay in *The Rio Times* (2020). [Accessed Feb 2024].

9 Higgs, N. D., Gates, A. R. & Jones, D. O. B. Fish Food in the Deep Sea: Revisiting the Role of Large Food-Falls. *PLOS ONE* **9**, e96016 (2014).

10 Sequeira, A. M., Mellin, C., Fordham, D. A., Meekan, M. G. & Bradshaw, C. J. Predicting current and future global distributions of whale sharks. *Glob Chang Biol* **20**, 778-789 (2014).

11 Weir, C. R. Sightings of whale sharks (*Rhincodon typus*) off Angola and Nigeria. *Marine Biodiversity Records* **3**, e50 (2010).

12 Pravin, P. Whale shark in the Indian coast – Need for conservation. *Current Science* **79**, 310-315 (2000).

13 Akhilesh, K. V. *et al.* Landings of whale sharks *Rhincodon typus* Smith, 1828 in Indian waters since protection in 2001 through the Indian Wildlife (Protection) Act, 1972. *Environmental Biology of Fishes* **96**, 713-722 (2013).

14 Arrowsmith, L. M., Sequeira, A. M. M., Pattiaratchi, C. B. & Meekan, M. G. Water temperature is a key driver of horizontal and vertical movements of an ocean giant, the whale shark *Rhincodon typus*. *Marine Ecology Progress Series* **679**, 101-114 (2021).

15 Donati, G. *et al.* New insights into the South Ari atoll whale shark, *Rhincodon typus*, aggregation. Intetnational Whale Shark Conference 2016 (2016).

16 Valsecchi, S. *et al.* Analysis of the temporal and spatial variability of whale shark (*Rhincodon typus*) aggregation in the South Ari Marine Protected Area, Maldives, Indian Ocean. *The European Zoological Journal* **88**, 684-697 (2021).

17 Riley, M. J., Hale, M. S., Harman, A. & Rees, R. G. Analysis of whale shark *Rhincodon typus* aggregations near South Ari Atoll, Maldives Archipelago. *Aquatic Biology* **8**, 145-150 (2010).

18 Rowat, D. *et al.* New records of neonatal and juvenile whale sharks (*Rhincodon typus*) from the Indian Ocean. *Environmental Biology of Fishes* **82**, 215-219 (2007).

19 Charoux, I. Whale shark encounter in Mauritius while Big game fishing with Van Charoux & Vacances Adventures in *YouTube* (2018) [Accessed Feb 2024].

20 Rowat, D. Occurrence of whale shark (*Rhincodon typus*) in the Indian Ocean: A case for regional conservation. *Fisheries Research* **84**, 96-101 (2007).

21 Gudger, E. W. The Geographical Distribution of the Whale-Shark (*Rhineodon typus*). *Proceedings of the Zoological Society of London* **104**, 863-894 (1934).

22 Unknown. Giant whale shark caught in Chattogram. *The Buisness Standard* (2019) [Accessed Feb 2024].

23 Haque, A. B., Cavanagh, R. D. & Seddon, N. Evaluating artisanal fishing of globally threatened sharks and rays in the Bay of Bengal, Bangladesh. *PLOS ONE* **16**, e0256146 (2021).

24 Tanvir, A. M. Effective protection of whale shark in the Swatch of No Ground in *Daily Sun* (2018) [Accessed Feb 2024].

25 Chen, V. Y. & Phipps, M. J. Management and Trade of Whale Sharks in Taiwan. (2002).

26 Hsu, H.-H., Joung, S.-J., Liao, Y.-Y. & Liu, K.-M. Satellite tracking of juvenile whale sharks, *Rhincodon typus*, in the Northwestern Pacific. *Fisheries Research* **84**, 25-31 (2007).

27 VFD & PIFFA. Vanuatu National Plan of Action on Sharks (2015–2018). (2014).

28 Donnelly, L. Watch whale shark get up close with diver at SS President Coolidge, Espiritu Santo in *YouTube* (2013) [Accessed Feb 2024].

29 MatavaFiji. Whale Shark in Kadavu, Fiji in *YouTube* (2009) [Accessed Feb 2024].

30 RaDivers. Whale shark at Volivoli Beach Resort, Fiji's Premier Dve Resort in *FaceBook* (2019) [Accessed Feb 2024].

31 Copping, J. P., Stewart, B. D., McClean, C. J., Hancock, J. & Rees, R. Does bathymetry drive coastal whale shark (*Rhincodon typus*) aggregations? *PeerJ* **6**, e4904 (2018).

32 McKinney, J. A., Hoffmayer, E. R., Wu, W., Fulford, R. & Hendon, J. Feeding habitat of the whale shark *Rhincodon typus* in the northern Gulf of Mexico determined using species distribution modelling. *Marine Ecology Progress Series* **458**, 199-211 (2012).

33 Sleeman, J. C. *et al.* To go or not to go with the flow: Environmental influences on whale shark movement patterns. *Journal of Experimental Marine Biology and Ecology* **390**, 84-98 (2010).

34 Sequeira, A., Mellin, C., Rowat, D., Meekan, M. G. & Bradshaw, C. J. A. Ocean-scale prediction of whale shark distribution. *Diversity and Distributions* **18**, 504-518 (2012).

35 Báez, J. C., Barbosa, A. M., Pascual, P., Ramos, M. L. & Abascal, F. Ensemble modeling of the potential distribution of the whale shark in the Atlantic Ocean. *Ecology and Evolution* **10**, 175-184 (2020).

36 Ryan, J. P., Green, J. R., Espinoza, E. & Hearn, A. R. Association of whale sharks (*Rhincodon typus*) with thermo-biological frontal systems of the eastern tropical Pacific. *PLOS ONE* **12**, e0182599 (2017).

37 Queiroz, N. *et al.* Global spatial risk assessment of sharks under the footprint of fisheries. *Nature* **572**, 461-466 (2019).

38 Block, B. A. *et al.* Tracking apex marine predator movements in a dynamic ocean. *Nature* **475**, 86-90 (2011).

39 Escalle, L. *et al.* Environmental factors and megafauna spatio-temporal co-occurrence with purse-seine fisheries. *Fisheries Oceanography* **25**, 433-447 (2016).

40 Vedor, M. *et al.* Climate-driven deoxygenation elevates fishing vulnerability for the ocean's widest ranging shark. *eLife* **10**, e62508 (2021).

41 Queiroz, N. *et al.* Ocean-wide tracking of pelagic sharks reveals extent of overlap with longline fishing hotspots. *Proc Natl Acad Sci U S A* **113**, 1582-1587 (2016).

42 Schlaff, A. M., Heupel, M. R. & Simpfendorfer, C. A. Influence of environmental factors on shark and ray movement, behaviour and habitat use: a review. *Reviews in Fish Biology and Fisheries* **24**, 1089-1103 (2014).

43 Braun, C. D., Gaube, P., Sinclair-Taylor, T. H., Skomal, G. B. & Thorrold, S. R. Mesoscale eddies release pelagic sharks from thermal constraints to foraging in the ocean twilight zone. *Proc Natl Acad Sci U S A* **116**, 17187-17192 (2019).

44 Rohner, C. A. & Prebble, C. E. Whale Shark Foraging, Feeding, and Diet. *Whale Sharks: Biology, Ecology, and Conservation* (2021).

45 Elith, J. & Leathwick, J. R. Species Distribution Models: Ecological Explanation and Prediction Across Space and Time. *Annual Review of Ecology, Evolution, and Systematics* **40**, 677-697 (2009).

46 Scales, K. L. *et al.* Identifying predictable foraging habitats for a wide-ranging marine predator using ensemble ecological niche models. *Diversity and Distributions* **22**, 212-224 (2016).

47 Sousa, L. L., Queiroz, N., Mucientes, G., Humphries, N. E. & Sims, D. W. Environmental influence on the seasonal movements of satellite-tracked ocean sunfish *Mola mola* in the north-east Atlantic. *Animal Biotelemetry* **4**, 7 (2016).

48 Sims, D. W. *et al.* Hunt warm, rest cool: bioenergetic strategy underlying diel vertical migration of a benthic shark. *J Anim Ecol* **75**, 176-190 (2006).

49 Papastamatiou, Y. P. *et al.* Drivers of Daily Routines in an Ectothermic Marine Predator: Hunt Warm, Rest Warmer? *PLoS One* **10**, e0127807 (2015).

50 Sedberry, G. & Loefer, J. Satellite telemetry tracking of swordfish, *Xiphias gladius*, off the eastern United States. *Marine Biology* **139**, 355-360 (2001).

51 Queiroz, N., Humphries, N. E., Noble, L. R., Santos, A. M. & Sims, D. W. Short-term movements and diving behaviour of satellite-tracked blue sharks *Prionace glauca* in the northeastern Atlantic Ocean. *Marine Ecology Progress Series* **406**, 265-279 (2010).

52 Reglero, P. *et al.* Atlantic bluefin tuna spawn at suboptimal temperatures for their offspring. *Proceedings of the Royal Society B: Biological Sciences* **285**, 20171405 (2018).

53 Nakamura, I., Matsumoto, R. & Sato, K. Body temperature stability in the whale shark, the world's largest fish. *Journal of Experimental Biology* **223** (2020).

54 Hearn, A. R. *et al.* Whale Shark Movements and Migrations. *Whale Sharks: Biology, Ecology, and Conservation* (2021).

55 Norman, B. M. *et al.* Undersea Constellations: The Global Biology of an Endangered Marine Megavertebrate Further Informed through Citizen Science. *BioScience* **67**, 1029-1043 (2017).

56 Sarmiento, J. L. in *Ocean Biogeochemical Dynamics* (Princeton University Press, 2013).

57 Sims, D. W. *et al.* Scaling laws of marine predator search behaviour. *Nature* **451**, 1098-1102 (2008).

58 Scales, K. L. *et al.* REVIEW: On the Front Line: frontal zones as priority at-sea conservation areas for mobile marine vertebrates. *Journal of Applied Ecology* **51**, 1575-1583 (2014).

59 Polovina, J. J., Howell, E., Kobayashi, D. R. & Seki, M. P. The transition zone chlorophyll front, a dynamic global feature defining migration and forage habitat for marine resources. *Progress in Oceanography* **49**, 469-483 (2001).

60 Wyatt, A. S. J. *et al.* Enhancing insights into foraging specialization in the world's largest fish using a multi-tissue, multi-isotope approach. *Ecological Monographs* **89**, e01339 (2019).

61 Keller, B. A., Putman, N. F., Grubbs, R. D., Portnoy, D. S. & Murphy, T. P. Map-like use of Earth’s magnetic field in sharks. *Current Biology* **31**, 2881-2886.e2883 (2021).

62 Barbosa, A. M. fuzzySim: applying fuzzy logic to binary similarity indices in ecology. *Methods in Ecology and Evolution* **6**, 853-858 (2015).

63 Carlson, C. J. embarcadero: Species distribution modelling with Bayesian additive regression trees in r. *Methods in Ecology and Evolution* **11**, 850-858 (2020).

64 Jurka, T. P. Maxent: an R package for low-memory multinomial logistic regression with support for semi-automated text classification. *The R Journal* **4**, 56-59 (2012).

65 Greenwell, B., Boehmke, B., Cunningham, J., Developers, G. & Greenwell, M. B. Package ‘gbm’. *R package version* **2** (2019).

66 Hastie, T. & Hastie, M. T. Package ‘gam’. *R package version*, 90124-90123 (2015).

67 RColorBrewer, S. & Liaw, M. A. Package ‘randomforest’. *University of California, Berkeley: Berkeley, CA, USA* (2018).

68 Liu, C., White, M. & Newell, G. Measuring and comparing the accuracy of species distribution models with presence–absence data. *Ecography* **34**, 232-243 (2011).

69 Barbosa, A. M., Real, R., Muñoz, A. R. & Brown, J. A. New measures for assessing model equilibrium and prediction mismatch in species distribution models. *Diversity and Distributions* **19**, 1333-1338 (2013).

70 Valavi, R., Elith, J., Lahoz-Monfort, J. J. & Guillera-Arroita, G. blockCV: An r package for generating spatially or environmentally separated folds for k-fold cross-validation of species distribution models. *Methods in Ecology and Evolution* **10**, 225-232 (2019).

71 Hazen, E. L. *et al.* Where did they not go? Considerations for generating pseudo-absences for telemetry-based habitat models. *Movement Ecology* **9**, 5 (2021).

72 O'Toole, M., Queiroz, N., Humphries, N. E., Sims, D. W. & Sequeira, A. M. M. Quantifying effects of tracking data bias on species distribution models. *Methods in Ecology and Evolution* **12**, 170-181 (2020).

73 O'Neill, B. C. *et al.* The Scenario Model Intercomparison Project (ScenarioMIP) for CMIP6. *Geosci. Model Dev.* **9**, 3461-3482 (2016).

74 Eyring, V. *et al.* Overview of the Coupled Model Intercomparison Project Phase 6 (CMIP6) experimental design and organization. *Geoscientific Model Development* **9**, 1937-1958 (2016).

75 Meinshausen, M. *et al.* The shared socio-economic pathway (SSP) greenhouse gas concentrations and their extensions to 2500. *Geosci. Model Dev.* **13**, 3571-3605 (2020).

76 Stock, C. A. *et al.* On the use of IPCC-class models to assess the impact of climate on living marine resources. *Progress in Oceanography* **88**, 1-27 (2011).

77 Cheung, W. W. L., Brodeur, R. D., Okey, T. A. & Pauly, D. Projecting future changes in distributions of pelagic fish species of Northeast Pacific shelf seas. *Progress in Oceanography* **130**, 19-31 (2015).

78 Perry, C. T. *et al.* St. Helena: An Important Reproductive Habitat for Whale Sharks (*Rhincodon typus*) in the Central South Atlantic. *Frontiers in Marine Science* **7** (2020).

79 Rohner, C. A., Norman, B., Araujo, G., Holmberg, J. & Pierce, S. J. Population ecology of whale sharks. *Whale Sharks: Biology, Ecology, and Conservation*, 129-152 (2021).

80 Rodrigues, N. V. *et al.* First record of a whale shark *Rhincodon typus* in continental Europe. *Journal of Fish Biology* **81**, 1427-1429 (2012).

81 de la Parra Venegas, R. *et al.* An unprecedented aggregation of whale sharks, *Rhincodon typus*, in Mexican coastal waters of the Caribbean Sea. *PLoS One* **6**, e18994 (2011).

82 Ramírez-Macías, D. *et al.* Patterns in composition, abundance and scarring of whale sharks *Rhincodon typus* near Holbox Island, Mexico. *J Fish Biol* **80**, 1401-1416 (2012).

83 Wosnick, N., Leite, R. D., Giareta, E. P., Morick, D. & Musyl, M. Global assessment of shark strandings. *Fish and Fisheries* **23**, 786-799 (2022).

84 Beckley, L. E., Cliff, G., Smale, M. J. & Compagno, L. J. Recent strandings and sightings of whale sharks in South Africa. *Environmental Biology of Fishes* **50**, 343-348 (1997).

85 Vidhya, V. *et al.* Oxygen minimum zone copepods in the Arabian Sea and the Bay of Bengal: Their adaptations and status. *Progress in Oceanography* **206**, 102839 (2022).

86 Gilly, W. F., Beman, J. M., Litvin, S. Y. & Robison, B. H. Oceanographic and biological effects of shoaling of the oxygen minimum zone. *Ann Rev Mar Sci* **5**, 393-420 (2013).

87 Hardenstine, R. S. *et al.* Pieces in a global puzzle: Population genetics at two whale shark aggregations in the western Indian Ocean. *Ecology and Evolution* **12**, e8492 (2022).

88 Pierce, S. J. & Norman, B. *Rhincodon typus. The IUCN Red List of Threatened Species 2016*, 2016).

89 Rohner, C. A. *et al.* Whale sharks target dense prey patches of sergestid shrimp off Tanzania. *J Plankton Res* **37**, 352-362 (2015).

90 Rohner, C. A. *et al.* No Place Like Home? High Residency and Predictable Seasonal Movement of Whale Sharks Off Tanzania. *Frontiers in Marine Science* **7**, 423 (2020).

91 Prebble, C. E. *et al.* Limited latitudinal ranging of juvenile whale sharks in the Western Indian Ocean suggests the existence of regional management units. *Marine Ecology Progress Series* **601**, 167-183 (2018).

92 Diamant, S. *et al.* Population structure, residency, and abundance of whale sharks in the coastal waters off Nosy Be, north-western Madagascar. *Aquatic Conservation: Marine and Freshwater Ecosystems* **31**, 3492-3506 (2021).

93 Rowat, D. & Gore, M. Regional scale horizontal and local scale vertical movements of whale sharks in the Indian Ocean off Seychelles. *Fisheries Research* **84**, 32-40 (2007).

94 Araujo, G. *et al.* Improving sightings-derived residency estimation for whale shark aggregations: A novel metric applied to a global data set. *Frontiers in Marine Science* **9** (2022).

95 Andrzejaczek, S. *et al.* The ecological connectivity of whale shark aggregations in the Indian Ocean: a photo-identification approach. *Royal Society Open Science* **3**, 160455 (2016).

96 Sims, D. W. Sieving a living: a review of the biology, ecology and conservation status of the plankton-feeding basking shark *Cetorhinus maximus*. *Advances in Marine Biology* **54**, 171-220 (2008).

97 Matsunaga, H., Nakano, H., Okamoto, H. & Suzuki, Z. in *16th Meeting of the Standing Committee on tuna and billfish (ed. J. Fisheries Research Agency), Nat Res Inst Far Sea Fish, Shizuoka.* 1-7.

98 Duffy, C. A. J. Distribution, seasonality, lengths, and feeding behaviour of whale sharks (*Rhincodon typus*) observed in New Zealand waters. *New Zealand Journal of Marine and Freshwater Research* **36**, 565-570 (2002).

99 Ramírez-Macías, D. *et al.* Oceanic adults, coastal juveniles: tracking the habitat use of whale sharks off the Pacific coast of Mexico. *PeerJ* **5**, e3271 (2017).

100 Acuña-Marrero, D. *et al.* Whale shark (*Rhincodon typus*) seasonal presence, residence time and habitat use at Darwin Island, Galapagos Marine Reserve. *PLoS One* **9**, e115946 (2014).

101 Pajuelo, M. *et al.* Occurrence and Bycatch of Juvenile and Neonate Whale Sharks (*Rhincodon typus*) in Peruvian Waters. *Pacific Science* **72**, 463-473, 411 (2018).

102 Edrén, S. M., Wisz, M. S., Teilmann, J., Dietz, R. & Söderkvist, J. Modelling spatial patterns in harbour porpoise satellite telemetry data using maximum entropy. *Ecography* **33**, 698-708 (2010).

103 Gentemann, C. L., Fewings, M. R. & García-Reyes, M. Satellite sea surface temperatures along the West Coast of the United States during the 2014–2016 northeast Pacific marine heat wave. *Geophysical Research Letters* **44**, 312-319 (2017).

104 Jacox, M. G., Tommasi, D., Alexander, M. A., Hervieux, G. & Stock, C. A. Predicting the Evolution of the 2014–2016 California Current System Marine Heatwave From an Ensemble of Coupled Global Climate Forecasts. *Frontiers in Marine Science* **6** (2019).

105 Di Lorenzo, E. & Mantua, N. Multi-year persistence of the 2014/15 North Pacific marine heatwave. *Nature Climate Change* **6**, 1042-1047 (2016).

106 Trenberth, K. E. & Fasullo, J. T. Climate extremes and climate change: The Russian heat wave and other climate extremes of 2010. *Journal of Geophysical Research: Atmospheres* **117** (2012).

107 Zhang, Y., Du, Y., Feng, M. & Hu, S. Long-Lasting Marine Heatwaves Instigated by Ocean Planetary Waves in the Tropical Indian Ocean During 2015–2016 and 2019–2020. *Geophysical Research Letters* **48**, e2021GL095350 (2021).

108 Piatt, J. F. *et al.* Extreme mortality and reproductive failure of common murres resulting from the northeast Pacific marine heatwave of 2014-2016. *PLOS ONE* **15**, e0226087 (2020).

109 Botkin, D. B. *et al.* Forecasting the Effects of Global Warming on Biodiversity. *BioScience* **57**, 227-236 (2007).

110 McHenry, J., Welch, H., Lester, S. E. & Saba, V. Projecting marine species range shifts from only temperature can mask climate vulnerability. *Global Change Biology* **25**, 4208-4221 (2019).

111 Burrows, M. T. *et al.* Ocean community warming responses explained by thermal affinities and temperature gradients. *Nature Climate Change* **9**, 959-963 (2019).

112 Duffy, K., Gouhier, T. C. & Ganguly, A. R. Climate-mediated shifts in temperature fluctuations promote extinction risk. *Nature Climate Change* **12**, 1037-1044 (2022).

113 Braun, C. D. *et al.* Building use-inspired species distribution models: Using multiple data types to examine and improve model performance. *Ecological Applications* ***33*****(6)**, e2893 (2023).

114 Redfern, J.V., Moore, T.J., Fiedler, P.C., de Vos, A., Brownell, R.L., Jr, Forney, K.A., Becker, E.A. and Ballance, L.T. Predicting cetacean distributions in data-poor marine ecosystems. *Diversity and Distributions* **23**, 394-408 (2017).

115 Dawson, T. P., Jackson, S. T., House, J. I., Prentice, I. C. & Mace, G. M. Beyond Predictions: Biodiversity Conservation in a Changing Climate. *Science* **332**, 53-58 (2011).

## 6. Supplementary Acknowledgements

We thank R.R. Reisinger for modelling advice and Global Fishing Watch for provision of shipping data (https://www.globalfishingwatch.org). Funding to support the data analysis was provided by the UK Natural Environment Research Council (NERC) through a University of Southampton INSPIRE DTP Studentship to F.C.W. (NE/S007210/1), a NERC Discovery Science Grant to D.W.S. (NE/R00997/X/1), and a European Research Council (ERC) Advanced Grant (883583 OCEAN DEOXYFISH) to D.W.S. within the EU Horizon 2020 Programme. Additional funding support was provided by Fundação para a Ciência e a Tecnologia (FCT) through PTDC/BMA/3536/2021 and CEECIND/02857/2018 and the EU Horizon 2020 Programme (857251) to N.Q. D.W.S. was supported by a Marine Biological Association Senior Research Fellowship. Acknowledgement of the funding sources supporting whale shark tagging and other contributions are given in full in the Supplementary Information. This research was conducted as part of the Global Shark Movement Project (www.globalsharkmovement.org).

**G.A.** and **J.L.** thank Mrs Angelique Songco and the Park Rangers for their collaboration and support while in Tubbataha Reefs Natural Park, and the Local Government Units and local communities of Cagayancillo, Talisayan, Malimono, Pintuyan and San Ricardo.

**A.B.** thanks Great Barrier Reef Marathon Research Fund and Slattery Family Trust for funding and Daniel McCarthy for tagging the first whale shark.

**S.B.-L.** acknowledges the Natural National Parks of Colombia, Fondo Patrimonial Malpelo, Foundation Prince Alberto II of Monaco, Colombia Bio and Embarcaciones Asturias.

**A.D.M.D.** acknowledges the support of the Environment and Natural Resources Directorate of the St Helena Government and the staff of Georgia Aquarium in Atlanta.

**M.V.E., A.B.S., A.H.** and **I.S.** would like to thank the Indonesian Ministry of Environment and Forestry, the Ministry of Marine Affairs and Fisheries, the Cenderawasih Bay National Park Authority, and the people and governments of West Papua and Nusa Tenggara Barat provinces (especially those from Desa Labuhan Jambu and Desa Kwatisore) for sponsoring and supporting this work. We thank the following donors who financially supported our whale shark tagging: the Sunbridge Foundation, MAC3 Impact Philanthropies, the Wolcott Henry Foundation, the owners and guests of the MV True North, Asia Coating Enterprise, M.E. Mali, D. Roozen, A. and S. Wong, E. Tan, S. Argyropolous, and D. Arnall.

**H.G.** acknowledges field assistance from Levan Mantell.

**F.H.V.H.** and **B.C.L.M.** research was funded by Coordenação de Aperfeiçoamento de Pessoal do Ensino Superior (CAPES- CIMAR II) and by Brazilian Council for Scientific and Technological Development (CNPq; 482557/2011-7).

**A.R.H.** research was funded by a grant from the Blake, Kymberly and George Rapier Foundation to the Galapagos Whale Shark Project (through Conservation International and the Galapagos Conservancy) and by the Galapagos Conservation Trust. Fieldwork was carried out under permits (PC-37-11 and MAE-PNG/CDS-2012-0020) issued by the Galapagos National Park Directorate.

**R.E.H.** acknowledges support and assistance of the Georgia Aquarium, Christopher Reynolds Foundation, National Geographic Society, NOAA, Mote Marine Laboratory, the Natural Protected Areas National Commission of Mexico (SEMARNAT/CONANP), and the UNDP/Global Environment Facility.

**B.M.N.** acknowledges the many supporters, funders, donors and volunteers of ECOCEAN Inc., without whom our long-term tagging programme could not have been conducted. We also thank

the Western Australian Department of Biodiversity Conservation and Attractions (formerly Department of Environment and Conservation and Department of Parks and Wildlife) and all involved in the whale shark ecotourism industry at Ningaloo Marine Park for their ongoing support of our work.

**S.D., C.A.R.** and **S.J.P.** thank the staff of Les Baleines Rand'eau and other tourism operators for their assistance, the Mada Megafauna team for practical and logistical support, and CNRO for their help with research permits in Madagascar.

**C.A.R** and **S.J.P.** thank Anthony Richardson, Scarla Weeks and Michael Bennett for support of tagging in Mozambique. This work was supported by two private trusts, the Shark Foundation, Aqua-Firma, Waterlust, a Rufford Small Grant and the PADI Foundation, and we thank all Marine Megafauna Foundation staff and volunteers for their assistance.

**D.R.-M.** acknowledges funding for tags provided by Save our Seas Foundation, Project Aware, WWF-Telcel, Royal Geographical Society through (EXERCISE JURASSIC SHARK 2), A1 scuba, downtown aquarium, Azul Marino Restaurant, Palapas Ventana. Whale Shark Mexico field work team.

**D.P.R.** acknowledges logistics for this project provided by the Qatar Ministry of Municipality and Environment (QMMOE) and, Maersk Oil Research and Technology Centre (MORTC). **S.B.** acted as an independent researcher throughout this study with financial support in the form of a salary from the MORTC. The MORTC or North Oil Company did not have any additional role in the study design, data collection and analysis, decision to publish, or preparation of the manuscript and only provided financial support in the form of SB's salary and research materials. **D.P.R.**'s work on this manuscript was supported by two small grants from the Save Our Seas Foundation. Many thanks to the Save Our Seas Foundation, Al Ghurair Foods and the Emirates Diving Association, Emirates Natural History Group and, Le Meridien Al Aqah Beach Resort for providing financial support for individual satellite tags. Christoph Rohner and Simon Pierce's contribution to this project were supported by private trusts, the Shark Foundation, Aqua-Firma, and Waterlust.

**A.M.M.S.** was supported by a Fellowship in Marine Conservation by the Pew Charitable Trusts and an ARC DP DP210103091.

**M.S.S.** and **B.W.M.** acknowledge financial support provided by Guy Harvey Ocean Foundation.

**M.T.** and **M.G.M.** acknowledge the generous support of Apache Energy Ltd (now Santos Ltd). AIMS acknowledges the Yinggarda, Baiyungu and Thalanyji Traditional Owners of Country adjacent to where this whale shark research was undertaken. We recognise these People's ongoing spiritual and physical connection to Country and pay our respects to the Aboriginal Elders past, present and emerging.

**C.R.P.** acknowledges funding from the Secretariat for Higher Education, Science, Technology and Innovation of the Ecuadorean Government; Holsworth Wildlife Research Endowment; Blake, Kimberly and George Rapier Charitable Trust; WWF Prince Bernard Scholarships; Lindblad Expeditions and the Galapagos Conservancy. Fieldwork was carried out under permit number MAE-DPNG PC-60-13.

## 8. Details of ethical compliance and approvals

All animal handling and tagging procedures were completed by trained personnel under permissions granted by institutional ethical review bodies and complied with all relevant ethical regulations in the jurisdictions in which they were performed. Details from individual research teams are given below with initials of lead investigators in bold.

Research in the Philippines was performed in collaboration with the respective Regional Offices of the Department of Environment and Natural Resources, the Department of Agriculture-Bureau of Fisheries and Aquatic Resources and the Palawan Council for Sustainable Development (Wildlife Gratuitous Permit 2017-13). All research in Tubbataha Reefs Natural Park was done in collaboration with the Tubbataha Management Office (**G.A., J.L.** and **S.J.P.**).

Whale sharks were tagged in Queensland with approval from James Cook University Animal Ethics Committee (ID - A2649) (**A.B.**).

Research was carried out under the general auspices of King Abdullah University of Science and Technology's (KAUST) arrangements for marine research with the Saudi Arabian Coast Guard and the Saudi Arabian Presidency of Meteorology and Environment. These are the relevant Saudi Arabian authorities governing all sea-going research actions in the Saudi marine environment. KAUST has negotiated a general and broad permission for marine research in Saudi Arabian Red Sea waters with these two agencies and thus there is no permit number to provide. The animal use protocol was performed in accordance with Woods Hole Oceanographic Institution's Animal Care and Use Committee (IACUC) protocol #16518 and approved by KAUST's Biosafety and Ethics Committee (**M.L.B.** and **C.D.B.**).

Research off Colombia was carried out according to Migramar animal ethical statements under permits from the Environmental Ministry of Colombia and the National Parks of Colombia (**S.B-L.**).

Research was approved by the Research and Animal Care Committee at Georgia Aquarium and carried out under permit (2019-SRE-01) from the St Helena Government (**A.D.M.D.**).

Tagging procedures were approved by the Cendrawasih Bay National Park Authority and are in accordance with the protocols established by Conservation International Indonesia's animal ethics review committee (**A.B.S., A.H.** and **M.V.E.**).

All procedures involving research animals were approved by the Smithsonian Institution's Institutional Animal Care and Use Committee (IACUC) (**H.G.**).

All tagging procedures were performed under a research license provided by the Brazilian Environmental Agency- Chico Mendes Institute for the Conservation of Biodiversity (ICMBio), Ministry of the Environment, under permit SISBIO/ Nº 14.124; and under the permit CEUA/Nº 044, by the Committee of Ethics and Use of Animals, of Universidade Federal Rural de Pernambuco-UFRPE (**F.H.V.H**. and **B.C.L.M**.).

All procedures performed in studies involving animals were in accordance with the ethical standards of the University of California Davis, under Institutional Animal Care and Use Committee (IACUC) Protocol #16022, and with permission from the authority of the Galapagos Marine Reserve in the figure of Permit PC-37-11 from the Galapagos National Park Directorate (**A.R.H.**).

All procedures involving research animals were approved by Mote Marine Laboratory's Institutional Animal Care and Use Committee (IACUC) (**R.E.H.**).

All tagging was conducted under animal ethics approvals from Murdoch University (permit numbers: W2058/7; W2402/11; R2926/17) and the University of Queensland (permit number:

SBS/085/18/WA/INTERNATIONAL). In addition, permits to conduct fieldwork on wildlife in Western Australian were issued from the Western Australian Department of Environment and Conservation (DEC) (permit numbers: SF007471; SF007949; SF008572), Department of Parks and Wildlife (DPaW) (permit numbers: SF009184; SF009897; SF010414; SF010781; 08-000533-2; 08-002082-2) and Department of Biodiversity Conservation and Attractions (DBCA) (permit numbers: FO25000033-4; FO25000033-9) (**B.M.N.**).

Research was conducted with the approval of and in partnership with the Centre National de Recherches Océano- graphiques (CNRO) in Madagascar (**S.D.** and **S.J.P.**).

In Mozambique, no animal was restrained, caught or removed from its natural habitat for the purpose of this study. Whale shark tagging was compliant with ethics guidelines from the University of Queensland's Animal Ethics Committee and was conducted under their approval certificate GPEM/186/10/MMF/WCS/SF (**C.A.R.** and **S.J.P.**).

Research was carried out under the general auspices of relevant Mexican authorities governing all research actions on wildlife and protected animals and areas in Mexico: CONACYT (Consejo Nacional de Ciencia y Tecnología), DGVS (Dirección General de Vida Silvestre), and SEMARNAT (Secretaría del Medio Ambiente y Recursos Naturales) (**D.R.-M.**).

Permissions for fieldwork and data collection on whale sharks in the Al Shaheen region of Qatar were given by the Qatar Ministry of Environment with whom this work was conducted (**D.P.R.**, **M.Y.J.** and **S.S.B.**).

Seychelles work was approved by the Ministry of Environment and in Djibouti the work was permitted under the Ministry of Tourism through the local non-governmental organization DECAN (**D.R.L.R.**).

All procedures were performed under Mexico Government Permits N° SGPA/DGVS/006539/18, N° SGPA/DGVS/5273/19 and Nova Southeastern University IACUC Permit 2017.11.MS1-A1 (**M.S.S.** and **B.M.W.**).

All procedures were approved by either the University of Western Australia, University of Adelaide, South Australia, or Charles Darwin University Animal Ethics Committees (**M.G.M.** and **M.T.**).
