# Supplementary material for: Climate-driven global redistribution of an ocean giant predicts increased threat from shipping
Source: Nat Clim Chang. 2024 Oct 7;14(12):1282–91. doi: 10.1038/s41558-024-02129-5 (PMC11618081; doi:10.1038/s41558-024-02129-5)
Supplement: Supplementary file 2 — Reporting Summary [file 41558_2024_2129_MOESM2_ESM.pdf]

nature portfolio

Corresponding author(s): Freya Womersley

Last updated by author(s): 2024/08/05

Reporting Summary

Nature Portfolio wishes to improve the reproducibility of the work that we publish. This form provides structure for consistency and transparency in reporting. For further information on Nature Portfolio policies, see our Editorial Policies and the Editorial Policy Checklist.

Statistics

For all statistical analyses, confirm that the following items are present in the figure legend, table legend, main text, or Methods section.

n/a	Confirmed	
☐	☒	The exact sample size (*n*) for each experimental group/condition, given as a discrete number and unit of measurement
☐	☒	A statement on whether measurements were taken from distinct samples or whether the same sample was measured repeatedly
☐	☒	The statistical test(s) used AND whether they are one- or two-sided<br>*Only common tests should be described solely by name; describe more complex techniques in the Methods section.*
☐	☒	A description of all covariates tested
☐	☒	A description of any assumptions or corrections, such as tests of normality and adjustment for multiple comparisons
☐	☒	A full description of the statistical parameters including central tendency (e.g. means) or other basic estimates (e.g. regression coefficient) AND variation (e.g. standard deviation) or associated estimates of uncertainty (e.g. confidence intervals)
☐	☒	For null hypothesis testing, the test statistic (e.g. *F*, *t*, *r*) with confidence intervals, effect sizes, degrees of freedom and *P* value noted<br>*Give P values as exact values whenever suitable.*
☐	☒	For Bayesian analysis, information on the choice of priors and Markov chain Monte Carlo settings
☒	☐	For hierarchical and complex designs, identification of the appropriate level for tests and full reporting of outcomes
☒	☐	Estimates of effect sizes (e.g. Cohen's *d*, Pearson's *r*), indicating how they were calculated

Our web collection on statistics for biologists contains articles on many of the points above.

Software and code

Policy information about availability of computer code

Data collection	Custom code developed to calculate the ship co-occurrence index (SCI) is available on GitHub at (ref.82 from the article) with examples based on the derived open access datasets needed to reproduce the results.
Data analysis	R and GIS

For manuscripts utilizing custom algorithms or software that are central to the research but not yet described in published literature, software must be made available to editors and reviewers. We strongly encourage code deposition in a community repository (e.g. GitHub). See the Nature Portfolio guidelines for submitting code & software for further information.

Data

Policy information about availability of data

All manuscripts must include a data availability statement. This statement should provide the following information, where applicable:

- Accession codes, unique identifiers, or web links for publicly available datasets
- A description of any restrictions on data availability
- For clinical datasets or third party data, please ensure that the statement adheres to our policy

Environmental data are available at https://data.marine.copernicus.eu/products. CMIP6 data are available at https://esgf-ui.ceda.ac.uk/cog/search/cmip6-ceda/. Shipping data are available upon request to Global Fishing Watch (https://www.globalfishingwatch.org). OBIS and SharkBook whale shark observation data are available at https://obis.org (open) and https://www.sharkbook.ai/ (upon request), respectively. AquaMaps data are available at https://www.aquamaps.org. EEZ

boundary data are available at https://www.marineregions.org/downloads.php. LME boundary data are available at https://github.com/datasets/lme-large-marine-ecosystems/. Land boundary data are available at https://www.naturalearthdata.com. IUCN boundary data are available at https://www.iucnredlist.org/ja/species/19488/2365291. Derived whale shark habitat suitability maps for the present day and future are available on GitHub (see ref. 82 in the article).

Research involving human participants, their data, or biological material

Policy information about studies with human participants or human data. See also policy information about sex, gender (identity/presentation), and sexual orientation and race, ethnicity and racism.

Reporting on sex and gender	*Use the terms sex (biological attribute) and gender (shaped by social and cultural circumstances) carefully in order to avoid confusing both terms. Indicate if findings apply to only one sex or gender; describe whether sex and gender were considered in study design; whether sex and/or gender was determined based on self-reporting or assigned and methods used. Provide in the source data disaggregated sex and gender data, where this information has been collected, and if consent has been obtained for sharing of individual-level data; provide overall numbers in this Reporting Summary. Please state if this information has not been collected. Report sex- and gender-based analyses where performed, justify reasons for lack of sex- and gender-based analysis.*
Reporting on race, ethnicity, or other socially relevant groupings	*Please specify the socially constructed or socially relevant categorization variable(s) used in your manuscript and explain why they were used. Please note that such variables should not be used as proxies for other socially constructed/relevant variables (for example, race or ethnicity should not be used as a proxy for socioeconomic status). Provide clear definitions of the relevant terms used, how they were provided (by the participants/respondents, the researchers, or third parties), and the method(s) used to classify people into the different categories (e.g. self-report, census or administrative data, social media data, etc.) Please provide details about how you controlled for confounding variables in your analyses.*
Population characteristics	*Describe the covariate-relevant population characteristics of the human research participants (e.g. age, genotypic information, past and current diagnosis and treatment categories). If you filled out the behavioural & social sciences study design questions and have nothing to add here, write "See above."*
Recruitment	*Describe how participants were recruited. Outline any potential self-selection bias or other biases that may be present and how these are likely to impact results.*
Ethics oversight	*Identify the organization(s) that approved the study protocol.*

Note that full information on the approval of the study protocol must also be provided in the manuscript.

Field-specific reporting

Please select the one below that is the best fit for your research. If you are not sure, read the appropriate sections before making your selection.

☐ Life sciences ☐ Behavioural & social sciences ☒ Ecological, evolutionary & environmental sciences

For a reference copy of the document with all sections, see nature.com/documents/nr-reporting-summary-flat.pdf

Ecological, evolutionary & environmental sciences study design

All studies must disclose on these points even when the disclosure is negative.

Study description	The study used a dataset of tracked whale sharks (n = 348) tagged in seven large scale ocean regions alongside freely available datasets of oceanographic variables and global climate models from the Coupled Model Intercomparison Phase 6 (CMIP6). Distribution models were developed to (i) generate a first order approximation of global habitat suitability and (ii) project the distribution of whale sharks in two future decades under three mitigation scenarios. These were then used to (iii) assess habitat changes and horizontal co-occurrence with shipping traffic based on data provided by Global Fishing Watch.
Research sample	The study used a dataset of tracked whale shark movements (n = 348). Shipping vessels were tracked using Automatic Identification Systems and provided by Global Fishing Watch. Environmental data was sourced from free online databases.
Sampling strategy	Dedicated field campaigns undertaken by researchers involved in the Global Shark Movement Project. Tagging was undertaken by multiple different research groups across many countries with tagging procedures approved by institutional ethical boards and conforming to national regulations.
Data collection	Each research group collected shark track data independently by download from the ARGOS satellite service provider.
Timing and spatial scale	2005-2019, 2046-2055, 2086-2095, global with regional subsets.
Data exclusions	Animal locations that were recorded after December 2019 (due to lags in environmental data availability) or that were deemed erroneous due to technology failure or early detachment (determined on a case-by-case basis using an algorithm to detect transmissions indicative of a floating device as opposed to one attached to the animal) were removed from the dataset.
Reproducibility	No experiments as such were conducted, rather our data are based on satellite tracked movements of individual whale sharks,

Reproducibility	associated environmental conditions and shipping activity.
Randomization	Individual whale sharks were allocated into groups based on the geographic positioning of their tracked movements. Where randomization procedures were used they are fully described in the Methods and Supplementary Information.
Blinding	Blinding is not relevant to this type of study because data are based on movements of wild animals and shipping vessels.

Did the study involve field work? ☒ Yes ☐ No

Field work, collection and transport

Field conditions	Tags were deployed on pelagic sharks in the Atlantic, Pacific and Indian Oceans under a range of conditions.
Location	Locations of tagging and subsequent tracks of sharks are detailed in the paper.
Access & import/export	No collections or import or export of samples was undertaken.
Disturbance	Disturbance to individual shark behaviour was minimised through completion of tagging procedures within a few minutes if captured, or during free swimming. All procedures were approved by institutional and national ethical review committees.

Reporting for specific materials, systems and methods

We require information from authors about some types of materials, experimental systems and methods used in many studies. Here, indicate whether each material, system or method listed is relevant to your study. If you are not sure if a list item applies to your research, read the appropriate section before selecting a response.

Materials & experimental systems

n/a	Involved in the study	
☒	☐	Antibodies
☒	☐	Eukaryotic cell lines
☒	☐	Palaeontology and archaeology
☐	☒	Animals and other organisms
☒	☐	Clinical data
☒	☐	Dual use research of concern
☒	☐	Plants

Methods

n/a	Involved in the study	
☒	☐	ChIP-seq
☒	☐	Flow cytometry
☒	☐	MRI-based neuroimaging

Animals and other research organisms

Policy information about studies involving animals; ARRIVE guidelines recommended for reporting animal research, and Sex and Gender in Research

Laboratory animals	N/A
Wild animals	A total of 348 whale sharks were tagged with satellite-linked transmitters between 2005 and 2019 at numerous sites within the Atlantic, Indian and Pacific Oceans. This included 39 individuals tagged in the north Atlantic, 14 in the south Atlantic, 44 in the northwest Indian Ocean, 26 in the southwest Indian Ocean, 74 in the east Indian Ocean, 62 in the west Pacific and 89 in the east Pacific. Tags were attached anterior to the first dorsal fin region (usually with tethers) or mounted onto the fin of free-swimming whale sharks or whale sharks captured in bagan lift-net fisheries by trained personnel. All animal-handling procedures were approved by institutional ethical review committees and carried out in accordance with laws of the countries where they were undertaken. No animals were killed as part of the study.
Reporting on sex	Both sexes were included in the analysis with a total of 106 females and 165 males plus 77 of unknown sex, ranging from 3 to 13.1 m total length. Sex was determined by trained personnel.
Field-collected samples	N/A
Ethics oversight	All animal-handling procedures were approved by institutional ethical review committees and carried out in accordance with laws of the countries where they were undertaken with a full list provided in the Supplementary Information document associated with the article.

Note that full information on the approval of the study protocol must also be provided in the manuscript.

Plants

Seed stocks

Report on the source of all seed stocks or other plant material used. If applicable, state the seed stock centre and catalogue number. If plant specimens were collected from the field, describe the collection location, date and sampling procedures.

Novel plant genotypes

Describe the methods by which all novel plant genotypes were produced. This includes those generated by transgenic approaches, gene editing, chemical/radiation-based mutagenesis and hybridization. For transgenic lines, describe the transformation method, the number of independent lines analyzed and the generation upon which experiments were performed. For gene-edited lines, describe the editor used, the endogenous sequence targeted for editing, the targeting guide RNA sequence (if applicable) and how the editor was applied.

Authentication

Describe any authentication procedures for each seed stock used or novel genotype generated. Describe any experiments used to assess the effect of a mutation and, where applicable, how potential secondary effects (e.g. second site T-DNA insertions, mosiacism, off-target gene editing) were examined.
